# Systematic optimization of fragment TLX ligands towards agonism and inverse agonism

**DOI:** 10.1021/acs.jmedchem.5c02718

**Published:** 2026-01-28

**Authors:** Emily C. Hank, Loris Knümann, Úrsula López-García, Arthur Kardanov, Vasily Morozov, Georg Höfner, Daniel Merk

**Affiliations:** 1 Ludwig-Maximilians-Universität München, Department of Pharmacy, 81377 Munich, Germany

## Abstract

The transcription factor tailless homologue (TLX, NR2E1) maintains persistence of neural stem cells (NSC) in a proliferating, undifferentiated state thereby controlling NSC homeostasis and enabling neurogenesis. TLX is responsive to small molecule ligands offering potential access to new neuroprotective treatments, but TLX ligands are very rare. Here, we used a drug fragment screening hit as lead to develop TLX modulators and identified substructures tuning activity between agonism and inverse agonism. Structural optimization provided potent TLX activating and inhibiting fragment ligands with validated binding and favorable ligand efficiency for structural extension.

## Introduction

The human tailless homologue receptor (TLX, NR2E1) is an orphan nuclear receptor (NR) with a peculiar repressor function in neural stem cells (NSC) and neural progenitors (NP), enabling their proliferation and self-renewal.^1,2^ It binds co-repressors such as atrophin^3^ and lysine-specific demethylase 1 (LSD1)^4^ and recruits histone deacetylases^5^ to suppress transcription of key cell cycle and proliferation regulators, such as p21 and the phosphatase and tensin homologue (pten).^3,5,6^ This ability of TLX to control the expression of tumor suppressor genes suggests a critical role in the regulation of NSC proliferation, cell cycle progression and differentiation.^2,5,7^ Accordingly, studies in mouse embryos have demonstrated that TLX-deficiency leads to premature NSC differentiation and cell cycle alterations.^8,9^ TLX-expressing cells isolated from adult mouse brains have been demonstrated to multiply, regenerate and differentiate in vitro while these abilities were lacking in cells isolated from TLX-null brains, but could be rescued by exogenous TLX expression.^2^ Moreover, isolated TLX-positive murine cells in culture can yield NSC in both activated or quiescent states while loss of TLX increases the amount of non-proliferating NSC, supporting the NR’s involvement in NSC activation.^6^

TLX is also critical in brain development since NR2E1-deletion in mice reduced dendritic length and arborization^13^ and caused thinning of the neocortical layers^14^ as well as an overall shrinkage of cerebral structures, including the limbic system, olfactory bulbs^14–16^ and forebrain^17^. Phenotypic observations of TLX germline deletion models included aggressive and violent behavior^8,16,18,19^, hyperactivity^20,21^ and decreased anxiety and fear conditioning^8,21,22^, potentially caused by impaired memory function.^8,20,22^ Conversely, animal models of TLX-overexpression have demonstrated improved NSC self-renewal^23,24^ and enhanced neurogenesis after stroke^25^, along with increased anterior brain mass and elevated learning and memory capacity.^23^ Outside the brain, TLX expression has been detected in retinal progenitor cells, where loss of TLX was found to cause retinal and optic nerve deficiencies and affect vision._16,26,27_

**Chart 1:**
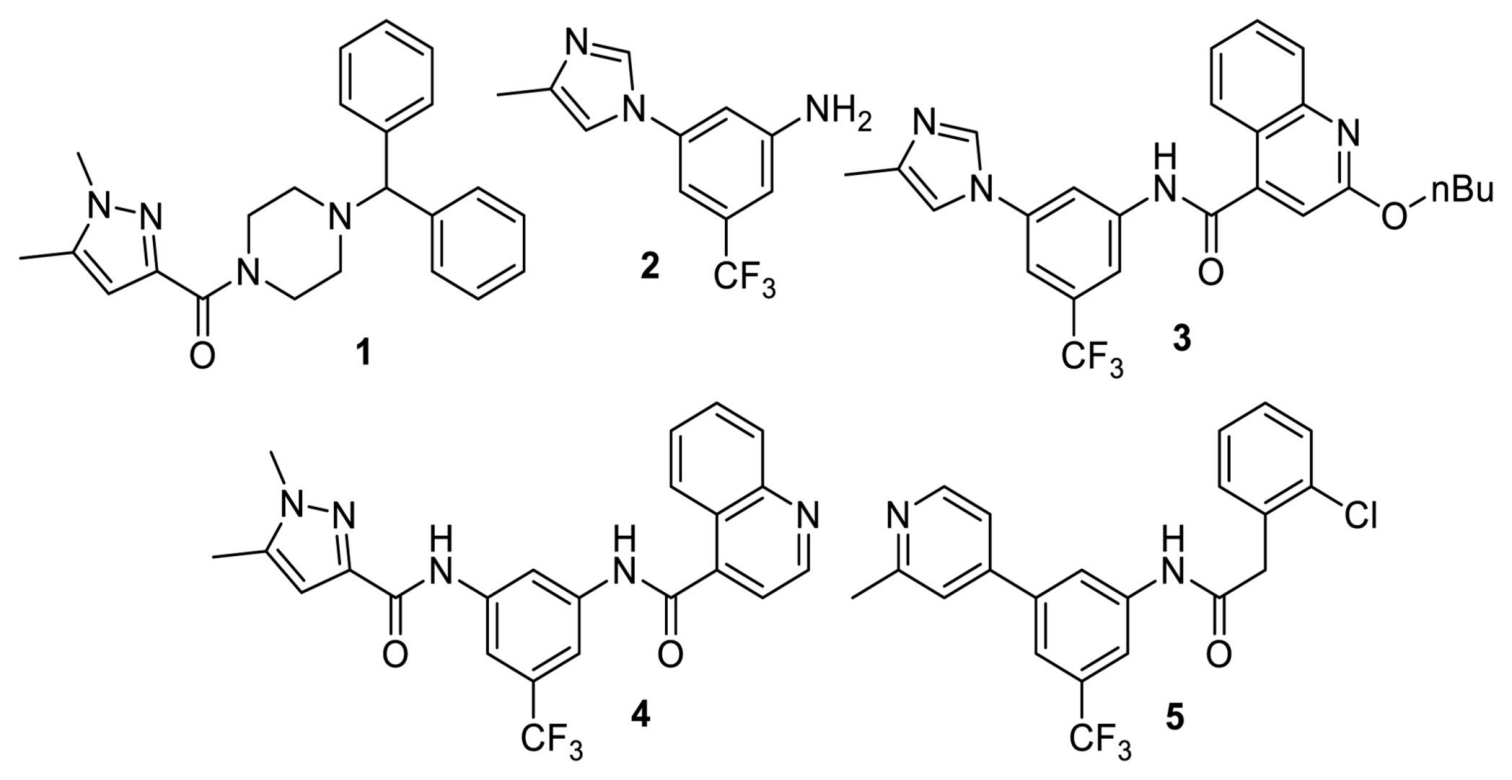
TLX agonists^10–12^

These lines of evidence support therapeutic potential of TLX in the context of neurodegenerative diseases by stimulating neurogenesis and counteracting neuroinflammation^23,28–32^, in retinal dystrophies^3^, and in mental disorders.^19,33,34^ However, effects of pharmacological TLX modulation have not been evaluated due to a lack of potent, selective and well-characterized TLX ligands.^32^ The receptor’s druggability was first explored in 2014 by a DSF-based fragment screening for binders of the TLX ligand binding domain (LBD), which led to the identification of three ligands, including ccrp2 (**1**, Chart 1, K_d_ = 0.65 µM).^10^ A few further TLX ligand scaffolds have been discovered since (reviewed by Lewandowski et al. ^35^), but high-quality chemical tools for target validation of TLX are still very rare. We have identified the fragment-like TLX agonist **2** (EC_50_ = 20 µM) by drug fragment screening.^11,36^ Extension of **2** and preliminary optimization offered access to the TLX agonists **3**-**5** displaying submicromolar potencies^11,12^, underscoring the potential of **2** as lead for TLX modulator development. The SAR of the fragment-like **2** has not been systematically studied, however. Here, we report extensive SAR elucidation of the TLX ligand chemotype of **2** and marked optimization on fragment level via systematic structural modification. Interestingly, removal of a key polar feature in the heterocyclic motif inverted activity and offered access to inverse TLX agonists.

## Results & Discussion

Aiming to increase potency (i.e., improve the EC_50_ value) and efficacy (i.e., enhance repression as observed by lower remaining reporter activity) of the fragment hit **2**, we commenced SAR elucidation by testing importance of the methylimidazole motif and by scanning for opportunities for structural extension (Table 1). Removal of the methyl group (**6**) enhanced potency by 5-fold and improved efficacy while further removal of the distal nitrogen in the pyrrole analogue **7** was not favored suggesting importance of the polar feature. The imidazole regiochemistry of **2** was preferred as evident from diminished TLX agonism of the isomers **8**-**10**. Replacement of the methylimidazole (**2**) by a 1-methyl-1*H*-pyrazole-4-yl motif (**11**) was tolerated while the alternative pyrazole regiochemistries of **12**-**15** decreased TLX agonism, further pointing to a key role of polarity distribution in this ring. Removal of the methyl substituent from the preferred pyrazole (**11**) in **16** was tolerated and enhanced efficacy while introduction of an additional methyl group in 5-position (**17**) improved potency thus indicating that the scaffold could be expanded in this region. Indeed, extension of the original imidazole (**2**) to the corresponding benzimidazole (**18**) was accompanied by an almost tenfold improvement in potency. The indole-1-yl derivative **19** failed to activate TLX while the inverted analogue **20** retained TLX agonism providing further evidence that the distal nitrogen was critical.

Based on these results, we continued SAR elucidation of **2** by exploring pyridine as alternative polar heterocycle to replace the imidazole (Table 2). The methylimidazole of **2** was mimicked well by a 2-methyl-4-pyridyl motif (**21**) and a 6-methyl-3-pyridyl motif (**22**) with the former being slightly more active. In both cases, the removal of the methyl group (**23, 24**) had no major impact on TLX agonism while double methylation (**25, 26**) boosted potency. Replacement of the (mono)methyl substituents by an acetyl substituent (**27, 28**) was tolerated and might be a valuable modification to improve polarity. Enhancing polarity within the ring by incorporation of a second nitrogen atom in the pyrimidin-5-yl derivative **29** was favored while the hydrophobic dimethylphenyl analogue **30** of the most active dimethylpyridyl derivative **25** interestingly displayed an inverted activity profile and acted as weak inverse TLX agonist.

These informative initial insights supported an SAR model for TLX agonism of the scaffold in which a polar (nitrogen) atom seemed critical in the distal region (i.e., meta/para to the trifluoromethylaniline core) but was not tolerated in other positions. Removal of this polar center disrupted activity or resulted in inverse agonism. Hydrophobic substituents surrounding the polar center were mostly favored and could valuably enhance potency. Especially installation of an additional aromatic ring (**18** vs. **2**) markedly increased TLX agonism prompting us to explore further two-ring systems in this region (Table 3).

Comparing the isomeric *N*-methylindol-3-yl (**20**), *N*-methylindol-4-yl (**31**) and *N*-methylindol-5-yl (**32**) derivatives revealed a strong preference in terms of potency for the indol-5-yl (**32**) regiochemistry that was also evident - though less pronounced - in the demethylated analogues (**33, 34**). The 2,3-dihydroindole **35** retained a similar TLX agonist profile as **34** indicating that aromaticity was not critical, but evaluation of the corresponding benzofuran (**36**), benzothiophene (**37**) and imidazopyridine (**38**) derivatives revealed a marked preference for nitrogen-containing motifs.

Structural fusion of the preferred pyridine (**21**/**25**) and *N*-methylindol-5-yl (**32**) motifs by incorporating an additional nitrogen atom in 7-position of the indole (**39**) yielded the first fragment TLX agonist with sub-micromolar potency. Introduction of an additional nitrogen atom in 2-position of the *N*-methylindol-5-yl motif (**40**) and inversion of **39** to the pyrrolo[2,3-b]pyridine-3-yl analogue **41** resembling the geometry of **18** were less favored.

After substantially improving the TLX agonist profile of the fragment hit **2** via modification of the methylimidazole region, we moved our attention to the trifluoromethylaniline core of the scaffold. Using the favored 2-methylpyridin-4-yl substituent (**21**) as fixed motif in 3-position we varied the substitution pattern on the aniline motif (Table 4). Removal of the trifluoromethyl group (**42**) was detrimental in terms of TLX activation efficacy and shifting it from 5- (**21**) to 4-position (**43**) diminished TLX agonist potency highlighting importance of the 5-substituent for potent and efficient TLX activation. Alternative groups in 5-position pointed to a preference for electron withdrawing groups with the 5-methyl (**44**) and 5-methoxy (**45**) derivatives displaying reduced potency and efficacy, while the trifluoromethoxy (**46**), cyano (**47**) and chloro (**48**) substituents were more favored. Nevertheless, the original trifluoromethyl group (**21**) remained superior. 4,5-Dichlorosubstitution (**49**) retained a similar activity profile as **21** suggesting that also larger motifs might be tolerated. Therefore, we tested incorporation of a small heterocycle to mimic the 4,5-disubstitution and indeed observed enhanced TLX agonism of the indole analogue **50** while the regioisomer **51** was inactive. The aniline core motif presented an overall rather strict SAR with few opportunities for improvement and only the indole motif of **50** emerged as a potential alternative to the original trifluoromethyl substituent of **21**. The fused compound **52** combining the indole of **50** and the favored 1-methyl-1*H*-pyrrolo[2,3-b]pyridin-5-yl substituent of the most active agonist **39** displayed potent TLX agonism thus supporting suitability of the alternative core scaffold for combination with other preferred modifications. Nevertheless, **52** failed to outmatch the most active agonist **39** bearing the original trifluoromethylaniline core.

Systematic SAR evaluation of the fragment-like TLX modulator scaffold of **2** enabled substantial improvement in potency via modification or replacement of the methylimidazole residue. The resulting optimized fragment TLX ligands may serve as valuable leads for structural extention. Additionally, the structure-activity data revealed that removal of the distal polar center which appeared critical for agonism could invert activity to modest TLX inhibition. Ligands blocking the repressor activity of TLX, i.e., inverse agonists, are very rare but would be valuable as complementary chemical tools to study the receptor’s biological roles. Therefore, we set out to explore the SAR around **30** for molecular determinants driving the observed activity switch (Table 5).

The methylation pattern of **30** turned out to be critical for inverse agonism (**53**-**55**) with a methyl substituent being favored in 3-position (**54**) of the phenyl motif over the 2- (**53**) and especially the 4-position (**55**). Double methylation in 2-/3-positions (**56**) was superior to the 3,4- (**57**) and 3,5- (**30**) dimethyl analogues indicating an overall preference for 3-substitution. Bulkier hydrophobic groups (ethyl (**58**), *tert*-butyl (**59**)), an acetyl substituent (**60**) and halogen atoms (fluorine (**61**), chlorine (**62**)) were not tolerated. The 3-trifluoromethyl (**63**) and trifluoromethoxy (**64**) analogues retained inverse TLX agonism but with reduced potency. Only a 3-methoxy substituent (**65**) and a 3-dimethylamino motif (**66**) achieved improved inverse TLX agonism. The more polar 3-hydroxy substituent (**67**) inverted the activity profile back to TLX agonism.

With this strict SAR for inverse TLX agonists based on **30**, we next explored scaffold-hopping to bi- and heterocyclic motifs (Table 6). The activities observed for **53**-**67** indicated that only hydrophobic groups maintained inverse agonist potency and that substitution was favored in 3-position. In line with this preferred geometry, the α-naphthyl (**68**) but not the β-naphthyl (**69**) derivative acted as inverse TLX agonist, and also the (di)methylthiophene analogues **70**-**72** mimicking the favored 3-tolyl derivative **54** displayed robust inhibition of TLX activity - even with improved efficacy. Replacement of the methyl group by an acetyl motif (**73, 74**), which had been tolerated in the agonist series (**27, 28**), inverted the activity profile to rather potent TLX activators. The same effect was evident for other polar substituents such as the ester (**75**) and amide (**76, 77**) analogues which exhibited TLX agonism, and also the incorporation of a nitrogen atom in the thiazoles **78**-**80** inverted or disrupted activity thus corroborating the hypothesis that polarity was not tolerated for inverse agonist activity.

**Figure 1:**
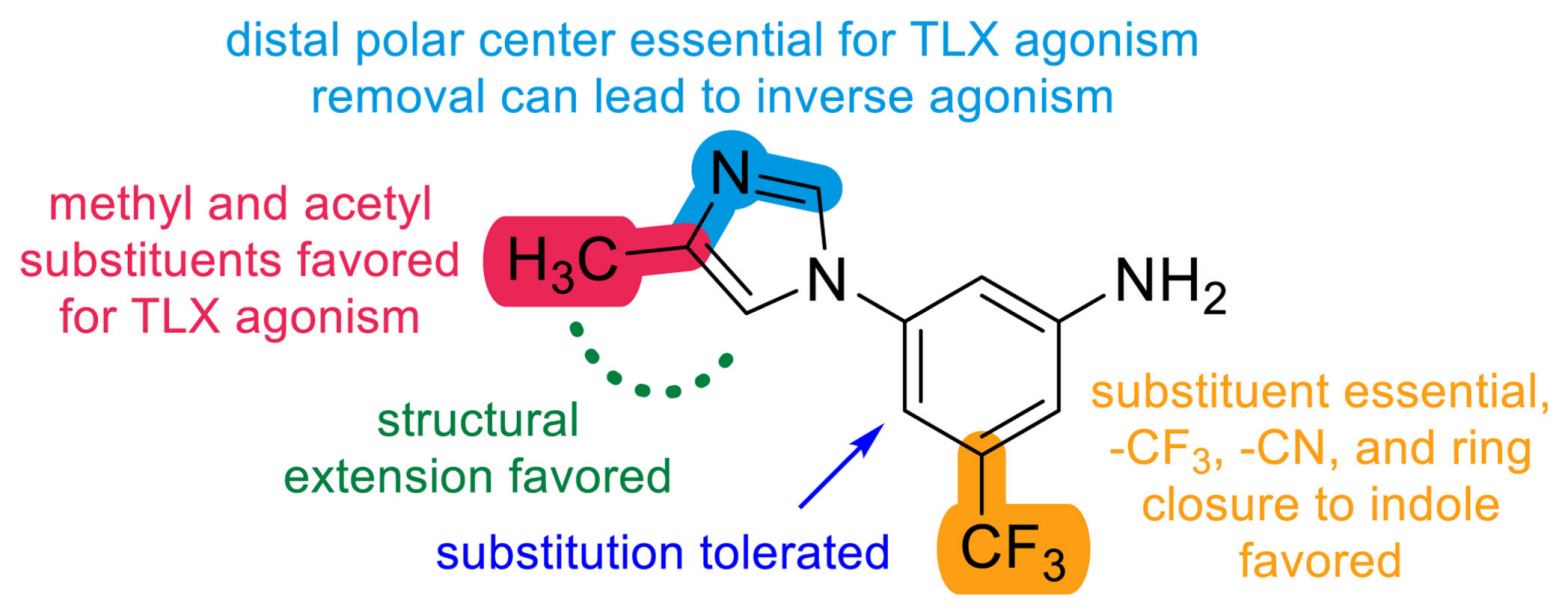
Summarized SAR for fragment TLX modulators.

Systematic SAR analysis of the scaffold of **2** revealed key features for bidirectional TLX modulation (Figure 1). The methylimidazol could be replaced by various mono- and bicyclic aromatic systems wherein 1-2 distal polar centers were essential for potent TLX agonism and methyl or acetyl substituents on the aromatic system were favored. Replacement by purely hydrophobic aromatic systems could invert the activity profile to inverse agonism, but inverse agonists displayed a more strict SAR. Modifications in the 5-trifluoromethylaniline core were less productive. The substituent in 5-position turned out to be essential but the trifluormethyl group could be replaced without full loss of activity with a preference for electron withdrawing motifs. Substitution in 4-position was tolerated but only an additional ring (i.e., indole) enhanced potency.

Within the series of fragment-like TLX modulators derived from **2**, the 2,6-dimethylpyridin-4-yl (**25**), 2-acetylpyridin-4-yl (**27**) and 1-methyl-1*H*-pyrrolo[2,3-*b*]pyridin-5-yl (**39**) derivatives evolved as the most favored agonists with low micromolar to sub-micromolar potencies (Table 7, dose-response curves in Figure S2). Favorable ligand efficiency (LE) and lipophilic ligand efficiency (LLE) as well as sufficient aqueous solubility support their suitability as leads for structural extension. Additionally, the 3-methoxyphenyl (**65**) and 3,5-dimethylthiophen-2-yl (**72**) residues emerged as motifs to obtain inverse agonists (dose response curves in Figure S2). Though slightly less potent and more hydrophobic, these fragments are also highly valuable starting points for medicinal chemistry as TLX inhibitors are very rare.

**Figure 2:**
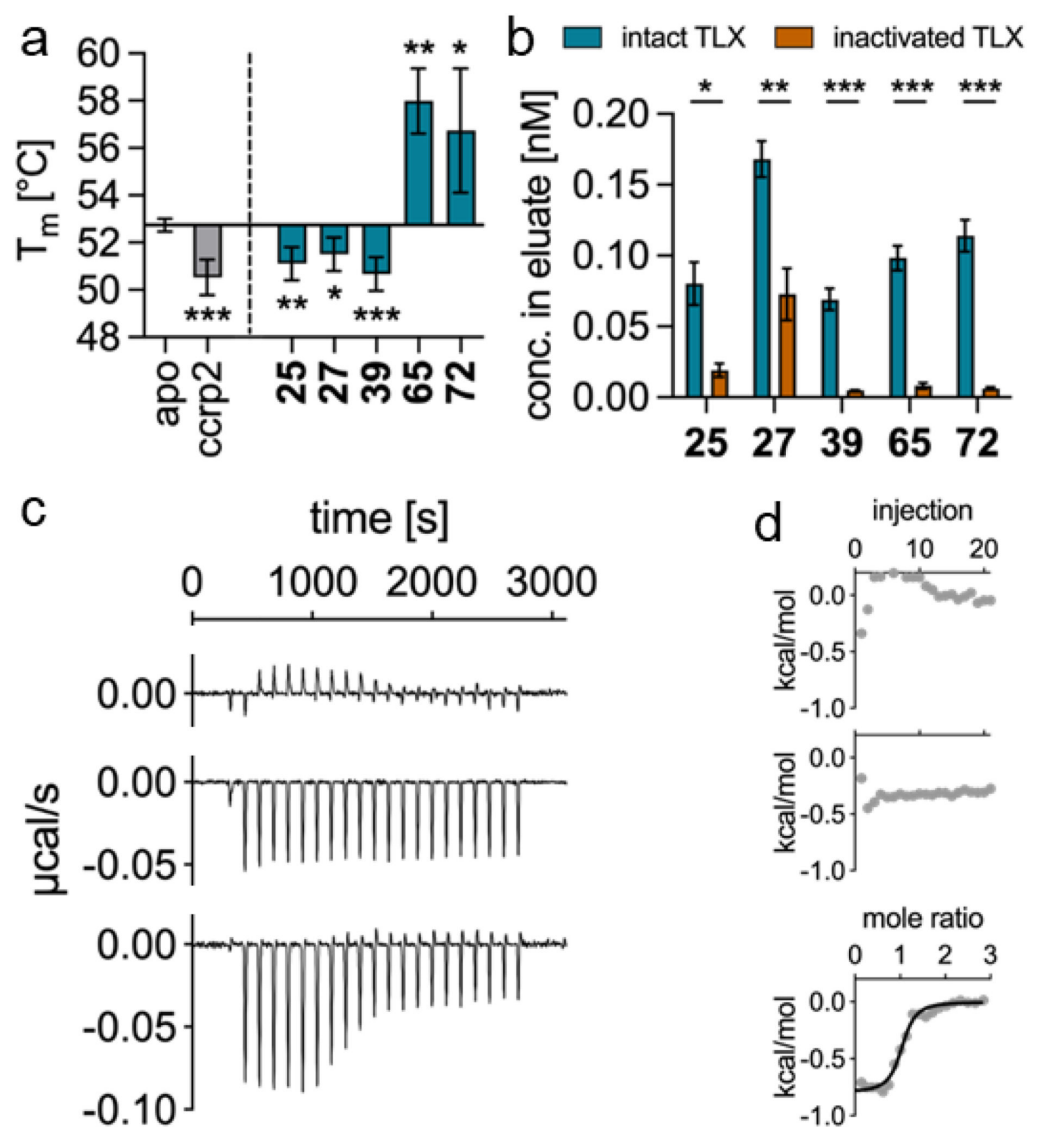
Validation of direct binding of **25, 27, 39, 65** and **72** to the recombinant TLX LBD. (a) DSF assay. The reference agonist ccrp2^10^ was used at 100 µM, the fragments at 300 µM with 2 µM TLX. N=3; * p<0.05, ** p<0.01, *** p<0.001 (one-way ANOVA with Dunnett’s multiple comparisons test). (b) Affinity-selection-mass-spectrometry (ASMS, Figure S3). The test compounds **25, 27** and **39** were used at 1 µM with 35 µM TLX LBD. **65** and **72** were used at 10 µM with 50 µM TLX LBD. N = 9 (intact), N=3 (inactivated); * p<0.05, ** p<0.01, *** p<0.001 (t-test). (c,d) Isothermal titration calorimetry (ITC) of the **39**-TLX LBD interaction (K_d_ 1.1±0.1 µM). Representative buffer-TLX LBD (top), **39**-buffer (middle) and **39**-TLX LBD titrations (bottom) are shown in (c) and the corresponding heats and fit are shown in (d).

For validation of direct TLX modulation, we employed two orthogonal binding assays using the recombinant TLX LBD. Differential scanning fluorimetry (DSF, Figure 2a) revealed effects of all favored fragments on thermal stability of TLX. As observed previously for the TLX agonists ccrp2 (**1**)^10^ and the lead **2**^36^ as well as for other nuclear receptors^39^, the TLX agonists **25, 27** and **39** diminished the melting temperature of the protein. Interestingly, the inverse agonists **65** and **72** mediated the opposite effect and caused a positive thermal shift of the TLX LBD indicating that agonism and inverse agonism induced different structural/conformational effects and might differently affect dimerization. The absolute effects on the melting temperature aligned with the respective rank-order of potency for agonists and inverse agonists adding further confidence to the binding results.

Additionally, we validated binding of the fragments by affinity-selection-mass-spectrometry (ASMS) as second binding assay (Figure 2b; schematic method illustration in Figure S3). TLX LBD protein (35-50 µM) was incubated with the agonists (1 µM) or inverse agonists (10 µM), separated from unbound ligands by size-exclusion chromatography (SEC), and denaturated to release the bound ligands for quantification by LC-MS/MS. The same procedure was repeated with heat inactivated protein to determine unspecific binding. The ASMS assay confirmed robust specific binding of **25, 27, 39, 65** and **72** to the TLX LBD validating direct TLX modulation. As additional orthogonal evidence for binding of the scaffold, we studied the interaction of the most potent representative **39** with the TLX LBD by isothermal titration calorimetry (ITC; Figure 2c,d) which revealed a direct 1:1 binding with a K_d_ value of 1.1±0.1 µM aligning well with the compound’s in vitro potency.

**Figure 3:**
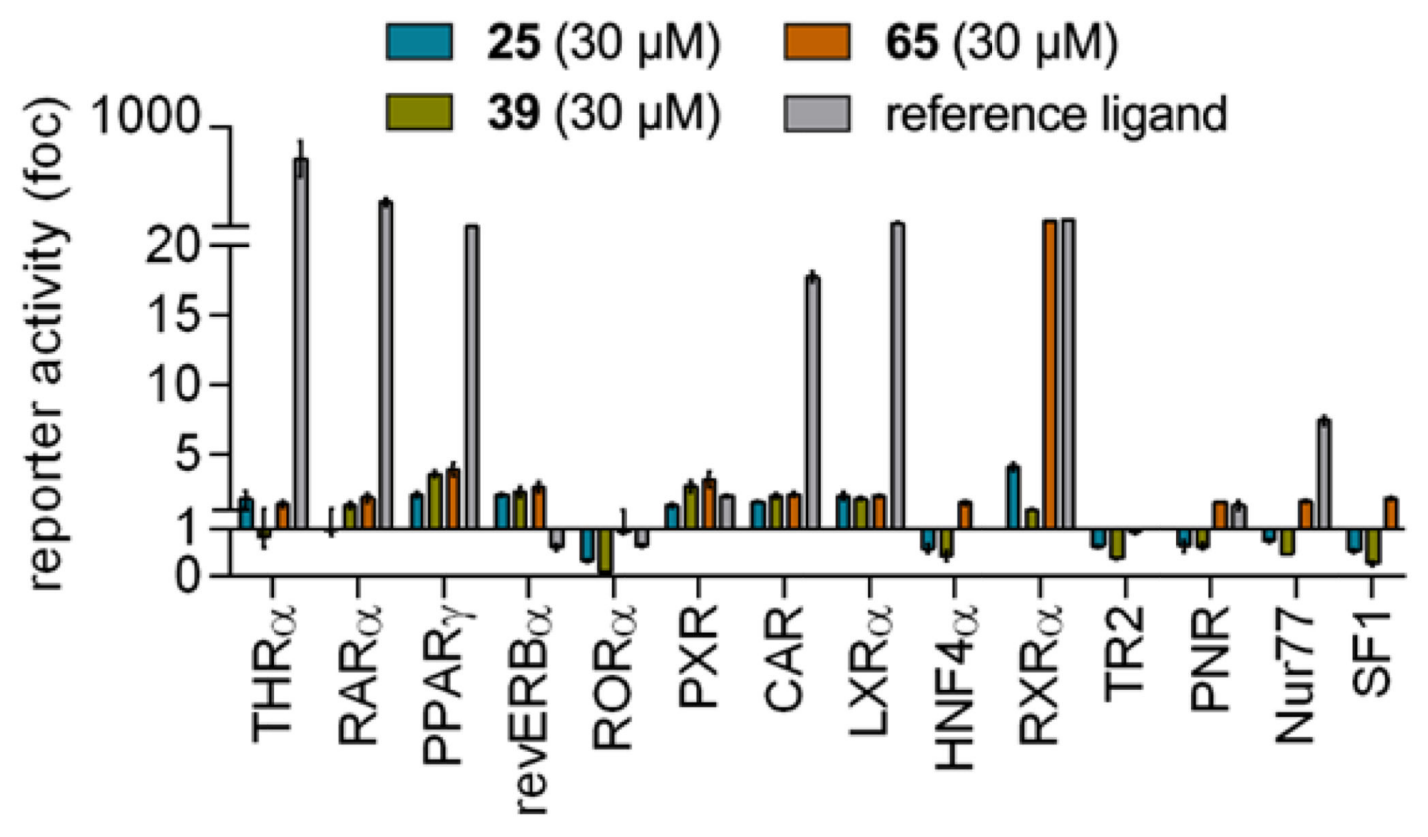
Selectivity profiling of the most active fragment TLX modulators **25, 39** and **65** in a representative panel of nuclear receptors in uniform Gal4-hybrid reporter gene assays. Reference ligands (where available) are reported in the Experimental section. Data are the mean±S.E.M.; n=3.

To further investigate the suitability of the most active fragment TLX modulators **25, 39** and **65** as leads, we determined their selectivity in a representative panel of nuclear receptors covering the NR1, NR2, NR4 and NR5 families (Figure 3). **25** and **39** displayed no pronounced off-target activity at the 30 µM test concentration with moderate RORα inhibition and weak activation of RXRα, PPARγ and PXR, while **65** mediated strong (~75-fold) RXRα activation. These off-targets will have to be considered in further optimization but given the typically higher promiscuity of fragments^40^, **25, 39** and **65** nevertheless displayed generally favorable selectivity profiles to serve as leads for structural extension to more potent TLX modulators. Additionally, the pronounced RXR agonism of **65** despite the absence of an acidic motif may be worth further investigation for the design of selective RXR ligands and dual TLX/RXR modulators.

**Figure 4:**
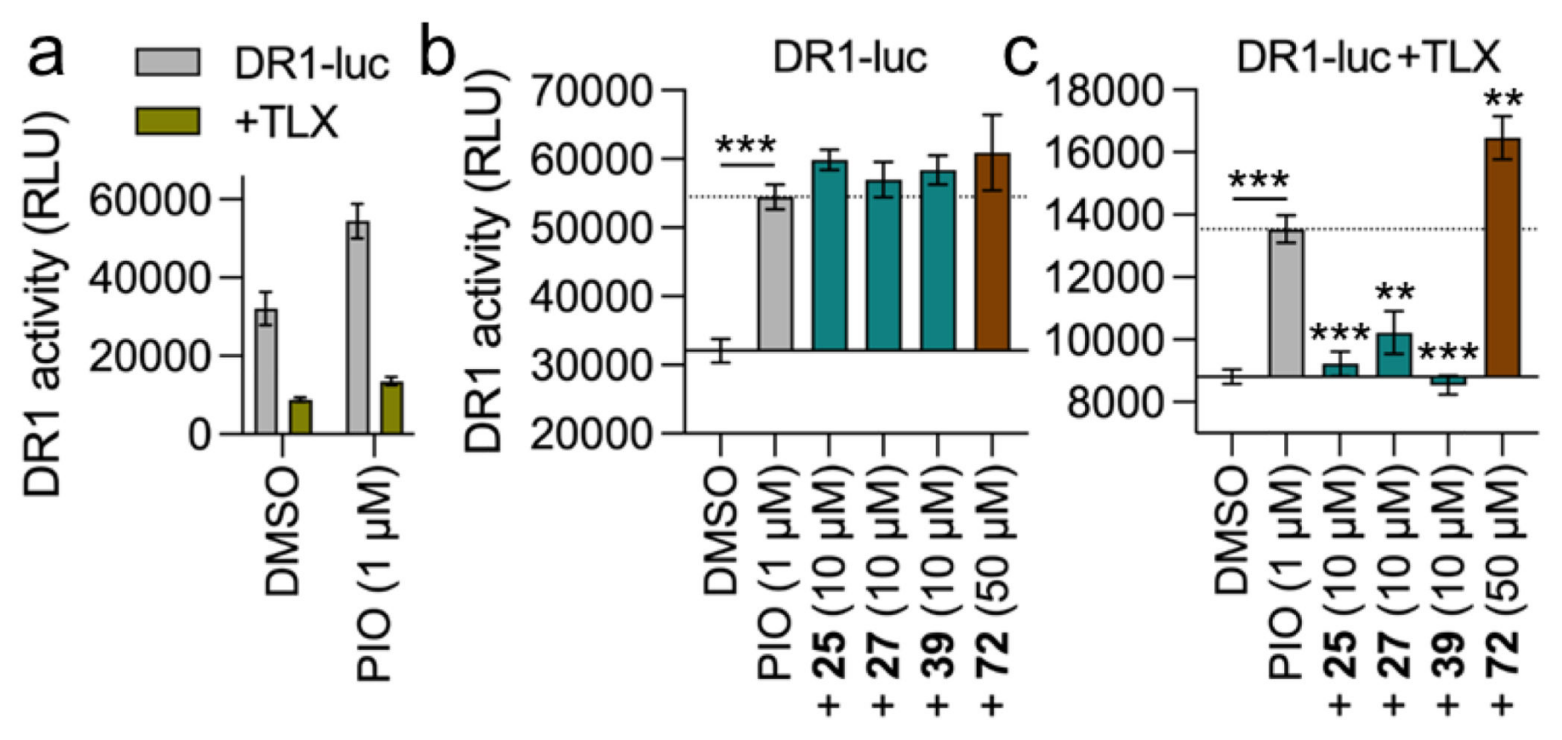
Effects of the TLX agonists **25, 27, 39** and the inverse agonist **72** on DR1 activity in HEK293T cells in absence and presence of TLX. (a) Exogenous TLX expression markedly diminished baseline and pioglitazone-induced (PIO) DR1 activity in HEK293T cells. (b) The TLX modulators **25, 27, 39** and **72** had no relevant effect on pioglitazone-induced DR1 activity in absence of exogenous TLX expression. (c) With exogenous TLX expression, the TLX agonists **25, 27** and **39** markedly enhanced repression of pioglitazone-induced DR1 activity while the inverse agonist **72** mediated de-repression. Data are the mean±SD normalized DR1 reporter activity [RLU]; n=6; ** p<0.01, *** p<0.001 (ANOVA with Bonferroni’s multiple comparisons test). Fragment **65** was excluded from these experiments for its RXR agonist activity that would cause DR1 activation.

As phenotypic effects of pharmacological TLX modulation are elusive and validated cellular models to determine TLX modulator effects are lacking, we employed a reporter system to test engagement of native human TLX in cellular context by the most active fragment TLX ligands. We used a reporter for the human nuclear receptor response element DR1 which binds several nuclear receptor dimers including RXR:PPAR and RXR:RXR^41^, and can be activated, for example, by the PPARγ agonist pioglitazone (Fig. 4a). Co-transfection of the DR1 reporter and an expression construct for full-length human TLX in HEK293T cells resulted in a markedly reduced reporter signal compared to cells transfected only with the DR1 reporter (Fig. 4a) indicating that the repressor TLX could inhibit DR1 activity. In absence of TLX, the ligands **25, 27, 39** and **72** had no effect on pioglitazone-induced DR1 activity (Figure 2b). However, when TLX was exogenously expressed, the TLX agonists **25, 27** and **39** robustly suppressed DR1 activation and the inverse TLX agonist **72** enhanced pioglitazone-induced DR1 activity. These results indicated that the TLX agonists enhanced and the inverse agonist blocked the receptor’s repressor activity supporting functional effects of the fragments in cellular context.

## Conclusion

The orphan nuclear receptor TLX is a promising target with a prominent role in neuronal health and regeneration. Its peculiar repressor function and the lack of high-quality structural data have impeded ligand discovery, however, and TLX is still highly understudied. Chemical tools with different, orthogonal TLX modulation effects are urgently needed to explore and validate therapeutic potential of the nuclear receptor. Using a moderately active screening hit as lead, we identified fragment-like TLX modulators with agonistic or inverse agonistic activity and orthogonally validated binding. Our results establish a preliminary SAR model for the bidirectional TLX modulation profile of the scaffold and form a strong basis for further optimization. Albeit their binding site in the TLX LBD and molecular activation/repression mechanisms remain to be elucidated, the new TLX modulators are an important contribution to the collection of ligands for this understudied orphan receptor. Further optimization of the fragments by structural extension is warranted.

### Chemistry

**Scheme 1:**
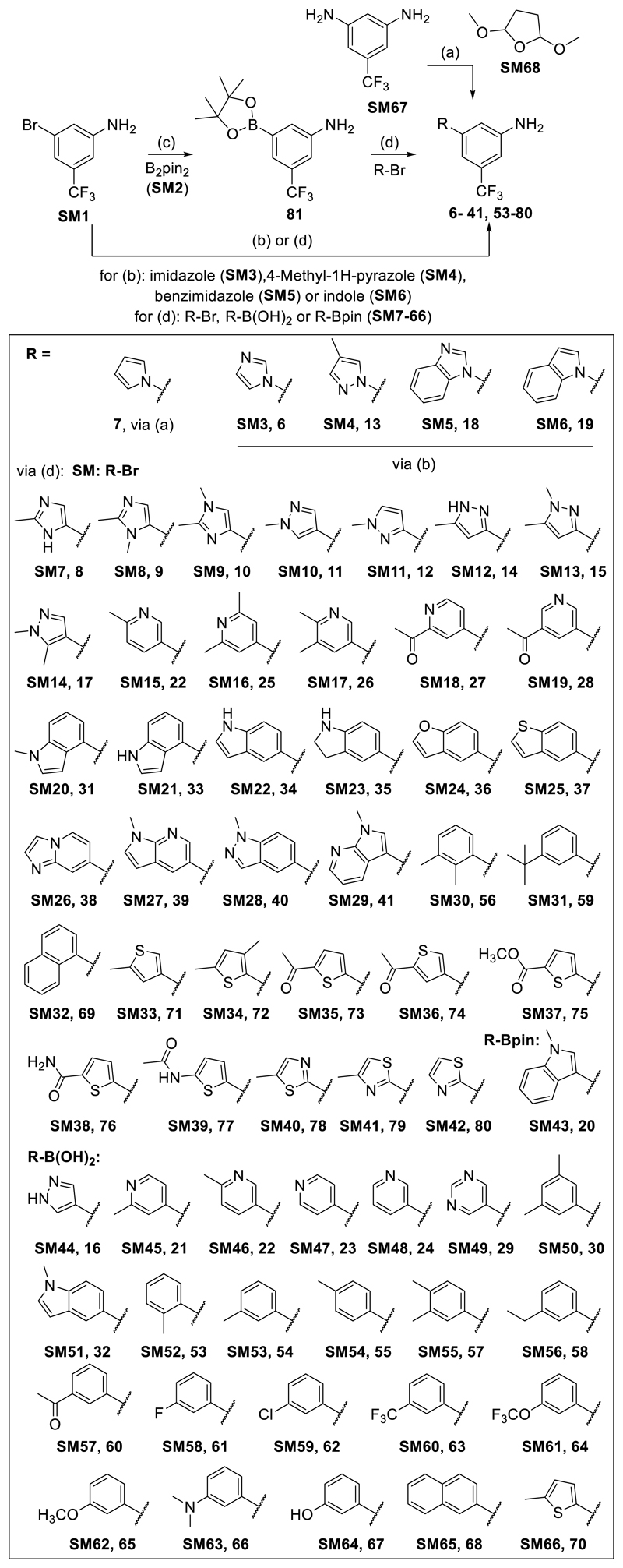
Synthesis of 6-41 and 53-80.^a^ ^a^ Reagents & Conditions: (a) FeCl_3_ (1 mol%), cat. HCl, H_2_O/DMF = 1:1, 60°C, 1 h, 29%;^43^ (b) CuI (10 mol%), *N*,*N*’-Dimethyl-1,2-cyclohexanediamine (20 mol%), sat. aq. K_2_CO_3_, 100°C, air, 48 h, 6-40%; (c) B_2_Pin_2_ (1.1 eq.), Pd(dppf)Cl_2_ (2.5 mol%), KOAc (3 eq.), 1,4-dioxane, N_2_, 90°C, overnight, 93%;^42^ (d) XPhosPdG3 (10 mol%), K_3_PO_4_, dioxane/H_2_O, N_2_, 100 °C, overnight, 14-98%.

Suzuki-Miyaura type coupling reactions under XPhos Pd G3 catalysis were performed to prepare compounds **8-12, 14-17, 20-41** and **56-80** either directly from 3-bromo-5-(trifluoromethyl)aniline (**SM1**) and the respective heteroaryl boronic acid or Bpin ester (**SM43-66**) or via the Bpin ester intermediate **81**, prepared from **SM1** by borylation^42^, which was reacted with the respective heteroaryl bromides (**SM7-SM42**, Scheme 1). Compounds **6, 13, 18** and **19** were generated from **SM1** via Ullman-type coupling with imidazole (**SM3**), 4-methyl-1*H*-pyrazole (**SM4**), benzimidazole (**SM5**) or indole (**SM6**), respectively. Synthesis of compound **5** was achieved as described in the literature^43^, from 5-(trifluoromethyl)benzene-1,3-diamine (**SM67**) and 2,5-dimethoxytetrahydrofuran (**SM68**). Starting materials described by SM numbers were commercially available.

**Scheme 2:**
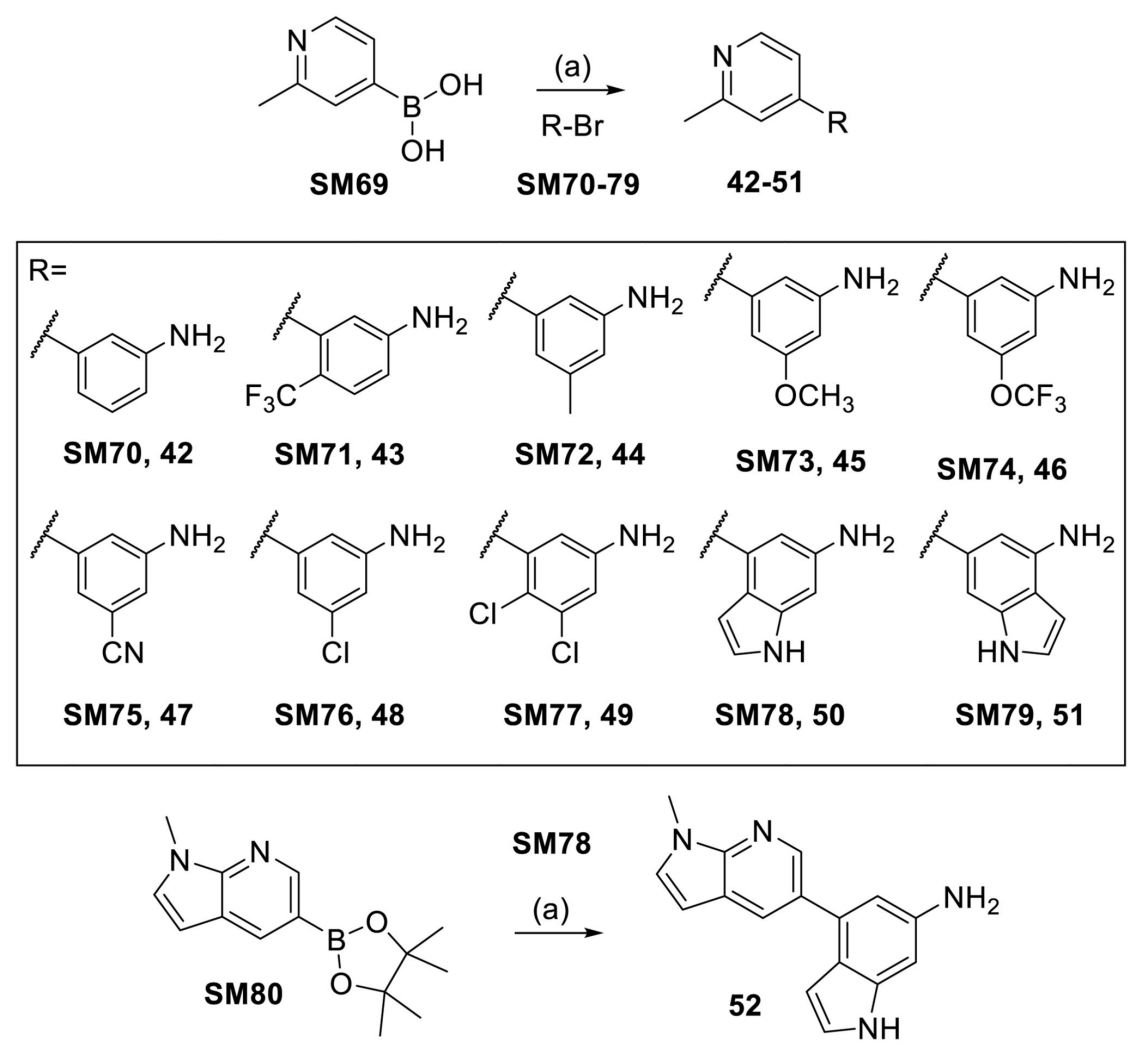
Synthesis of 42-52.^a^ ^a^ Reagents & Conditions: (a) XPhosPdG3 (10 mol%), K_3_PO_4_, dioxane/H_2_O, N_2_, 100 °C, overnight, 14-70%.

For the variation of the core aniline substitution pattern, 2-methylpyridine-4-boronic acid (**SM69**) was coupled with the aryl bromides **SM70-79** in Suzuki-Miyaura type reactions to yield compounds **42-51** (Scheme 2). Compound **52** was prepared by the same strategy from **SM78** and 1-methyl-5-(4,4,5,5-tetramethyl-1,3,2-dioxaborolan-2-yl)-1*H*-pyrrolo[2,3-b]pyridine (**SM80**, Scheme 2).

## Experimental Procedures

### Chemistry

#### General

All chemicals were of reagent grade, purchased from commercial sources (e.g., Sigma-Aldrich, TCI, BLDpharm) and used without further purification unless otherwise specified. All reactions were conducted under nitrogen (N_2_) or argon atmosphere and in absolute solvents purchased from Sigma-Aldrich. Other solvents, especially for work-up procedures, were of reagent grade or purified by distillation (iso-hexane, cyclohexane, ethyl acetate, EtOH). Reactions were monitored by thin layer chromatography (TLC) on TLC Silica gel 60 F254 coated aluminum sheets by Merck and visualized under ultraviolet light (254 nm). Purification by column chromatography was performed on a puriFlash® XS520Plus system (Advion, Ithaca, NY, USA) using high performance spherical silica columns (SIHP, 30 or 50 µm) by Interchim and a gradient of iso-hexane or cyclohexane to ethyl acetate, reversed-phase (RP) column chromatography was performed on a puriFlash® 5.250 system (Advion) using C18HP columns (SIHP, 15 µm) by Interchim and a gradient of H_2_O with 10% MeCN to 100% MeCN (HPLC gradient grade). Mass spectra were obtained on a puriFlash®-CMS system (Advion) using atmospheric pressure chemical ionization (APCI). High-resolution mass spectra (HRMS) were obtained with a Thermo Finnigan LTQ FT instrument for electron impact ionization (EI). NMR spectra were recorded on Bruker Avance III HD 400 MHz or 500 MHz spectrometers equipped with a CryoProbeTM Prodigy broadband probe (Bruker). Chemical shifts are reported in δ values (ppm) relative to residual protium signals in the NMR solvent (^1^H-NMR: Acetone-*d*_*6*_: δ = 2.05 ppm; DMSO-*d*_*6*_: δ = 2.50 ppm; MeOD-*d*_*4*_: δ = 3.31 ppm; CD_2_Cl_2_: δ = 5.32 ppm, ^13^C-NMR: Acetone-*d*_*6*_: δ = 206.26, 29.84 ppm; DMSO-*d*_*6*_: δ = 39.52 ppm; MeOD-*d*_*4*_:δ = 49.0 ppm; CD_2_Cl_2_: δ = 53.84 ppm) and coupling constants (*J*) in hertz (Hz). The splitting of signals is indicated by s for singlet, d for doublet, t for triplet, q for quartet, dd for doublet of a doublet and m for multiplet. The purity of the compounds was determined by ^1^H NMR (qHNMR) according to the method described by Pauli et al.^44^ with internal calibration. To ensure accurate determination of peak area ratio, the qHNMR measurements were conducted under conditions allowing for complete relaxation. Maleic acid (LOT#BCCH2407, purity 99.85%) was used as internal standard in DMSO-*d*_*6*_. All compounds for biological testing had a purity >95% according to quantitative ^1^H NMR (qHNMR).

#### General procedure A (Suzuki-coupling)

A 50-100 mL Schlenk flask was charged with a stir bar, the boronic acid/pinacol ester (1.0 eq.) and potassium phosphate (3.0 eq.). The flask was evacuated and backfilled with nitrogen three times. The previously de-gassed (3 freeze thaw-cycles) solvent mixture (dioxane/water = 85:15), together with the Pd catalyst (10 mol%) and the heteroaryl bromide (1.5-2.0 eq.) was added and the reaction mixture was stirred at 100°C under reflux and nitrogen atmosphere. After reaction control by TLX indicated completion of the reaction, the solution was cooled down to room temperature and the solvents were evaporated. The residue was taken up in EtOAc and washed with water and brine. The organic layer was dried over Na_2_SO_4_, concentrated and purified by flash column chromatography give the respective compound.

#### General procedure B (Ullmann coupling)

A 20 mL reaction tube was charged with the heterocycle (1.0 eq.), the aryl bromide (1.5-2.0 eq.), CuI (10 mol%) and N,N-dimethyl-1,2-diamino cyclohexane (20 mol%). 5 mL of sat. K_2_CO_3_ in water were added and the reaction mixture was stirred for 24-48h at 100°C open to air. After cooling down to room temperature, the mixture was filtered over celite and eluted with EtOAc. The phases were separated and the aqueous layer was extracted two more times with EtOAc. The combined organic layers were washed with brine and dried over Na_2_SO_4_ and the crude product was purified by flash column chromatography to yield the desired compounds.

#### *3-(1H-Imidazol-1-yl)-5-(trifluoromethyl)aniline* (**6**)

Preparation according to general procedure B from imidazole (**SM3**, 217 mg, 3.15 mmol, 1.0 eq.) and 3-bromo-5-(trifluoromethyl)aniline (**SM1**, 566 µL, 4.00 mmol, 1.3 eq.). Further purification by RP column chromatography yielded a colorless solid (43 mg, 6%). ^1^H NMR (400 MHz, MeOD-*d*_4_) δ 8.11 (s, 1H), 7.54 (s, 1H), 7.14 (s, 1H), 7.01 – 6.96 (m, 2H), 6.95 – 6.89 (m, 1H). ^13^C NMR (101 MHz, MeOD-*d*_4_) δ 152.21, 139.92, 136.95, 134.02 (q, *J* = 32.2 Hz), 130.26, 125.29 (q, *J* = 271.7 Hz), 119.71, 110.69 (q, *J* = 4.0 Hz), 110.42, 106.30 (q, *J* = 3.8 Hz). HRMS (GC/EI+): *m/z* calculated 227.0665 for C_10_H_8_F_3_N_3_, found 227.0664 ([M]^•+^). qHNMR (400 MHz, DMSO-*d*_6_) purity = 95.3%.

#### *3-(1H-Pyrrol-1-yl)-5-(trifluoromethyl)aniline* (**7**)

Preparation according a the literature procedure^43^ from 2,5-dimethoxytetrahydrofuran (**SM67**, 333 µL, 2.54 mmol, 1.0 eq.) and 5-(trifluoromethyl)benzene-1,3-diamine (**SM66**, 447 mg (2.54 mmol, 1.0 eq.) in water/DMF = 1:1. Purification by flash column chromatography and by RP column chromatography yielded an orange crystalline solid (166 mg, 29%). ^1^H NMR (400 MHz, DMSO) δ 7.29 (t, *J* = 2.2 Hz, 2H), 6.97 – 6.89 (m, 2H), 6.77 – 6.71 (m, 1H), 6.25 (t, *J* = 2.2 Hz, 2H), 5.79 (s, 2H).^13^C NMR (101 MHz, DMSO-*d*_6_) δ 150.73, 141.37, 131.02 (q, *J* = 31.4 Hz), 124.15 (q, *J* = 272.2 Hz), 119.00, 110.60, 107.38, 106.78 (q, *J* = 4.0 Hz), 102.76 (q, *J* = 3.9 Hz). HRMS (GC/EI+): *m/z* calculated 226.0712 for C_10_H_8_F_3_N_3_, found 226.0712 ([M]^•+^). qHNMR (400 MHz, DMSO-*d*_6_) purity = 99.9%.

#### *3-(4*,*4*,*5*,*5-Tetramethyl-1*,*3*,*2-dioxaborolan-2-yl)-5-(trifluoromethyl)aniline* (**81**)

This intermediate was prepared as described in previous literature^42^ with dry dioxane instead of DMSO as solvent. Purification by flash column chromatography and lyophilization yielded the compound as a yellowish crystalline solid (93%). ^1^H NMR (400 MHz, DMSO-*d*_*6*_) *δ* = 7.19 – 7.14 (m, 1H), 7.02 – 6.97 (m, 1H), 6.96 – 6.90 (m, 1H), 5.59 (s, 2H), 1.28 (s, 12H). ^13^C NMR (101 MHz, DMSO-*d*_*6*_) *δ =* 149.04, 129.40 (d, J = 30.5 Hz), 125.88, 123.31, 123.17, 116.80, 111.89, 83.87, 24.66. MS (APCI+): *m/z* 287.6 ([M+H]^+^).

#### *3-(2-Methyl-1H-imidazol-5-yl)-5-(trifluoromethyl)aniline* (**8**)

Preparation according to general procedure A from **81** (144 mg, 0.50 mmol, 1.0 eq.) and 5-bromo-2-methyl-1*H*-imidazole (**SM7**, 121 mg, 0.75 mmol, 1.5 eq.). Further purification by RP column chromatography yielded a yellowish oil (35 mg, 29 %). ^1^H NMR (400 MHz, Acetone-*d*_*6*_) δ 7.44 – 7.27 (m, 3H), 6.80 – 6.75 (m, 1H), 5.00 (s, 2H), 2.35 (s, 3H). ^13^C NMR (126 MHz, Acetone) δ 150.13, 145.44, 140.74, 138.16, 131.77 (q, *J* = 31.1 Hz), 125.87 (q, *J* = 270.8 Hz), 114.25, 112.99, 110.28, 108.73, 14.18. HRMS (GC/EI+): *m/z* calculated 241.0821 for C_11_H_10_F_3_N_3_, found 241.0822 ([M]^•+^). qHNMR (400 MHz, DMSO-*d*_6_) purity = 97.4%.

#### *3-(1*,*2-Dimethyl-1H-imidazol-5-yl)-5-(trifluoromethyl)aniline* (**9**)

Preparation according to general procedure A from **81** (144 mg, 0.50 mmol, 1.0 eq.) and 5-bromo-1,2-dimethyl-1*H*-imidazole (**SM8**, 135 mg, 0.75 mmol, 1.5 eq.). Further purification by RP column chromatography yielded a yellowish oil (35 mg, 27 %). ^1^H NMR (400 MHz, DMSO-*d*_*6*_) δ 6.86 (s, 1H), 6.84 – 6.81 (m, 2H), 6.79 – 6.74 (m, 1H), 5.70 (s, 2H), 3.51 (s, 3H), 2.33 (s, 3H). ^13^C NMR (101 MHz, MeOD-*d*_*4*_) δ 150.77, 147.76, 134.48, 133.11, 133.46 – 132.40 (m), 125.78, 125.68 (q, *J* = 273.0 Hz), 118.62, 114.23 (q, *J* = 4.1 Hz), 111.37 – 111.09 (m), 31.82, 13.09. HRMS (GC/EI+): *m/z* calculated 255.0978 for C_12_H_12_F_3_N_3_, found 255.0978 ([M]^•+^). qHNMR (400 MHz, DMSO-*d*_6_) purity = 97.1%.

#### *3-(1*,*2-Dimethyl-1H-imidazol-4-yl)-5-(trifluoromethyl)aniline* (**10**)

Preparation according to general procedure A from **81** (149 mg, 0.52 mmol, 1.0 eq.) and 4-bromo-1,2-dimethyl-1*H*-imidazole (**SM9**, 102 mg, 0.56 mmol, 1.1 eq.). Further purification by RP column chromatography yielded a colorless solid (39 mg, 29%). ^1^H NMR (400 MHz, DMSO-*d*_*6*_) δ 7.47 (s, 1H), 7.20 – 7.15 (m, 1H), 7.11 – 7.06 (m, 1H), 6.66 – 6.60 (m, 1H), 5.49 (s, 2H), 3.56 (s, 3H), 2.30 (s, 3H). ^13^C NMR (101 MHz, DMSO-*d*_*6*_) δ 149.49, 144.90, 137.26, 136.32, 129.91 (q, *J* = 30.5 Hz), 124.69 (d, *J* = 272.2 Hz), 117.62, 112.43, 107.82 – 107.56 (m), 107.25 – 106.94 (m), 32.53, 12.50. HRMS (GC/EI+): *m/z* calculated 255.0978 for C_12_H_12_F_3_N_3_, found 255.0976 ([M]^•+^). qHNMR (400 MHz, DMSO-*d*_6_) purity = 95.9%.

#### *3-(1-Methyl-1H-pyrazol-4-yl)-5-(trifluoromethyl)aniline* (**11**)

Preparation according to general procedure A from **81** (140 mg, 0.49 mmol, 1.0 eq.) and 4-bromo-1-methyl-1*H*-pyrazole (**SM10**, 76 µL, 0.73 mmol, 1.5 eq.). Further purification by RP column chromatography yielded a pale-yellow solid (53 mg, 46%). ^1^H NMR (400 MHz, DMSO-*d*_6_) *δ =* 8.11 (s, 1H), 7.78 (s, 1H), 6.95 (s, 2H), 6.68 (s, 1H), 5.54 (s, 2H), 3.85 (s, 3H). ^13^C NMR (126 MHz, DMSO-*d*_6_) *δ =* 149.77, 136.00, 134.18, 130.38 (q, *J* = 30.6 Hz), 128.13, 127.92 – 121.29 (m), 121.28, 113.18, 108.56 (q, *J* = 4.1 Hz), 107.43 (q, *J* = 4.0 Hz), 38.66. HRMS (GC/EI+): *m/z* calculated 241.0821 for C_11_H_10_F_3_N_3_, found 241.0821 ([M]^•+^). qHNMR (400 MHz, DMSO-*d*_6_) purity = 96.0%.

#### *3-(1-Methyl-1H-pyrazol-3-yl)-5-(trifluoromethyl)aniline* (**12**)

Preparation according to general procedure A from **81** (159 mg, 0.55 mmol, 1.0 eq.) and 3-bromo-1-methyl-1*H*-pyrazole (**SM11**, 85 µL, 0.83 mmol, 1.5 eq.). Further purification by RP column chromatography yielded a colorless solid (90 mg, 68%). ^1^H NMR (400 MHz, DMSO-*d*_6_) δ 7.72 (d, *J* = 2.3 Hz, 1H), 7.30 – 7.23 (m, 1H), 7.18 – 7.14 (m, 1H), 6.79 – 6.73 (m, 1H), 6.63 (d, *J* = 2.3 Hz, 1H), 5.60 (s, 2H), 3.87 (s, 3H). ^13^C NMR (126 MHz, DMSO) δ 149.70, 149.22, 134.97, 132.41, 130.13 (q, *J* = 30.7 Hz), 124.57 (q, *J* = 272.3 Hz), 113.38, 108.46 (q, *J* = 4.1 Hz), 102.59, 38.68. HRMS (GC/EI+): *m/z* calculated 241.0821 for C_11_H_10_F_3_N_3_, found 241.0820 ([M]^•+^). qHNMR (400 MHz, DMSO-*d*_6_) purity = 98.4%.

#### *3-(4-Methyl-1H-pyrazol-1-yl)-5-(trifluoromethyl)aniline* (**13**)

The compound was prepared according to general procedure B from 4-methyl-1*H*-pyrazole (**SM4**, 63 mg, 1.50 mmol, 1.0 eq.) and 3-bromo-5-(trifluoromethyl)aniline (**SM1**, 283 µL, 2.00 mmol, 1.3 eq.) to yield a colorless solid (107 mg, 30%). ^1^H NMR (400 MHz, DMSO) δ 8.25 – 8.22 (m, 1H), 7.56 – 7.52 (m, 1H), 7.25 – 7.22 (m, 1H), 7.15 – 7.12 (m, 1H), 6.75 – 6.72 (m, 1H), 5.83 (s, 2H), 2.08 (s, 3H). ^13^C NMR (101 MHz, DMSO) δ 150.61, 141.75, 141.13, 130.82 (q, J = 32.0 Hz), 126.20, 127.92 – 119.91 (m), 117.88, 107.13 – 106.86 (m), 105.90, 101.22 – 100.93 (m), 8.71. HRMS (GC/EI+): *m/z* calculated 241.0821 for C_11_H_10_F_3_N_3_, found 241.0820 ([M]^•+^). qHNMR (400 MHz, DMSO-*d*_6_) purity = 95.2%.

#### *3-(5-Methyl-1H-pyrazol-3-yl)-5-(trifluoromethyl)aniline* (**14**)

Preparation according to general procedure A from **81** (162 mg, 0.56 mmol, 1.0 eq.) and 3-bromo-5-methyl-1*H*-pyrazole (**SM12**, 143 mg, 0.87 mmol, 1.5 eq.). Further purification by RP column chromatography yielded a colorless solid (58 mg, 43%). ^1^H NMR (400 MHz, DMSO-*d*_*6*_) δ 7.21 (s, 1H), 7.16 (s, 1H), 6.80 – 6.75 (m, 1H), 6.38 (s, 1H), 2.25 (s, 3H). ^13^C NMR (101 MHz, DMSO) δ 149.20, 148.49, 141.06, 134.86, 130.13 (q, *J* = 30.9 Hz), 124.53 (q, *J* = 272.3 Hz), 113.75, 108.84 (d, *J* = 3.7 Hz), 108.73 (d, *J* = 4.1 Hz), 101.30, 10.98. HRMS (GC/EI+): *m/z* calculated 241.0821 for C_11_H_10_F_3_N_3_, found 241.0821 ([M]^•+^). qHNMR (400 MHz, DMSO-*d*_6_) purity = 96.9%.

#### *3-(1*,*5-Dimethyl-1H-pyrazol-3-yl)-5-(trifluoromethyl)aniline* (**15**)

Preparation according to general procedure A from **81** (145 mg, 0.50 mmol, 1.0 eq.) and 3-bromo-1,5-dimethyl-1*H*-pyrazole (**SM13**, 98 mg, 0.56 mmol, 1.1 eq.). Further purification by RP column chromatography yielded a colorless solid (73 mg, 57%). ^1^H NMR (400 MHz, DMSO-*d*_*6*_) δ 7.20 (s, 1H), 7.11 (s, 1H), 6.74 (s, 1H), 6.42 (s, 1H), 5.58 (s, 2H), 3.75 (s, 3H), 2.27 (s, 3H). ^13^C NMR (126 MHz, DMSO-*d*_*6*_) δ 149.66, 147.56, 139.99, 135.16, 130.08 (q, *J* = 30.7 Hz), 124.58 (q, *J* = 272.3 Hz), 113.23, 108.30 (q, *J* = 4.0 Hz), 102.25, 36.06, 10.77. HRMS (GC/EI+): *m/z* calculated 255.0978 for C_12_H_12_F_3_N_3_, found 255.0978 ([M]^•+^). qHNMR (400 MHz, DMSO-*d*_6_) purity = 97.1%.

#### *3-(1H-Pyrazol-4-yl)-5-(trifluoromethyl)aniline* (**16**)

Preparation according to general procedure A from 1*H*-pyrazol-4-ylboronic acid (**SM44**, 68 mg, 0.58 mmol, 1.05 eq.) and 3-bromo-5-(trifluoromethyl)aniline (**SM1**, 80 µL, 0.55 mmol, 1.0 eq.). Further purification by RP column chromatography yielded a colorless solid (28 mg, 22%). ^1^H NMR (400 MHz, MeOD-*d*_*4*_) δ 7.94 (s, 2H), 7.12 – 7.05 (m, 2H), 6.82 – 6.77 (m, 1H). ^13^C NMR (101 MHz, MeOD-*d*_*4*_) δ 150.51, 135.72, 133.00 (q, *J* = 31.5 Hz), 125.90 (q, *J* = 271.4 Hz), 122.98, 115.75, 111.82 (q, *J* = 3.9 Hz), 110.06 (q, *J* = 3.9 Hz). HRMS (GC/EI+): *m/z* calculated 227.0665 for C_10_H_8_F_3_N_3_, found 227.0664 ([M]^•+^). qHNMR (400 MHz, DMSO-*d*_6_) purity = 96.0%.

#### *3-(1*,*5-Dimethyl-1H-pyrazol-4-yl)-5-(trifluoromethyl)aniline* (**17**)

Preparation according to general procedure A from **81** (149 mg, 0.52 mmol, 1.0 eq.) and 4-bromo-1,5-dimethyl-1*H*-pyrazole (**SM14**, 128 mg, 0.71 mmol, 1.4 eq.). Further purification by RP column chromatography yielded a colorless solid (90 mg, 68%). ^1^H NMR (400 MHz, DMSO) δ 7.53 (s, 1H), 6.86 – 6.80 (m, 1H), 6.77 – 6.70 (m, 2H), 5.59 (s, 2H), 3.77 (s, 3H), 2.35 (s, 3H). ^13^C NMR (101 MHz, DMSO-*d*_*6*_) δ 149.75, 136.24, 135.33, 135.08, 130.17 (q, *J* = 30.7 Hz), 124.55 (q, *J* = 272.3 Hz), 118.97, 115.46, 110.44 – 110.19 (m), 107.32 – 107.05 (m), 36.33, 10.14. MS (APCI+): *m/z* 255.5 ([M]^•+^). qHNMR (400 MHz, DMSO-*d*_6_) purity = 96.5%.

#### *3-(1H-Benzo[d]imidazol-1-yl)-5-(trifluoromethyl)aniline* (**18**)

Preparation according to general procedure B from benzimidazole (**SM5**, 241 mg, 2.02 mmol, 1.0 eq.) and 3-bromo-5-(trifluoromethyl)aniline (**SM1**, 354 µL, 2.50 mmol, 1.2 eq.). Tetrabutylammonium bromide (32 mg, 0.10 mmol, 5 mol%) was added for better solubility. Further purification by RP column chromatography yielded a colorless solid (166 mg, 30%). ^1^H NMR (400 MHz, DMSO-*d*_6_) δ 8.56 (s, 1H), 7.80 – 7.74 (m, 1H), 7.64 – 7.58 (m, 1H), 7.40 – 7.28 (m, 2H), 7.11 – 7.07 (m, 1H), 7.06 – 7.01 (m, 1H), 6.99 – 6.93 (m, 1H), 6.03 (s, 2H). ^13^C NMR (126 MHz, DMSO-*d*_6_) δ 151.13, 143.83, 143.17, 137.47, 132.83, 131.36 (q, *J* = 31.6 Hz), 123.97 (q, *J* = 272.4 Hz), 123.55, 122.58, 120.04, 111.24, 110.74, 108.73 (d, *J* = 3.9 Hz), 106.28 (d, *J* = 4.0 Hz). HRMS (GC/EI+): *m/z* calculated 277.0821 for C_14_H_10_F_3_N_3_, found 277.0820 ([M]^•+^). qHNMR (400 MHz, DMSO-*d*_6_) purity = 98.4%.

#### *3-(1H-Indol-1-yl)-5-(trifluoromethyl)aniline* (**19**)

Preparation according to general procedure B from indole (**SM6**, 245 mg, 2.01 mmol, 1 eq.) and 3-bromo-5-(trifluoromethyl)aniline (**SM1**, 443 µL, 3.00 mmol, 1.5 eq.). Tetrabutylammonium bromide (30 mg, 0.10 mmol, 5 mol%) was added for better solubility. Further purification by RP column chromatography yielded a yellow oil (232 mg, 40^1^H NMR (400 MHz, DMSO) δ 7.69 – 7.61 (m, 2H), 7.56 (dt, *J* = 8.3, 0.9 Hz, 1H), 7.26 – 7.19 (m, 1H), 7.18 – 7.09 (m, 1H), 7.06 – 7.00 (m, 1H), 6.91 – 6.89 (m, 1H), 6.88 – 6.85 (m, 1H), 6.69 (dd, *J* = 3.3, 0.8 Hz, 1H), 5.94 (s, 2H).^13^C NMR (126 MHz, DMSO-*d*_6_) δ 150.94, 140.55, 134.89, 131.10 (q, *J* = 31.3 Hz), 129.18, 128.29, 124.10 (q, *J* = 272.4 Hz), 122.42, 121.04, 120.42, 111.58, 110.43, 107.59 (q, *J* = 3.9 Hz), 106.45 (q, *J* = 4.0 Hz), 103.78. HRMS (GC/EI+): *m/z* calculated 276.0869 for C_15_H_11_F_3_N_2_, found 276.0866 ([M]^•+^). qHNMR (400 MHz, DMSO-*d*_6_) purity = 96.2%.

#### *3-(1-Methyl-1H-indol-3-yl)-5-(trifluoromethyl)aniline* (**20**)

Preparation according to general procedure A from 1-methyl-3-(4,4,5,5-tetramethyl-1,3,2-dioxaborolan-2-yl)-1*H*-indole (**SM43**, 137 mg, 0.51 mmol, 1.0 eq.) and 3-bromo-5-(trifluoromethyl)aniline (**SM1**, 81 µL, 0.56 mmol, 1.1 eq.). Further purification by RP column chromatography yielded a yellow oily solid (96 mg, 64%). ^1^H NMR (400 MHz, DMSO-*d*_6_) δ 7.87 (d, *J* = 8.0 Hz, 1H), 7.73 (s, 1H), 7.50 (d, *J* = 8.1 Hz, 1H), 7.27 – 7.12 (m, 3H), 7.02 (s, 1H), 6.72 (s, 1H), 5.62 (s, 2H), 3.83 (s, 3H). ^13^C NMR (101 MHz, DMSO-*d*_6_) δ 149.88, 137.25, 136.99, 130.26 (q, *J* = 30.7 Hz), 128.15, 125.12, 124.69 (q, *J* = 272.1 Hz), 121.63, 119.89, 119.14, 114.76, 114.04, 110.35, 109.72 (q, *J* = 3.4 Hz), 106.63 (q, *J* = 3.8 Hz), 32.57. HRMS (GC/EI+): *m/z* calculated 290.1025 for C_16_H_13_F_3_N_2_, found 290.1025 ([M]^•+^). qHNMR (400 MHz, DMSO-*d*_6_) purity = 96.0%.

#### *3-(2-Methylpyridin-4-yl)-5-(trifluoromethyl)aniline* (**21**)

Preparation according to general procedure A from 3-bromo-5-(trifluoromethyl)aniline (**SM1**, 106 µL, 0.75 mmol, 1.5 eq.) and 2-methylpyridine-4-boronic acid (**SM45**, 68.5 mg, 0.5 mmol, 1.0 eq.). Further purification by RP column chromatography yielded a colorless solid (43 mg, 34%). ^1^H NMR (400 MHz, DMSO-*d*_*6*_) δ 8.49 (d, *J* = 5.3 Hz, 1H), 7.51 (s, 1H), 7.41 (d, *J* = 5.3 Hz, 1H), 7.17 – 7.13 (m, 1H), 7.12 – 7.08 (m, 1H), 6.95 – 6.91 (m, 1H), 5.77 (s, 2H), 2.53 (s, 3H). ^13^C NMR (126 MHz, Acetone-*d*_*6*_) δ 160.04, 150.96, 150.67, 148.38, 141.25, 132.65 (q, *J* = 31.5 Hz), 125.54 (q, *J* = 271.5 Hz), 121.59, 119.44, 116.66, 112.05 (q, *J* = 4.1 Hz), 111.49 (q, *J* = 3.8 Hz), 24.63. HRMS (GC/EI+): *m/z* calculated 252.0869 for C_13_H_11_F_3_N_2_, found 252.0869 ([M]^•+^). qHNMR (400 MHz, DMSO-*d*_6_) purity = 95.5%.

#### *3-(6-Methylpyridin-3-yl)-5-(trifluoromethyl)aniline* (**22**)

Preparation according to general procedure A from **81** (155 mg, 0.54 mmol, 1.0 eq.) and 5-bromo-2-methylpyridine (**SM15**, 105 mg, 0.61 mmol, 1.1 eq.). Further purification by RP column chromatography yielded a colorless solid (93 mg, 69%). ^1^H NMR (400 MHz, DMSO-*d*_6_) δ 8.67 (d, *J* = 2.4 Hz, 1H), 7.90 (dd, *J* = 8.1, 2.5 Hz, 1H), 7.33 (d, *J* = 8.1 Hz, 1H), 7.10 – 7.05 (m, 1H), 7.05 – 7.00 (m, 1H), 6.90 – 6.85 (m, 1H), 5.72 (s, 2H), 2.50 (s, 3H). ^13^C NMR (126 MHz, DMSO-*d*_6_) δ 157.38, 150.08, 146.73, 138.98, 134.41, 132.21, 130.64 (q, *J* = 30.9 Hz), 124.42 (q, *J* = 272.2 Hz), 123.17, 115.03, 109.81 (q, *J* = 4.1 Hz), 108.96 (q, *J* = 3.9 Hz), 23.68. HRMS (GC/EI+): *m/z* calculated 252.0869 for C_13_H_11_F_3_N_2_, found 252.0870 ([M]^•+^). qHNMR (400 MHz, DMSO-*d*_6_) purity = 98.0%.

#### *3-(Pyridin-4-yl)-5-(trifluoromethyl)aniline* (**23**)

Preparation according to general procedure A from 4-pyridylboronic acid (**SM47**, 63 mg, 0.50 mmol, 1.0 eq.) and 3-bromo-5-(trifluoromethyl)aniline (**SM1**, 74 µL, 0.53 mmol, 1.1 eq.). Further purification by RP column chromatography yielded a colorless solid (50 mg, 42%). ^1^H NMR (400 MHz, DMSO-*d*_6_) δ 8.67 – 8.61 (m, 2H), 7.66 – 7.60 (m, 2H), 7.20 – 7.15 (m, 1H), 7.14 – 7.10 (m, 1H), 6.98 – 6.92 (m, 1H), 5.82 (s, 2H^13^C NMR (101 MHz, DMSO) δ 150.24, 150.23, 146.57, 139.10, 130.77 (q, *J* = 30.9 Hz), 124.33 (q, *J* = 272.2 Hz), 121.28, 115.15, 110.13 (q, *J* = 4.1 Hz), 109.88 (q, *J* = 4.1 Hz). HRMS (GC/EI+): *m/z* calculated 238.0712 for C_12_H_9_F_3_N_2_, found 238.0713 ([M]^•+^). qHNMR (400 MHz, DMSO-*d*_6_) purity = 97.8%.

#### *3-(Pyridin-3-yl)-5-(trifluoromethyl)aniline* (**24**)

Preparation according to general procedure A from 3-pyridylboronic acid (**SM48**,111 mg, 0.90 mmol, 1.2 eq.) and 3-bromo-5-(trifluoromethyl)aniline (**SM1**,106 µL, 0.75 mmol, 1.0 eq.). Further purification by RP column chromatography yielded a colorless solid (174 mg, 98%). ^1^H NMR (400 MHz, DMSO-*d*_6_) δ 8.82 (d, *J* = 2.4 Hz, 1H), 8.59 (dd, *J* = 4.8, 1.6 Hz, 1H), 8.05 – 7.98 (m, 1H), 7.52 – 7.44 (m, 1H), 7.12 – 7.08 (m, 1H), 7.08 – 7.04 (m, 1H), 6.94 – 6.88 (m, 1H), 5.75 (s, 2H). ^13^C NMR (101 MHz, DMSO-*d*_6_) δ 150.13, 148.92, 147.55, 138.94, 135.11, 134.22, 131.28 – 130.29 (m), 123.89, 115.32, 110.34 – 109.83 (m), 109.55 – 108.96 (m). HRMS (GC/EI+): *m/z* calculated 238.0712 for C_12_H_9_F_3_N_2_, found 238.0711 ([M]^•+^). qHNMR (400 MHz, DMSO-*d*_6_) purity = 96.7%.

#### *3-(2*,*6-Dimethylpyridin-4-yl)-5-(trifluoromethyl)aniline* (**25**)

Preparation according to general procedure A from **81** (140 mg, 0.49 mmol, 1.0 eq.) and 4-bromo-2,6-dimethylpyridine (**SM16**, 97 mg, 0.52 mmol, 1.0 eq.). Further purification by RP column chromatography yielded a colorless solid (28 mg, 22%). ^1^H NMR (400 MHz, MeOD-*d*_*4*_) δ 7.30 (s, 2H), 7.18 – 7.14 (m, 1H), 7.14 – 7.10 (m, 1H), 7.01 – 6.96 (m, 1H), 2.55 (s, 6H). ^13^C NMR (126 MHz, MeOD-*d*_*4*_) δ 159.30, 151.05, 150.71, 141.26, 133.24 (q, *J* = 31.6 Hz), 125.73 (q, *J* = 271.4 Hz), 119.83, 117.07, 112.69 (q, *J* = 4.0 Hz), 112.27 (q, *J* = 3.9 Hz), 23.78. HRMS (GC/EI+): *m/z* calculated 266.1025 for C_14_H_13_F_3_N_2_, found 266.1027 ([M]^•+^). qHNMR (400 MHz, DMSO-*d*_6_) purity = 95.3%.

#### *3-(5*,*6-Dimethylpyridin-3-yl)-5-(trifluoromethyl)aniline* (**26**)

Preparation according to general procedure A from **81** (146 mg, 0.51 mmol, 1.0 eq.) and 5-bromo-2,3-dimethylpyridine (**SM17**, 73 µL, 0.53 mmol, 1.1 eq.). Further purification by RP column chromatography yielded a colorless solid (48 mg, 35%). ^1^H NMR (400 MHz, DMSO-*d*_6_) δ 8.49 (d, *J* = 2.3 Hz, 1H), 7.76 (d, *J* = 2.3 Hz, 1H), 7.10 – 7.06 (m, 1H), 7.05 – 7.01 (m, 1H), 6.90 – 6.84 (m, 1H), 5.71 (s, 2H), 2.45 (s, 3H), 2.31 (s, 3H). ^13^C NMR (101 MHz, DMSO-*d*_6_) δ 156.28, 150.05, 143.84, 139.06, 134.94, 132.68, 131.34, 130.62 (q, *J* = 31.6 Hz), 126.15 – 122.93 (m), 115.01, 109.81 (d, *J* = 4.0 Hz), 108.90 (d, *J* = 3.9 Hz), 22.01, 18.57. HRMS (GC/EI+): *m/z* calculated 266.1025 for C_14_H_13_F_3_N_2_, found 266.1025 ([M]^•+^). qHNMR (400 MHz, DMSO-*d*_6_) purity = 98.8%.

#### *1-(4-(3-Amino-5-(trifluoromethyl)phenyl)pyridin-2-yl)ethan-1-one* (**27**)

Preparation according to general procedure A from **81** (153 mg, 0.53 mmol, 1.1 eq.) and 1-(4-bromopyridin-2-yl)ethanone (**SM18**, 100 mg, 0.49 mmol, 1.0 eq.). Further purification by RP column chromatography yielded a pale-yellow solid (53 mg, 39%). ^1^H NMR (400 MHz, DMSO-*d*_6_) δ 8.78 (dd, *J* = 5.0, 0.8 Hz, 1H), 8.12 (dd, *J* = 1.9, 0.8 Hz, 1H), 7.95 (dd, *J* = 5.1, 1.9 Hz, 1H), 7.28 – 7.23 (m, 1H), 7.21 – 7.16 (m, 1H), 7.00 – 6.95 (m, 1H), 5.84 (s, 2H), 2.68 (s, 3H). ^13^C NMR (101 MHz, MeOD-*d*_4_) δ 208.84, 163.18, 159.83, 159.53, 157.14, 147.80, 140.36 (q, *J* = 31.2 Hz), 134.30, 137.98 – 129.49 (m), 127.63, 124.63, 119.94 (q, *J* = 3.9 Hz), 119.30 (q, *J* = 4.0 Hz), 35.28. HRMS (GC/EI+): *m/z* calculated 280.0818 for C_14_H_11_F_3_N_2_O, found 280.0819 ([M]^•+^). qHNMR (400 MHz, DMSO-*d*_6_) purity = 95.5%.

#### *1-(5-(3-Amino-5-(trifluoromethyl)phenyl)pyridin-3-yl)ethan-1-one* (**28**)

Preparation according to general procedure A from **81** (144 mg, 0.50 mmol, 1.0 eq.) and 3-acetyl-5-bromopyridine (**SM19**, 71 µL, 0.53 mmol, 1.1 eq.). Further purification by RP column chromatography yielded a colorless solid (59 mg, 42%). ^1^H NMR (400 MHz, DMSO-*d*_6_) δ 9.11 (d, *J* = 2.0 Hz, 1H), 9.05 (d, *J* = 2.3 Hz, 1H), 8.42 (t, *J* = 2.2 Hz, 1H), 7.20 – 7.15 (m, 2H), 6.97 – 6.91 (m, 1H), 5.79 (s, 2H), 2.70 (s, 3H^13^C NMR (101 MHz, DMSO) δ 197.48, 151.25, 150.22, 148.63, 138.02, 135.07, 133.37, 132.02, 130.83 (q, *J* = 31.5 Hz), 124.38 (q, *J* = 272.6 Hz), 115.50, 110.25 (q, *J* = 4.1 Hz), 109.63 (q, *J* = 3.8 Hz), 27.19. HRMS (GC/EI+): *m/z* calculated 280.0818 for C_14_H_11_F_3_N_2_O, found 280.0818 ([M]^•+^). qHNMR (400 MHz, DMSO-*d*_6_) purity = 97.3%.

#### *3-(Pyrimidin-5-yl)-5-(trifluoromethyl)aniline* (**29**)

Preparation according to general procedure A from pyrimidine-5-boronic acid (**SM49**, 63 mg, 0.51 mmol, 1.0 eq.) and 3-bromo-5-(trifluoromethyl)aniline (**SM1**, 74 µL, 0.53 mmol, 1.1 eq.). Further purification by RP column chromatography yielded a colorless solid (54 mg, 45%). ^1^H NMR (400 MHz, DMSO-*d*_6_) δ 9.20 (s, 1H), 9.07 (s, 2H), 7.19 – 7.15 (m, 1H), 7.14 – 7.11 (m, 1H), 6.98 – 6.93 (m, 1H), 5.80 (s, 2H). ^13^C NMR (101 MHz, DMSO-*d*_6_) δ 157.62, 154.78, 150.23, 135.68, 132.88, 130.90 (q, *J* = 31.1 Hz), 128.47 – 120.08 (m), 115.24, 110.11 (q, *J* = 3.9 Hz), 109.86 (q, *J* = 3.9 Hz). HRMS (GC/EI+): *m/z* calculated 239.0665 for C_11_H_8_F_3_N_3_, found 239.0665 ([M]^•+^). qHNMR (400 MHz, DMSO-*d*_6_) purity = 99.0%.

#### *3’*,*5’-Dimethyl-5-(trifluoromethyl)-[1*,*1’-biphenyl]-3-amine* (**30**)

Preparation according to general procedure A from 3,5-dimethylbenzeneboronic acid (**SM50**, 79 mg, 0.53 mmol, 1.1 eq.) and 3-bromo-5-(trifluoromethyl)aniline (**SM1**, 71 µL, 0.50 mmol, 1.0 eq.). Further purification by RP column chromatography yielded a yellowish solid (102 mg, 77%). ^1^H NMR (400 MHz, DMSO-*d*_*6*_) δ 7.20 (s, 2H), 7.09 – 7.03 (m, 1H), 7.01 (s, 1H), 6.99 – 6.96 (m, 1H), 6.86 – 6.81 (m, 1H), 5.66 (s, 2H), 2.32 (s, 6H). ^13^C NMR (101 MHz, DMSO-*d*_*6*_) δ 149.81, 142.16, 139.52, 137.95, 130.31 (q, *J* = 30.8 Hz), 129.27, 124.53 (q, *J* = 272.5 Hz), 124.41, 115.32, 109.95 (q, *J* = 3.8 Hz), 108.50 (q, *J* = 4.0 Hz), 20.94, 20.92. HRMS (GC/EI+): *m/z* calculated 265.1073 for C_15_H_14_F_3_N, found 265.1076 ([M]^•+^). qHNMR (400 MHz, DMSO-*d*_6_) purity = 96.2%.

#### *3-(1-Methyl-1H-indol-4-yl)-5-(trifluoromethyl)aniline* (**31**)

Preparation according to general procedure A from **81** (195 mg, 0.68 mmol, 1.1 eq.) and 4-bromo-1-methyl-1*H*-indole (**SM20**, 132 mg, 0.62 mmol, 1.0 eq.). Further purification by RP column chromatography yielded a colorless oily solid (108 mg, 61%). ^1^H NMR (400 MHz, DMSO-*d*_6_) δ 7.46 (d, *J* = 8.2 Hz, 1H), 7.41 (d, *J* = 3.1 Hz, 1H), 7.28 – 7.20 (m, 1H), 7.15 – 7.12 (m, 1H), 7.10 (dd, *J* = 7.3, 0.9 Hz, 1H), 7.02 – 6.96 (m, 1H), 6.91 – 6.84 (m, 1H), 6.50 (dd, *J* = 3.2, 0.9 Hz, 1H), 3.83 (s, 3H). ^1^H NMR (400 MHz, DMSO) δ 7.46 (d, *J* = 8.2 Hz, 1H), 7.41 (d, *J* = 3.1 Hz, 1H), 7.28 – 7.20 (m, 1H), 7.15 – 7.12 (m, 1H), 7.10 (dd, *J* = 7.3, 0.9 Hz, 1H), 7.02 – 6.96 (m, 1H), 6.91 – 6.84 (m, 1H), 6.50 (dd, *J* = 3.2, 0.9 Hz, 1H), 5.70 (s, 2H), 3.83 (s, 3H). HRMS (GC/EI+): *m/z* calculated 290.1025 for C_16_H_13_F_3_N_2_, found 290.1026 ([M]^•+^). qHNMR (400 MHz, DMSO-*d*_6_) purity = 96.1%.

#### *3-(1-Methyl-1H-indol-5-yl)-5-(trifluoromethyl)aniline* (**32**)

Preparation according to general procedure A from 3-bromo-5-(trifluoromethyl)aniline (**SM1**, 212 µL, 1.50 mmol, 1.5 eq.) and 1-methylindole-5-boronic acid (**SM51**, 175 mg, 1.0 mmol, 1.0 eq.). Further purification by RP column chromatography yielded a colorless oil (104 mg, 36%). ^1^H NMR (400 MHz, MeOD-*d*_4_) δ 7.77 – 7.72 (m, 1H), 7.42 – 7.32 (m, 2H), 7.17 – 7.13 (m, 2H), 7.12 (d, *J* = 3.1 Hz, 1H), 6.90 – 6.84 (m, 1H), 6.46 (dd, *J* = 3.1, 0.7 Hz, 1H), 3.75 (s, 3H). ^13^C NMR (101 MHz, MeOD-*d*_4_) δ 150.17, 145.82, 138.03, 132.82, 133.23 – 132.06 (m), 130.86, 130.47, 126.06 (q, *J* = 271.4 Hz), 121.78, 119.96, 117.79, 113.64 (q, *J* = 4.0 Hz), 110.50, 110.00 (q, *J* = 3.8 Hz), 102.18, 32.86. HRMS (GC/EI+): *m/z* calculated 290.1025 for C_16_H_13_F_3_N_2_, found 290.1029 ([M]^•+^). qHNMR (400 MHz, DMSO-*d*_6_) purity = 95.0%.

#### *3-(1H-Indol-4-yl)-5-(trifluoromethyl)aniline* (**33**)

Preparation according to general procedure A from **81** (147 mg, 0.51 mmol, 1.0 eq.) and 4-bromo-1*H*-indole (**SM21**, 65 µL, 0.56 mmol, 1.1 eq.). Further purification by RP column chromatography yielded a colorless oily solid (84 mg, 60%). ^1^H NMR (400 MHz, DMSO-*d*_6_) δ 11.28 (s, 1H), 7.46 – 7.39 (m, 2H), 7.20 – 7.15 (m, 1H), 7.15 – 7.13 (m, 1H), 7.06 (dd, *J* = 7.3, 1.0 Hz, 1H), 7.02 – 6.99 (m, 1H), 6.88 – 6.83 (m, 1H), 6.55 – 6.49 (m, 1H), 5.69 (s, 2H). ^13^C NMR (101 MHz, DMSO-*d*_6_) δ 149.77, 142.61, 136.38, 132.21, 130.03 (q, *J* = 31.1 Hz), 125.96, 125.37, 128.84 – 120.06 (m), 121.30, 118.31, 116.90, 111.64 – 111.28 (m), 111.22, 108.37 – 107.85 (m), 100.03. HRMS (GC/EI+): *m/z* calculated 276.0869 for C_15_H_11_F_3_N_2_, found 276.0868 ([M]^•+^). qHNMR (400 MHz, DMSO-*d*_6_) purity = 95.4%.

#### *3-(1H-Indol-5-yl)-5-(trifluoromethyl)aniline* (**34**)

Preparation according to general procedure A from **81** (150 mg, 0.52 mmol, 1.0 eq.) and 5-bromoindole (**SM22**, 160 mg, 0.82 mmol, 1.6 eq.). Further purification by RP column chromatography yielded a yellow solid (79 mg, 55%). ^1^H NMR (400 MHz, MeOD-*d*_4_) δ 7.76 (dd, *J* = 1.8, 0.8 Hz, 1H), 7.44 (dt, *J* = 8.4, 0.9 Hz, 1H), 7.34 (dd, *J* = 8.5, 1.8 Hz, 1H), 7.26 (d, *J* = 3.1 Hz, 1H), 7.19 – 7.16 (m, 1H), 7.14 – 7.11 (m, 1H), 6.89 – 6.83 (m, 1H), 6.50 (dd, *J* = 3.1, 0.9 Hz, 1H). ^13^C NMR (101 MHz, MeOD-*d*_4_) δ 150.21, 146.05, 137.49, 132.81 (d, *J* = 3.5 Hz), 133.16 – 132.10 (m), 129.97, 126.45, 130.33 – 121.88 (m), 121.73, 119.62, 117.82, 113.93 – 113.41 (m), 112.46, 110.11 – 109.68 (m), 102.83. HRMS (GC/EI+): *m/z* calculated 276.0869 for C_15_H_11_F_3_N_2_, found 276.0869 ([M]^•+^). qHNMR (400 MHz, DMSO-*d*_6_) purity = 98.8%.

#### *3-(Indolin-5-yl)-5-(trifluoromethyl)aniline* (**35**)

Preparation according to general procedure A from **81** (144 mg, 0.50 mmol, 1.0 eq.) and 5-bromoindoline (**SM23**, 116 mg, 0.58 mmol, 1.2 eq.). Further purification by RP column chromatography yielded a colorless solid (53 mg, 38%). ^1^H NMR (400 MHz, MeOD-*d*_4_) δ 7.35 – 7.31 (m, 1H), 7.25 – 7.20 (m, 1H), 7.06 – 7.03 (m, 1H), 7.03 – 7.00 (m, 1H), 6.83 – 6.80 (m, 1H), 6.70 (d, *J* = 8.1 Hz, 1H), 3.51 (t, *J* = 8.4 Hz, 2H), 3.04 (t, *J* = 8.3 Hz, 2H). ^13^C NMR (101 MHz, MeOD-*d*_4_) δ 153.14, 150.22, 144.92, 132.67 (q, *J* = 31.3 Hz), 132.53, 131.89, 127.17, 126.03 (q, *J* = 271.3 Hz), 124.07, 116.91, 112.81 (q, *J* = 4.1 Hz), 111.10, 109.87 (q, *J* = 3.7 Hz), 48.28, 30.68. HRMS (GC/EI+): *m/z* calculated 278.1025 for C_15_H_13_F_3_N_2_, found 278.1027 ([M]^•+^). qHNMR (400 MHz, DMSO-*d*_6_) purity = 98.1%.

#### *3-(Benzofuran-5-yl)-5-(trifluoromethyl)aniline* (**36**)

Preparation according to general procedure A from **81** (159 mg, 0.55 mmol, 1.0 eq.) and 5-bromobenzufurane (**SM24**, 76 µL, 0.61 mmol, 1.1 eq.). Further purification by RP column chromatography yielded a colorless oil (87 mg, 57%). ^1^H NMR (400 MHz, DMSO-*d*_6_) δ 8.04 (d, *J* = 2.2 Hz, 1H), 7.87 (dd, *J* = 2.0, 0.6 Hz, 1H), 7.67 (d, *J* = 8.6 Hz, 1H), 7.53 (dd, *J* = 8.6, 1.9 Hz, 1H), 7.13 – 7.07 (m, 1H), 7.05 – 6.98 (m, 2H), 6.87 – 6.82 (m, 1H), 5.68 (s, 2H). ^13^C NMR (101 MHz, DMSO-*d*_6_) δ 154.11, 149.92, 146.78, 142.40, 134.92, 130.39 (q, *J* = 30.8 Hz), 127.89, 124.54 (q, *J* = 272.1 Hz), 123.40, 119.41, 115.62, 111.59, 110.25 (q, *J* = 3.7 Hz), 108.25 (q, *J* = 4.0 Hz), 107.02. HRMS (GC/EI+): *m/z* calculated 277.0709 for C_15_H_10_F_3_NO, found 277.0710 ([M]^•+^). qHNMR (400 MHz, DMSO-*d*_6_) purity = 97.2%.

#### *3-(Benzo[b]thiophen-5-yl)-5-(trifluoromethyl)aniline* (**37**)

Preparation according to general procedure A from **81** (143 mg, 0.50 mmol, 1.0 eq.) and 5-bromobenzo[b]thiophene (**SM25**, 144 mg, 0.68 mmol, 1.4 eq.). Further purification by RP column chromatography yielded a colorless oil (104 mg, 72%). ^1^H NMR (400 MHz, MeOD-*d*_4_) δ 8.05 – 8.00 (m, 1H), 7.94 (d, *J* = 8.4 Hz, 1H), 7.63 – 7.52 (m, 2H), 7.41 (d, *J* = 5.5 Hz, 1H), 7.21 – 7.17 (m, 1H), 7.17 – 7.14 (m, 1H), 6.96 – 6.90 (m, 1H). ^13^C NMR (101 MHz, MeOD-*d*_4_) δ 150.58, 144.42, 141.77, 140.63, 138.10, 132.93 (q, *J* = 31.5 Hz), 128.33, 125.94 (q, *J* = 271.6 Hz), 125.17, 124.54, 123.72, 122.80, 117.72, 113.43 (q, *J* = 4.0 Hz), 110.78 (q, *J* = 3.9 Hz). HRMS (GC/EI+): *m/z* calculated 293.0481 for C_15_H_10_F_3_NS, found 293.0485 ([M]^•+^). qHNMR (400 MHz, DMSO-*d*_6_) purity = 96.0%.

#### *3-(Imidazo[1*,*2-a]pyridin-7-yl)-5-(trifluoromethyl)aniline* (**38**)

Preparation according to general procedure A from **81** (145 mg, 0.50 mmol, 1.0 eq.) and 7-bromoimidazo[1,2-a]pyridine (**SM26**, 123 mg, 0.62 mmol, 1.2 eq.). Further purification by RP column chromatography yielded a colorless solid (61 mg, 44%). ^1^H NMR (400 MHz, DMSO-*d*_6_) δ 8.62 (d, *J* = 7.1 Hz, 1H), 7.98 (s, 1H), 7.81 – 7.76 (m, 1H), 7.62 (s, 1H), 7.23 – 7.18 (m, 2H), 7.15 (s, 1H), 6.92 – 6.87 (m, 1H), 5.73 (s, 2H). ^13^C NMR (101 MHz, DMSO-*d*_6_) δ 150.05, 144.76, 139.83, 135.40, 134.10, 130.62 (q, *J* = 30.9 Hz), 127.08, 124.43 (q, *J* = 272.4 Hz), 114.96, 113.41, 113.05, 111.18, 109.81 (q, *J* = 4.0 Hz), 109.24 (q, *J* = 3.3 Hz). HRMS (GC/EI+): *m/z* calculated 277.0821 for C_14_H_10_F_3_N_3_, found 277.0821 ([M]^•+^). qHNMR (400 MHz, DMSO-*d*_6_) purity = 95.6%.

#### *3-(1-Methyl-1H-pyrrolo[2*,*3-b]pyridin-5-yl)-5-(trifluoromethyl)aniline* (**39**)

Preparation according to general procedure A from **81** (193 mg, 0.67 mmol, 1 eq.) and 5-bromo-1-methyl-1*H*-pyrrolo[2,3-b]pyridine (**SM27**, 150 mg, 0.69 mmol, 1.0 eq.). Further purification by RP column chromatography yielded a colorless solid (123 mg, 63%). ^1^H NMR (400 MHz, DMSO-*d*_6_) δ 8.49 (d, *J* = 2.2 Hz, 1H), 8.17 (d, *J* = 2.2 Hz, 1H), 7.57 (d, *J* = 3.4 Hz, 1H), 7.14 – 7.08 (m, 1H), 7.08 – 7.03 (m, 1H), 6.88 – 6.83 (m, 1H), 6.52 (d, *J* = 3.4 Hz, 1H), 5.69 (s, 2H), 3.85 (s, 3H). ^13^C NMR (126 MHz, DMSO) δ 149.98, 147.20, 141.14, 140.75, 131.02, 130.52 (q, *J* = 30.8 Hz), 127.63, 126.51, 124.54 (q, *J* = 272.2 Hz), 119.93, 115.48, 110.21 (q, *J* = 4.0 Hz), 108.20 (q, *J* = 3.9 Hz), 99.28, 30.97. HRMS (GC/EI+): *m/z* calculated 291.0978 for C_15_H_12_F_3_N_3_, found 291.0980 ([M]^•+^). qHNMR (400 MHz, DMSO-*d*_6_) purity = 98.0%.

#### *3-(1-Methyl-1H-indazol-5-yl)-5-(trifluoromethyl)aniline* (**40**)

Preparation according to general procedure A from **81** (165 mg, 0.58 mmol, 1.0 eq.) and 5-bromo-1-methyl-1*H*-indazole (**SM28**, 124 mg, 0.58 mmol, 1.0 eq.). Further purification by RP column chromatography yielded a colorless solid (101 mg, 60%). ^1^H NMR (400 MHz, DMSO-*d*_6_) δ 8.10 (d, *J* = 1.0 Hz, 1H), 7.99 – 7.96 (m, 1H), 7.72 (dt, *J* = 8.8, 0.9 Hz, 1H), 7.65 (dd, *J* = 8.8, 1.7 Hz, 1H), 7.15 – 7.10 (m, 1H), 7.08 – 7.02 (m, 1H), 6.86 – 6.81 (m, 1H), 5.67 (s, 2H), 4.07 (s, 3H). ^13^C NMR (126 MHz, DMSO) δ 149.93, 142.44, 139.28, 132.93, 132.19, 130.41 (q, *J* = 30.7 Hz), 125.42, 124.57 (q, *J* = 272.4 Hz), 124.06, 118.60, 115.49, 110.24 – 110.07 (m), 110.15, 108.13 (q, *J* = 3.9 Hz), 35.46. HRMS (GC/EI+): *m/z* calculated 291.0978 for C_15_H_12_F_3_N_3_, found 291.0979 ([M]^•+^). qHNMR (400 MHz, DMSO-*d*_6_) purity = 95.7%.

#### *3-(1-Methyl-1H-pyrrolo[2*,*3-b]pyridin-3-yl)-5-(trifluoromethyl)aniline* (**41**)

Preparation according to general procedure A from **81** (144 mg, 0.5 mmol, 1.0 eq.) and 3-bromo-1-methyl-1*H*-pyrrolo[2,3-b]pyridine (**SM29**, 163 mg, 0.75 mmol, 1.5 eq.). Further purification by RP column chromatography yielded a colorless solid (55 mg, 38%). ^1^H NMR (400 MHz, DMSO) δ 8.34 (dd, *J* = 4.6, 1.5 Hz, 1H), 8.27 (dd, *J* = 8.0, 1.6 Hz, 1H), 7.98 (s, 1H), 7.25 – 7.18 (m, 2H), 7.07 – 7.02 (m, 0H), 6.77 – 6.71 (m, 1H), 5.64 (s, 2H), 3.87 (s, 3H). ^13^C NMR (101 MHz, DMSO-*d*_6_) δ 149.97, 147.87, 142.90, 136.24, 130.42 (q, *J* = 30.7 Hz), 128.13, 127.65, 126.40 – 122.87 (m), 117.40, 116.18, 114.53, 112.43, 109.53 – 109.19 (m), 107.08 – 106.78 (m), 30.96. HRMS (GC/EI+): *m/z* calculated 291.0978 for C_15_H_12_F_3_N_3_, found 291.0976 ([M]^•+^). qHNMR (400 MHz, DMSO-*d*_6_) purity = 95.0%.

#### *3-(2-Methylpyridin-4-yl)aniline* (**42**)

Preparation according to general procedure A from 2-methylpyridine-4-boronic acid (**SM69**, 83 mg, 0.61 mmol, 1.2 eq.) and 3-bromoaniline (**SM70**, 54 µL, 0.50 mmol, 1.0 eq.). Further purification by RP column chromatography yielded a colorless solid (32 mg, 34%). ^1^H NMR (400 MHz, MeOD-*d*_*4*_) δ 8.38 (d, *J* = 5.4 Hz, 1H), 7.51 – 7.49 (m, 1H), 7.44 – 7.41 (m, 1H), 7.25 – 7.16 (m, 1H), 7.07 – 7.02 (m, 1H), 7.02 – 6.98 (m, 1H), 6.83 – 6.75 (m, 1H), 2.56 (s, 3H). ^13^C NMR (101 MHz, MeOD-*d*_*4*_) δ 159.54, 151.72, 149.80, 149.58, 139.87, 130.88, 122.60, 120.30, 117.51, 117.29, 114.54, 23.80. HRMS (FIA/ESI+): *m/z* calculated 184.0995 for C_12_H_13_N_2_, found 185.1072 ([M]^•+^). qHNMR (400 MHz, DMSO-*d*_6_) purity = 98.4%.

#### *3-(2-Methylpyridin-4-yl)-4-(trifluoromethyl)aniline* (**43**)

Preparation according to general procedure A from 2-methylpyridine-4-boronic acid (**SM69**, 83 mg, 0.60 mmol, 1.2 eq.) and 3-bromo-4-(trifluoromethyl)aniline (**SM71**, 73 µL, 0.50 mmol, 1.0 eq.). Further purification by RP column chromatography yielded a colorless solid (53 mg, 42%). ^1^H NMR (400 MHz, DMSO) δ 8.45 (dd, *J* = 5.1, 0.9 Hz, 1H), 7.42 (d, *J* = 8.6 Hz, 1H), 7.16 (s, 1H), 7.12 – 7.05 (m, 1H), 6.67 (dd, *J* = 8.4, 2.3 Hz, 1H), 6.43 (d, *J* = 2.3 Hz, 1H), 5.96 (s, 2H), 2.50 (s, 3H). ^13^C NMR (101 MHz, DMSO-*d*_*6*_) δ 157.46, 151.80, 148.36, 139.44, 127.56 (q, *J* = 5.2 Hz), 125.07 (q, *J* = 271.2 Hz), 122.77, 120.76, 115.28, 112.76 (q, *J* = 30.1 Hz), 112.15, 23.98. HRMS (FIA/ESI+): *m/z* calculated 252.0869 for C_13_H_11_F_3_N_2_, found 253.0945 ([M+H]^+^). qHNMR (400 MHz, DMSO-*d*_6_) purity = 98.5%.

#### *3-Methyl-5-(2-methylpyridin-4-yl)aniline* (**44**)

Preparation according to general procedure A from 2-methylpyridine-4-boronic acid (**SM69**, 115 mg, 0.84 mmol, 1.2 eq.) and 3-bromo-5-methylaniline (**SM72**, 87 µL, 0.70 mmol, 1.0 eq.). Further purification by RP column chromatography yielded a colorless solid (98 mg, 70%). ^1^H NMR (400 MHz, DMSO-*d*_*6*_) δ 8.43 (d, *J* = 5.2 Hz, 1H), 7.44 – 7.39 (m, 1H), 7.32 (dd, *J* = 5.2, 2.1 Hz, 1H), 6.74 – 6.71 (m, 1H), 6.73 – 6.67 (m, 1H), 6.50 – 6.44 (m, 1H), 5.15 (s, 2H), 2.22 (s, 3H). ^13^C NMR (101 MHz, DMSO-*d*_*6*_) δ 158.27, 149.30, 149.24, 148.30, 138.71, 138.05, 120.27, 118.25, 115.29, 115.17, 109.33, 24.11, 21.22. HRMS (GC/EI+): *m/z* calculated 198.1151 for C_13_H_14_N_2_, found 198.1152 ([M]^+^). qHNMR (400 MHz, DMSO-*d*_6_) purity = 97.5%.

#### *3-Methoxy-5-(2-methylpyridin-4-yl)aniline* (**45**)

Preparation according to general procedure A from 2-methylpyridine-4-boronic acid (**SM69**, 83 mg, 0.60 mmol, 1.2 eq.) and 3-bromo-4-methoxyaniline (**SM73**, 69 µL, 0.50 mmol, 1.0 eq.). Further purification by RP column chromatography yielded a brown oily solid (35 mg, 32%). ^1^H NMR (500 MHz, MeOD-*d*_*4*_) δ 8.35 (d, *J* = 5.3 Hz, 1H), 7.44 (s, 1H), 7.37 (d, *J* = 5.3 Hz, 1H), 6.65 – 6.60 (m, 1H), 6.56 – 6.52 (m, 1H), 6.41 – 6.36 (m, 1H), 3.78 (s, 3H), 2.54 (s, 3H). ^13^C NMR (101 MHz, MeOD-*d*_*4*_) δ 162.48, 159.28, 151.35, 150.78, 149.31, 140.65, 122.38, 120.11, 107.44, 103.31, 102.41, 55.49, 23.61. HRMS (FIA/ESI+): *m/z* calculated 214.1101 for C_13_H_14_N_2_O, found 215.1177 ([M+H]^+^). qHNMR (400 MHz, DMSO-*d*_6_) purity = 98.2%.

#### *3-(2-Methylpyridin-4-yl)-5-(trifluoromethoxy)aniline* (**46**)

Preparation according to general procedure A from 2-methylpyridine-4-boronic acid (**SM69**, 83 mg, 0.60 mmol, 1.2 eq.) and 3-bromo-5-(trifluoromethoxy)aniline (**SM74**, 76 µL, 0.51 mmol, 1.0 eq.). Further purification by RP column chromatography yielded a colorless solid (70 mg, 52%). ^1^H NMR (400 MHz, MeOD-*d*_*4*_) δ 8.42 (d, *J* = 5.3 Hz, 1H), 7.52 – 7.47 (m, 1H), 7.43 – 7.40 (m, 1H), 6.98 – 6.93 (m, 1H), 6.82 – 6.76 (m, 1H), 6.67 – 6.61 (m, 1H), 2.58 (s, 3H^13^C NMR (101 MHz, MeOD) δ 159.90, 152.16 (q, *J* = 1.9 Hz), 152.10, 150.37, 149.85, 141.59, 122.63, 121.98 (q, *J* = 255.2 Hz), 120.30, 112.56, 108.46, 108.23, 23.83. HRMS (GC/EI): *m/z* calculated 268.0818 for C_13_H_11_F_3_N_2_O, found 268.0817 ([M]^+^). qHNMR (400 MHz, DMSO-*d*_6_) purity = 95.2%.

#### *3-Amino-5-(2-methylpyridin-4-yl)benzonitrile* (**47**)

Preparation according to general procedure A from 2-methylpyridine-4-boronic acid (**SM69**, 85 mg, 0.62 mmol, 1.2 eq.) and 3-amino-5-benzonitrile (**SM75**, 59 µL, 0.50 mmol, 1.0 eq.). Further purification by RP column chromatography yielded a colorless solid (33 mg, 31%). ^1^H NMR (500 MHz, MeOD-*d*_*4*_) δ 8.44 (d, *J* = 5.3 Hz, 1H), 7.52 (s, 1H), 7.44 (dd, *J* = 5.3, 2.0 Hz, 1H), 7.25 – 7.23 (m, 1H), 7.23 – 7.22 (m, 1H), 7.01 – 6.97 (m, 1H), 2.59 (s, 3H). ^13^C NMR (101 MHz, MeOD-*d*_*4*_) δ 160.03, 151.36, 149.95, 149.69, 141.33, 122.66, 120.27, 119.98, 119.49, 118.36, 118.04, 114.59, 23.85. HRMS (FIA/ESI+): *m/z* calculated 209.0947 for C_13_H_11_N_3_, found 210.1024 ([M+H]^+^). qHNMR (400 MHz, DMSO-*d*_6_) purity = 95.1%.

#### *3-Chloro-5-(2-methylpyridin-4-yl)aniline* (**48**)

Preparation according to general procedure A from 2-methylpyridine-4-boronic acid (**SM69**, 81 mg, 0.59 mmol, 1.2 eq.) and 3-amino-5-chloroaniline (**SM76**, 60 µL, 0.49 mmol, 1.0 eq.). Further purification by RP column chromatography yielded a colorless solid (50 mg, 46%). ^1^H NMR (400 MHz, MeOD-*d*_*4*_) δ 8.40 (d, *J* = 5.4 Hz, 1H), 7.48 (s, 1H), 7.40 (ddd, *J* = 5.4, 1.9, 0.7 Hz, 1H), 6.93 – 6.91 (m, 1H), 6.90 – 6.89 (m, 1H), 6.76 – 6.75 (m, 1H), 2.57 (s, 3H). ^13^C NMR (101 MHz, MeOD-*d*_*4*_) δ 159.80, 151.62, 150.46, 149.77, 141.47, 136.55, 122.61, 120.26, 116.37, 115.97, 112.54, 23.83. HRMS (FIA/ESI+): *m/z* calculated 218.0605 for C_12_H_11_ClN_2_, found 219.0682 ([M+H]^+^). qHNMR (400 MHz, DMSO-*d*_6_) purity = 97.9%.

#### *3*,*4-Dichloro-5-(2-methylpyridin-4-yl)aniline* (**49**)

Preparation according to general procedure A from 2-methylpyridine-4-boronic acid (**SM69**, 70 mg, 0.51 mmol, 1.0 eq.) and 3-bromo-4,5-dichloroaniline (**SM77**, 70 µL, 0.51 mmol, 1.0 eq.). Further purification by RP column chromatography yielded a yellow solid (18 mg, 14%). ^1^H NMR (400 MHz, MeOD-*d*_*4*_) δ 8.44 (d, *J* = 5.3 Hz, 1H), 7.33 – 7.28 (m, 1H), 7.24 (dd, *J* = 5.3, 1.7 Hz, 1H), 6.88 (d, *J* = 2.6 Hz, 1H), 6.57 (d, *J* = 2.7 Hz, 1H), 2.58 (s, 3H). ^13^C NMR (101 MHz, MeOD-*d*_*4*_) δ 159.26, 150.62, 149.45, 149.14, 141.33, 134.80, 125.35, 123.03, 117.65, 116.94, 116.33, 23.80. HRMS (GC/EI): *m/z* calculated 252.0216 for C_12_H_10_Cl_2_N_2_, found 252.0216 ([M]^+^). qHNMR (400 MHz, DMSO-*d*_6_) purity = 97.8%.

#### *4-(2-Methylpyridin-4-yl)-1H-indol-6-amine* (**50**)

Preparation according to general procedure A from 2-methylpyridine-4-boronic acid (**SM69**, 83 mg, 0.60 mmol, 1.0 eq.) and 4-bromo-1*H*-indol-6-amine (**SM78**, 110 mg, 0.50 mmol, 1.0 eq.). Further purification by RP column chromatography yielded a brown solid (39 mg, 35%). ^1^H NMR (400 MHz, MeOD-*d*_*4*_) δ 8.52 (d, *J* = 5.2 Hz, 1H), 7.62 – 7.61 (m, 1H), 7.56 (dd, *J* = 5.1, 1.4 Hz, 1H), 7.19 (d, *J* = 3.2 Hz, 1H), 6.89 – 6.84 (m, 1H), 6.79 (d, *J* = 1.9 Hz, 1H), 6.54 (dd, *J* = 3.3, 1.0 Hz, 1H), 2.65 (s, 3H). ^13^C NMR (101 MHz, MeOD-*d*_*4*_) δ 159.42, 151.61, 149.82, 144.72, 139.67, 131.75, 124.66, 124.04, 121.94, 119.92, 111.29, 101.14, 98.65, 24.38. HRMS (GC/EI): *m/z* calculated 223.1103 for C_**14**_H_**13**_N_**3**_, found 223.1103 ([M]^+^). qHNMR (400 MHz, DMSO-*d*_6_) purity = 97.9%.

#### *6-(2-Methylpyridin-4-yl)-1H-indol-4-amine* (**51**)

Preparation according to general procedure A from 2-methylpyridine-4-boronic acid (**SM69**, 863 mg, 0.63 mmol, 1.0 eq.) and 6-bromo-1*H*-indol-4-amine (**SM79**, 114 mg, 0.51 mmol, 1.0 eq.). Further purification by RP column chromatography yielded a brown solid (52 mg, 46%). ^1^H NMR (400 MHz, MeOD-*d*_*4*_) δ 8.33 (d, *J* = 5.3 Hz, 1H), 7.56 – 7.52 (m, 1H), 7.49 – 7.44 (m, 1H), 7.20 – 7.19 (m, 1H), 7.19 – 7.19 (m, 1H), 6.74 (d, *J* = 1.5 Hz, 1H), 6.55 (dd, *J* = 3.2, 1.0 Hz, 1H), 2.55 (s, 3H). ^13^C NMR (101 MHz, MeOD-*d*_*4*_) δ 159.14, 153.09, 149.28, 141.67, 139.01, 133.23, 125.27, 122.52, 120.39, 120.30, 103.55, 102.21, 99.62, 23.82. HRMS (GC/EI): *m/z* calculated 223.1103 for C_14_H_13_N_3_, found 223.1102 ([M]^+^). qHNMR (400 MHz, DMSO-*d*_6_) purity = 98.3%.

#### *4-(1-Methyl-1H-pyrrolo[2*,*3-b]pyridin-5-yl)-1H-indol-6-amine* (**52**)

Preparation according to general procedure A from 1-methyl-5-(4,4,5,5-tetramethyl-1,3,2-dioxaborolan-2-yl)-1*H*-pyrrolo[2,3-b]pyridine (**SM80**, 156 mg, 0.57 mmol, 1.2 eq.) and bromo-1*H*-indol-6-amine (**SM78**, 105 mg, 0.48 mmol, 1.0 eq.). Further purification by RP column chromatography yielded a colorless solid (46 mg, 37%). ^1^H NMR (400 MHz, DMSO-*d*_6_) δ 10.64 (s, 1H), 8.49 (d, *J* = 2.0 Hz, 1H), 8.11 (d, *J* = 2.1 Hz, 1H), 7.54 (d, *J* = 3.4 Hz, 1H), 7.08 – 7.02 (m, 1H), 6.62 – 6.57 (m, 1H), 6.56 – 6.47 (m, 2H), 6.34 – 6.28 (m, 1H), 4.81 (s, 2H), 3.87 (s, 3H). ^13^C NMR (101 MHz, DMSO-*d*_6_) δ 146.67, 144.09, 142.35, 138.09, 131.28, 130.50, 129.13, 127.40, 122.44, 119.84, 118.13, 109.71, 99.86, 99.06, 94.43, 30.94. HRMS (DEP/EI+): *m*/*z* calculated 262.1213 for C_16_H_14_N_4_, found 262.1224 ([M]^•+^). qHNMR (400 MHz, DMSO-*d*_6_) purity = 95.7%.

#### *2’-Methyl-5-(trifluoromethyl)-[1*,*1’-biphenyl]-3-amine* (**53**)

Preparation according to general procedure A from 2-tolylboronic acid (**SM52**, 87 mg, 0.61 mmol, 1.2 eq.) and 3-bromo-5-(trifluoromethyl)aniline (**SM1**, 72 µL, 0.51 mmol, 1.0 eq.). Further purification by RP column chromatography yielded a colorless oil (60 mg, 47%). ^1^H NMR (400 MHz, DMSO-*d*_*6*_) δ 7.31 – 7.21 (m, 3H), 7.19 – 7.15 (m, 1H), 6.87 – 6.82 (m, 1H), 6.77 – 6.71 (m, 1H), 6.69 – 6.64 (m, 1H), 5.65 (s, 2H), 2.21 (s, 3H). ^13^C NMR (101 MHz, DMSO-*d*_*6*_) δ 149.38, 142.89, 140.68, 134.53, 130.33, 129.69 (q, *J* = 30.8 Hz), 129.13, 127.57, 125.94, 124.49 (q, *J* = 272.6 Hz), 117.71, 112.03 (q, *J* = 4.2 Hz), 108.16 (q, *J* = 4.1 Hz), 19.98. HRMS (GC/EI+): *m/z* calculated 251.0916 for C_14_H_12_F_3_N, found 251.0918 ([M]^•+^). qHNMR (400 MHz, DMSO-*d*_6_) purity = 98.7%.

#### *3’-Methyl-5-(trifluoromethyl)-[1*,*1’-biphenyl]-3-amine* (**54**)

Preparation according to general procedure A from 3-tolylboronic acid (**SM53**, 70 mg, 0.50 mmol, 1.0 eq.) and 3-bromo-5-(trifluoromethyl)aniline (**SM1**, 74 µL, 0.53 mmol, 1.1 eq.). Further purification by RP column chromatography yielded a yellow oil (29 mg, 23%). ^1^H NMR (400 MHz, DMSO-*d*_*6*_) δ 7.44 – 7.40 (m, 1H), 7.40 – 7.31 (m, 2H), 7.23 – 7.16 (m, 1H), 7.09 – 7.03 (m, 1H), 7.01 – 6.95 (m, 1H), 6.88 – 6.81 (m, 1H), 5.67 (s, 2H), 2.37 (s, 3H). ^13^C NMR (101 MHz, DMSO-*d*_*6*_) δ 149.95, 142.12, 139.60, 138.17, 130.40 (q, *J* = 30.6 Hz), 128.87, 128.54, 127.29, 128.79 – 120.36 (m), 123.76, 115.33, 109.96 (d, *J* = 4.2 Hz), 108.58 (d, *J* = 3.8 Hz), 21.06. HRMS (GC/EI+): *m/z* calculated 251.0916 for C_14_H_12_F_3_N, found 251.0917 ([M]^•+^). qHNMR (400 MHz, DMSO-*d*_6_) purity = 95.8%.

#### *4’-Methyl-5-(trifluoromethyl)-[1*,*1’-biphenyl]-3-amine* (**55**)

Preparation according to general procedure A from 4-tolylboronic acid (**SM54**, 71 mg, 0.50 mmol, 1.0 eq.) and 3-bromo-5-(trifluoromethyl)aniline (**SM1**, 74 µL, 0.53 mmol, 1.1 eq.). Further purification by RP column chromatography yielded a colorless solid (49 mg, 39%). ^1^H NMR (400 MHz, DMSO) δ 7.49 (d, *J* = 8.3 Hz, 2H), 7.26 (d, *J* = 7.7 Hz, 2H), 7.08 – 7.02 (m, 1H), 7.00 – 6.94 (m, 1H), 6.85 – 6.80 (m, 1H), 5.66 (s, 2H), 2.34 (s, 3H). ^13^C NMR (101 MHz, DMSO) δ 149.94, 141.88, 137.24, 136.71, 130.39 (q, *J* = 30.9 Hz), 129.54, 126.45, 124.53 (q, *J* = 272.5 Hz), 115.03, 109.70 (q, *J* = 3.8 Hz), 108.37 (q, *J* = 3.8 Hz), 20.66. HRMS (GC/EI+): *m/z* calculated 251.0916 for C_14_H_12_F_3_N, found 251.0916 ([M]^•+^). qHNMR (400 MHz, DMSO-*d*_6_) purity = 96.4%.

#### *2’*,*3’-Dimethyl-5-(trifluoromethyl)-[1*,*1’-biphenyl]-3-amine* (**56**)

Preparation according to general procedure A from **81** (146 mg, 0.51 mmol, 1.0 eq.) and 1-bromo-2,3-dimethylbenzene (**SM30**, 90 µL, 0.66 mmol, 1.3 eq.). Further purification by RP column chromatography yielded a colorless solid (80 mg, 60%). ^1^H NMR (400 MHz, DMSO-*d*_*6*_) δ 7.17 (d, *J* = 7.2 Hz, 1H), 7.12 (t, *J* = 7.5 Hz, 1H), 6.99 (dd, *J* = 7.5, 1.6 Hz, 1H), 6.86 – 6.81 (m, 1H), 6.72 – 6.66 (m, 1H), 6.64 – 6.59 (m, 1H), 5.65 (s, 2H), 2.28 (s, 3H), 2.08 (s, 3H). ^13^C NMR (101 MHz, DMSO-*d*_*6*_) δ 149.32, 143.57, 141.00, 136.89, 133.20, 129.59 (q, *J* = 30.8 Hz), 128.97, 126.92, 125.30, 124.50 (q, *J* = 272.5 Hz), 117.86, 112.22 (q, *J* = 3.6 Hz), 108.02 (q, *J* = 3.3 Hz), 20.27, 16.54. HRMS (GC/EI+): *m/z* calculated 265.1073 for C_15_H_14_F_3_N, found 265.1073 ([M]^•+^). qHNMR (400 MHz, DMSO-*d*_6_) purity = 98.6%.

#### *3’*,*4’-Dimethyl-5-(trifluoromethyl)-[1*,*1’-biphenyl]-3-amine* (**57**)

Preparation according to general procedure A from 3,4-dimethylphenylboronic acid (**SM55**, 70 µL, 0.50 mmol, 1.0 eq.) and 3-bromo-5-(trifluoromethyl)aniline (**SM1**, 74 µL, 0.53 mmol, 1.1 eq.). Further purification by RP column chromatography yielded a yellow oil (68 mg, 51%). ^1^H NMR (400 MHz, DMSO-*d*_*6*_) δ 7.38 (d, *J* = 2.0 Hz, 1H), 7.31 (dd, *J* = 7.8, 2.0 Hz, 1H), 7.21 (d, *J* = 7.9 Hz, 1H), 7.08 – 7.02 (m, 1H), 6.99 – 6.95 (m, 1H), 6.85 – 6.79 (m, 1H), 5.64 (s, 2H), 2.28 (s, 3H), 2.25 (s, 3H). ^13^C NMR (101 MHz, DMSO) δ 149.88, 142.00, 137.07, 136.76, 135.99, 130.33 (q, *J* = 30.7 Hz), 130.03, 127.63, 124.56 (q, *J* = 272.6 Hz), 123.88, 115.04, 109.71 (q, *J* = 4.0 Hz), 108.30 (q, *J* = 3.9 Hz), 19.45, 19.03. HRMS (GC/EI+): *m/z* calculated 265.1073 for C_15_H_14_F_3_N, found 265.1073 ([M]^•+^). qHNMR (400 MHz, DMSO-*d*_6_) purity = 95.7%.

#### *3’-Ethyl-5-(trifluoromethyl)-[1*,*1’-biphenyl]-3-amine* (**58**)

Preparation according to general procedure A from 3-ethylphenylboronic acid (**SM56**, 100 mg, 0.65 mmol, 1.0 eq.) and 3-bromo-5-(trifluoromethyl)aniline (**SM1**, 96 µL, 0.68 mmol, 1.1 eq.). Further purification by RP column chromatography yielded a yellowish oily solid (148 mg, 86%). ^1^H NMR (400 MHz, DMSO-*d*_*6*_) δ 7.45 (t, *J* = 1.8 Hz, 1H), 7.42 (dt, *J* = 7.7, 1.7 Hz, 1H), 7.38 (t, *J* = 7.5 Hz, 1H), 7.24 (dt, *J* = 7.3, 1.6 Hz, 1H), 7.22 – 7.19 (m, 1H), 7.18 – 7.12 (m, 1H), 7.01 – 6.95 (m, 1H), 6.00 (s, 2H), 2.67 (q, *J* = 7.6 Hz, 2H), 1.22 (t, *J* = 7.6 Hz, 3H). ^13^C NMR (101 MHz, DMSO-*d*_*6*_) δ 147.61, 144.57, 142.32, 139.32, 130.43 (q, *J* = 31.0 Hz), 128.97, 127.49, 126.20, 124.37 (q, *J* = 273.0 Hz), 124.07, 116.82, 111.81 (q, *J* = 3.9 Hz), 110.01 (q, *J* = 3.3 Hz), 28.19, 15.70. HRMS (GC/EI+): *m/z* calculated 265.1073 for C_15_H_14_F_3_N, found 265.1077 ([M]^•+^). qHNMR (400 MHz, DMSO-*d*_6_) purity = 96.1%.

#### *3’-(Tert-butyl)-5-(trifluoromethyl)-[1*,*1’-biphenyl]-3-amine* (**59**)

Preparation according to general procedure A from 3-*tert*-butylphenylboronic acid (**SM57**, 196 mg, 1.1 mmol, 1.1 eq.) and 3-bromo-5-(trifluoromethyl)aniline (**SM1**, 141 µL, 1.0 mmol, 1.0 eq.). Further purification by RP column chromatography yielded a light-yellow oil (251 mg, 86%). ^1^H NMR (400 MHz, CD_2_Cl_2_) δ 7.58 (dt, *J* = 2.6, 1.0 Hz, 1H), 7.46 – 7.36 (m, 3H), 7.19 (tq, *J* = 1.5, 0.7 Hz, 1H), 7.06 (ddd, *J* = 2.2, 1.6, 0.6 Hz, 1H), 6.88 (ddt, *J* = 2.2, 1.6, 0.7 Hz, 1H), 4.02 (s, 2H), 1.37 (s, 9H). ^13^C NMR (101 MHz, CD_2_Cl_2_) δ 152.31, 147.96, 144.18, 140.23, 132.13 (q, *J* = 31.4 Hz), 128.94, 125.41, 124.79 (q, *J* = 272.2 Hz), 124.63, 124.57, 117.02, 114.07 (q, *J* = 3.9 Hz), 110.22 (q, *J* = 3.9 Hz), 35.13, 31.52. HRMS (GC/EI+): *m/z* calculated 293.1386 for C_17_H_18_F_3_N, found 293.1387 ([M]^•+^). qHNMR (400 MHz, DMSO-*d*_6_) purity = 98.6%.

#### *1-(3’-Amino-5’-(trifluoromethyl)-[1*,*1’-biphenyl]-3-yl)ethan-1-one* (**60**)

Preparation according to general procedure A from **81** (145 mg, 0.50 mmol, 1.0 eq.) and 3’-bromoacetophenone (**SM57**, 80 µL, 0.60 mmol, 1.2 eq.). Further purification by RP column chromatography yielded a colorless solid (113 mg, 80%). ^1^H NMR (400 MHz, DMSO-*d*_*6*_) δ 8.12 (t, *J* = 1.8 Hz, 1H), 8.01 – 7.94 (m, 1H), 7.91 – 7.84 (m, 1H), 7.62 (t, *J* = 7.8 Hz, 1H), 7.17 – 7.11 (m, 1H), 7.10 – 7.05 (m, 1H), 6.92 – 6.87 (m, 1H), 5.74 (s, 2H), 2.65 (s, 3H). ^13^C NMR (101 MHz, DMSO-*d*_*6*_) δ 197.92, 150.09, 141.12, 139.99, 137.43, 131.31, 130.58 (q, *J* = 31.0 Hz), 129.43, 127.69, 126.10, 124.45 (q, *J* = 272.4 Hz), 115.37, 110.00 (q, *J* = 3.5 Hz), 109.03 (q, *J* = 4.2 Hz), 26.88. HRMS (GC/EI+): *m/z* calculated 279.0866 for C_15_H_12_F_3_NO, found 279.0868 ([M]^•+^). qHNMR (400 MHz, DMSO-*d*_6_) purity = 98.9%.

#### *3’-Fluoro-5-(trifluoromethyl)-[1*,*1’-biphenyl]-3-amine* (**61**)

Preparation according to general procedure A from 3-fluorophenylboronic acid (**SM58**, 87 mg, 0.63 mmol, 1.2 eq.) and 3-bromo-5-(trifluoromethyl)aniline (**SM1**, 74 µL, 0.52 mmol, 1.0 eq.). Further purification by RP column chromatography yielded a colorless oil (67 mg, 51%). ^1^H NMR (400 MHz, DMSO-*d*_*6*_) δ 7.55 – 7.40 (m, 3H), 7.26 – 7.17 (m, 1H), 7.11 – 7.07 (m, 1H), 7.06 – 7.01 (m, 1H), 6.91 – 6.85 (m, 1H), 5.71 (s, 2H). ^13^C NMR (101 MHz, DMSO-*d*_*6*_) δ 162.60 (d, *J* = 243.6 Hz), 150.01, 142.08 (d, *J* = 7.8 Hz), 140.61 (d, *J* = 2.3 Hz), 130.90 (d, *J* = 8.6 Hz), 130.54 (q, *J* = 30.9 Hz), 124.42 (q, *J* = 272.5 Hz), 122.76 (d, *J* = 2.6 Hz), 115.35, 114.59 (d, *J* = 21.0 Hz), 113.42 (d, *J* = 22.1 Hz), 110.05 (q, *J* = 4.0 Hz), 109.15 (q, *J* = 3.6 Hz). HRMS (GC/EI+): *m/z* calculated 255.0666 for C_13_H_9_F_4_N, found 255.0669 ([M]^•+^). qHNMR (400 MHz, DMSO-*d*_6_) purity = 97.3%.

#### *3’-Chloro-5-(trifluoromethyl)-[1*,*1’-biphenyl]-3-amine* (**62**)

Preparation according to general procedure A from 3-chlorophenylboronic acid (**SM59**, 87 mg, 0.56 mmol, 1.1 eq.) and 3-bromo-5-(trifluoromethyl)aniline (**SM1**, 72 µL, 0.51 mmol, 1.0 eq.). Further purification by RP column chromatography yielded a yellowish solid (89 mg, 64%). ^1^H NMR (400 MHz, DMSO-*d*_*6*_) δ 7.65 (t, *J* = 1.9 Hz, 1H), 7.58 (dt, *J* = 7.5, 1.6 Hz, 1H), 7.49 (t, *J* = 7.7 Hz, 1H), 7.45 (dt, *J* = 8.1, 1.6 Hz, 1H), 7.11 – 7.06 (m, 1H), 7.06 – 7.01 (m, 1H), 6.91 – 6.86 (m, 1H), 5.72 (s, 2H). ^13^C NMR (101 MHz, DMSO-*d*_*6*_) δ 150.04, 141.75, 140.42, 133.71, 130.80, 130.57 (q, *J* = 31.1 Hz), 127.72, 126.42, 125.40, 124.40 (q, *J* = 272.4 Hz), 115.36, 110.03 (q, *J* = 3.3 Hz), 109.18 (q, *J* = 3.8 Hz). HRMS (GC/EI+): *m/z* calculated 271.0370 for C_13_H_9_ClF_3_N, found 271.0376 ([M]^•+^). qHNMR (400 MHz, DMSO-*d*_6_) purity = 97.0%.

#### *3’*,*5-Bis(trifluoromethyl)-[1*,*1’-biphenyl]-3-amine* (**63**)

Preparation according to general procedure A from 3-(trifluoromethyl)phenylboronic acid (**SM60**, 139 mg, 0.73 mmol, 1.1 eq.) and 3-bromo-5-(trifluoromethyl)aniline (**SM1**, 94 µL, 0.67 mmol, 1.0 eq.). Further purification by RP column chromatography yielded a colorless solid (128 mg, 63%). ^1^H NMR (400 MHz, DMSO-*d*_*6*_) δ 7.93 (d, *J* = 7.7 Hz, 1H), 7.91 – 7.88 (m, 1H), 7.72 (dt, *J* = 15.4, 7.7 Hz, 2H), 7.17 – 7.12 (m, 1H), 7.11 – 7.06 (m, 1H), 6.94 – 6.88 (m, 1H), 5.75 (s, 2H). ^13^C NMR (101 MHz, DMSO-*d*_*6*_) δ 150.13, 140.64, 140.36, 130.84, 130.66 (q, *J* = 30.8 Hz), 130.11, 129.58 (q, *J* = 31.3 Hz), 124.85 (q, *J* = 271.6 Hz), 124.48 (q, *J* = 3.7 Hz), 124.17 (q, *J* = 272.8 Hz), 123.08 (q, *J* = 3.6 Hz), 115.44, 110.10 (q, *J* = 3.8 Hz), 109.30 (q, *J* = 3.5 Hz). HRMS (GC/EI+): *m/z* calculated 305.0634 for C_14_H_9_F_6_N, found 305.0639 ([M]^•+^). qHNMR (400 MHz, DMSO-*d*_6_) purity = 98.0%.

#### *3’-(Trifluoromethoxy)-5-(trifluoromethyl)-[1*,*1’-biphenyl]-3-amine* (**64**)

Preparation according to general procedure A from 3-trifluoromethoxyphenylboronic acid (**SM61**, 227 mg, 1.1 mmol, 1.1 eq.) and 3-bromo-5-(trifluoromethyl)aniline (**SM1**, 141 µL, 1.0 mmol, 1.0 eq.). Further purification by RP column chromatography yielded a colorless solid (206 mg, 64%). ^1^H NMR (400 MHz, CD_2_Cl_2_) δ 7.53 (dt, *J* = 7.8, 1.5 Hz, 1H), 7.49 (td, *J* = 7.8, 0.6 Hz, 1H), 7.43 (ddp, *J* = 2.5, 1.5, 0.9 Hz, 1H), 7.26 (ddq, *J* = 7.8, 2.5, 1.2 Hz, 1H), 7.17 (tt, *J* = 1.5, 0.8 Hz, 1H), 7.04 (ddd, *J* = 2.2, 1.6, 0.6 Hz, 1H), 6.92 (ddq, *J* = 2.2, 1.3, 0.7 Hz, 1H), 4.06 (s, 2H). ^13^C NMR (101 MHz, CD_2_Cl_2_) δ 150.03 (q, *J* = 1.9 Hz), 148.19, 142.67, 141.96, 132.45 (q, *J* = 31.8 Hz), 130.71, 126.02, 124.61 (q, *J* = 272.5 Hz), 120.96 (q, *J* = 257.1 Hz), 120.62, 120.12, 116.77, 113.86 (q, *J* = 3.9 Hz), 111.02 (q, *J* = 3.8 Hz). HRMS (GC/EI+): *m/z* calculated 321.0583 for C_14_H_19_F_6_NO, found 321.0582 ([M]^•+^). qHNMR (400 MHz, DMSO-*d*_6_) purity = 99.4%.

#### *3’-Methoxy-5-(trifluoromethyl)-[1*,*1’-biphenyl]-3-amine* (**65**)

Preparation according to general procedure A from 3-methoxyphenylboronic acid (**SM62**, 167 mg, 1.1 mmol, 1.1 eq.) and 3-bromo-5-(trifluoromethyl)aniline (**SM1**, 141 µL, 1.0 mmol, 1.0 eq.). Further purification by RP column chromatography yielded a light-yellow oily solid (224 mg, 84%). ^1^H NMR (400 MHz, CD_2_Cl_2_) δ 7.36 (ddd, *J* = 8.2, 7.6, 0.4 Hz, 1H), 7.19 (tt, *J* = 1.5, 0.8 Hz, 1H), 7.15 (ddd, *J* = 7.7, 1.7, 1.0 Hz, 1H), 7.09 (dd, *J* = 2.6, 1.7 Hz, 1H), 7.05 (ddt, *J* = 2.2, 1.6, 0.6 Hz, 1H), 6.92 (ddd, *J* = 8.3, 2.6, 0.9 Hz, 1H), 6.89 (ddt, *J* = 2.3, 1.6, 0.7 Hz, 1H), 4.02 (s, 2H), 3.85 (s, 3H). ^13^C NMR (101 MHz, CD_2_Cl_2_) δ 160.50, 147.98, 143.40, 141.93, 132.16 (q, *J* = 31.5 Hz), 130.25, 124.74 (q, *J* = 272.2 Hz), 119.79, 116.89, 113.98 (q, *J* = 3.8 Hz), 113.72, 113.09, 110.48 (q, *J* = 3.8 Hz), 55.72. HRMS (GC/EI+): *m/z* calculated 267.0866 for C_14_H_12_F_3_NO, found 267.0864 ([M]^•+^). qHNMR (400 MHz, DMSO-*d*_6_) purity = 99.8%.

#### *N*^*3*^,*N*^*3*^*-Dimethyl-5’-(trifluoromethyl)-[1*,*1’-biphenyl]-3*,*3’-diamine* (**66**)

Preparation according to general procedure A from 3-(*N*,*N*-dimethylamino)phenylboronic acid (**SM63**, 182 mg, 1.1 mmol, 1.1 eq.) and 3-bromo-5-(trifluoromethyl)aniline (**SM1**, 141 µL, 1.0 mmol, 1.0 eq.). Further purification by RP column chromatography yielded a light-yellow oil (221 mg, 79%). ^1^H NMR (500 MHz, CD_2_Cl_2_) δ 7.28 (ddd, *J* = 8.2, 7.3, 0.7 Hz, 1H), 7.19 (td, *J* = 1.5, 0.7 Hz, 1H), 7.06 (dt, *J* = 2.1, 1.0 Hz, 1H), 6.90 – 6.85 (m, 3H), 6.75 (ddd, *J* = 8.3, 2.5, 1.0 Hz, 1H), 4.00 (s, 2H), 2.99 (s, 6H). ^13^C NMR (126 MHz, CD_2_Cl_2_) δ 151.51, 147.86, 144.58, 141.34, 131.98 (q, *J* = 31.6 Hz), 129.81, 124.81 (q, *J* = 272.3 Hz), 117.05, 115.63, 114.11 (q, *J* = 4.1 Hz), 112.49, 111.45, 110.17 (q, *J* = 3.8 Hz), 40.83. HRMS (GC/EI+): *m/z* calculated 280.1182 for C_15_H_15_F_3_N_2_, found 280.1187 ([M]^•+^). qHNMR (400 MHz, DMSO-*d*_6_) purity = 99.0%.

#### *3’-Amino-5’-(trifluoromethyl)-[1*,*1’-biphenyl]-3-ol* (**67**)

Preparation according to general procedure A from 3-hydroxyphenylboronic acid (**SM64**, 152 mg, 1.1 mmol, 1.1 eq.) and 3-bromo-5-(trifluoromethyl)aniline (**SM1**, 141 µL, 1.0 mmol, 1.0 eq.). Further purification by RP column chromatography yielded a colorless solid (166 mg, 66 ^1^H NMR (500 MHz, CD_2_Cl_2_) δ 7.31 (t, *J* = 7.8 Hz, 1H), 7.20 – 7.16 (m, 1H), 7.15 – 7.12 (m, 1H), 7.06 – 7.01 (m, 2H), 6.90 – 6.87 (m, 1H), 6.87 – 6.82 (m, 1H), 5.16 (s, 1H), 4.02 (s, 2H). ^13^C NMR (126 MHz, CD_2_Cl_2_) δ 156.53, 147.96, 143.06, 142.16, 132.18 (q, *J* = 31.8 Hz), 130.49, 124.71 (q, *J* = 272.2 Hz), 119.86, 116.82, 115.19, 114.31, 113.95 (q, *J* = 3.9 Hz), 110.57 (q, *J* = 3.8 Hz). HRMS (GC/EI+): *m/z* calculated 253.0709 for C_13_H_10_F_3_NO, found 253.0708 ([M]^•+^). qHNMR (400 MHz, DMSO-*d*_6_) purity = 98.6%.

#### *3-(Naphthalen-2-yl)-5-(trifluoromethyl)aniline* (**68**)

Preparation according to general procedure A from 3-naphthaleneboronic acid (**SM65**, 95 mg, 0.55 mmol, 1.1 eq.) and 3-bromo-5-(trifluoromethyl)aniline (**SM1**, 71 µL, 0.50 mmol, 1.0 eq.). Further purification by RP column chromatography yielded a colorless solid (103 mg, 71%). ^1^H NMR (400 MHz, DMSO-*d*_*6*_) δ 8.18 (s, 1H), 8.06 – 7.90 (m, 3H), 7.77 (dd, *J* = 8.5, 1.9 Hz, 1H), 7.61 – 7.49 (m, 2H), 7.26 – 7.21 (m, 1H), 7.19 – 7.15 (m, 1H), 6.93 – 6.87 (m, 1H), 5.74 (s, 2H). ^13^C NMR (126 MHz, DMSO-*d*_*6*_) δ 150.05, 141.81, 136.92, 133.23, 132.44, 130.54 (q, *J* = 30.8 Hz), 128.53, 128.25, 127.49, 126.50, 126.31, 125.36, 124.91, 124.55 (q, *J* = 272.2 Hz), 115.57, 110.22 (q, *J* = 4.1 Hz), 108.73 (q, *J* = 3.9 Hz). HRMS (GC/EI+): *m/z* calculated 287.0916 for C_17_H_12_F_3_N, found 287.0920 ([M]^•+^). qHNMR (400 MHz, DMSO-*d*_6_) purity = 96.0%.

#### *3-(Naphthalen-1-yl)-5-(trifluoromethyl)aniline* (**69**)

Preparation according to general procedure A from **81** (146 mg, 0.51 mmol, 1.0 eq.) and 1-bromonapthalene (**SM32**, 85 µL, 0.61 mmol, 1.2 eq.). Further purification by RP column chromatography yielded a colorless oily solid (79 mg, 54%). ^1^H NMR (400 MHz, DMSO-*d*_*6*_) δ 8.02 – 7.98 (m, 1H), 7.96 (d, *J* = 8.3 Hz, 1H), 7.82 – 7.77 (m, 1H), 7.60 – 7.49 (m, 3H), 7.43 (dd, *J* = 7.0, 1.3 Hz, 1H), 6.98 – 6.93 (m, 1H), 6.92 – 6.88 (m, 1H), 6.83 – 6.78 (m, 1H), 5.76 (s, 2H). ^13^C NMR (101 MHz, DMSO-*d*_*6*_) δ 149.57, 141.64, 138.83, 133.37, 130.64, 129.97 (q, *J* = 30.8 Hz), 128.40, 127.92, 126.59, 126.50, 126.00, 125.53, 125.04, 124.50 (q, *J* = 273.7 Hz), 118.42, 112.69 (q, *J* = 4.1 Hz), 108.63 (q, *J* = 3.6 Hz). HRMS (GC/EI+): *m/z* calculated 287.0916 for C_17_H_12_F_3_N, found 287.0920 ([M]^•+^). qHNMR (400 MHz, DMSO-*d*_6_) purity = 95.5%.

#### *3-(5-Methylthiophen-2-yl)-5-(trifluoromethyl)aniline* (**70**)

Preparation according to general procedure A from 5-methylthiphene-2-boronic acid (**SM66**, 71 mg, 0.5 mmol, 1.0 eq.) and 3-bromo-5-(trifluoromethyl)aniline (**SM1**, 74 µL, 0.53 mmol, 1.1 eq.). Further purification by RP column chromatography yielded a pale-yellow solid (18 mg, 14%). ^1^H NMR (400 MHz, MeOD) δ 7.16 (d, *J* = 3.6 Hz, 1H), 7.08 – 7.06 (m, 1H), 7.03 – 7.00 (m, 1H), 6.83 – 6.78 (m, 1H), 6.76 – 6.74 (m, 1H), 2.49 (s, 3H). ^13^C NMR (101 MHz, MeOD-*d*_*4*_) δ 150.64, 142.12, 141.13, 137.61, 132.99 (q, *J* = 31.4 Hz), 127.43, 127.90 – 124.08 (m), 124.70, 115.30, 111.39 – 111.17 (m), 110.68 – 110.40 (m), 15.25. HRMS (GC/EI+): *m/z* calculated 257.0481 for C_12_H_10_F_3_NS, found 257.0484 ([M]^•+^). qHNMR (400 MHz, DMSO-*d*_6_) purity = 98.0%.

#### *3-(5-Methylthiophen-3-yl)-5-(trifluoromethyl)aniline* (**71**)

Preparation according to general procedure A from **81** (147 mg, 0.51 mmol, 1.0 eq.) and 4-bromo-2-methylthiophene (**SM33**, 86 µL, 0.77 mmol, 1.5 eq.). Further purification by RP column chromatography yielded a colorless solid (57 mg, 44%). ^1^H NMR (400 MHz, DMSO-*d*_*6*_) δ 7.59 (d, *J* = 1.6 Hz, 1H), 7.18 (s, 1H), 7.16 – 7.10 (m, 2H), 6.85 (s, 1H), 5.52 (s, 2H), 2.48 (s, 3H). ^13^C NMR (101 MHz, DMSO-*d*_*6*_) δ 147.53, 140.40, 140.06, 137.05, 130.45 (q, *J* = 30.9 Hz), 124.42, 124.37 (q, *J* = 272.1 Hz), 119.73, 115.78, 111.18, 109.73, 15.04. HRMS (GC/EI+): *m/z* calculated 257.0481 for C_12_H_10_F_3_NS, found 257.0481 ([M]^•+^). qHNMR (400 MHz, DMSO-*d*_6_) purity = 97.0%.

#### *3-(3*,*5-Dimethylthiophen-2-yl)-5-(trifluoromethyl)aniline* (**72**)

Preparation according to general procedure A from **81** (144 mg, 0.5 mmol, 1.0 eq.) and 2-bromo-3,5-dimethyl thiophene (**SM34**, 149 mg, 101 µL, 0.75 mmol, 1.5 eq.). Further purification by RP column chromatography yielded a colorless oily solid (103 mg, 76%). ^1^H NMR (400 MHz, DMSO-*d*_*6*_) δ 6.89 – 6.83 (m, 1H), 6.81 – 6.76 (m, 1H), 6.76 – 6.70 (m, 1H), 6.67 (s, 1H), 5.72 (s, 2H), 2.40 (s, 3H), 2.20 (s, 3H). ^13^C NMR (101 MHz, DMSO-*d*_*6*_) δ 149.81, 137.44, 135.82, 133.53, 133.21, 130.15 (q, *J* = 31.1 Hz), 130.08, 128.46 – 120.11 (m), 116.45, 111.19 – 110.91 (m), 108.29 – 107.99 (m), 14.86, 14.79. HRMS (GC/EI+): *m/z* calculated 271.0637 for C_13_H_12_F_3_NS, found 271.0641 ([M]^•+^). qHNMR (400 MHz, DMSO-*d*_6_) purity = 97.8%.

#### *1-(5-(3-Amino-5-(trifluoromethyl)phenyl)thiophen-2-yl)ethan-1-one* (**73**)

Preparation according to general procedure A from **81** (150 mg, 0.52 mmol, 1.0 eq.) and 2-acetyl-5-bromothiophene (**SM35**, 66 µL, 0.53 mmol, 1.1 eq.). Further purification by RP column chromatography yielded a colorless solid (70 mg, 49%). ^1^H NMR (400 MHz, DMSO-*d*_*6*_) δ 7.93 (d, *J* = 3.9 Hz, 1H), 7.64 (d, *J* = 4.0 Hz, 1H), 7.15 (s, 2H), 6.88 (s, 1H), 5.83 (s, 2H), 2.54 (s, 3H). ^13^C NMR (101 MHz, DMSO-*d*_*6*_) δ 190.68, 150.44, 150.23, 142.99, 135.05, 134.26, 130.85 (q, *J* = 31.4 Hz), 125.59, 124.17 (q, *J* = 272.4 Hz), 114.01, 110.16 – 109.87 (m), 109.03 – 108.75 (m), 26.39. HRMS (GC/EI+): *m/z* calculated 285.0430 for C_13_H_10_F_3_NOS, found 285.0433 ([M]^•+^). qHNMR (400 MHz, DMSO-*d*_6_) purity = 95.5%.

#### *1-(4-(3-Amino-5-(trifluoromethyl)phenyl)thiophen-2-yl)ethan-1-one* (**74**)

Preparation according to general procedure A from **81** (144 mg, 0.5 mmol, 1.0 eq.) and 2-acetyl-4-bromo thiophene (**SM36**, 159 mg, 99 µL, 0.75 mmol, 1.5 eq.). Further purification by flash column yielded a yellowish solid (136 mg, 96%). ^1^H NMR (400 MHz, DMSO-*d*_6_) δ 8.33 (d, *J* = 1.5 Hz, 1H), 8.26 (d, *J* = 1.5 Hz, 1H), 7.22 – 7.18 (m, 1H), 7.18 – 7.14 (m, 1H), 6.87 – 6.81 (m, 1H), 5.67 (s, 2H), 2.61 (s, 3H). ^13^C NMR (101 MHz, DMSO-*d*_6_) δ 191.08, 149.93, 144.77, 141.79, 135.81, 132.34, 130.56 (q, *J* = 31.1 Hz), 129.88, 128.78 – 120.10 (m), 114.41, 109.87 – 109.45 (m), 109.15 – 108.81 (m), 26.70. HRMS (GC/EI+): *m/z* calculated 285.0430 for C_13_H_10_F_3_NOS, found 285.0431 ([M]^•+^). qHNMR (400 MHz, DMSO-*d*_6_) purity = 95.7%.

#### *Methyl 5-(3-amino-5-(trifluoromethyl)phenyl)thiophene-2-carboxylate* (**75**)

Preparation according to general procedure A from **81** (144 mg, 0.5 mmol, 1.0 eq.) and methyl 5-bromothiophene 2-carboxylate (**SM37**, 167 mg, 100 µL, 0.75 mmol, 1.5 eq.). Further purification by RP column chromatography yielded a colorless solid (112 mg, 74%). ^1^H NMR (400 MHz, DMSO-*d*_*6*_) δ 7.79 (d, *J* = 4.0 Hz, 1H), 7.59 (d, *J* = 4.0 Hz, 1H), 7.15 – 7.10 (m, 2H), 6.91 – 6.85 (m, 1H), 5.83 (s, 2H), 3.84 (s, 3H). ^13^C NMR (101 MHz, DMSO-*d*_*6*_) δ 161.68, 150.24, 149.47, 134.77, 134.09, 131.55, 130.88 (q, *J* = 31.0 Hz), 125.26, 128.31 – 119.62 (m), 113.95, 110.19 – 109.76 (m), 109.09 – 108.73 (m), 52.35. HRMS (GC/EI+): *m/z* calculated 301.0379 for C_13_H_10_F_3_NO_2_S, found 301.0383 ([M]^•+^). qHNMR (400 MHz, DMSO-*d*_6_) purity = 97.7%.

#### *5-(3-Amino-5-(trifluoromethyl)phenyl)thiophene-2-carboxamide* (**76**)

Preparation according to general procedure A from **81** (149 mg, 0.52 mmol, 1.1 eq.) and 5-bromo-2-thiophenecarboxamide (**SM38**, 100 mg, 0.47 mmol, 1.0 eq.). Further purification by RP column chromatography yielded a colorless solid (84 mg, 63%). ^1^H NMR (400 MHz, DMSO-*d*_*6*_) δ 8.01 (s, 1H), 7.73 (d, *J* = 3.9 Hz, 1H), 7.51 (d, *J* = 3.9 Hz, 1H), 7.45 (s, 1H), 7.08 (s, 2H), 6.84 (s, 1H), 5.79 (s, 2H). ^13^C NMR (101 MHz, DMSO-*d*_*6*_) δ 162.66, 150.17, 146.75, 139.57, 134.74, 130.76 (q, *J* = 31.1 Hz), 129.74, 124.89, 124.25 (q, *J* = 272.4 Hz), 113.80, 109.79 – 109.23 (m), 108.93 – 108.39 (m). HRMS (GC/EI+): *m/z* calculated 286.0382 for C_12_H_9_F_3_N_2_OS, found 286.0384 ([M]^•+^). qHNMR (400 MHz, DMSO-*d*_6_) purity = 97.0%.

#### *N-(5-(3-Amino-5-(trifluoromethyl)phenyl)thiophen-2-yl)acetamide* (**77**)

Preparation according to general procedure A from **81** (160 mg, 0.56 mmol, 1.2 eq.) and N-(5-bromothiophen-2-yl)acetamide (**SM39**, 110 mg, 0.48 mmol, 1.0 eq.). Further purification by RP column chromatography yielded a colorless solid (62 mg, 43%). ^1^H NMR (400 MHz, DMSO-*d*_*6*_) δ 7.23 (d, *J* = 4.0 Hz, 1H), 7.01 – 6.98 (m, 1H), 6.97 – 6.94 (m, 1H), 6.72 – 6.67 (m, 1H), 6.60 (d, *J* = 4.0 Hz, 1H), 5.66 (s, 2H), 2.08 (s, 3H). ^13^C NMR (101 MHz, DMSO-*d*_*6*_) δ 166.46, 149.97, 139.92, 135.96, 132.11, 130.52 (q, *J* = 30.9 Hz), 124.42 (q, *J* = 271.0 Hz), 121.19, 112.71, 111.24, 107.71, 107.68, 22.53. HRMS (GC/EI+): *m/z* calculated 300.0539 for C_13_H_11_F_3_N_2_OS, found 300.0543 ([M]^•+^). qHNMR (400 MHz, DMSO-*d*_6_) purity = 96.9%.

#### *3-(5-Methylthiazol-2-yl)-5-(trifluoromethyl)aniline* (**78**)

Preparation according to general procedure A from **81** (160 mg, 0.56 mmol, 1.0 eq.) and 2-bromo-5-methylthiazole (**SM40**, 79 µL, 0.75 mmol, 1.5 eq.). Further purification by RP column chromatography yielded a colorless solid (38 mg, 29%). ^1^H NMR (400 MHz, MeOD-*d*_*4*_) δ 7.55 – 7.50 (m, 1H), 7.36 – 7.31 (m, 2H), 6.99 – 6.95 (m, 1H), 2.53 (d, *J* = 1.2 Hz, 3H). ^13^C NMR (126 MHz, MeOD-*d*_*4*_) δ 167.36, 149.88, 142.18, 136.66, 136.27, 133.36 (q, *J* = 32.0 Hz), 125.45 (q, *J* = 271.6 Hz), 116.51, 113.58 (q, *J* = 3.9 Hz), 112.52 (q, *J* = 4.0 Hz), 11.82. HRMS (GC/EI+): *m/z* calculated 258.0433 for C_11_H_9_F_3_N_2_S, found 258.0436 ([M]^•+^). qHNMR (400 MHz, DMSO-*d*_6_) purity = 96.9%.

#### *3-(4-Methylthiazol-2-yl)-5-(trifluoromethyl)aniline* (**79**)

Preparation according to general procedure A from **81** (144 mg, 0.5 mmol, 1.0 eq.) and 2-bromo-4-methylthiazole (**SM41**, 135 mg, 82 µL, 0.75 mmol, 1.5 eq.). Further purification by flash column chromatography yielded a colorless solid (82 mg, 64%). ^1^H NMR (400 MHz, Acetone-*d*_*6*_) δ 7.53 – 7.48 (m, 1H), 7.47 – 7.41 (m, 1H), 7.26 – 7.16 (m, 1H), 7.06 – 7.01 (m, 1H), 5.38 (s, 2H), 2.49 – 2.39 (m, 3H). ^13^C NMR (101 MHz, Acetone-*d*_*6*_) δ 166.73, 154.88, 150.93, 136.40, 133.09 – 131.98 (m), 129.54 – 121.08 (m), 115.51, 115.17, 112.40 – 111.97 (m), 111.28 – 110.82 (m), 17.25. HRMS (GC/EI+): *m/z* calculated 258.0433 for C_11_H_9_F_3_N_2_S, found 258.0435 ([M]^•+^). qHNMR (400 MHz, DMSO-*d*_6_) purity = 95.5%.

#### *3-(Thiazol-2-yl)-5-(trifluoromethyl)aniline* (**80**)

Preparation according to general procedure A from **81** (143 mg, 0.50 mmol, 1.0 eq.) and 2-bromothiazole (**SM42**, 68 µL, 0.75 mmol, 1.5 eq.). Further purification by RP column chromatography yielded a colorless solid (27 mg, 22%). ^1^H NMR (400 MHz, MeOD-*d*_*4*_) δ 7.87 (d, *J* = 3.3 Hz, 1H), 7.62 (d, *J* = 3.3 Hz, 1H), 7.45 – 7.38 (m, 2H), 7.01 – 6.98 (m, 1H). ^13^C NMR (126 MHz, MeOD-*d*_*4*_) δ 169.25, 151.24, 144.49, 136.05, 133.36 (q, *J* = 31.9 Hz), 125.53 (q, *J* = 271.6 Hz), 121.15, 116.15, 113.11 (q, *J* = 3.8 Hz), 111.88 (q, *J* = 4.1 Hz). HRMS (GC/EI+): *m/z* calculated 244.0277 for C_10_H_7_F_3_N_2_S, found 244.0277 ([M]^•+^). qHNMR (400 MHz, DMSO-*d*_6_) purity = 96.2%.

### In vitro characterization

#### Hybrid Reporter Gene Assays

Hybrid reporter gene assays. TLX modulation was determined in a Gal4 hybrid reporter gene assay in HEK293T cells (German Collection of Microorganisms and Cell Culture GmbH, DSMZ) using pFR-Luc (Stratagene, La Jolla, CA, USA; reporter), pRL-SV40 (Promega, Madison, WI, USA; internal control), pECE-SV40-Gal4-VP16^45^ (#71728, Addgene, Watertown, MA), and pFA-CMV-hTLX-LBD^36^, coding for the hinge region and ligand binding domain of the canonical isoform of human TLX. Cells were cultured in Dulbecco’s modified Eagle’s medium (DMEM), high glucose supplemented with 10% fetal calf serum (FCS), sodium pyruvate (1 mM), penicillin (100 U/mL), and streptomycin (100 μg/mL) at 37 °C and 5% CO2 and seeded in 96-well plates (3×104 cells/well). After 24 h, medium was changed to Opti-MEM without supplements and cells were transiently transfected using Lipofectamine LTX reagent (Invitrogen, Carlsbad, CA, USA) according to the manufacturer’s protocol. Five hours after transfection, cells were incubated with the test compounds in Opti-MEM supplemented with penicillin (100 U/mL), streptomycin (100 μg/mL) and 0.1% DMSO for 16 h before luciferase activity was measured using the Dual-Glo Luciferase Assay System (Promega) according to the manufacturer’s protocol on a Tecan Spark luminometer (Tecan Deutschland GmbH, Crailsheim, Germany). Firefly luminescence was divided by Renilla luminescence and multiplied by 1000 resulting in relative light units (RLU) to normalize for transfection efficiency and cell growth. Fold activation was obtained by dividing the mean RLU of test compound by the mean RLU of the untreated control. All samples were tested in at least three biologically independent experiments in duplicates. For dose-response curve fitting and calculation of EC_50_ values and remaining activity (= “bottom”), the equation “[Agonist] vs. response -- Variable slope (four parameters)” was used in GraphPad Prism (version 7.00, GraphPad Software, La Jolla, CA, USA). Selectivity profiling was performed with pFA-CMV-THRα-LBD^46^, pFA-CMV-RARα-LBD^47^, pFA-CMV-PPARγ-LBD^48^, pFA-CMV-RORα-LBD^49^, pFA-CMV-CAR-LBD^50^, pFA-CMV-PXR-LBD^50^, pFA-CMV-LXRα-LBD^50^, pFA-CMV-HNF4α-LBD^51^, pFA-CMV-hRXRα-LBD^52^, pFA-CMV-TR2-LBD^48^, pFA-CMV-PNR-LBD^53^, pFA-CMV-Nur77-LBD^54^, and pFA-CMV-SF1-LBD^55^. The following reference ligands were used: T3 (1 µM, THRα), Tretinoin (1 µM, RARα), Pioglitazone (1 µM, PPARγ), SR1001 (1 µM, revERBα and RORα), SR12813 (1 µM, PXR), CITCO (10 µM, CAR), T0901317 (1 µM, LXRα), Bexarotene (1 µM, RXRα), PR3 (1 µM, PNR) and compound 29 from Vietor et al.^56^ (0.3 µM, Nur77).

#### DR1 activity assay

The repression of DR1 activity by TLX was determined in a reporter gene assay in transiently transfected HEK293T cells using a firefly luciferase reporter for the human DR1 response element (#1015, Addgene, gift from Bruce Spiegelman)^57^ and pRL-SV40 (Promega) as internal control. To observe TLX effects on DR1 activity, a construct for constitutive expression (CMV promoter) of full-length human TLX^36^ was co-transfected. Cell culture, seeding, transient transfection, and test compound treatment were performed as described for hybrid reporter gene assays. Cells were treated with 0.1% DMSO as negative control, pioglitazone (1 µM) to induce DR1 activity, or with pioglitazone (1 µM) and a TLX modulator (**25, 27, 39** (10 µM each) or **72** (50 µM)). After 16 h incubation, luciferase activity was assayed as described for reporter gene assays, and firefly activity was normalized to renilla activity to obtain RLU. All samples were tested in at least six biologically independent experiments in duplicates.

#### Affinity Selection Mass Spectrometry (ASMS)

A mixture containing compounds **25, 27** and **39** (1 µM final concentration each) and ccrp2^10^ (1 µM) as reference, was incubated (at 4 °C for 1h) with TLX LBD protein (35 µM) in buffer (50 mM Tris, 200 mM NaCl, pH 7.4) containing 1% (v/v) DMSO in a total volume of 55 µL (N=9). Compounds **65** and **72** were tested at 10 µM for sufficient detection with 50 µM TLX LBD to maintain excess of the protein. In parallel, this procedure was performed for denatured (82 °C, 30 min) TLX LBD (35 µM or 50 µM) under otherwise identical conditions (N=3). After equilibration of the PD SpinTrap G-25 columns containing Sephadex G-25 medium (Cytiva, Little Chalfont, United Kingdom) according to the manufacturer’s instructions, 50 µL of each binding sample were transferred onto an SEC column followed by centrifugation (800 g, 2 min) to generate the eluates E1. In the same way the pure compound mixture (1 µM in buffer with 1% DMSO) without TLX incubation was subjected to SEC as further negative control (N=3). Next, aliquots of 40 µL of each E1 eluate were diluted to 400 µL with 0.1% HCOOH in H_2_O and 0.1% HCOOH in MeCN (90:10, v/v) containing 100 nM Terfenadine as internal standard for quantification by LC-MS. In the same way samples containing 10 nM test compounds and 100 nM Terfenadine, as well as blanks, were generated by dilution with 40 µL of pure buffer as LC-ESI-MS/MS control. Subsequently, the generated samples were sonicated for 5 min, vortexed and finally centrifuged (15.000 g, 5 min). After transfer of the samples to a 96 deep well plate LC-ESI-MS/MS was performed with an API5500 QTrap mass spectrometer (Sciex, Darmstadt, Germany) coupled to an Agilent 1260 infinity HPLC system (Agilent Technologies, Waldbronn, Germany) equipped with a SIL-20AHT autosampler (Shimadzu, Duisburg, Germany) and a rack changer II (Shimadzu, Duisburg, Germany) controlled by the Analyst software 1.6.3. For chromatography, a Triart C18 column (3 µm, 50 mm x 2 mm, YMC-Europe, protected with a 0.5 µm and 0.2 µm frit) as stationary phase in combination with 0.1 % (v/v) HCOOH in H_2_O (A) and 0.1 % (v/v) HCOOH in MeCN (B) as mobile phase under gradient elution (0-2 min: 90%A/10%B, 2-4 min: 90%A/10%B → 5%A/95%B, 4-6 min: 5%A/95%B, 6-7 min: 5%A/95%B → 90%A/10%B, 7-10 min: 90%A/10%B) was used at a flow rate of 400 µL/min, injecting 50 µL per sample. MS data acquisition was performed under positive ESI conditions in the MRM mode recording **25** at *m/z* 267 → 250, **27** at *m/z* 281 → 263, **39** at *m/z* 292 → 252, **65** at *m/z* 268 → 139, **72** at *m/z* 272 → 255 and Terfenadine at *m/z* 472 → 436 from 4.7 to 7.0 min. Quantification of the test compounds was based on area ratios of analyte vs internal standard.

#### Differential Scanning Fluorimetry (DSF)

2 µM of recombinant TLX LBD protein in buffer (15 mM HEPES pH 7.5, 150 mM NaCl) supplemented with SYPRO orange dye (1:1000 dilution) and compounds **25, 27, 39, 65** and **72** (300 µM, solubilized with DMSO at a final concentration of 2%) or DMSO (2%) were added to a white 96-well PCR plate to a final volume of 20 µL. The experiment was conducted on a QuantStudio 1 PCR system (Applied Biosystems, Waltham, CA, USA) heating from 25°C to 90°C with a heating rate of 0.015°C s^-1^. ccrp2^10^ (100 µM) served as reference ligand. Each sample was tested in at least three replicates. Amplification plots were analyzed by using a Boltzmann sigmoidal fit in GraphPad Prism (version 7.00, GraphPad Software, La Jolla, CA, USA) to obtain melting points (T_m_). Δ Tm corresponds to Δ Tm = Tm (TLX LBD with compound) − Tm (TLX LBD untreated). Statistical significance was evaluated with a one-way ANOVA with Dunnett’s multiple comparisons test in GraphPad Prism (version 7.00, GraphPad Software, La Jolla, CA, USA).

#### Determination of Thermodynamic Aqueous Solubility

The aqueous solubility of **25, 27, 39, 65** and **72** was assessed by mixing 0.5-1 mg of each test compound with an appropriate volume of water for a theoretical concentration of 2 mM to obtain an oversaturated mixture. The mixture was agitated in a VWR Thermal Shake lite (VWR International GmbH, Darmstadt, Germany) for 48 h at 600 rpm and a constant temperature of 25 °C. The supersaturated mixtures were subsequently centrifuged at 16.400 g for 15 min (25 °C). Part of the supernatant was taken off for quantification by ultraviolet (UV) absorbance at 254 nm (for **39, 65** and **72**) or 260 nm (for **25** and **27**) with external calibration. The external calibration samples contained 1% DMSO and the test samples were spiked with DMSO to 1% concentration right before the measurement. Absorbance was measured with a Tecan Spark luminometer using a Quartz 96-well plate (Tecan Deutschland GmbH, Crailsheim, Germany). The solubility test was repeated in three independent experiments.

#### Isothermal titration calorimetry (ITC)

ITC experiments were conducted on an Affinity ITC instrument (TA Instruments, New Castle, DE) at 25 °C with a stirring rate of 75 rpm. TLX LBD protein (60 µM) in buffer (20 mM Tris pH 7.5, 150 mM NaCl, mM TCEP, 5% glycerol) containing 3% DMSO was titrated with compound **39** (300 μM in the same buffer containing 3% DMSO) in 21 injections (1x 1 µL, 20x 4 μL) with an injection interval of 120 s. As control experiments, the compound was titrated to the buffer, and the buffer was titrated to the TLX LBD protein under otherwise identical conditions. The heats of the compound-TLX LBD titrations were corrected using the heats of the compound-buffer titrations. Results were analyzed using NanoAnalyze software (version 3.11.0, TA Instruments, New Castle, DE) with independent binding model.

## Supplementary Material

Supporting info.

## Figures and Tables

**Table 1:** Impact of modifications on the methylimidazole

ID	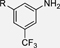 R=	EC_50_ (TLX)^a^	efficacy (remain. act.)^a^
**2**	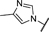	20±3 μM	0.44±0.04
**6**	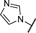	4.4±0.9 μM	0.28±0.05
**7**	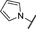	15±7 μM	0.2±0.2
**8**	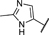	inactive (100 μM)	-
**9**	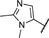	2.8±0.9	0.81±0.05
**10**	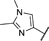	> 30 μM	0.70±0.06 at 100 μM
**11**	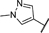	10±4 μM	0.4±0.1
**12**	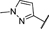	> 30 μM	0.74±0.09 at 100 μM
**13**	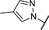	> 30 μM	0.48±0.09 at 100 μM
**14**	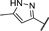	> 30 μM	0.29±0.05 at 100 μM
**15**	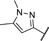	> 30 μM	0.6±0.1 at 100 μM
**16**	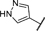	> 10 μM	0.15±0.02 at 100 μM
**17**	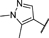	4±2 μM	0.5±0.1
**18**	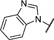	2.4±0.9 μM	0.27±0.09
**19**	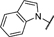	inactive (30 μM)	-
**20**	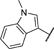	9.1±0.7 μM	0.20±0.05

a TLX modulation was determined in a hybrid reporter gene assay measuring the TLX-mediated repression of VP16-induced reporter activity (Figure S1). Remaining activity (remain. act.) refers to the reporter activity vs. DMSO (0.1%) control. Data are the mean±S.E.M., n≥3. All compounds were counterscreened to exclude non-specific effects on VP16 activity. Data for **2** from ^36^.

**Table 2:** Replacement of the methylimidazole

ID	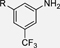 R=	EC_50_ (TLX)^a^	efficacy (fold act.)/ (remain. act.)^a^
**2**	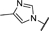	20±3 μM	0.44±0.04
**21**	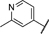	7±2 μM	0.28±0.07
**22**	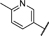	16±4 μM	0.37±0.07
**23**	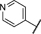	13±4 μM	0.2±0.1
**24**	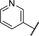	10±2 μM	0.35±0.06
**25**	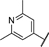	1.6±0.8 μM	0.31±0.08
**26**	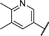	6±2 μM	0.24±0.09
**27**	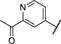	5±2 μM	0.30±0.05
**28**	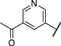	5±2 μM	0.25±0.09
**29**	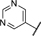	2.1±0.5 μM	0.40±0.06
**30**	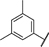	inverse agonist >30 μM	2.2±0.2 at 50 μM

a TLX modulation was determined in a hybrid reporter gene assay measuring the TLX-mediated repression of VP16-induced reporter activity (Figure S1). Maximum fold reporter activation (fold act.) or remaining activity (remain. act.) refer to the reporter activity vs. DMSO (0.1%) control. Data are the mean±S.E.M., n≥3. All compounds were counterscreened to exclude non-specific effects on VP16 activity. Data for **2** from ^36^.

**Table 3:** SAR of bicyclic motifs

ID	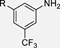 R=	EC_50_ (TLX)^a^	efficacy (remain. act.)^a^
**2**	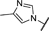	20±3 μM	0.44±0.04
**18**	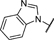	2.4±0.9 μM	0.27±0.09
**20**	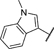	9.1±0.7 μM	0.19±0.05
**31**	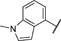	18±1 μM	0.2±0.1
**32**	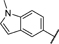	1.1±0.1 μM	0.44±0.03
**33**	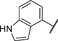	3.0±0.2 μM	0.18±0.05
**34**	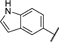	1.4±0.4 μM	0.27±0.07
**35**	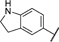	2.3±0.7 μM	0.27±0.07
**36**	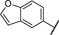	Toxic ≥30 μM	0.84±0.06 at 10 μM
**37**	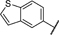	inactive (10 μM)	-
**38**	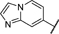	3.7±0.6 μM	0.32±0.04
**39**	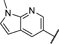	0.7±0.2 μM	0.27±0.06
**40**	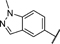	9.1±0.7 μM	0.29±0.06
**41**	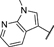	3.5±0.8 μM	0.23±0.08

a TLX modulation was determined in a hybrid reporter gene assay measuring the TLX-mediated repression of VP16-induced reporter activity (Figure S1). Remaining activity (remain. act.) refers to the reporter activity vs. DMSO (0.1%) control. Data are the mean±S.E.M., n≥3. All compounds were counterscreened to exclude non-specific effects on VP16 activity. Data for **2** from ^36^.

**Table 4:** SAR of the trifluoromethylaniline core.

ID	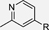 R=	EC_50_ (TLX)^a^	efficacy (remain. act.)^a^
**21**	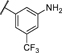	7±2 μM	0.28±0.07
**42**	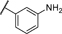	0.7±0.3 μM	0.76±0.03
**43**	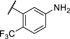	> 30 μM	0.39±0.04 at 50 μM
**44**	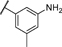	> 30 μM	0.44±0.09 at 30 μM
**45**	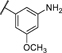	15±4 μM	0.56±0.05
**46**	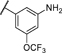	13.6±0.6 μM	0.24±0.03
**47**	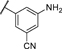	6±2 μM	0.4±0.1
**48**	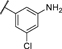	10±7 μM	0.44±0.08
**49**	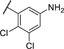	9.6±0.7 μM	0.31±0.04
**50**	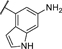	3.8±0.7 μM	0.26±0.06
**51**	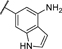	inactive (50 μM)	-
**52**	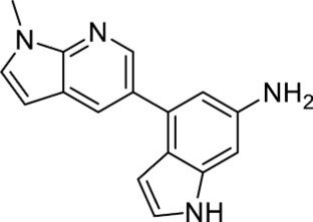	1.4±0.3 μM	0.50±0.03

a TLX modulation was determined in a hybrid reporter gene assay measuring the TLX-mediated repression of VP16-induced reporter activity (Figure S1). Remaining activity (remain. act.) refers to the reporter activity vs. DMSO (0.1%) control. Data are the mean±S.E.M., n≥3. All compounds were counterscreened to exclude non-specific effects on VP16 activity.

**Table 5:** Preliminary SAR of inverse TLX agonists

ID	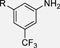 R=	TLX modulation IC_50_/EC_50_^a^	efficacy (fold act.)/ (remain. act.)^a^
**30**	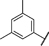	inverse agonist IC_50_ >30 μM	2.2±0.2 at 50 μM
**53**	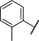	inverse agonist IC_50_ 25±3 μM	1.9±0.1
**54**	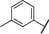	inverse agonist IC_50_ 16±3 μM	2.5±0.1
**55**	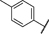	inverse agonist IC_50_ > 30 μM	2.0±0.5 at 30 μM
**56**	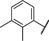	inverse agonist IC_50_ 10±2 μM	2.0±0.2
**57**	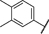	inverse agonist IC_50_ >30 μM	2.2±0.5 at 30 μM
**58**	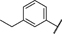	inverse agonist IC_50_ >30 μM	2.1±0.5 at 50 μM
**59**	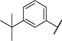	inactive (10 μM)	-
**60**	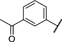	inactive (10 μM)	-
**61**	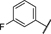	inverse agonist IC_50_ 19±3 μM	1.66±0.09
**62**	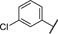	inverse agonist IC_50_ > 30 μM	1.8±0.4 at 30 μM
**63**	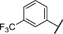	inverse agonist IC_50_ 32±2 μM	2.3±0.1
**64**	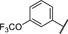	inverse agonist IC_50_ 39±7 μM	1.7±0.1
**65**	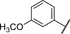	inverse agonist IC_50_ 2.9±0.8 μM	1.81±0.07
**66**	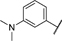	inverse agonist IC_50_ 2.9±0.9 μM	2.3±0.1
**67**	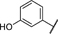	agonist EC50 6±1 μM	0.32±0.08

a TLX modulation was determined in a hybrid reporter gene assay measuring the TLX-mediated repression of VP16-induced reporter activity (Figure S1). Maximum fold reporter activation (fold act.) or remaining activity (remain. act.) refer to the reporter activity vs. DMSO (0.1%) control. Data are the mean±S.E.M., n≥3. All compounds were counterscreened to exclude non-specific effects on VP16 activity.

**Table 6:** Hetero- and bicyclic motifs in inverse agonists

ID	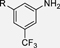 R=	TLX modulation IC_50_/EC_50_^a^	efficacy (fold act.)/ (remain. act.)^a^
**68**	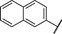	inverse agonist IC_50_ 12±1 μM	3.1±0.1
**69**	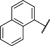	inactive (10 μM)	-
**70**	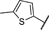	inverse agonist IC_50_ 15±5 μM	3.1±0.4
**71**	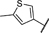	inverse agonist IC_50_ 12±3 μM	3.0±0.2
**72**	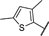	inverse agonist IC_50_ 16±5 μM	3.1±0.4
**73**	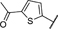	agonist EC_50_ 4±2 μM	0.27±0.07
**74**	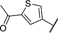	agonist EC_50_ 1.5±0.5 μM	0.19±0.06
**75**	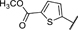	agonist EC_50_ 4±2 μM	0.41±0.09
**76**	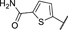	agonist EC_50_ 2.8±0.5 μm	0.44±0.04
**77**	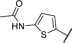	agonist EC_50_ 5±1 μM	0.52±0.04
**78**	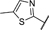	agonist EC_50_ 33±9 μM	0.5±0.2
**79**	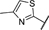	inactive (30 μM)	-
**80**	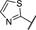	agonist	0.6±0.1 at 100 μM

a TLX modulation was determined in a hybrid reporter gene assay measuring the TLX-mediated repression of VP16-induced reporter activity (Figure S1). Maximum fold reporter activation (fold act.) or remaining activity (remain. act.) refer to the reporter activity vs. DMSO (0.1%) control. Data are the mean±S.E.M., n≥3. All compounds were counterscreened to exclude non-specific effects on VP16 activity.

**Table 7:** Features of the most favored fragment TLX agonists (25, 27, 39) and inverse agonists (65, 72)

	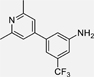	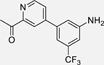	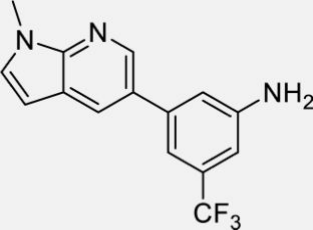	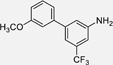	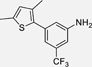
	25	27	39	65	72
TLX^a^	EC_50_ 1.6±0.8 μM (0.31±0.08 rem.)	EC_50_ 5±2 μM (0.30±0.05 rem.)	EC_50_ 0.7±0.2 μM (0.27±0.06 rem.)	IC_50_ 2.9±0.8 μM (1.81±0.07 fold)	IC_50_ 16±5 μM (3.1±0.4 fold)
DSF ^b^	ΔT_m_ -(1.6±0.4)°C	ΔT_m_ -(1.2±0.4)°C	ΔT_m_ -(2.1±0.4)°C	ΔT_m_ 5±1°C	ΔT_m_ 4±1°C
ITC^c^	n.d.	n.d.	K_d_ 1.1±0.1	n.d.	n.d.
logP^d^	3.6	2.8	3.3	3.9	4.3
LE^e^	0.42	0.37	0.40	0.40	0.37
LLE^e^	2.18	2.53	2.81	1.60	0.54
aq. sol. [mg/L]^f^	68±16	15.7±0.4	12±1	39.3±0.6	12±2

a From Gal4-TLX hybrid assay (Figures S1; dose-response curves in Figure S2).

b Difference of the melting points T_m_ between compound-treated TLX LBD (2 µM) and TLX LBD (2 µM) in the DMSO control.

c K_d_ value (mean±SD, n=2) from isothermal titration calorimetry (ITC). Data in Figure 2c,d. n.d. – not determined.

d LogP was calculated with the AlogPS^37^ resource. ^e^ Ligand efficiency (LE) and lipophilic ligand efficiency (LLE) were calculated according to ref. ^38^.

f Thermodynamic aqueous solubility was determined by UV-metric quantification from the supernatant of oversaturated compound solutions, n = 3.

## Abbreviations Used

ASMS: affinity-selection-mass-spectrometry
DSF: differential scanning fluorimetry
ITC: isothermal titration calorimetry
LBD: ligand binding domain
LE: ligand efficiency
LLE: lipophilic ligand efficiency
LSD1: lysine-specific demethylase 1
NP: neural progenitors
NR: nuclear receptor
NSC: neural stem cells
pten: phosphatase and tensin homologue
ROR: retinoic acid receptor-related orphan receptor
RXR: retinoic X receptor
SEC: size-exclusion chromatography
TLX: tailless homologue.
