# Supplementary material for: Systematic optimization of fragment TLX ligands towards agonism and inverse agonism
Source: J Med Chem. Author manuscript; Available in PMC 2026 Mar 5. (PMC7618817; doi:10.1021/acs.jmedchem.5c02718)
Supplement: Supporting info. [file EMS212277-supplement-Supporting_info_.pdf]

- Supporting Information -

Systematic optimization of fragment TLX ligands towards agonism and inverse agonism

Emily C. Hank[1], Loris Knümann[1], Úrsula López-García[1], Arthur Kardanov[1], Vasily Morozov[1], Georg Höfner[1], Daniel Merk[1]*

[1] Ludwig-Maximilians-Universität (LMU) München, Department of Pharmacy, 81377 Munich, Germany
* daniel.merk@cup.lmu.de

Table of Contents

Supplementary Figures and Tables S2
NMR spectra (qH NMR) of **6-80** S6
Supplementary References S44

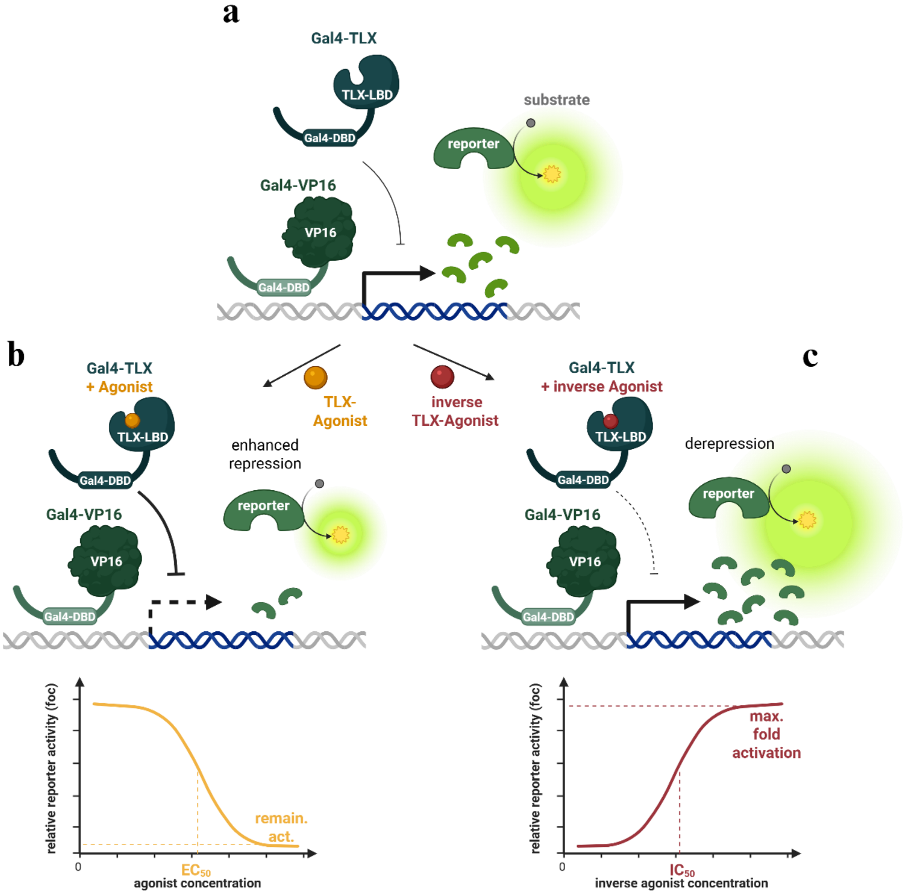

Figure S1. Schematic representation of TLX agonist and inverse agonist responses in the Gal4-TLX hybrid reporter gene assay. (a) The transcriptional activator Gal4-VP16 induces constitutive expression of the firefly luciferase reporter at a Gal4 response element. Gal4-TLX acts as constitutive repressor of the Gal4-VP16 mediated reporter gene expression. (b) A bound TLX agonists enhances TLX constitutive repressor activity and thereby reduces the relative reporter activity (as fold over the DMSO control). Efficacy of the TLX activation is described by the remaining reporter activity (remain. act.) at the bottom of the dose-response curve. (c) A bound TLX inverse agonists derepresses the reporter gene expression and increases the relative reporter activity (as fold over the DMSO control). Efficacy of the TLX inhibition is described by the max. fold reporter activation (fold act.) at the top of the dose-response curve. Figure created with BioRender.com and adapted with permission from [1]. Copyright 2024 Hank et al. Published by the American Chemical Society.

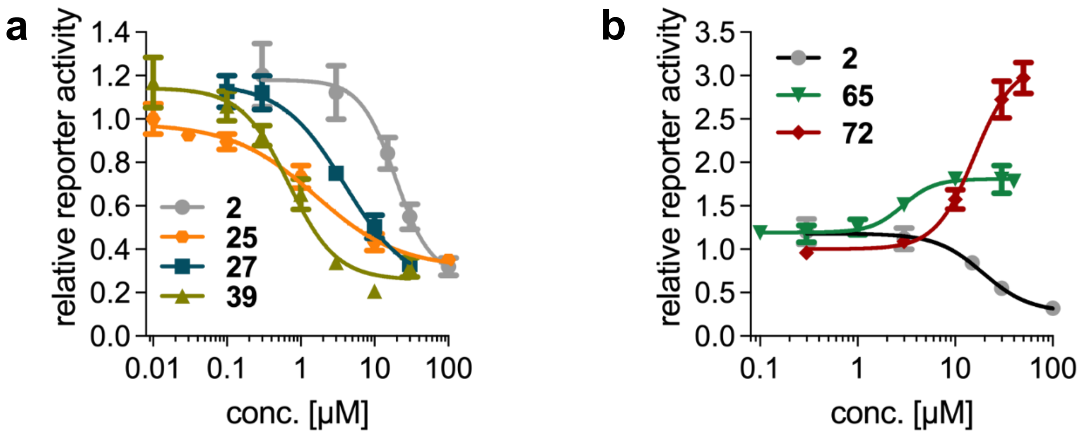

Figure S2. Dose-response curves of compounds 25, 27, 39, 65 and 72 in the Gal4-TLX hybrid reporter gene assay. (a) Fragment agonists **25**, **27** and **39** show comparable efficacy (remaining reporter activity), but improved potency over the lead fragment **2**. (b) Inverse agonist fragments **65** and **72** revert the activity profile of the lead fragment **2** to inverse agonism while displaying similar (**72**) or improved (**65**) potency. Relative reporter activity as fold over the DMSO-control (0.1% DMSO: relative reporter activity = 1). Data are the mean±S.E.M., $n \geq 3$. Data for **2** from the literature[2].

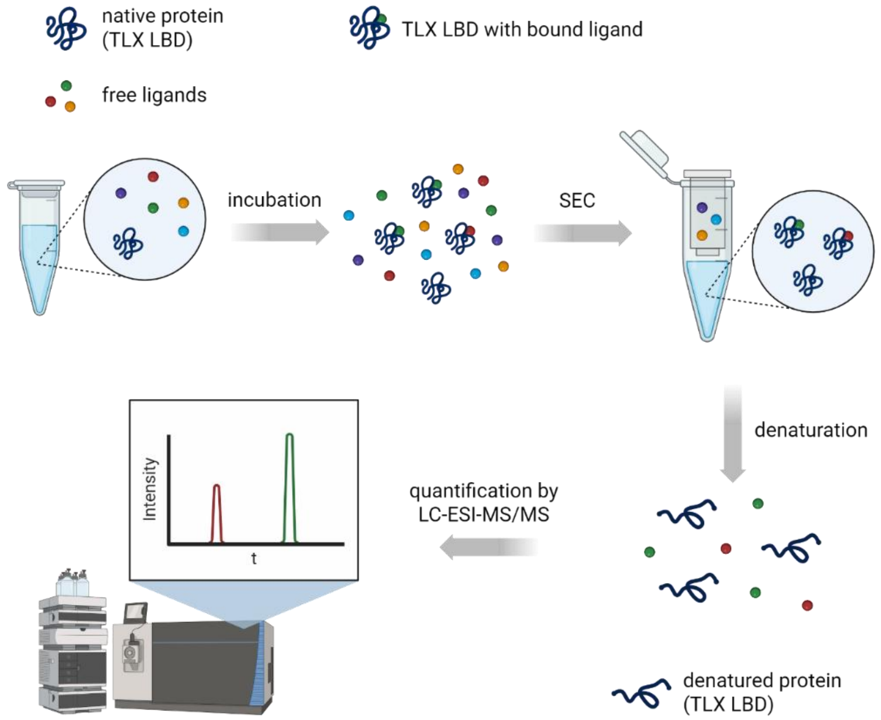

Figure S3. Schematic illustration of the affinity-selection-mass-spectrometry (ASMS) assay. A mixture of ligands is incubated with native TLX LBD protein. Size-exclusion-chromatography (SEC) separates protein and protein-ligand complexes from unbound ligands. The proteins in the eluate are denatured to release bound ligands which are analyzed and quantified by LC-ESI-MS/MS. The protocol is repeated with previously denatured protein to counterscreen for unspecific binding. Figure created with BioRender.com and adapted with permission from [3]. Copyright 2025 Nawa et al. Published by the American Chemical Society.

Table S1. Selectivity data for compounds 25, 39 and 65 against other members of the nuclear receptor family. Relative reporter activity as fold over the DMSO-control (0.1% DMSO: relative reporter activity = 1). Data are the mean±S.E.M., *n*=3.

	Reporter activity (0.1% DMSO = 1)			
	**25** (30 µM)	**39** (30 µM)	**65** (30 µM)	**positive control**
**THRα**	1.8±1.0	0.8±0.4	1.4±0.4	697±304<br>1 µM T3
**RARα**	1.0±0.2	1.3±0.3	1.9±0.5	270±64<br>1 µM tretinoin
**PPARγ**	2.1±0.4	3.6±0.4	3.9±0.8	26±3<br>1 µM pioglitazone
**revERBα**	2.1±0.2	2.3±0.6	2.6±0.6	0.62±0.12<br>1 µM SR1001
**RORα**	0.34±0.06	0.11±0.02	0.95±0.11	0.65±0.06<br>1 µM SR1001
**PXR**	1.4±0.3	2.7±0.7	3±1	2.0±0.2<br>1 µM SR12813
**CAR**	1.6±0.1	2.0±0.3	2.1±0.2	17.8±0.6<br>10 µM CITCO
**LXRα**	2.0±0.5	1.8±0.2	2.0±0.1	57±10<br>1 µM T0901317
**HNF4α**	0.6±0.1	0.4±0.2	1.5±0.3	no ref. ligand
**RXRα**	4.1±0.5	1.04±0.04	78±3	90±8<br>1 µM bexarotene
**TR2**	0.63±0.05	0.38±0.03	0.94±0.07	no ref. ligand
**PNR**	0.7±0.2	0.6±0.1	1.56±0.03	1.4±0.6<br>1 µM PR3
**Nur77**	0.75±0.06	0.47±0.02	1.6±0.2	7.5±0.6<br>0.3 µM compound 29 from Vietor et al.[4]
**SF1**	0.53±0.06	0.28±0.07	1.8±0.2	no ref. ligand

NMR spectra (qH NMR) of **6-80**:

Compound **6**:

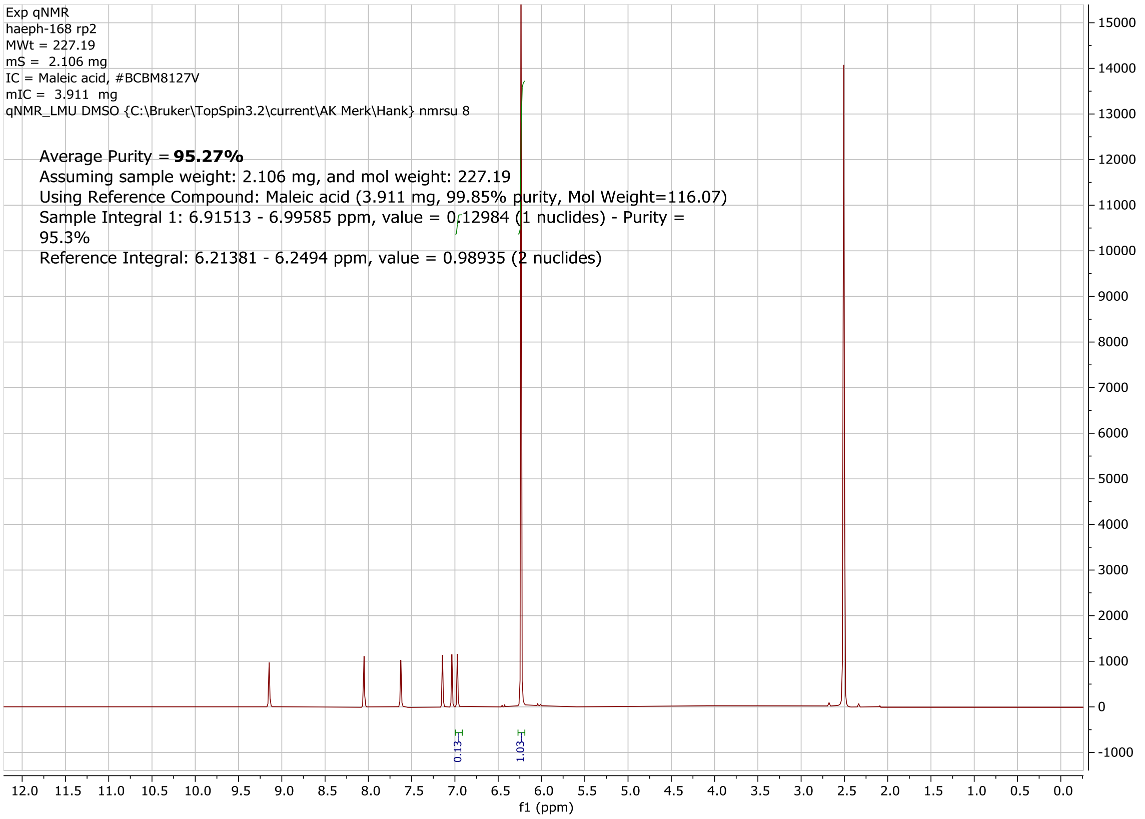

Compound **7**:

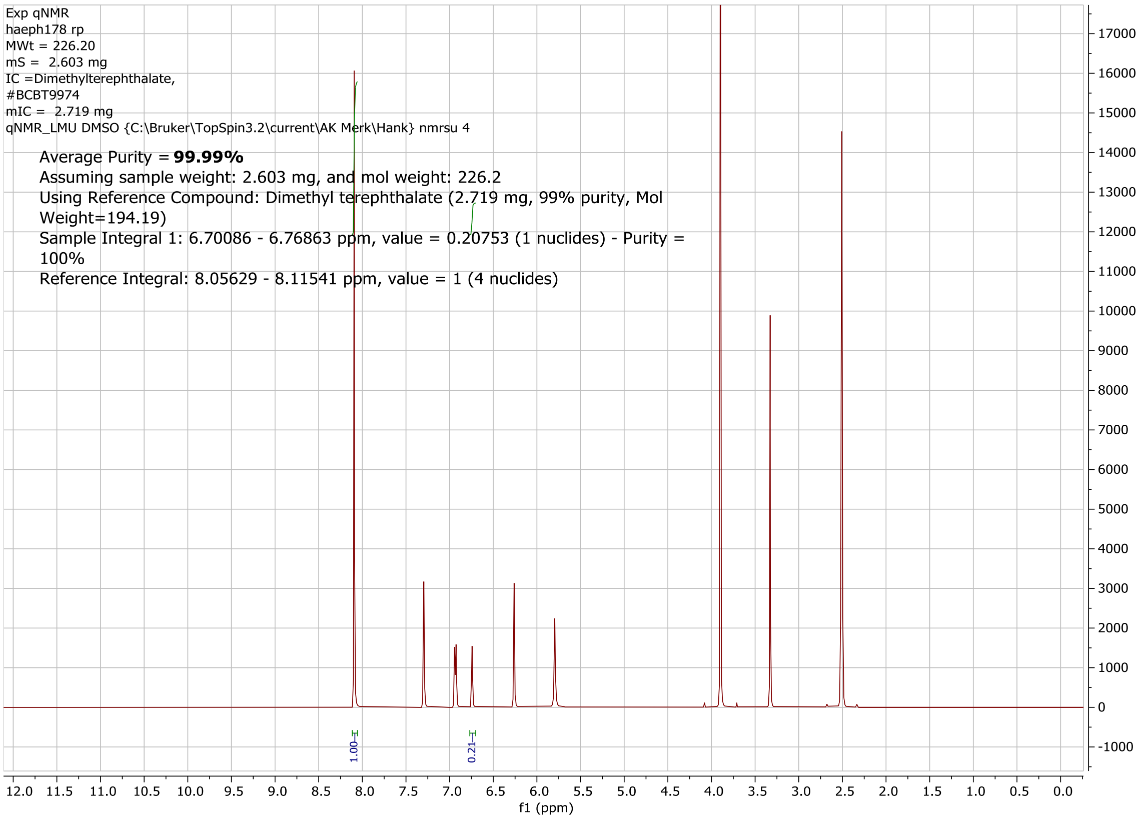

Compound **8**:

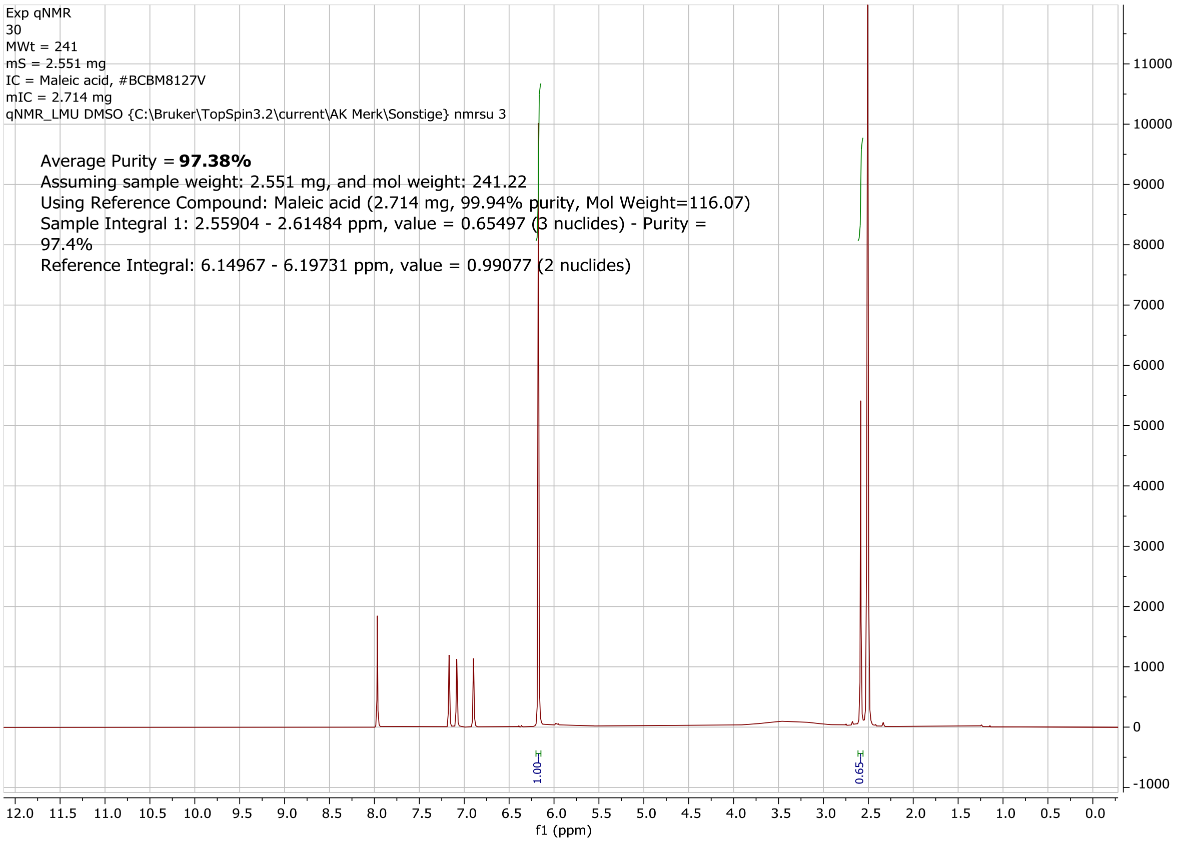

Compound **9**:

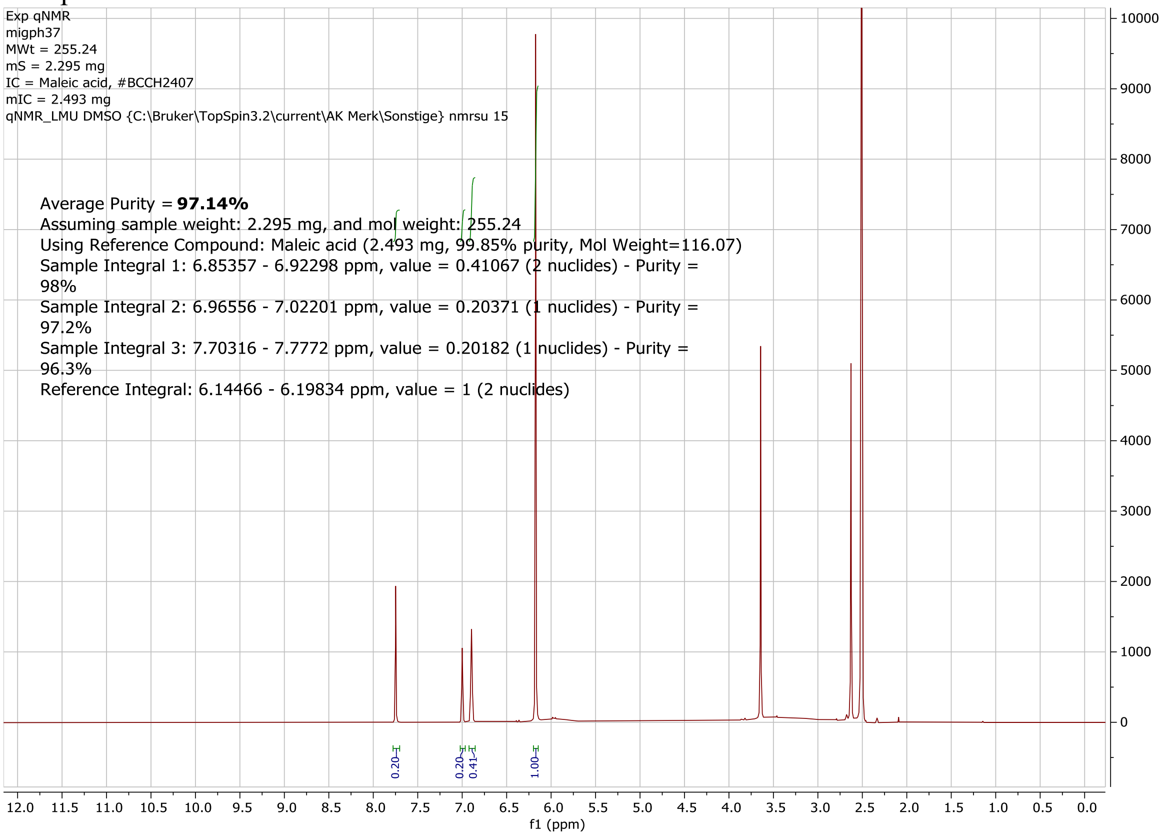

Compound **10**:

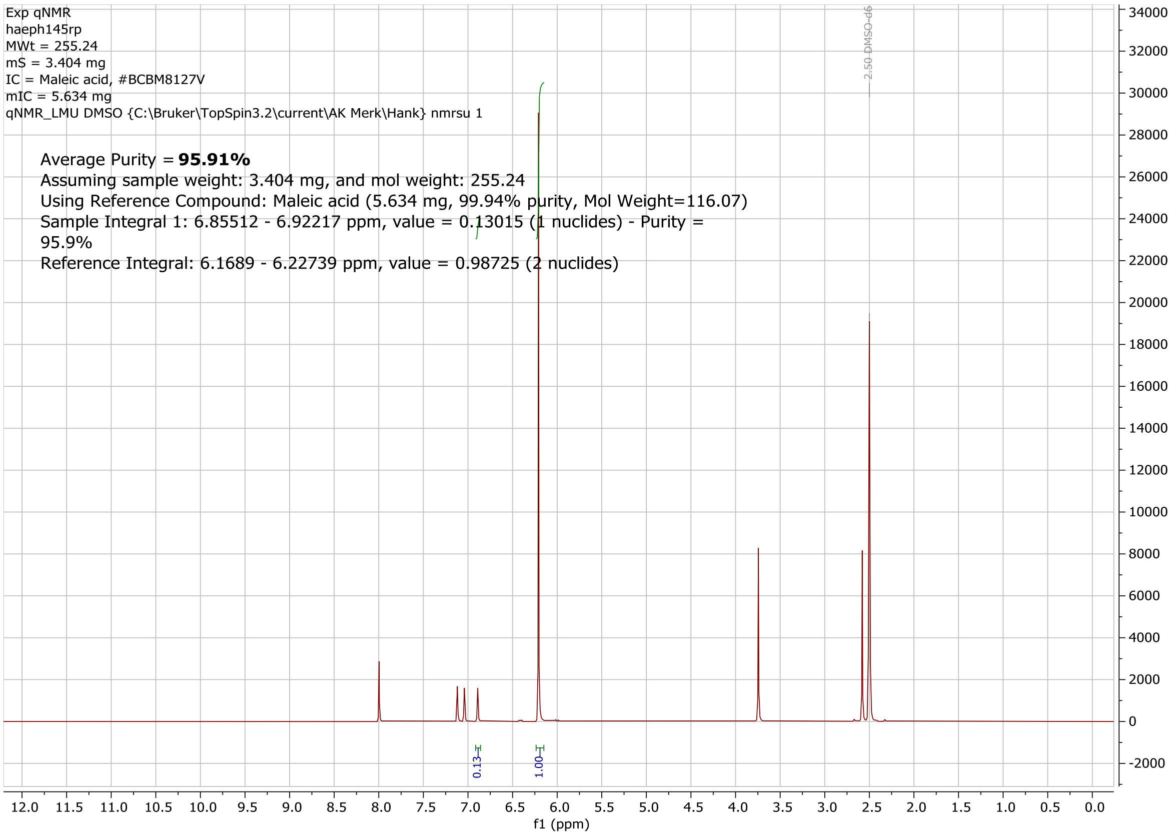

Compound **11**:

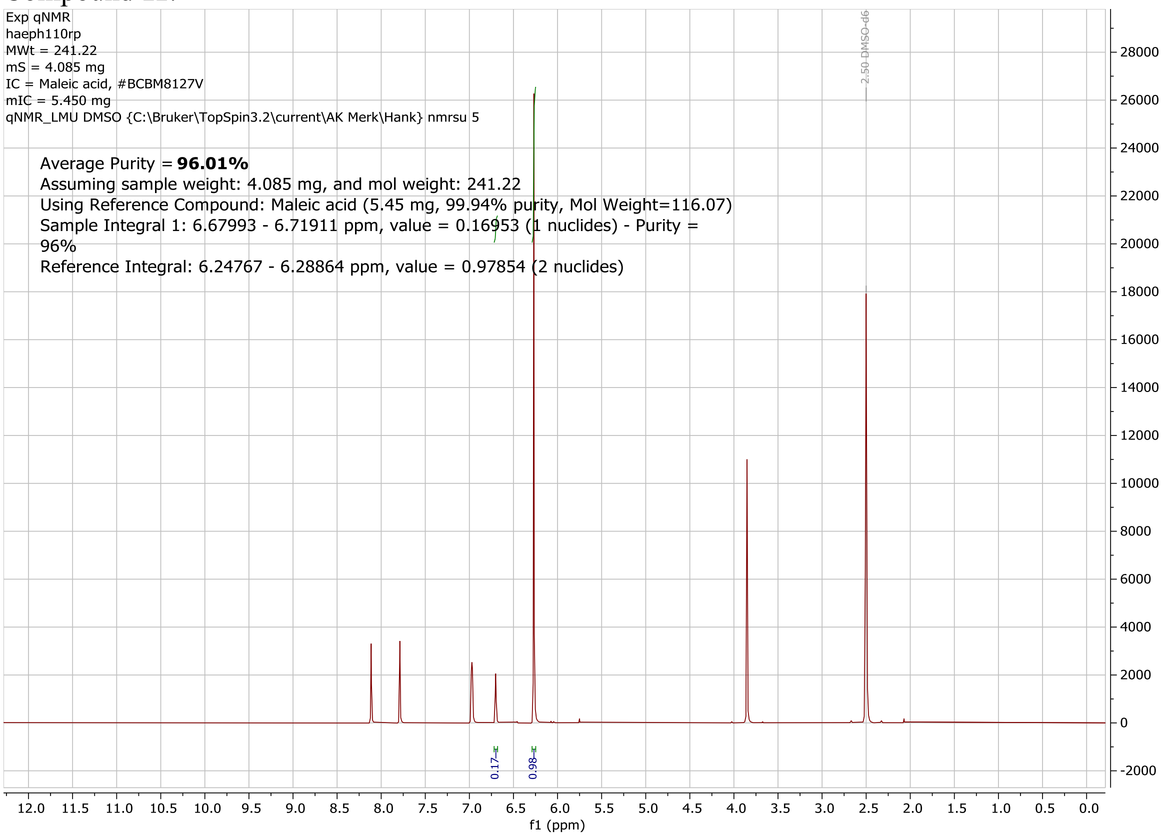

Compound **12**:

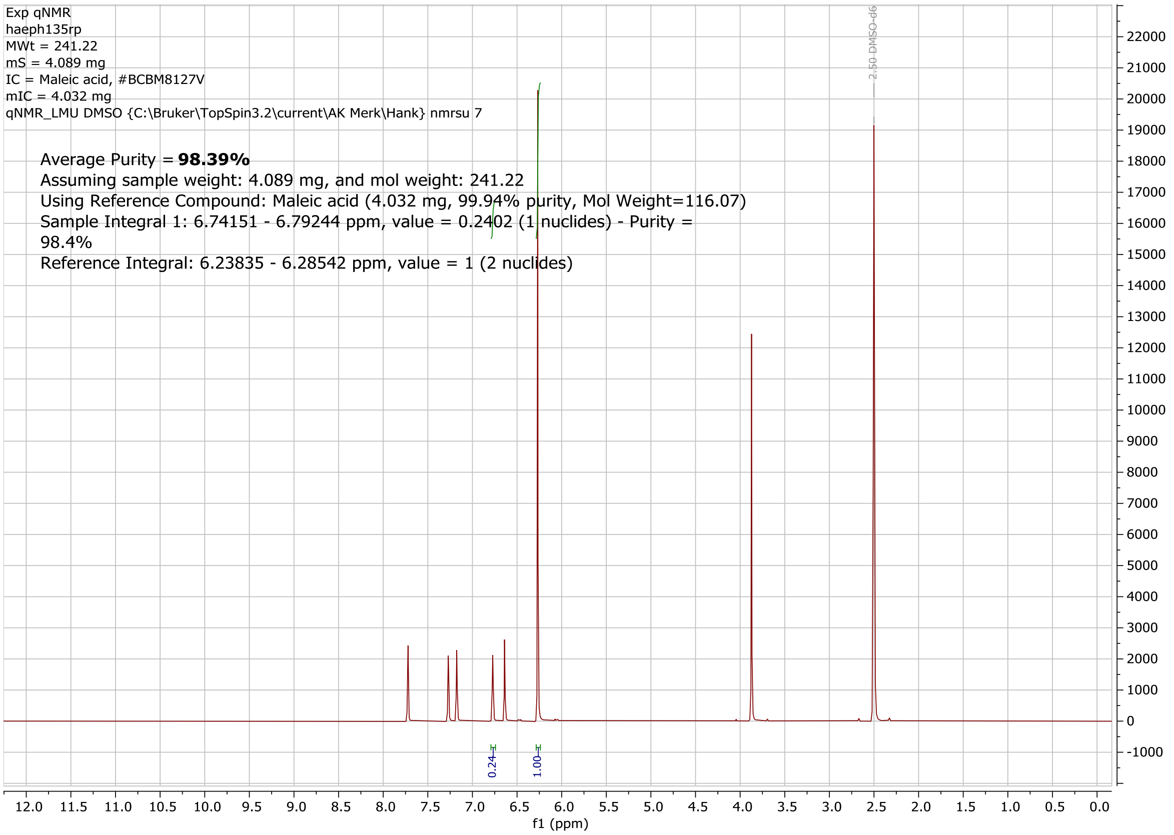

Compound **13**:

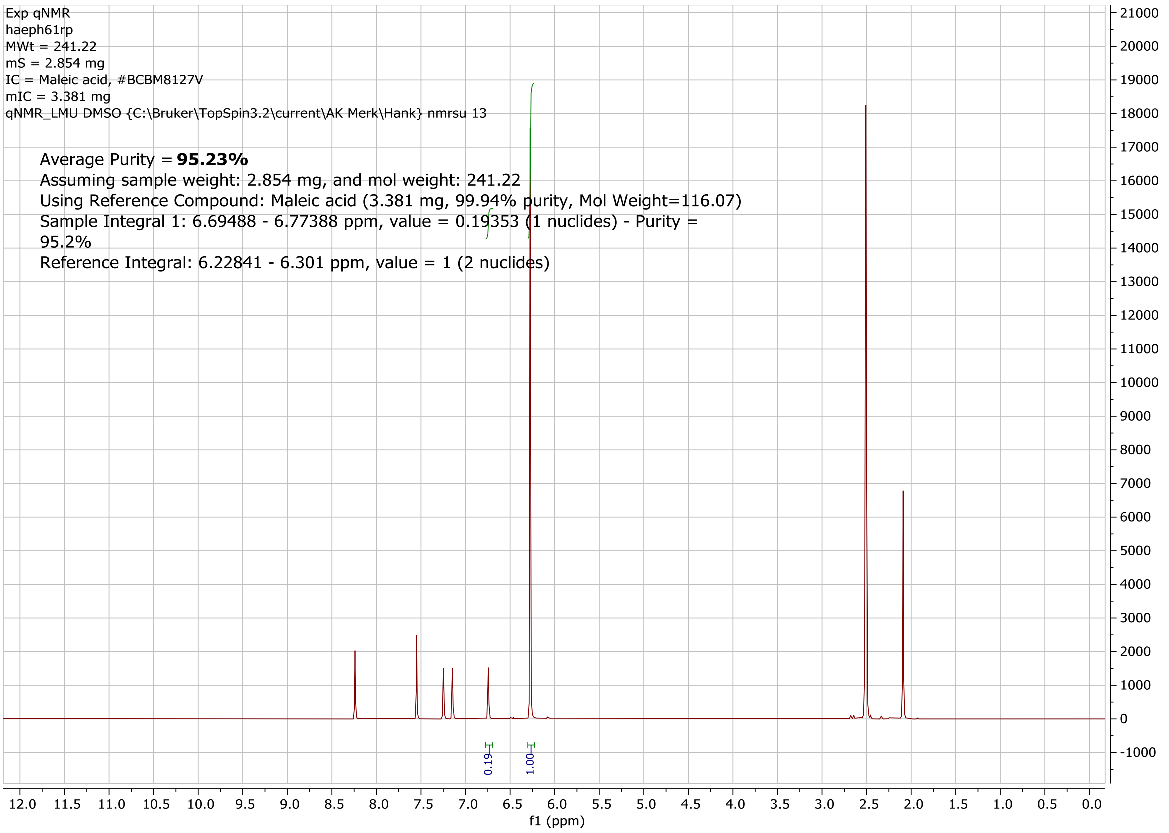

Compound **14**:

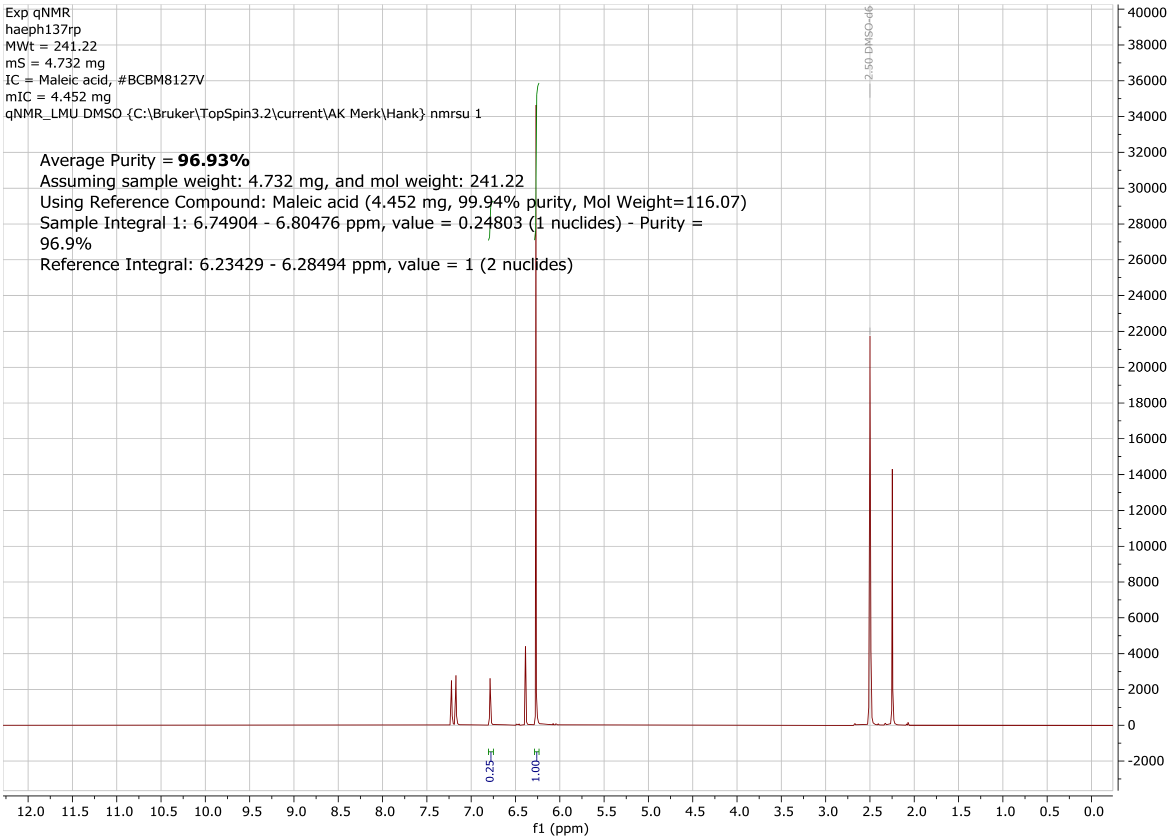

Compound **15**:

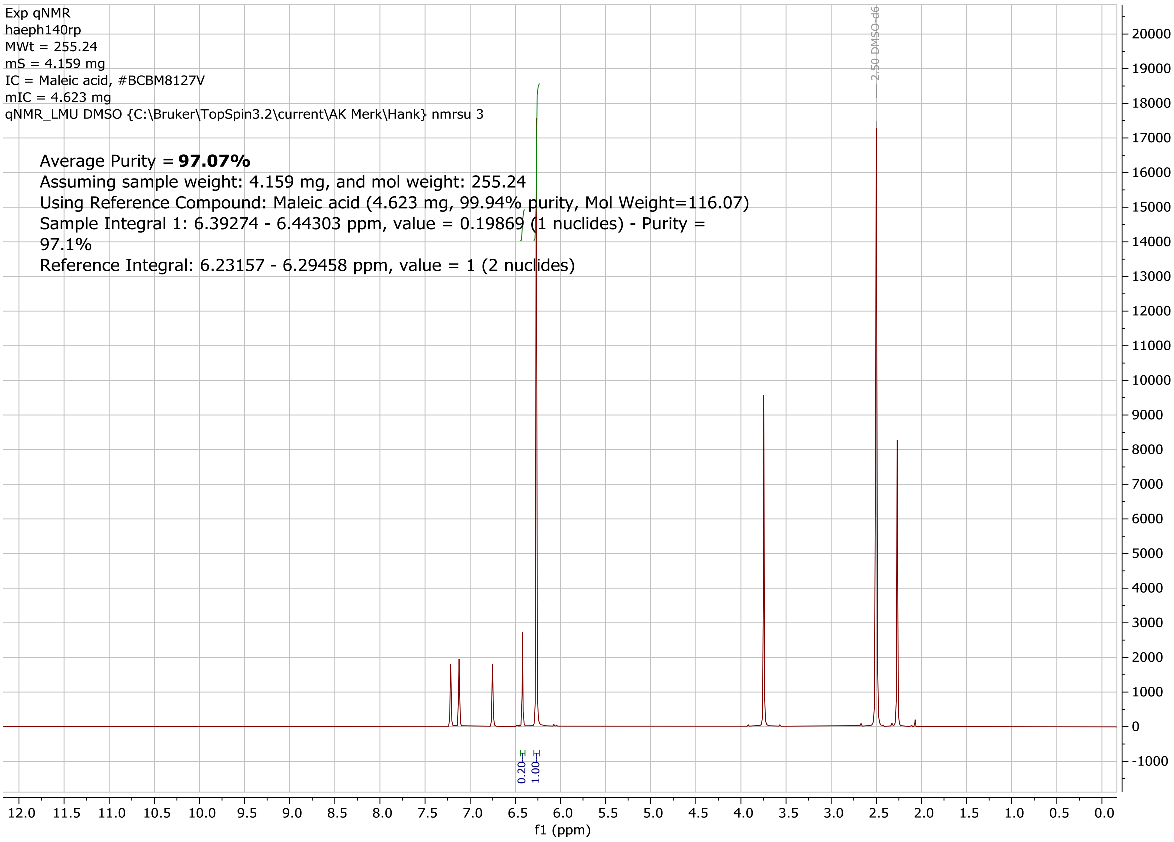

Compound **16**:

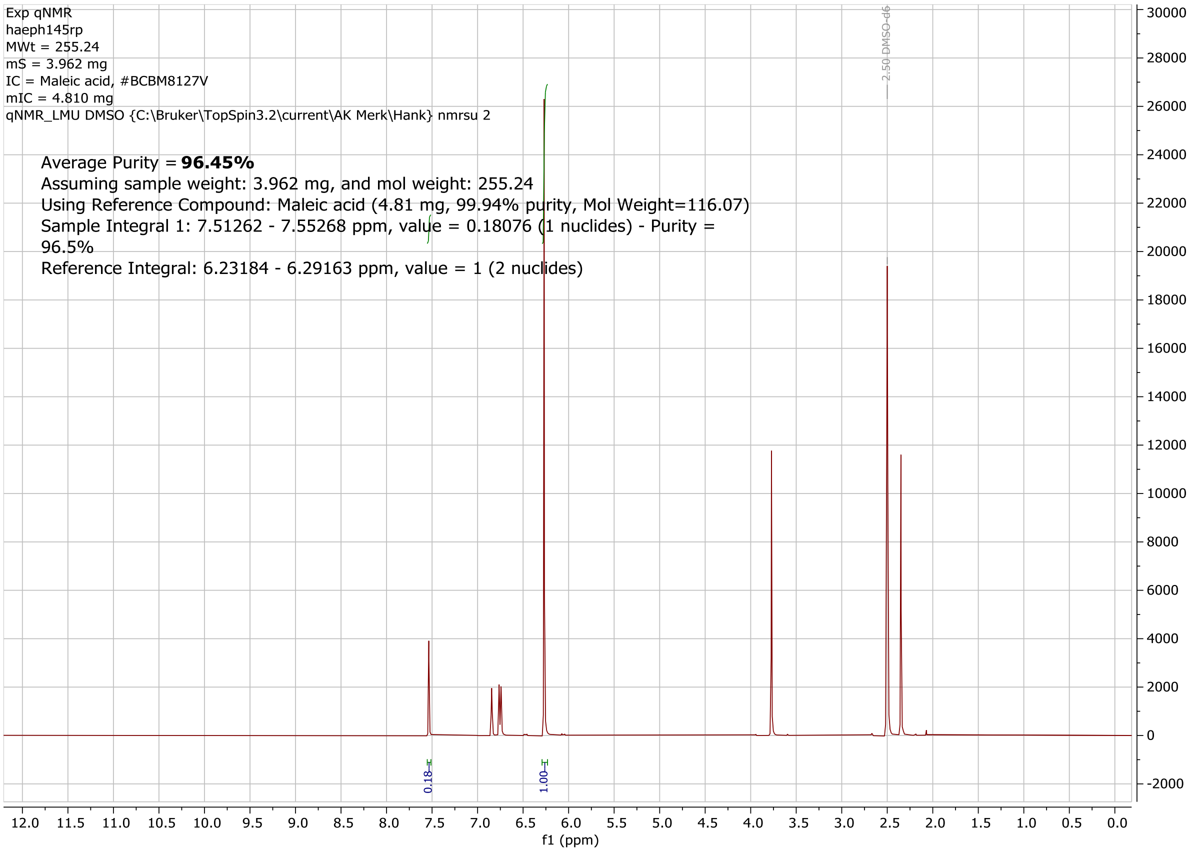

Compound **17**:

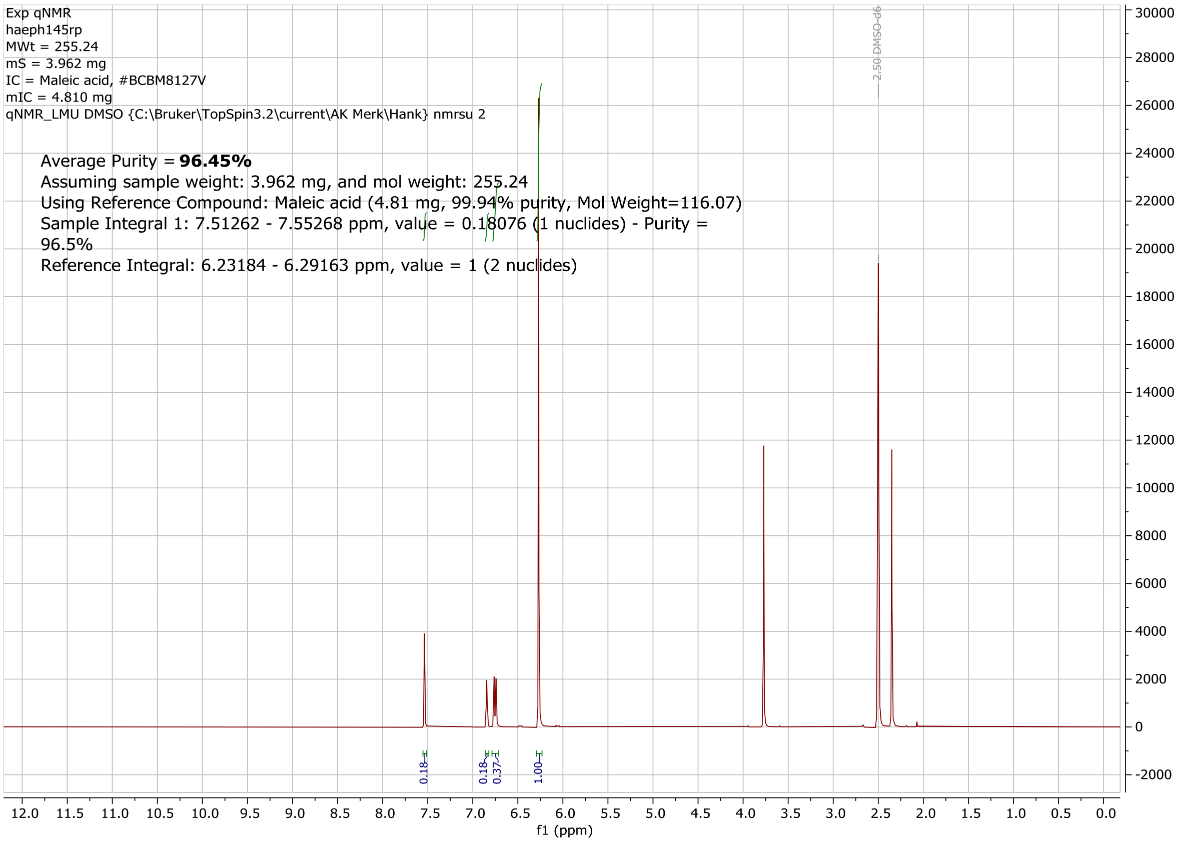

Compound **18**:

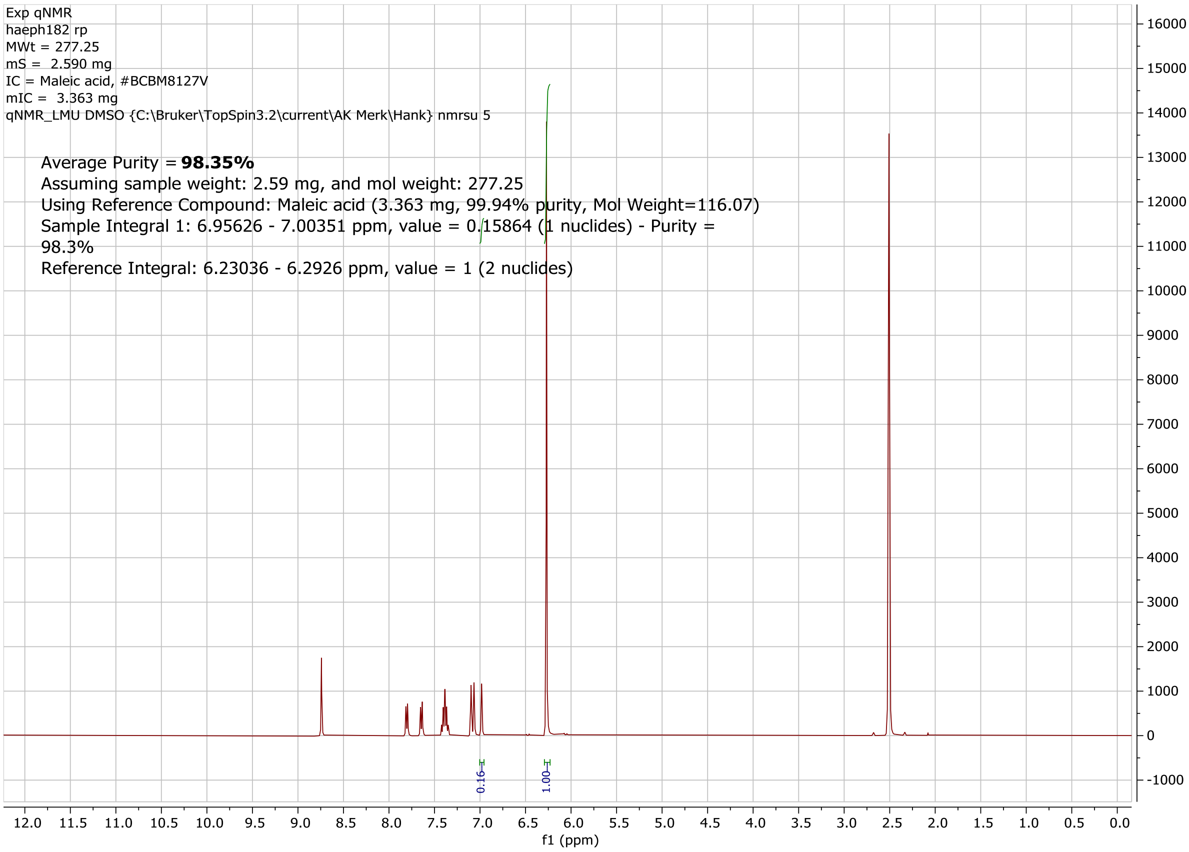

Compound **19**:

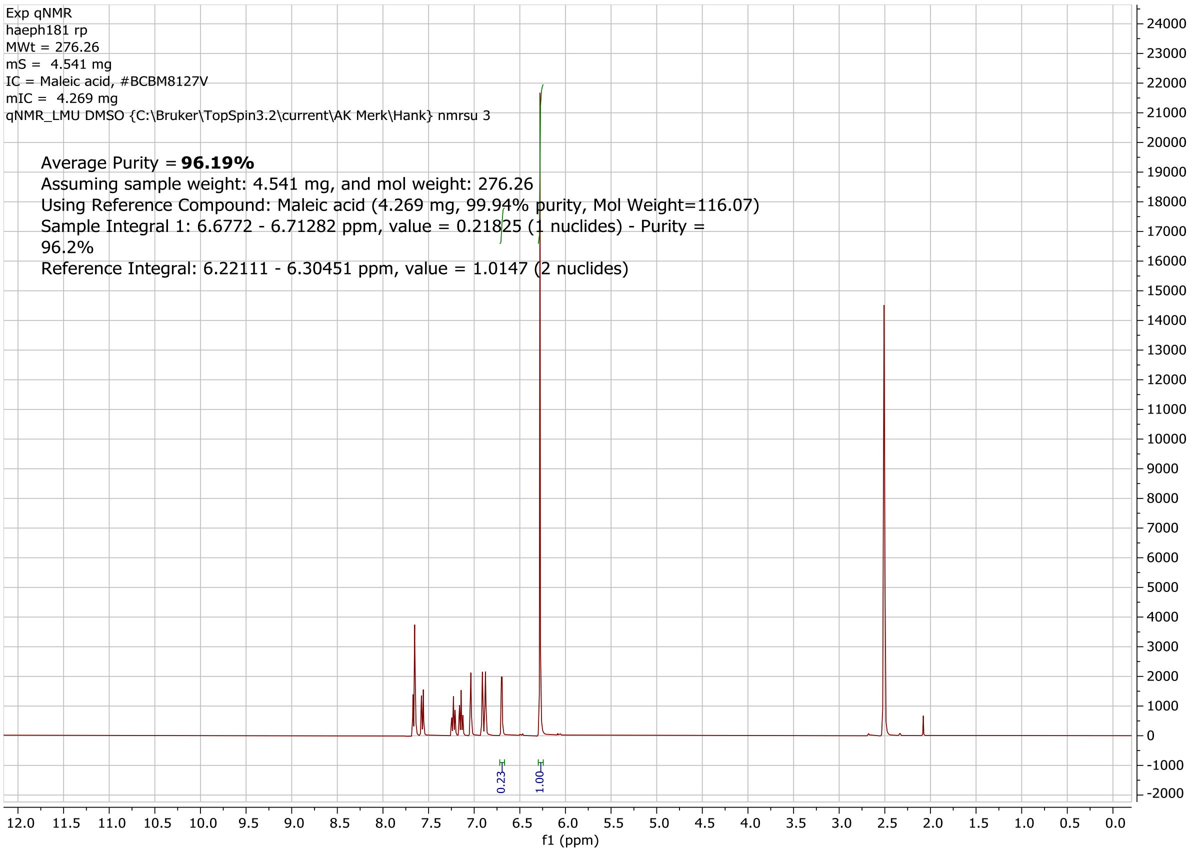

Compound **20**:

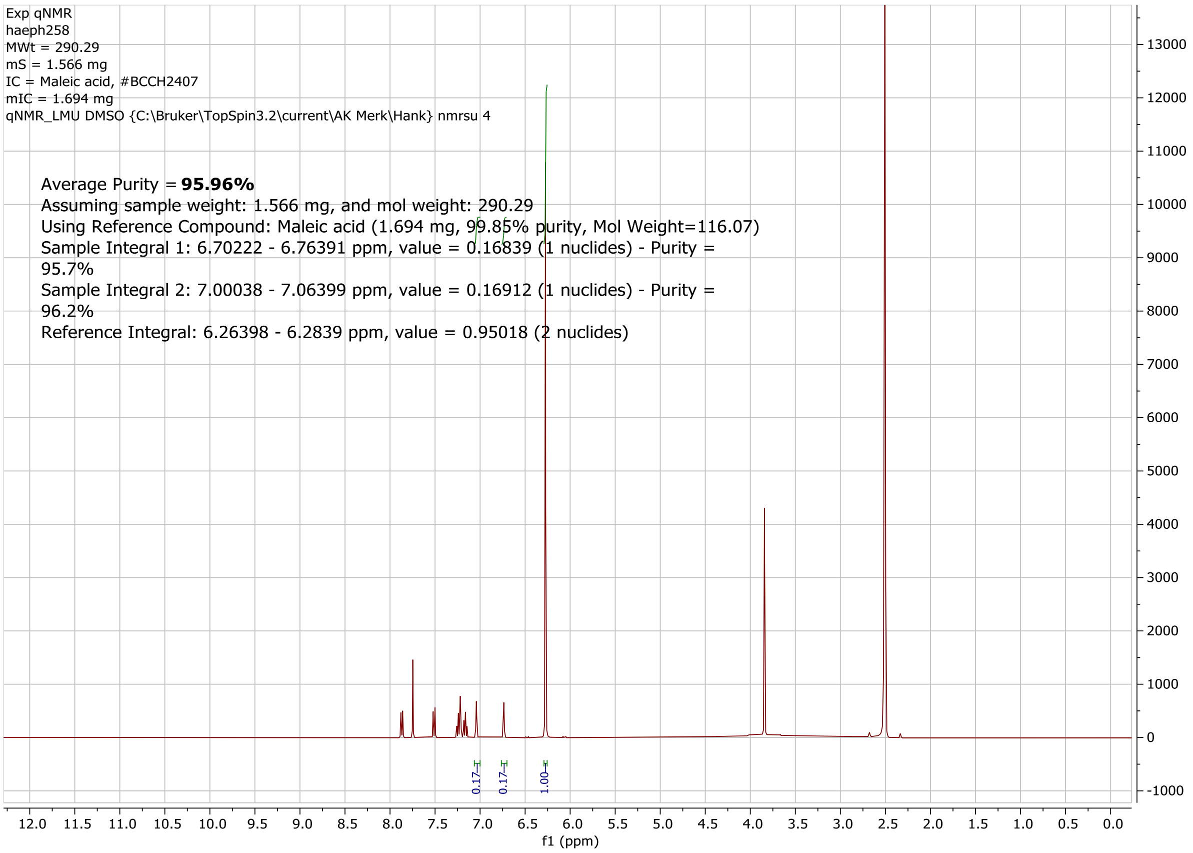

Compound **21**:

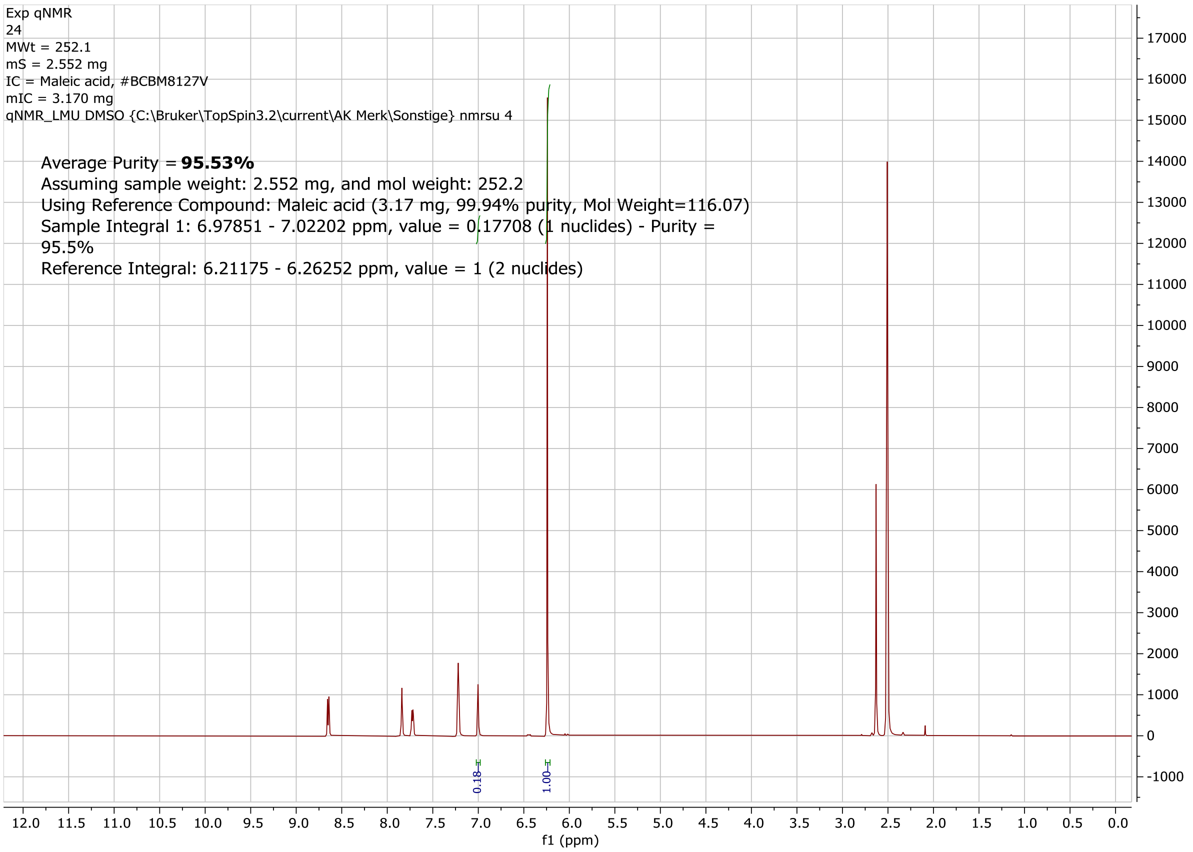

Compound **22**:

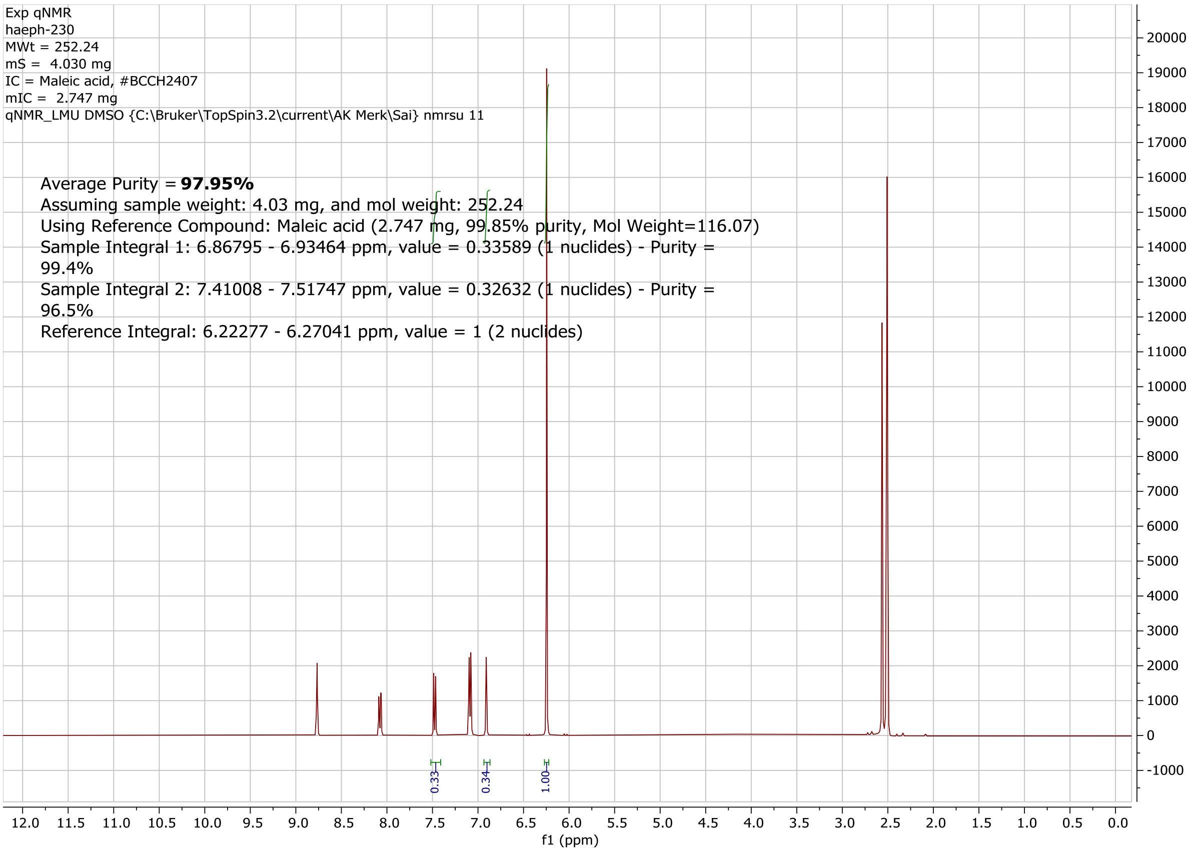

Compound **23**:

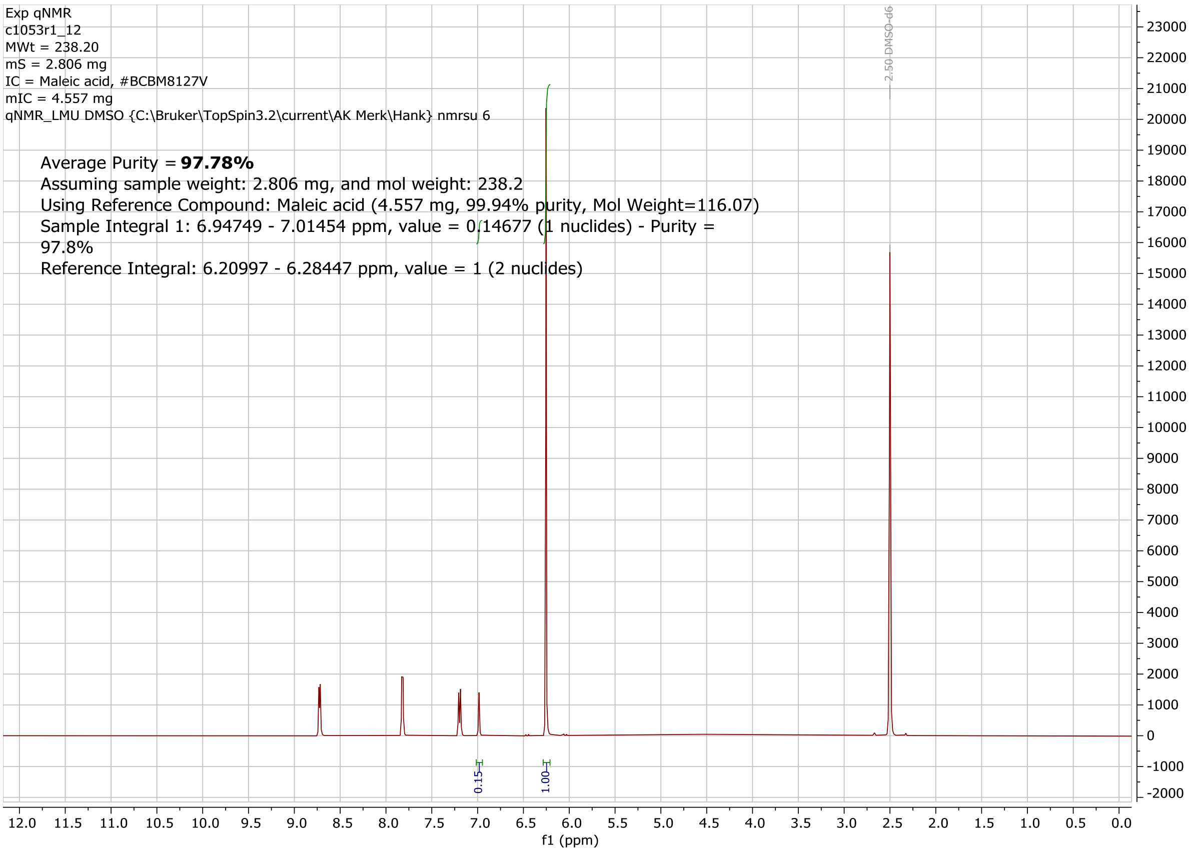

Compound **24**:

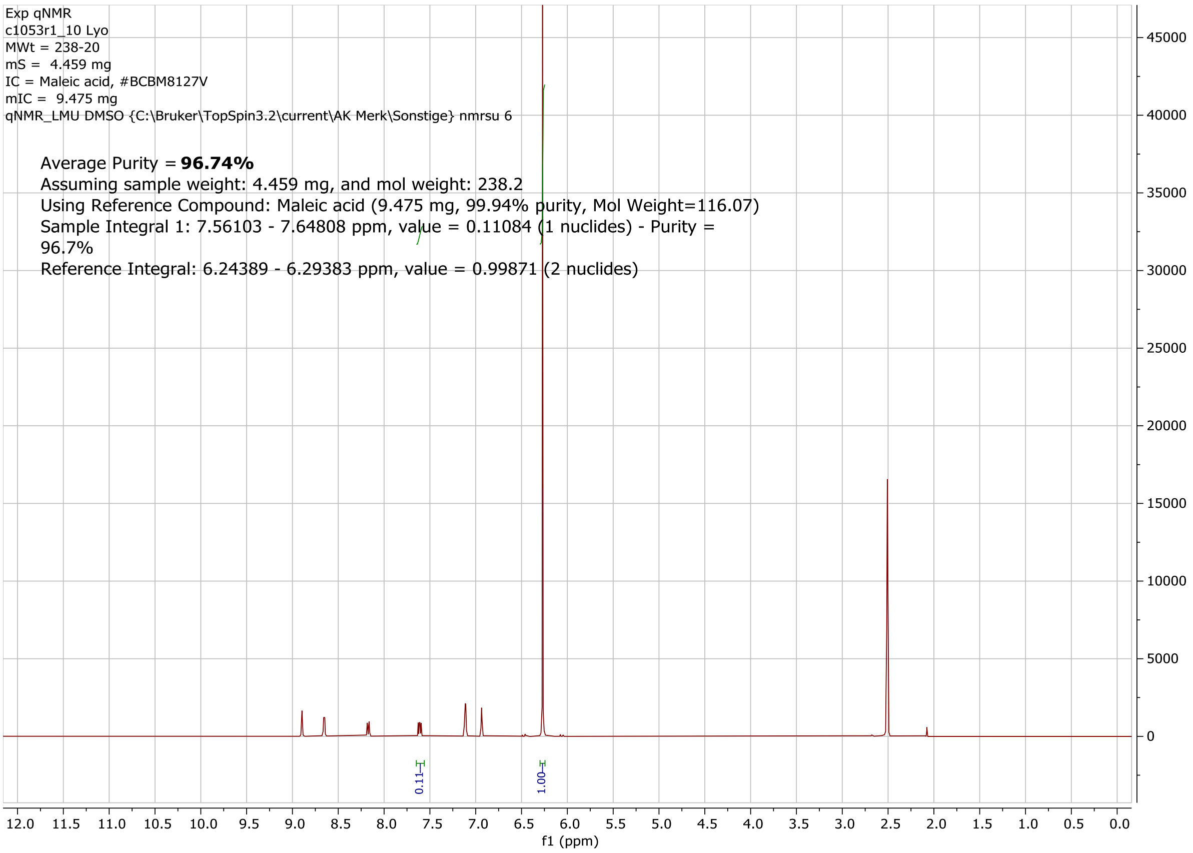

Compound **25**:

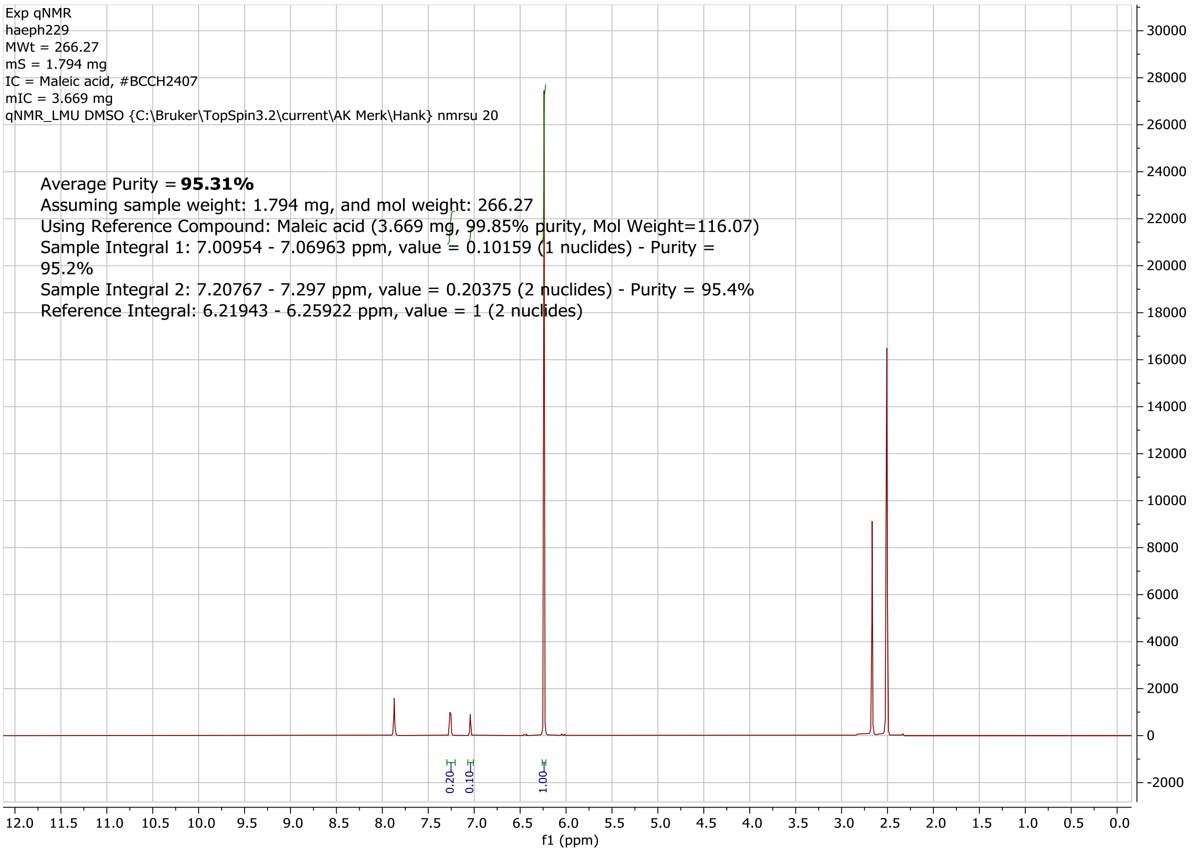

Compound **26**:

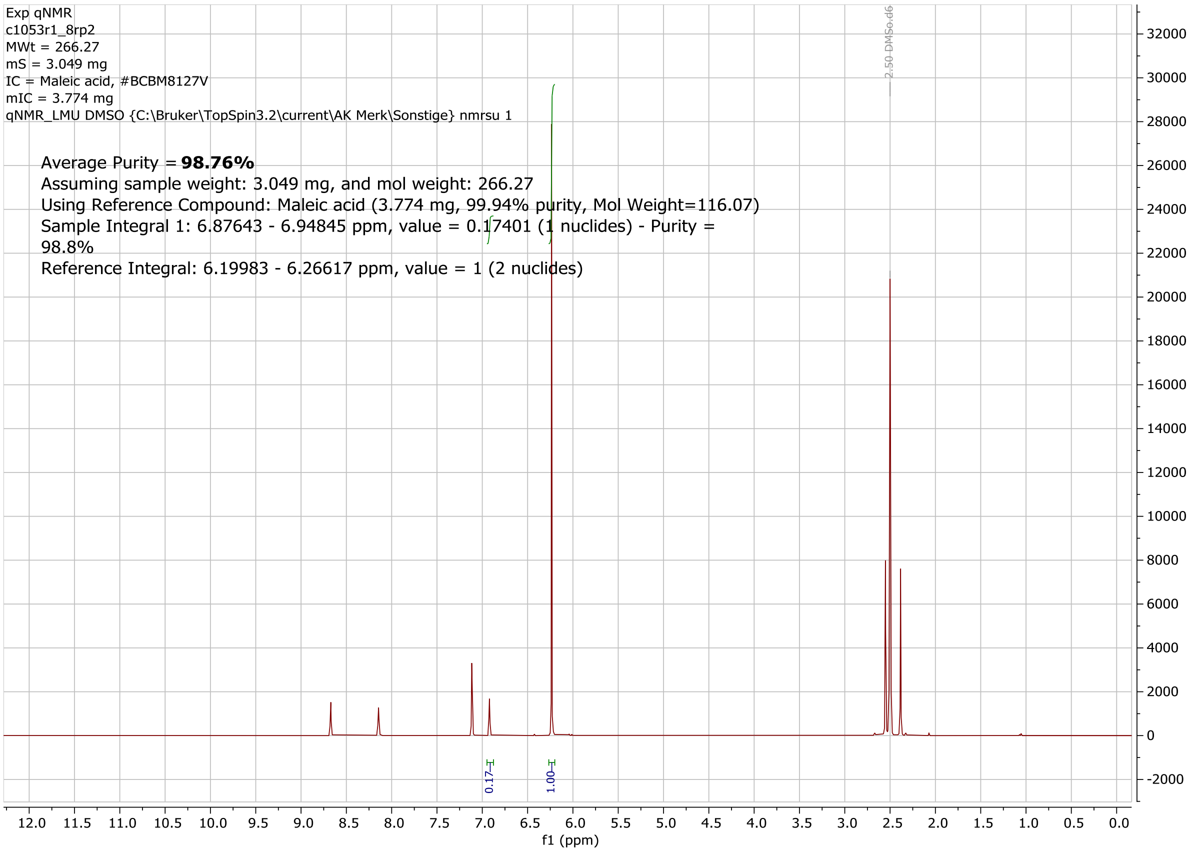

Compound **27**:

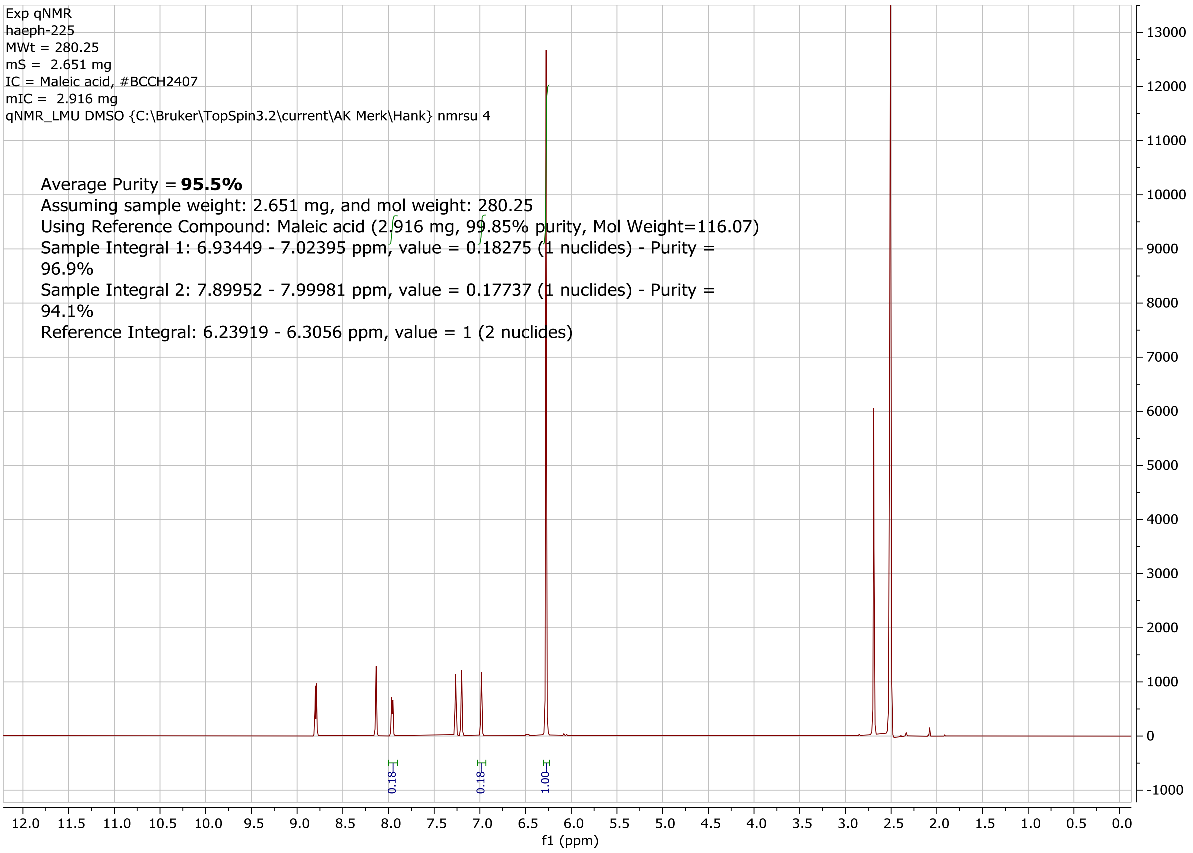

Compound **28**:

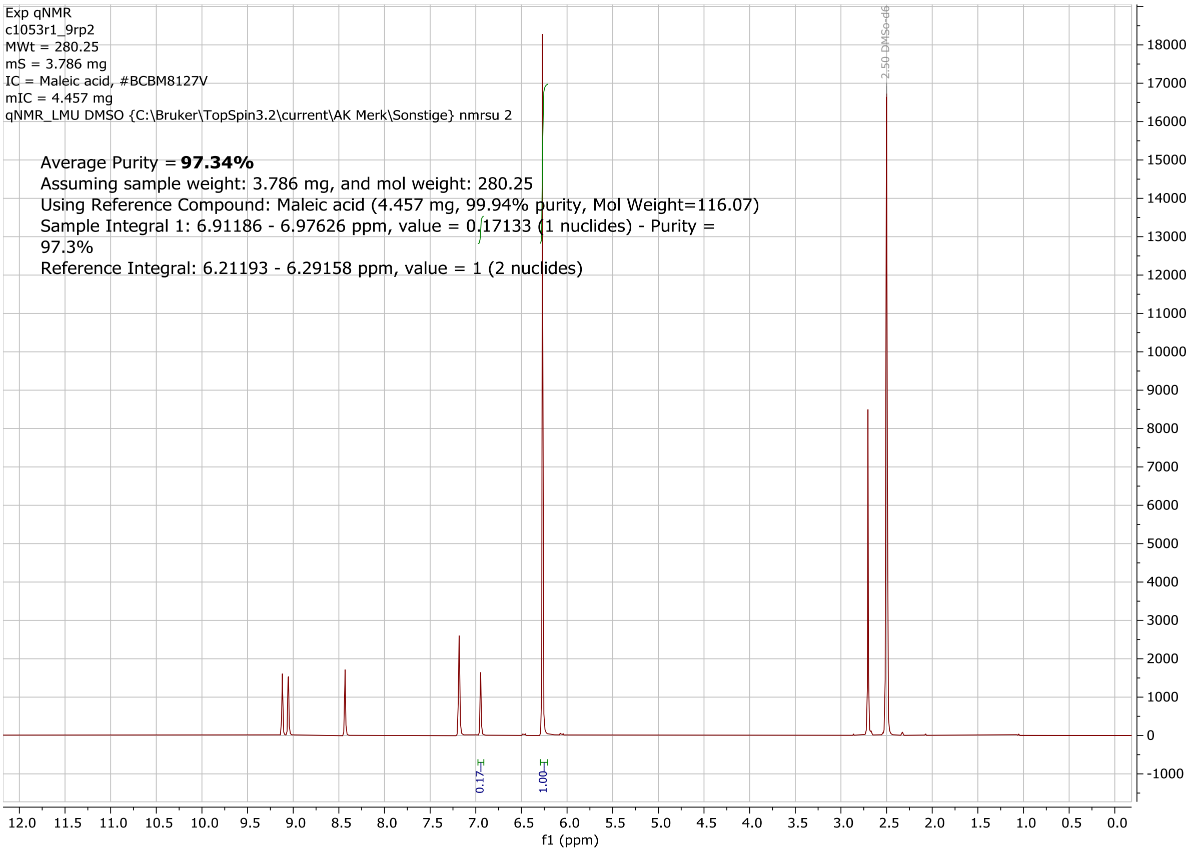

Compound **29**:

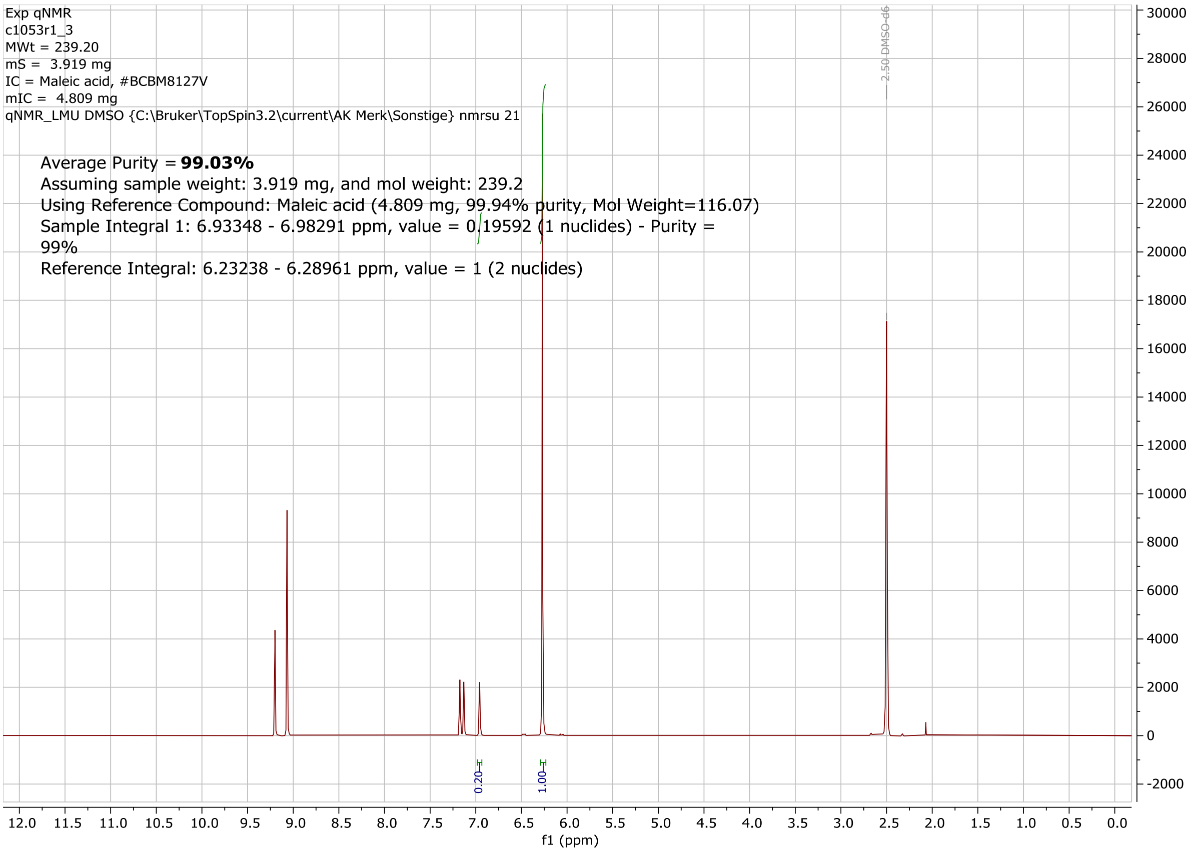

Compound **30**:

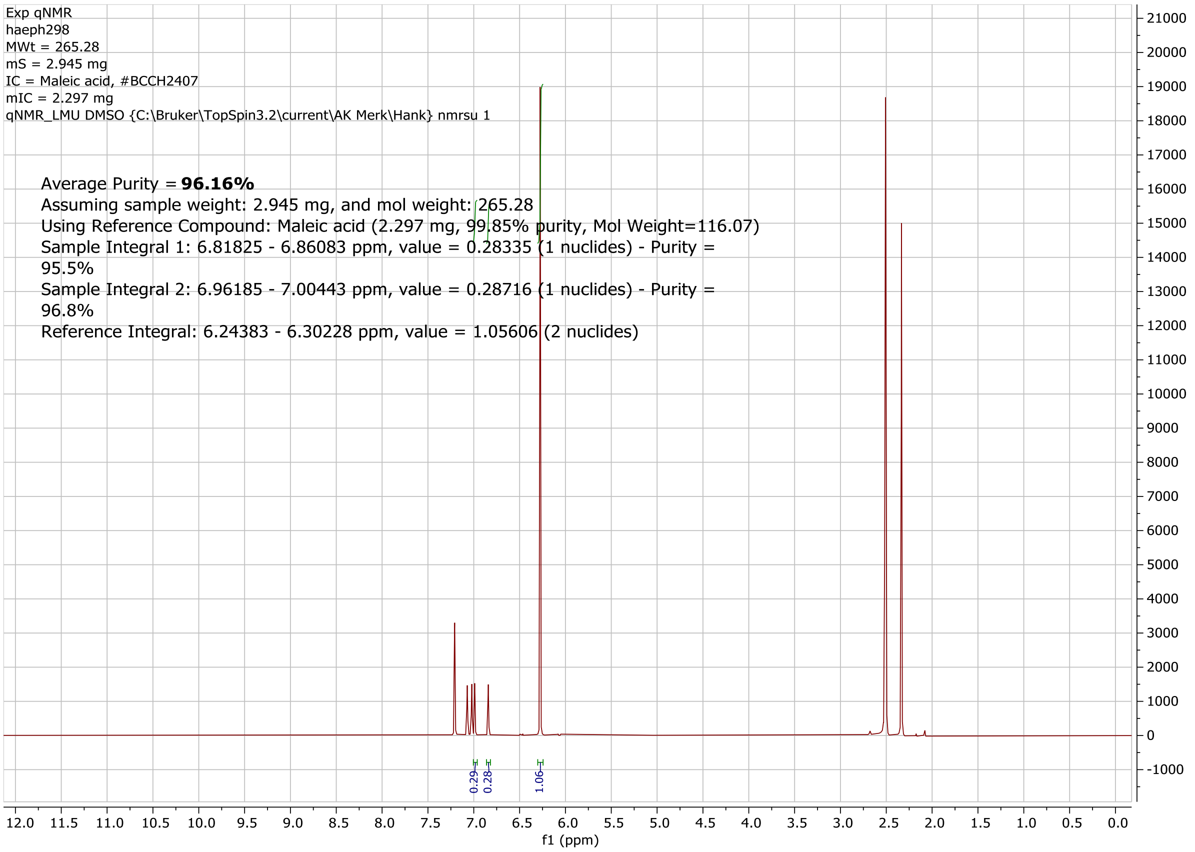

Compound **31**:

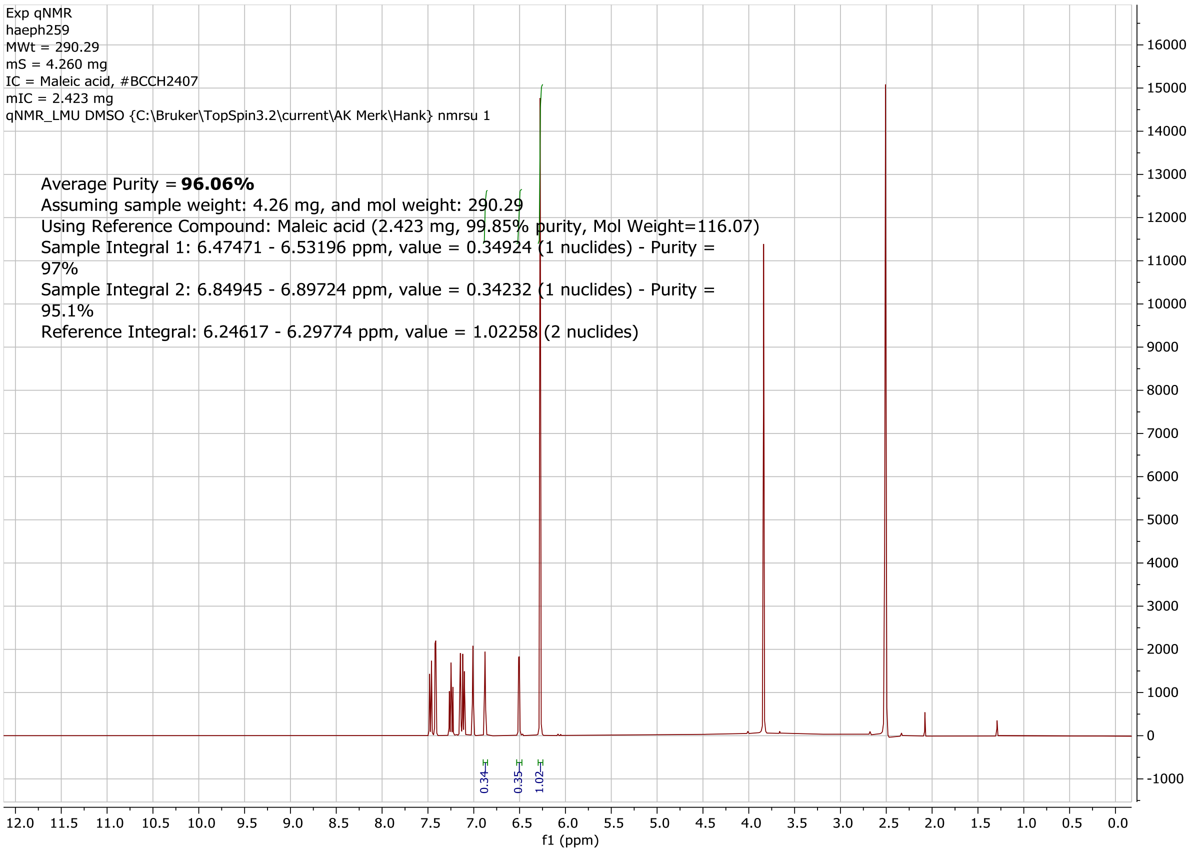

Compound **32**:

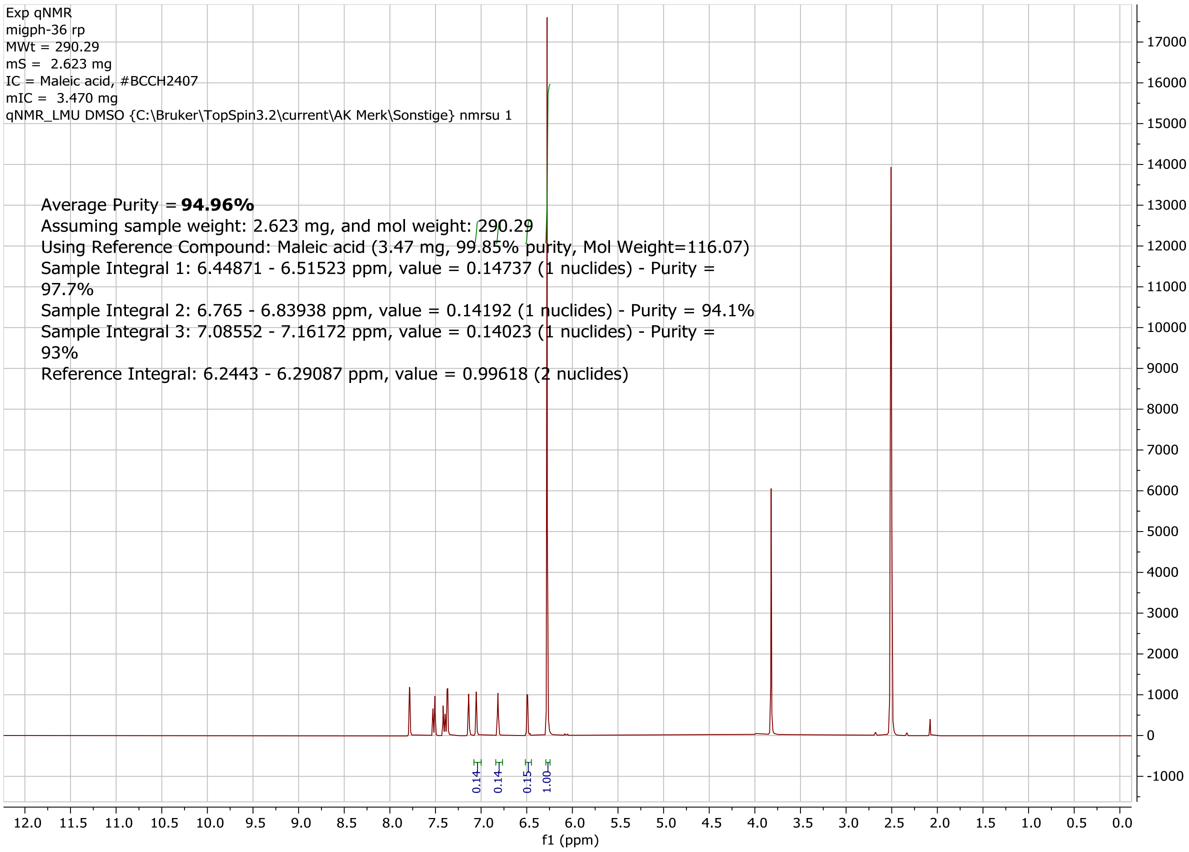

Compound **33**:

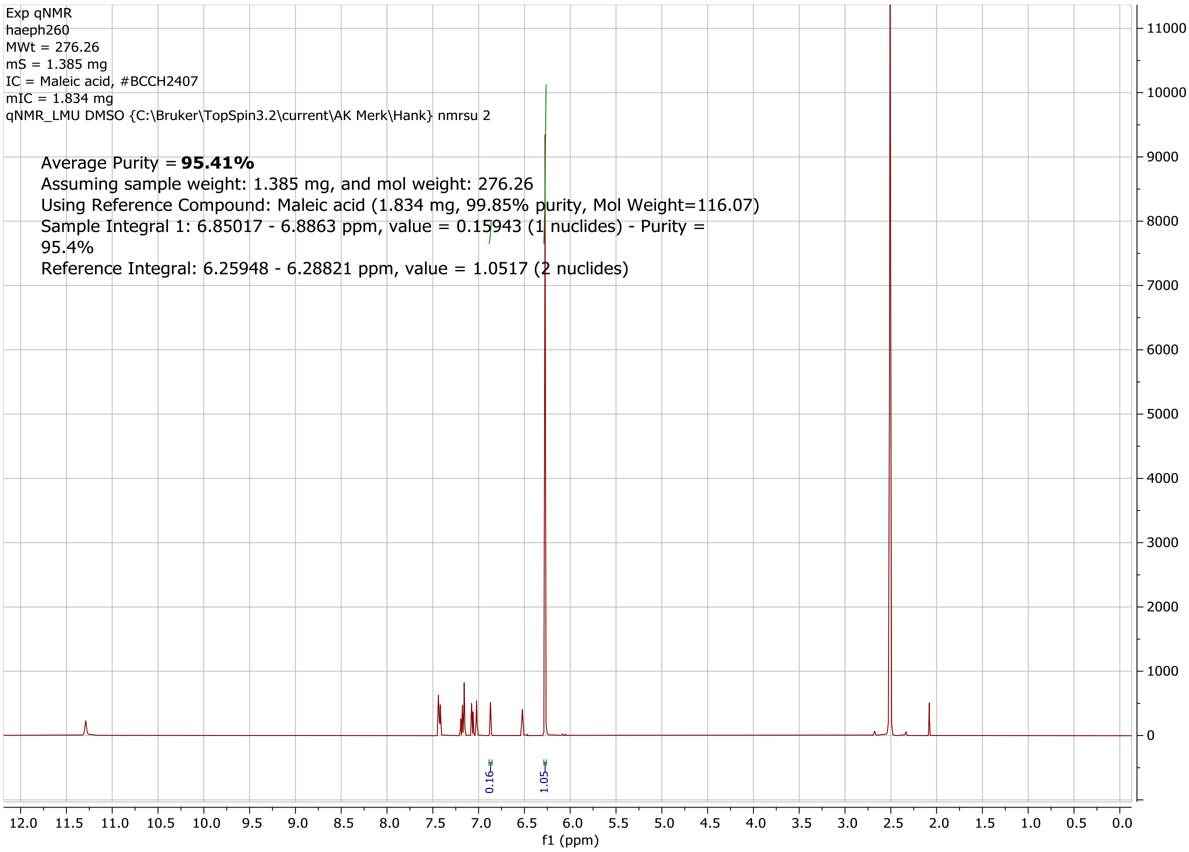

Compound **34**:

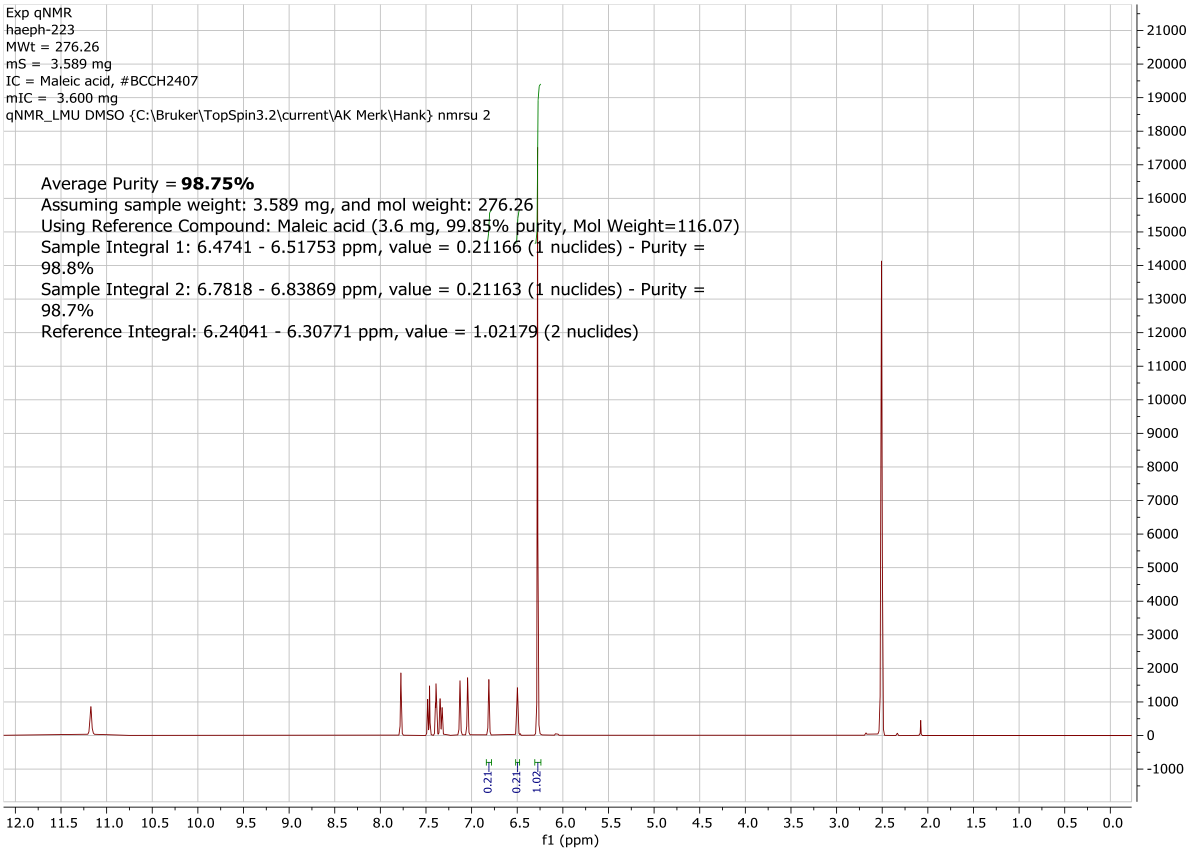

Compound **35**:

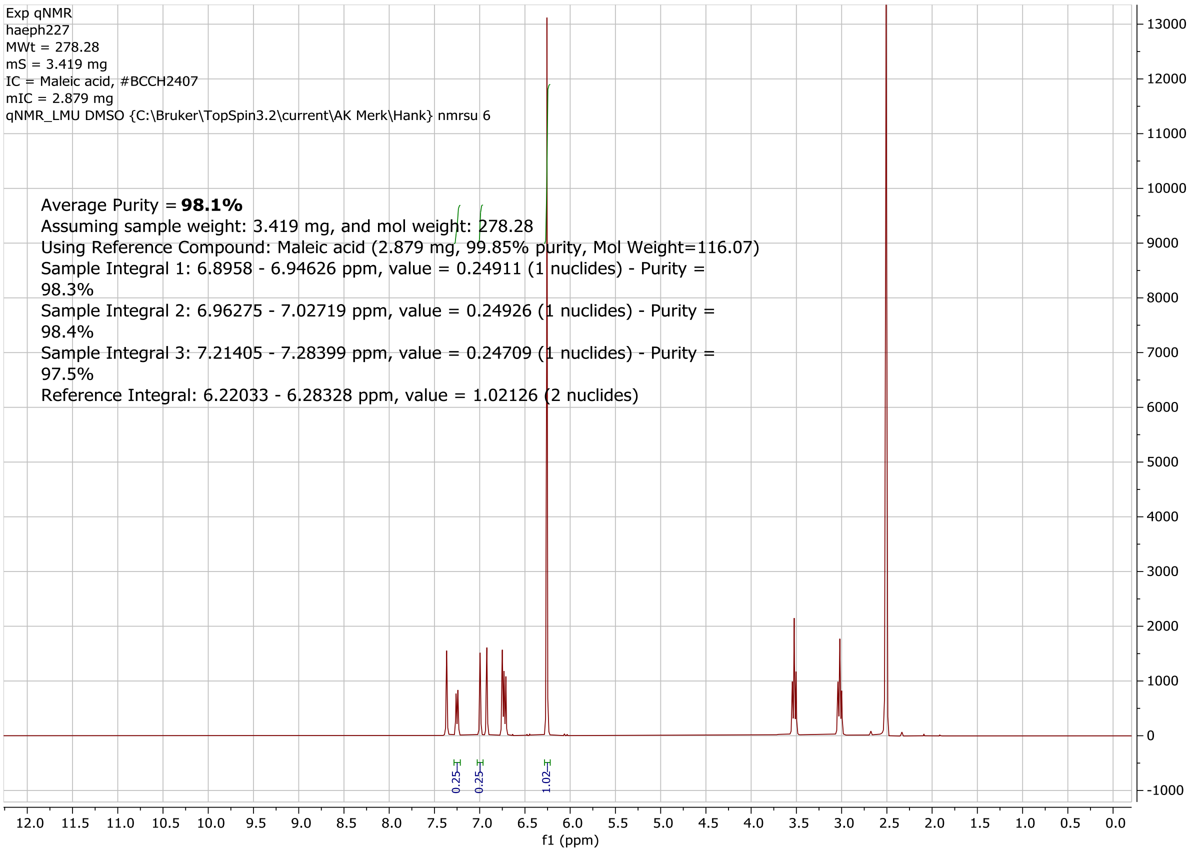

Compound **36**:

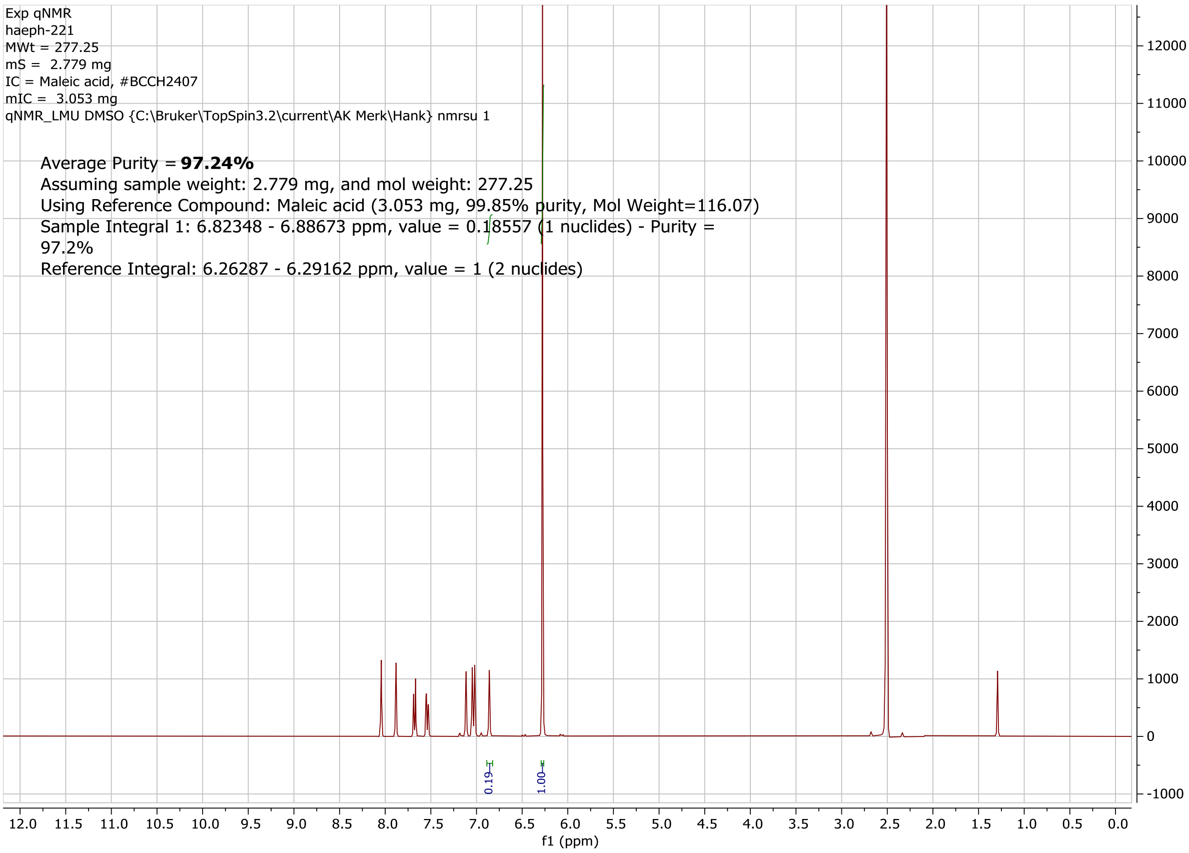

Compound **37**:

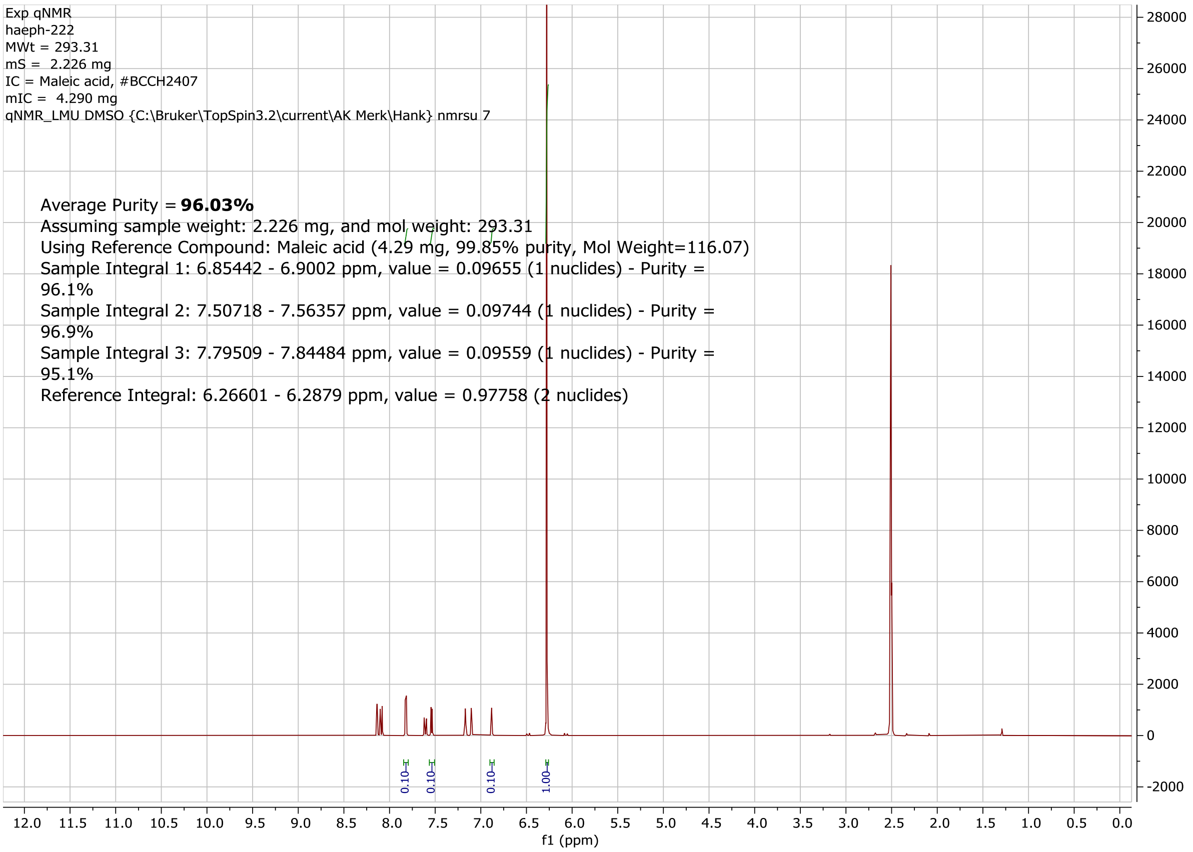

Compound **38**:

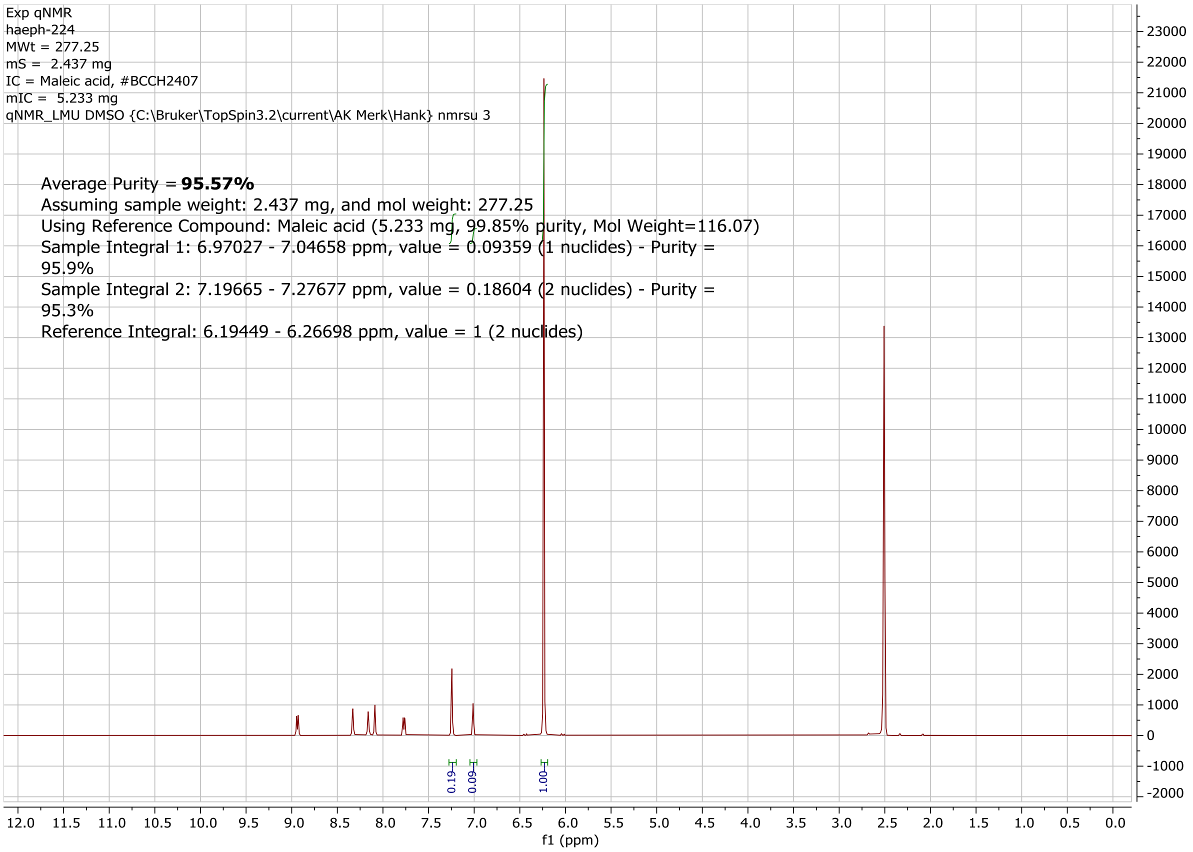

Compound **39**:

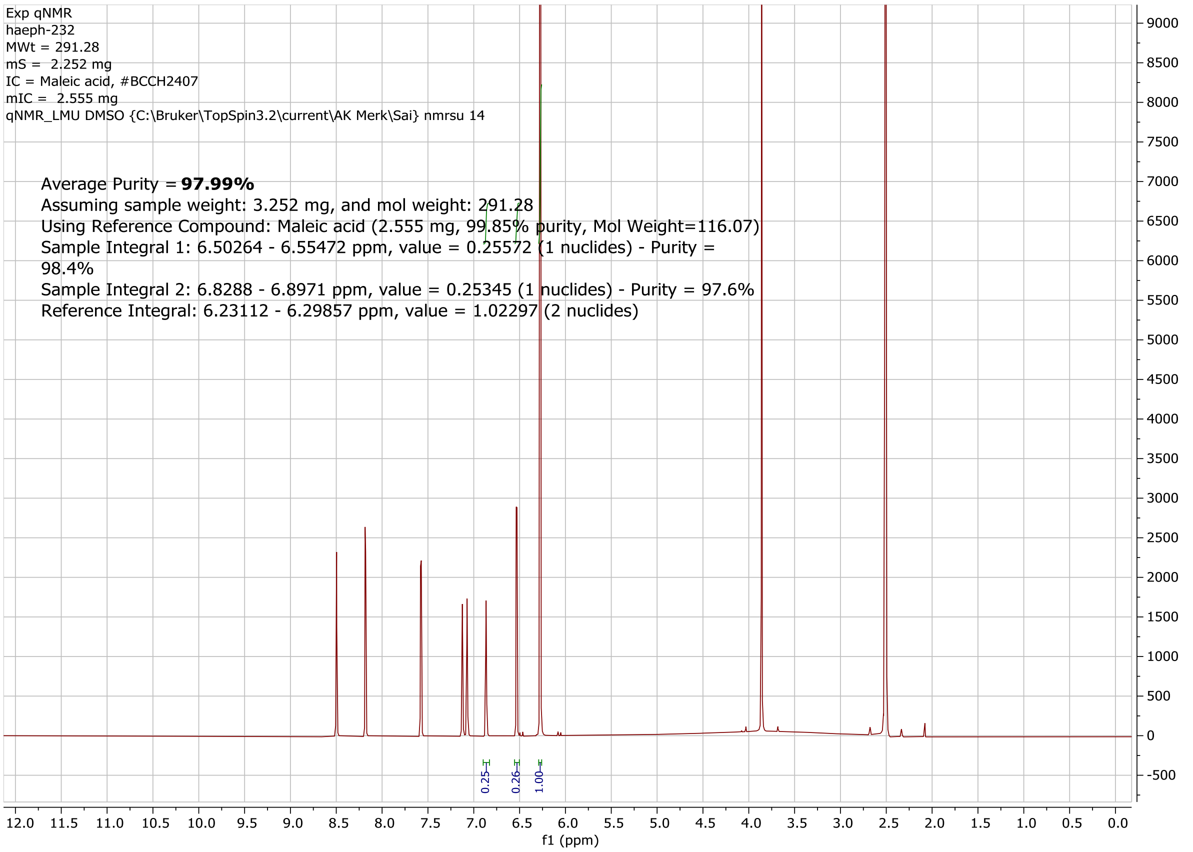

Compound **40**:

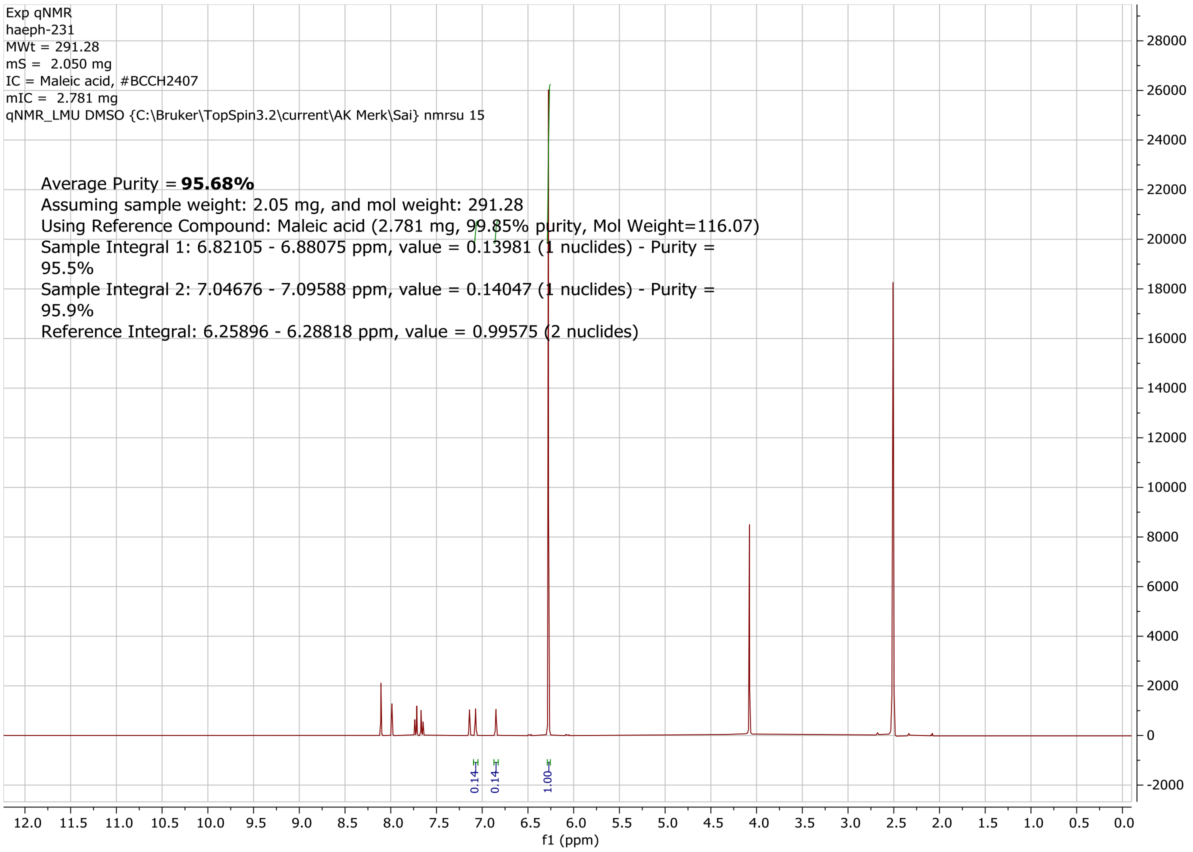

Compound **41**:

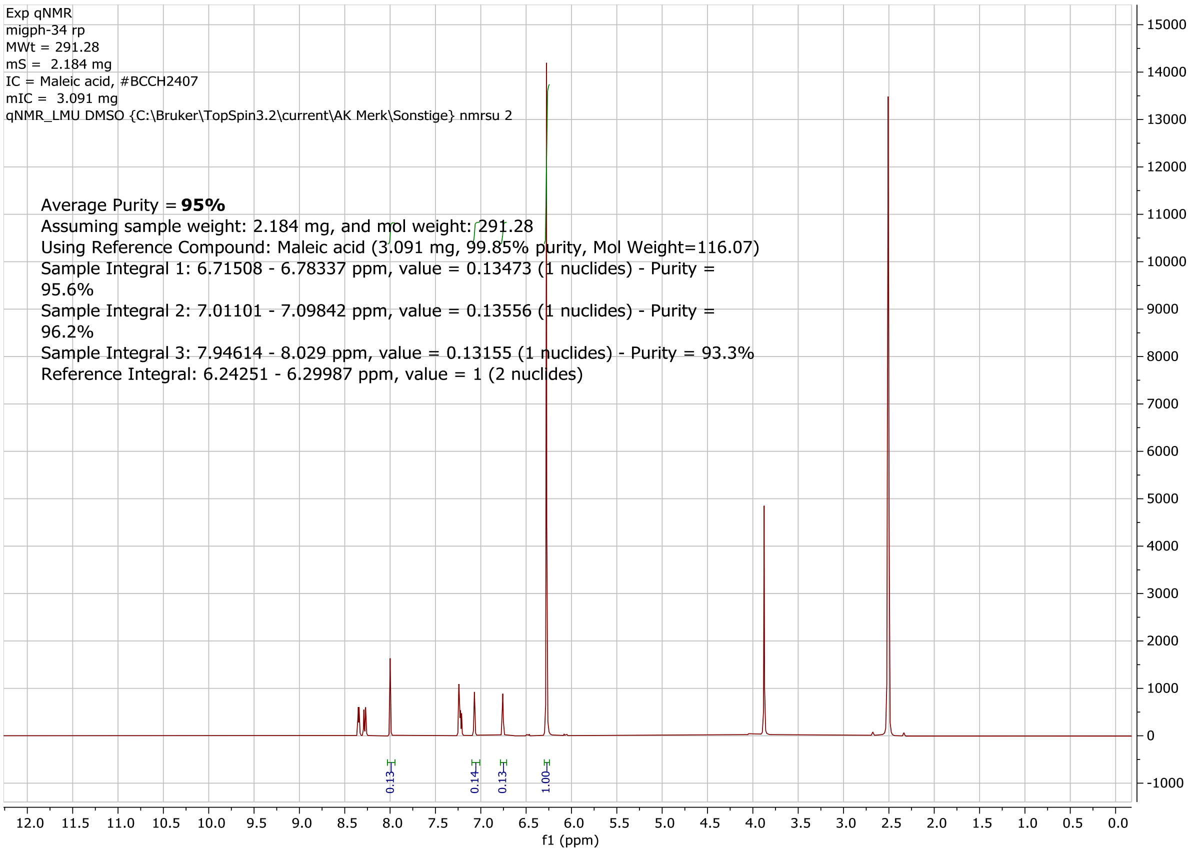

Compound **42**:

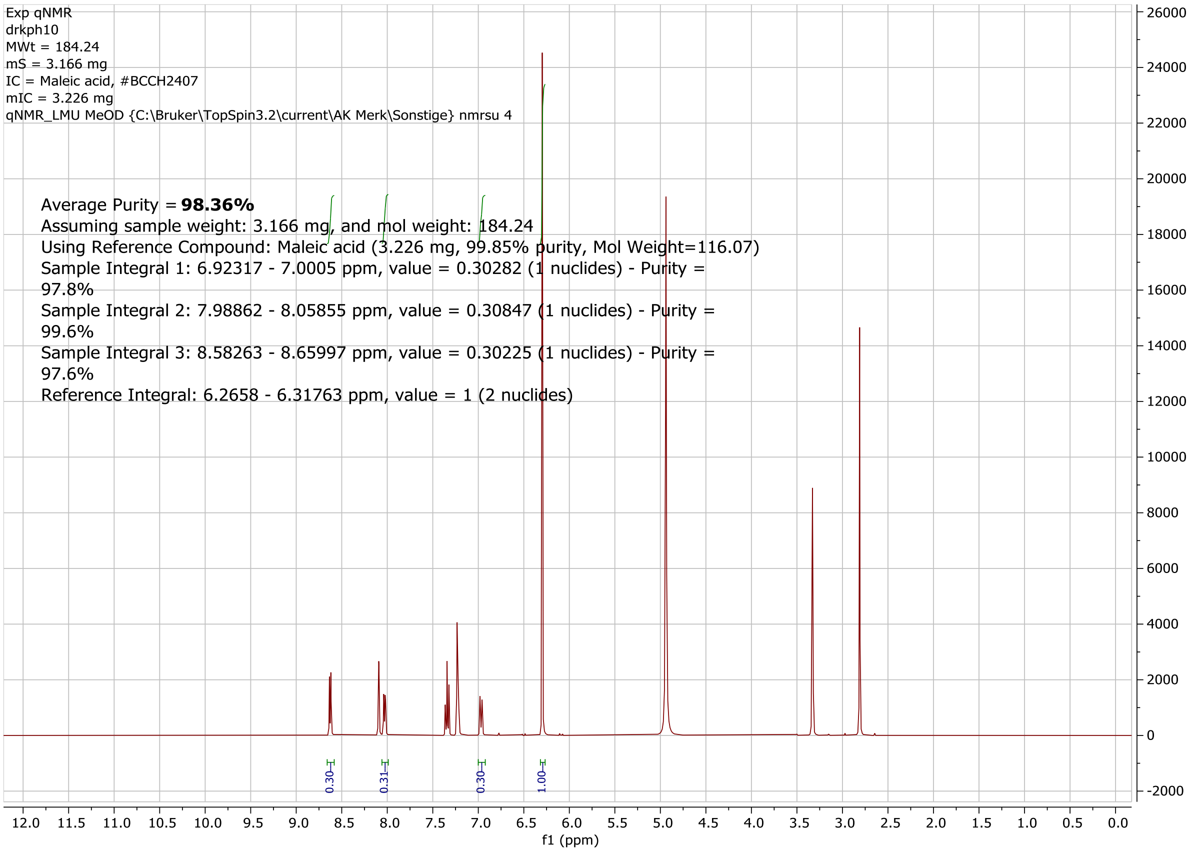

Compound **43**:

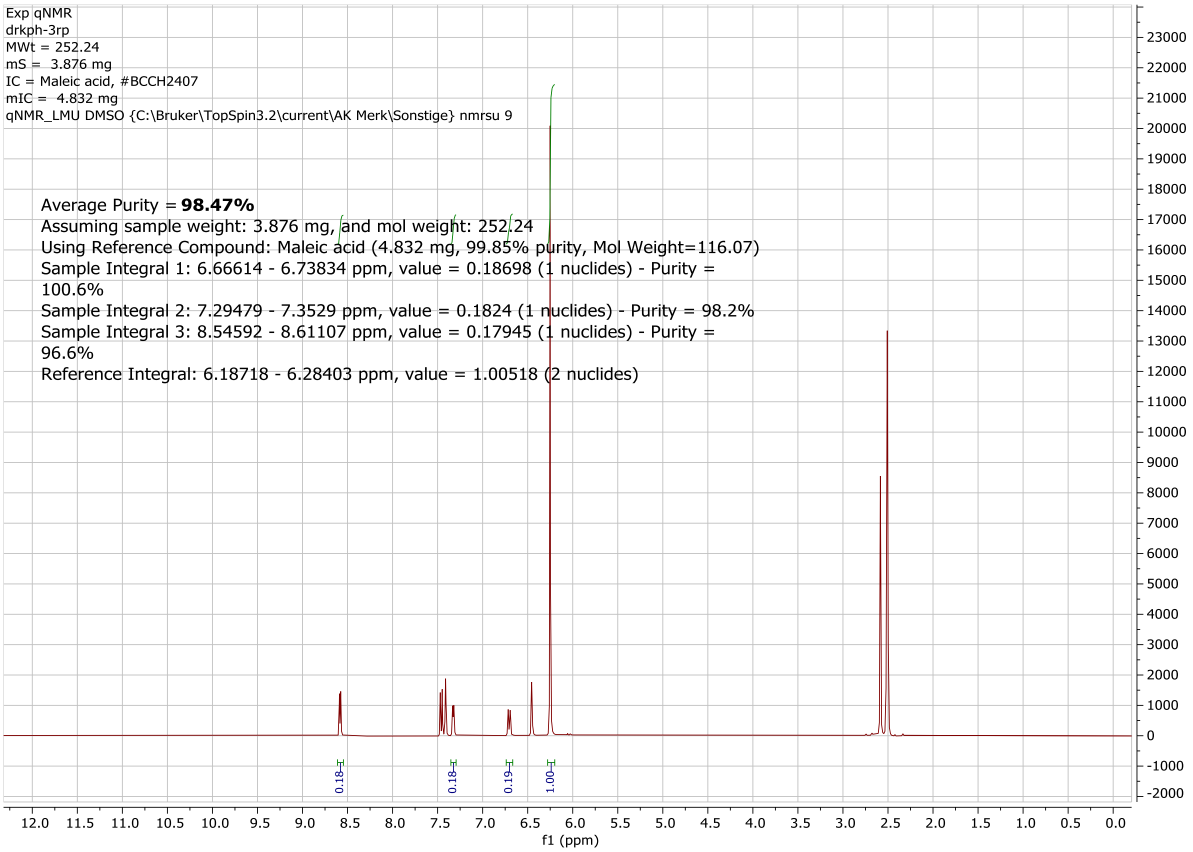

Compound **44**:

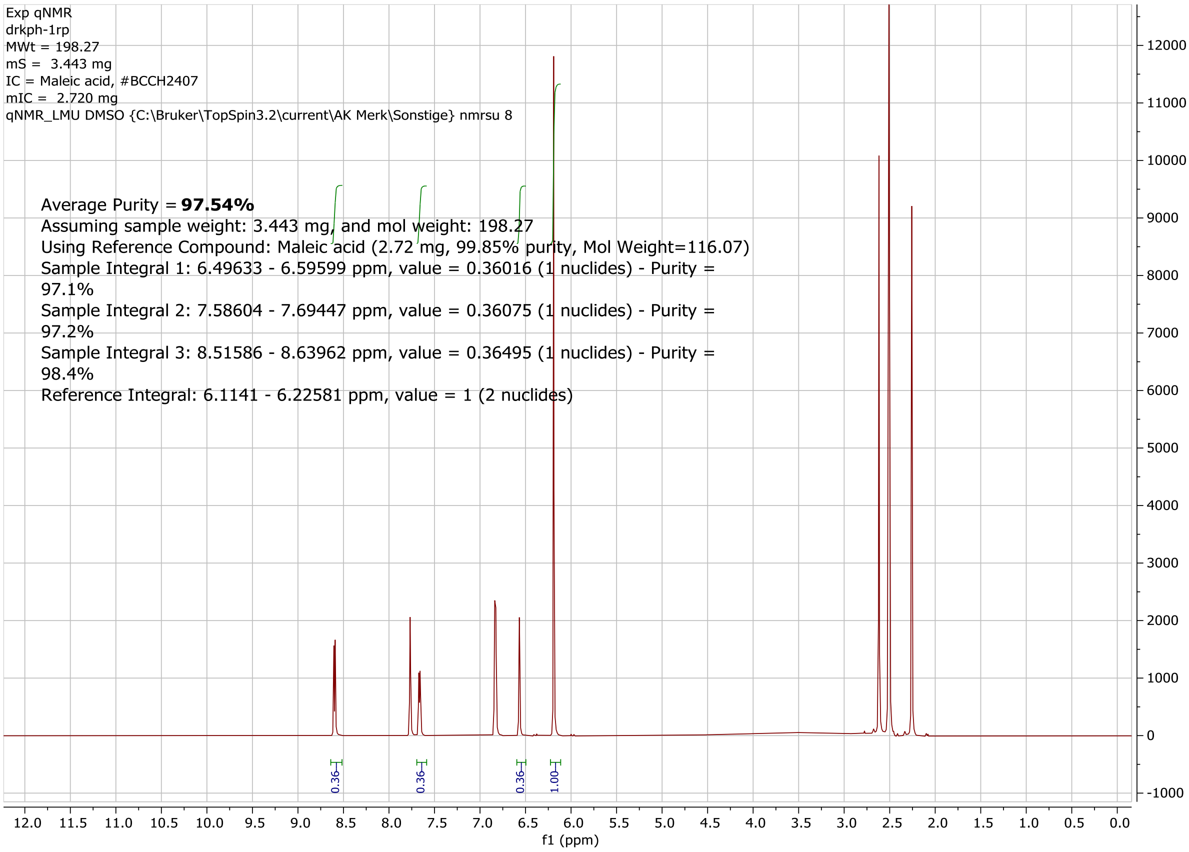

Compound **45**:

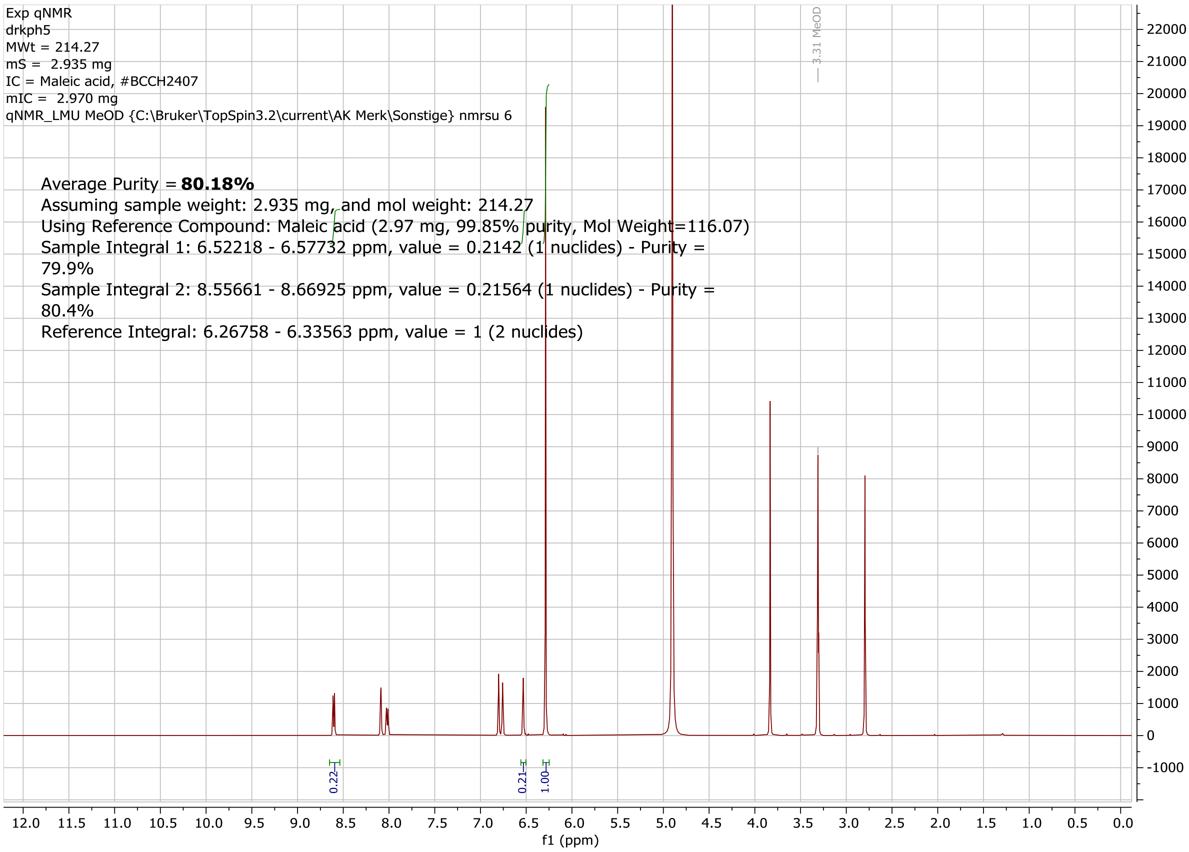

Compound **46**:

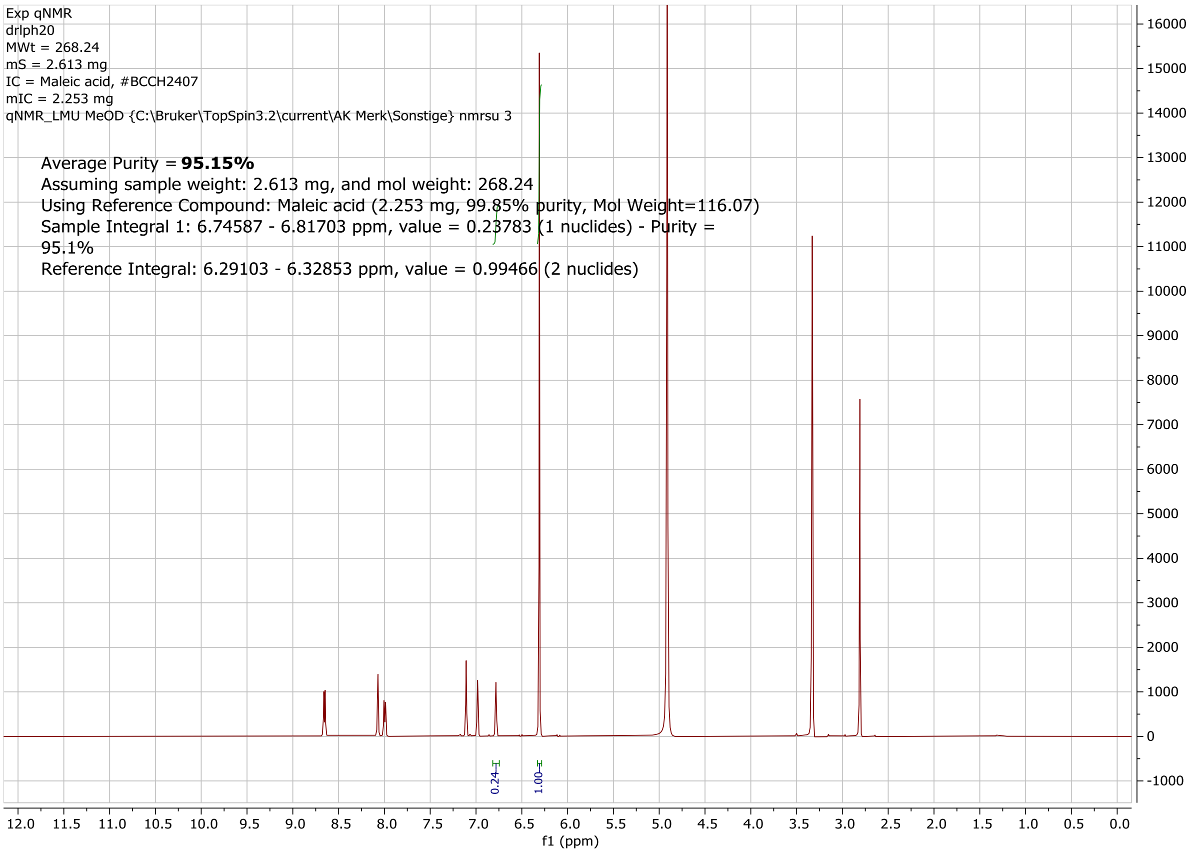

Compound **47**:

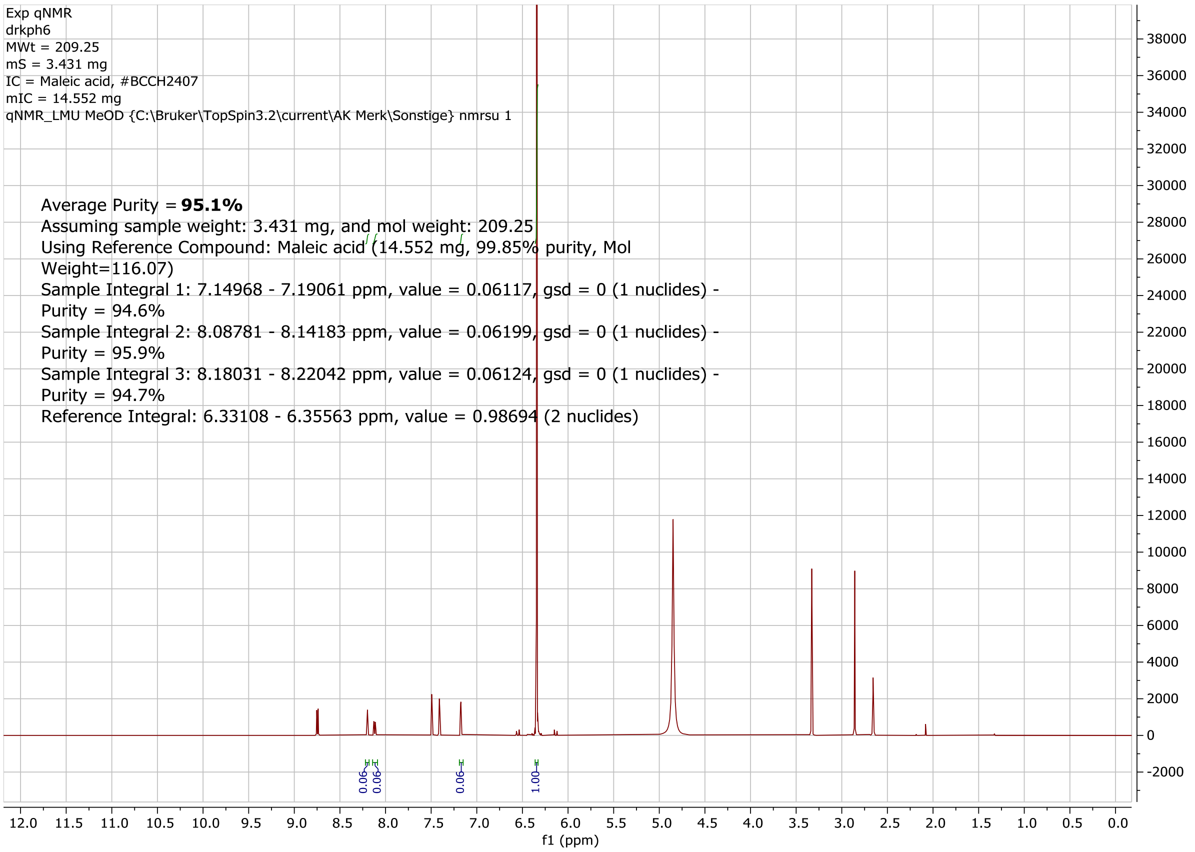

Compound **48**:

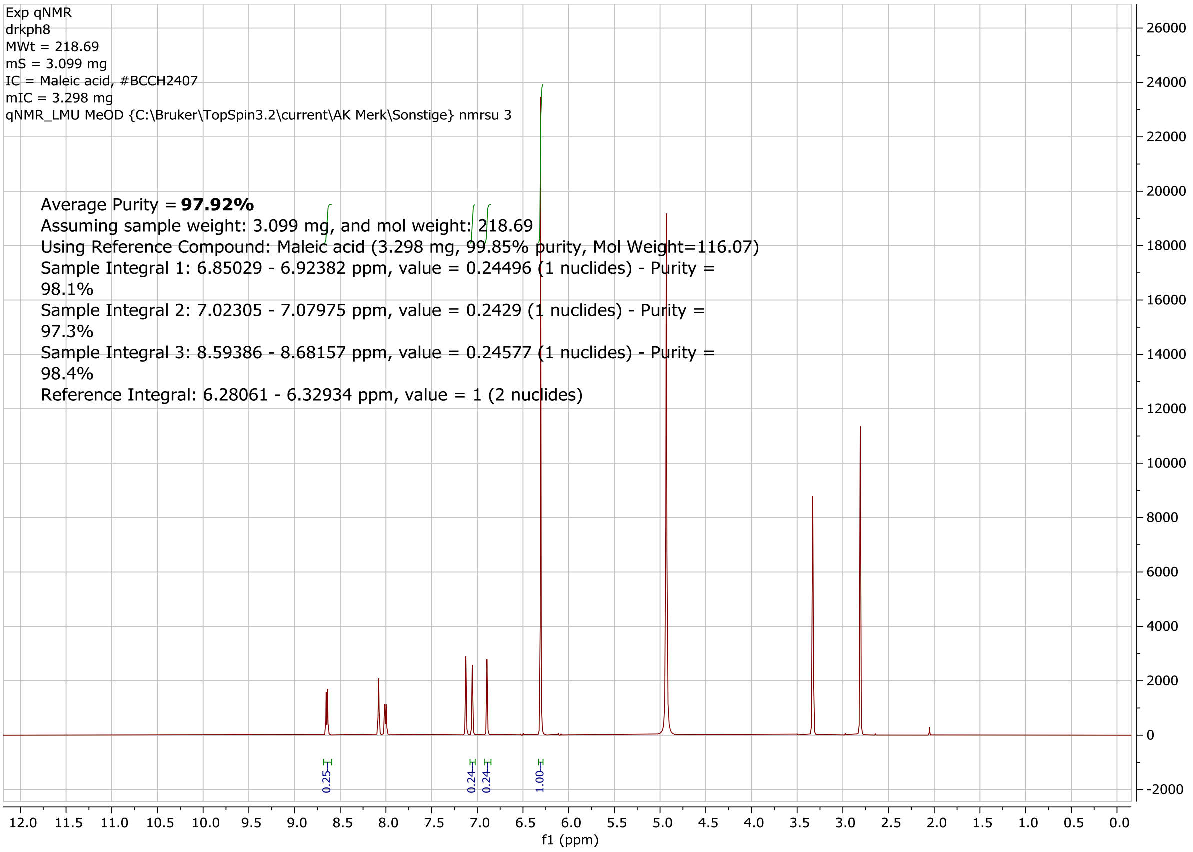

Compound **49**:

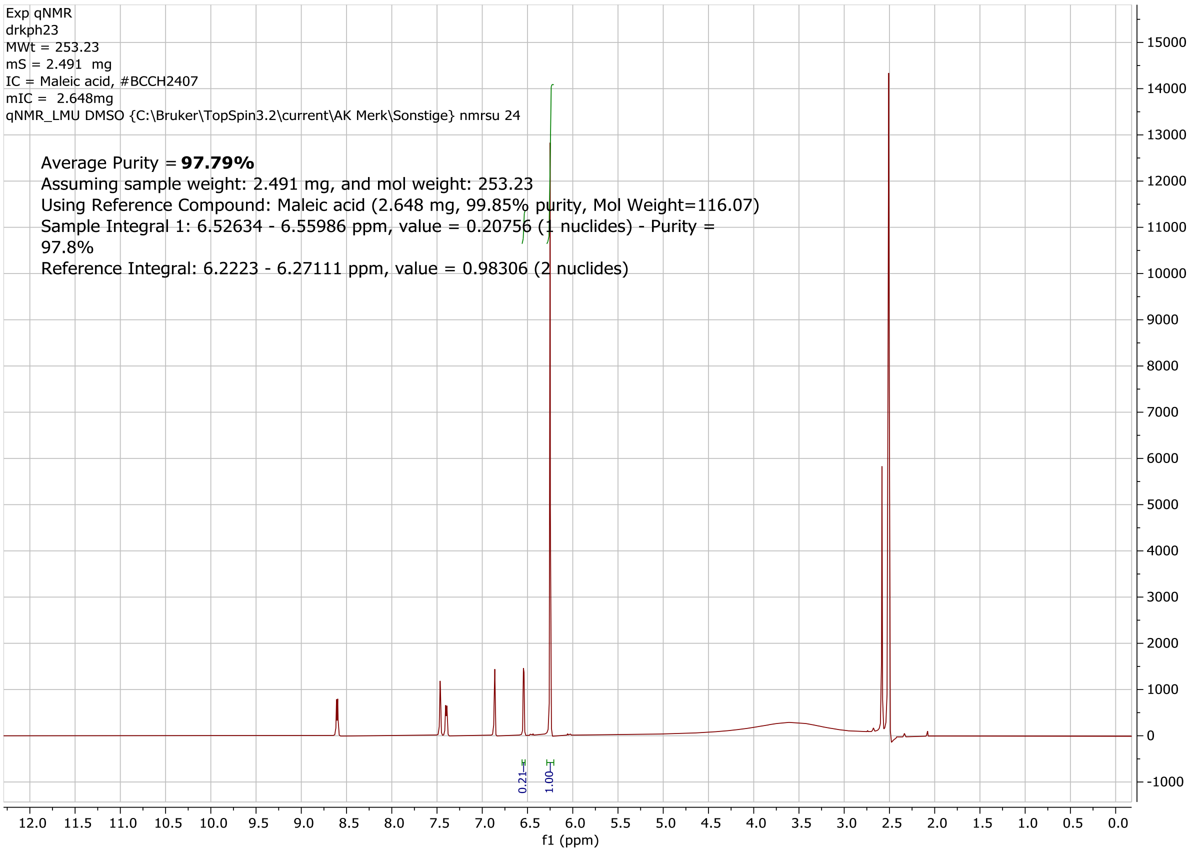

Compound **50**:

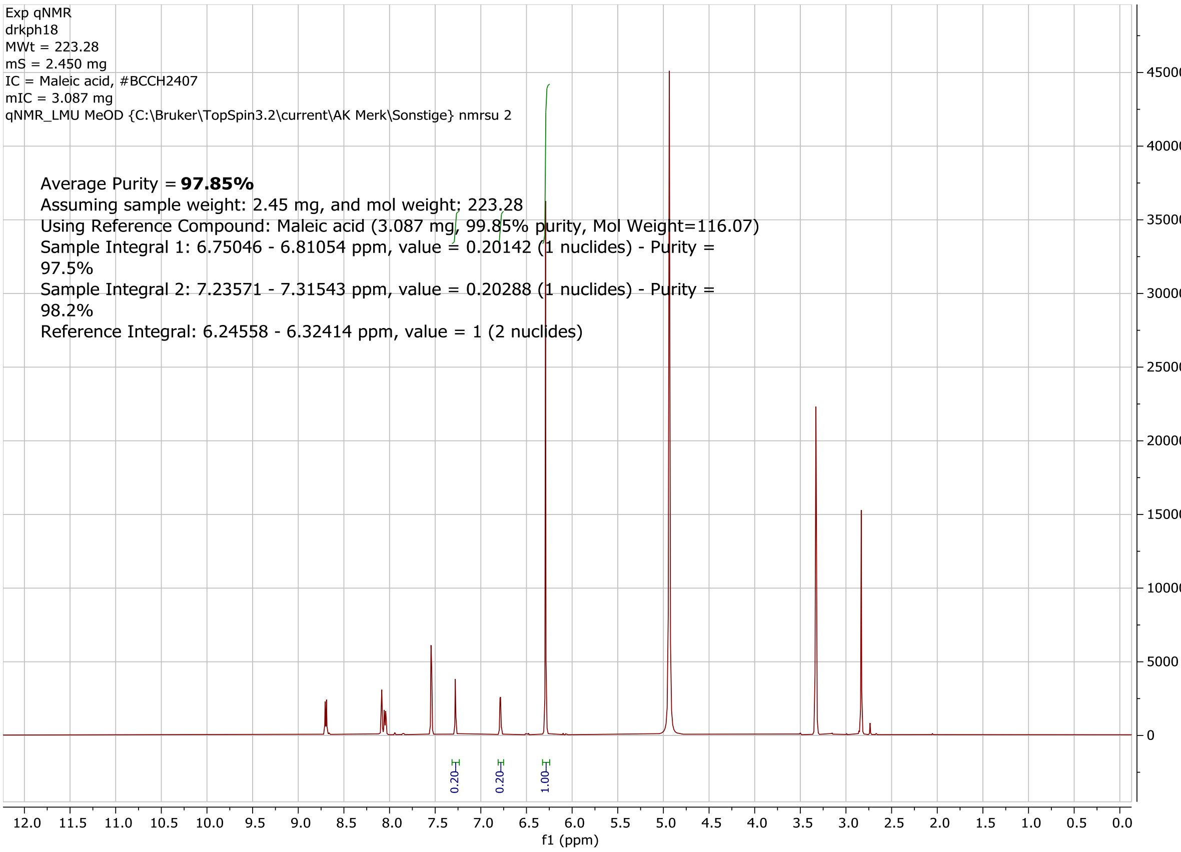

Compound **51**:

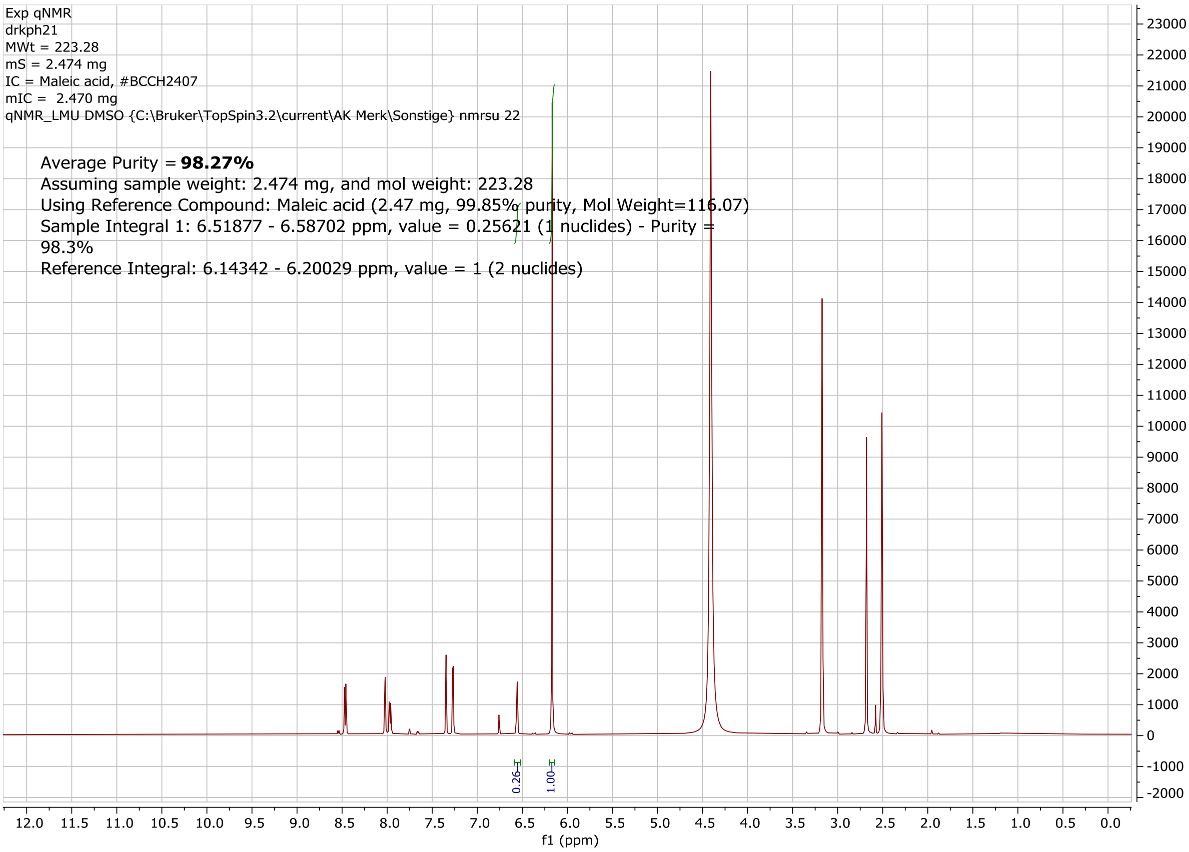

Compound **52**:

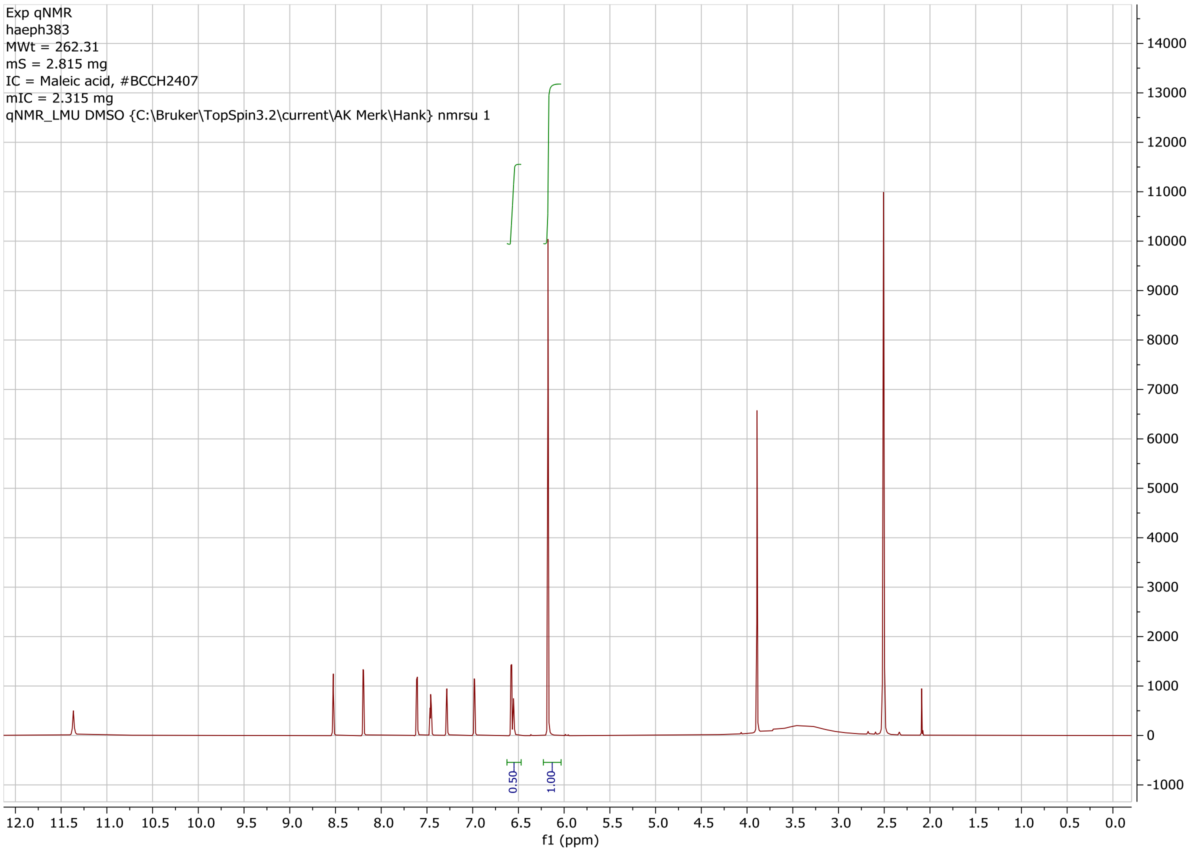

Compound **53**:

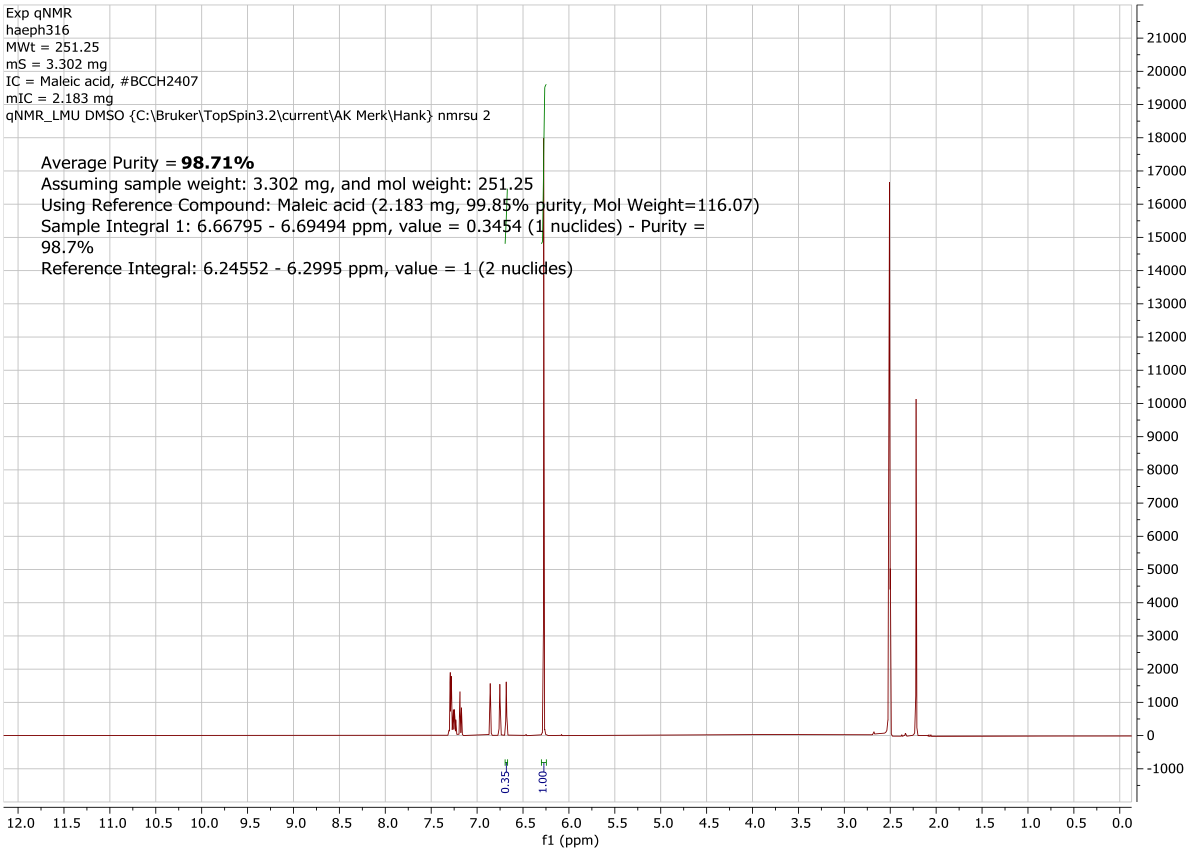

Compound **54**:

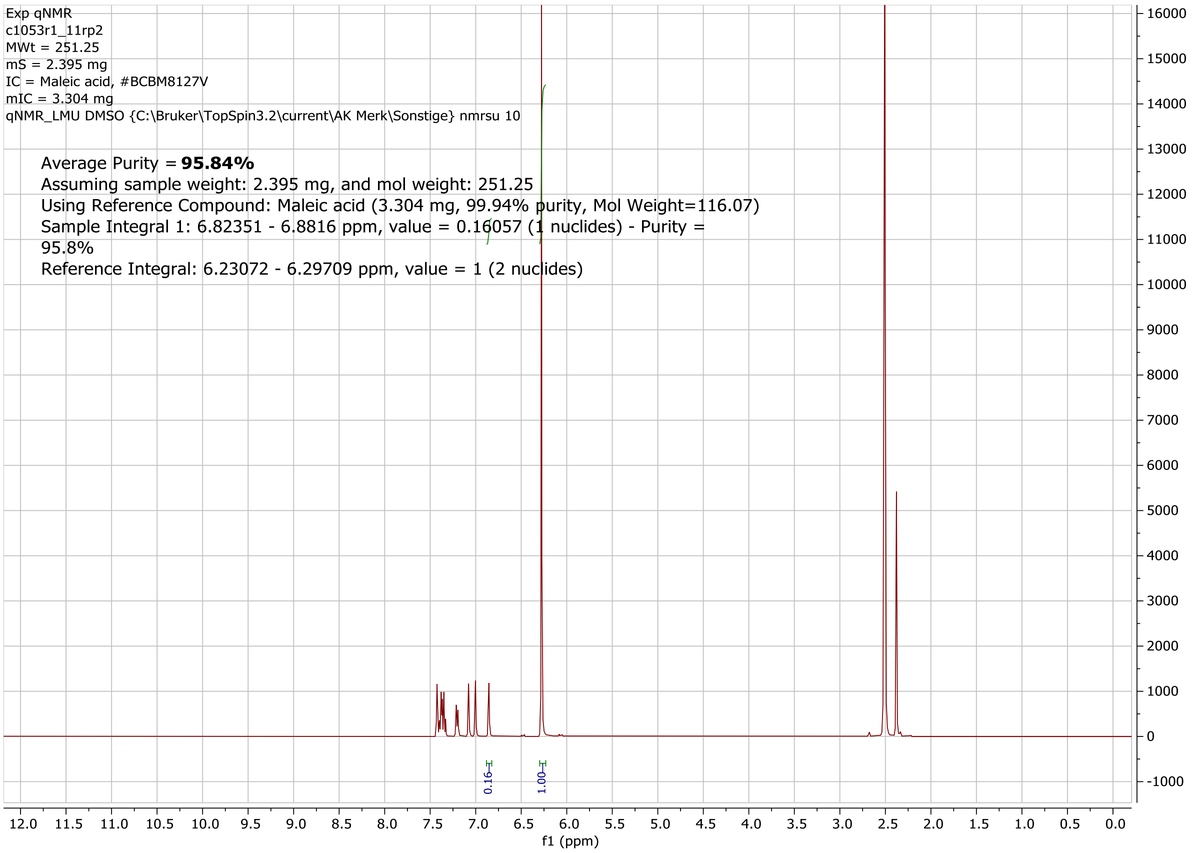

Compound **55**:

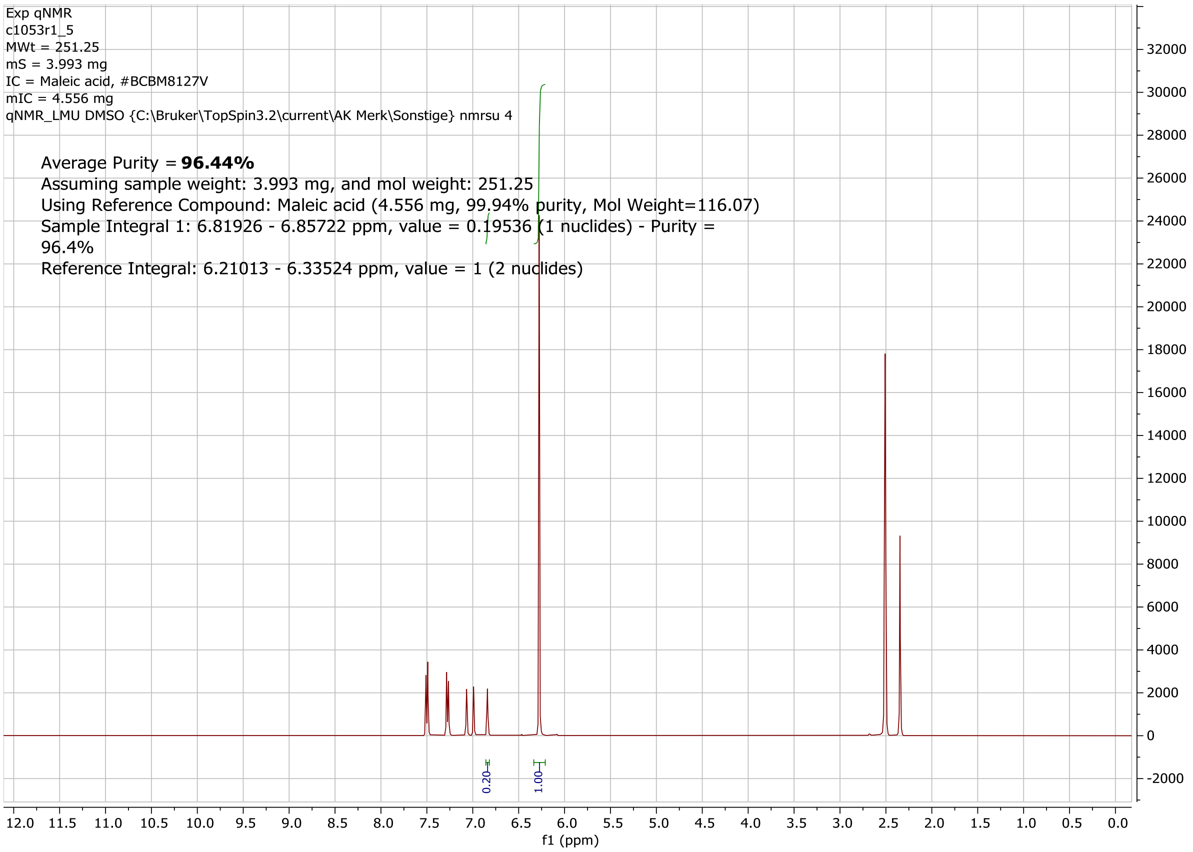

Compound **56**:

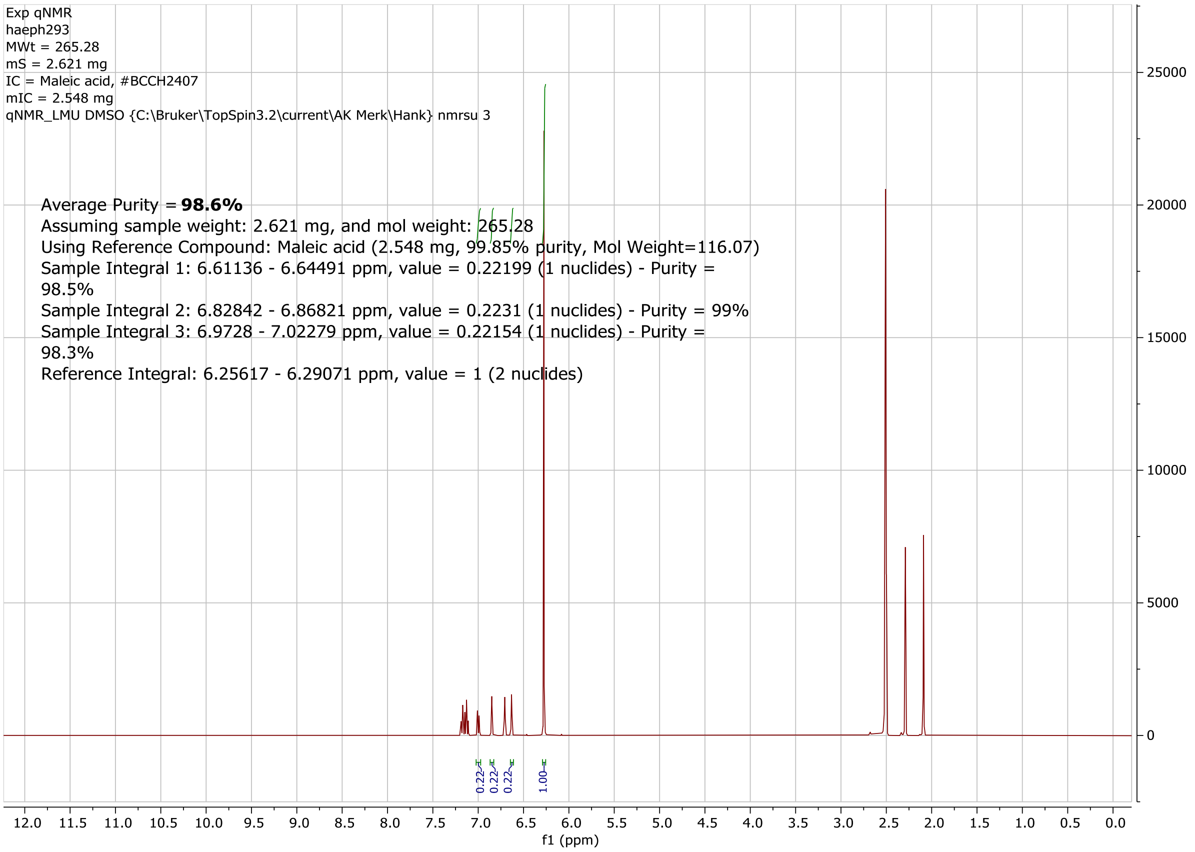

Compound **57**:

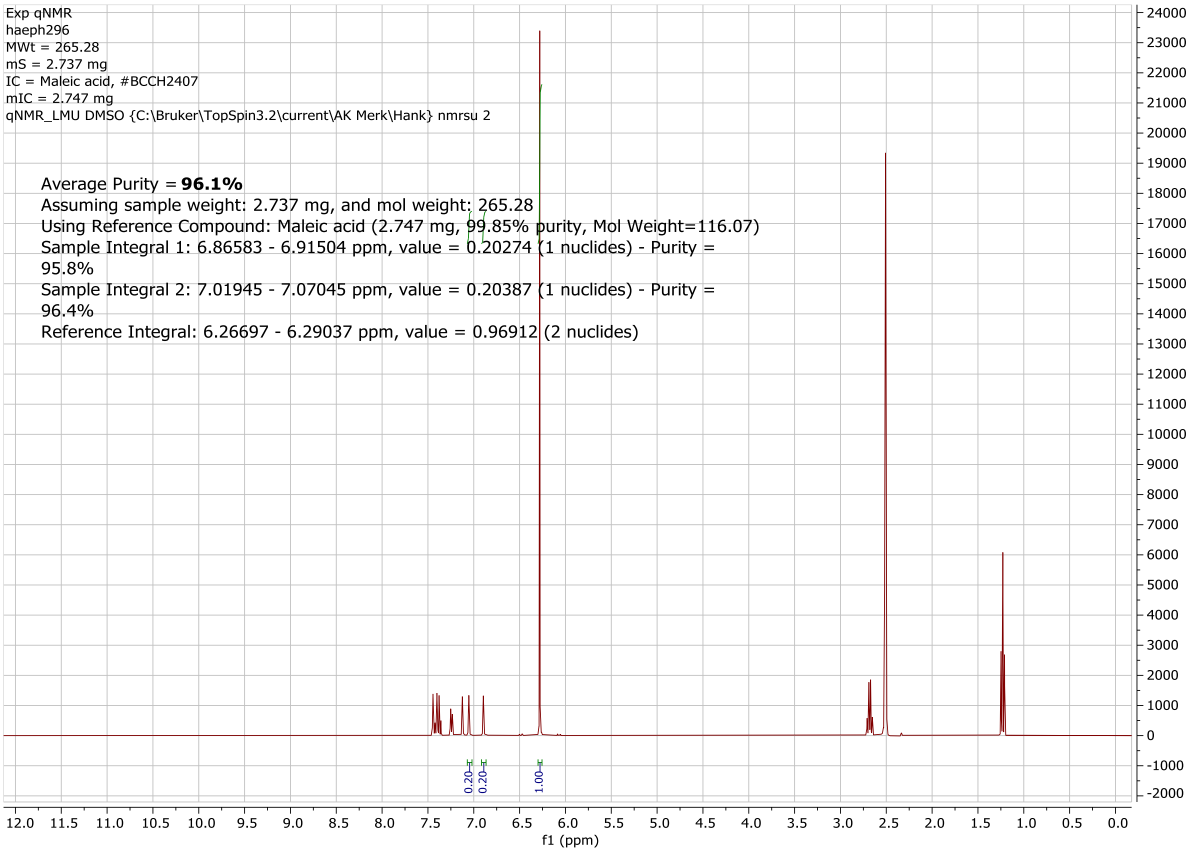

Compound **58**:

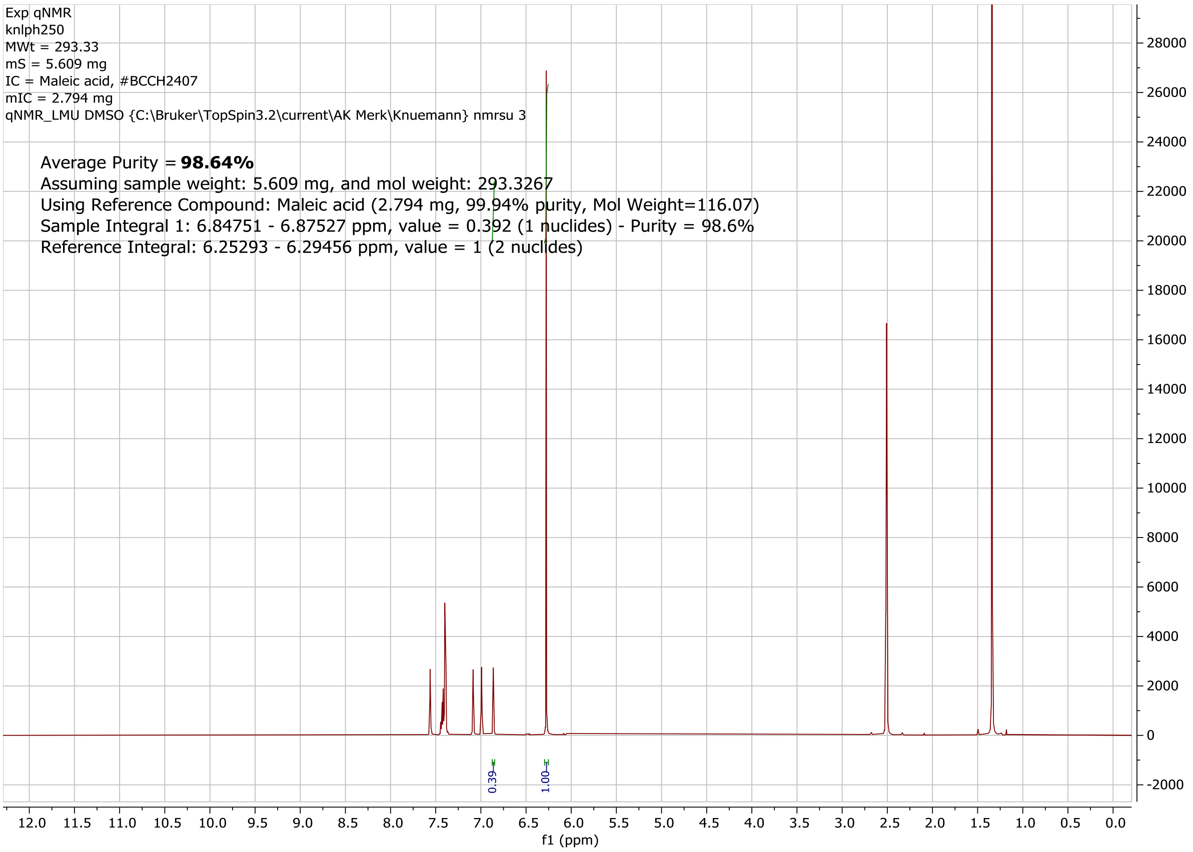

Compound **59**:

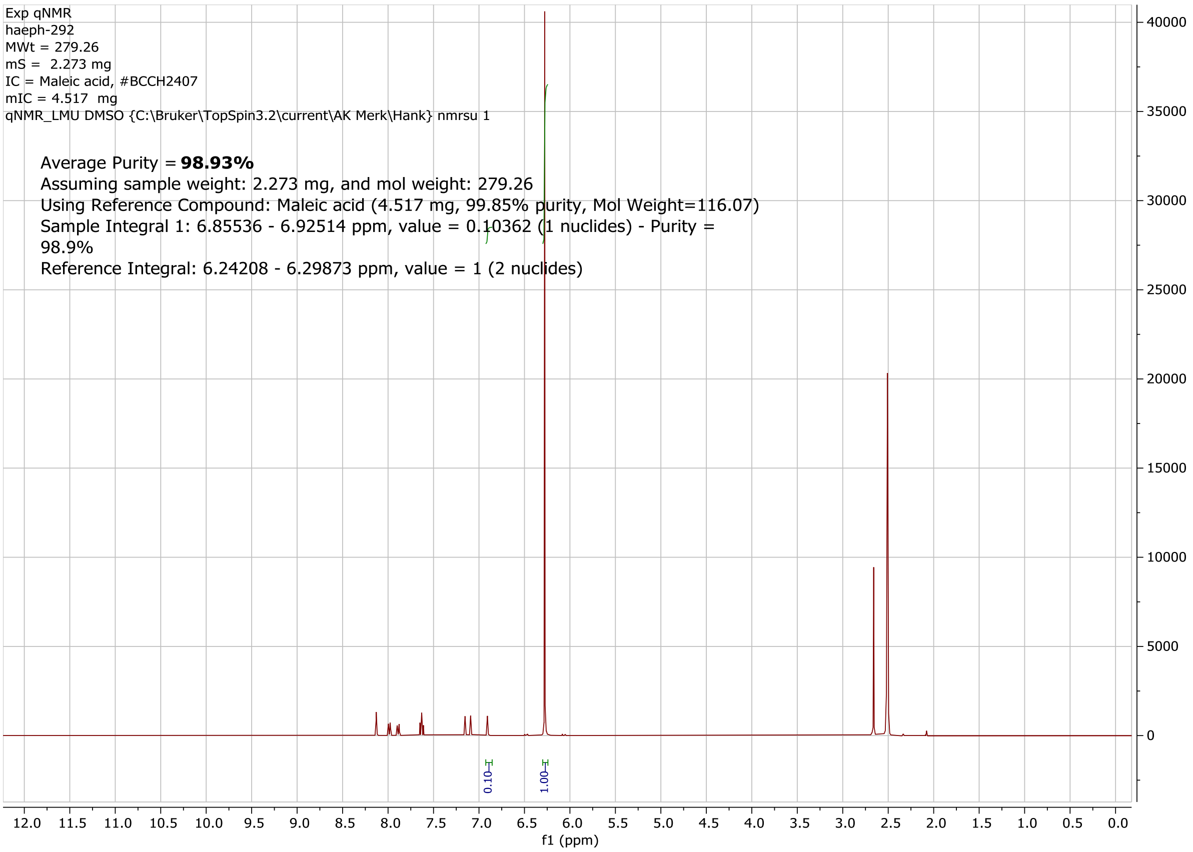

Compound **60**:

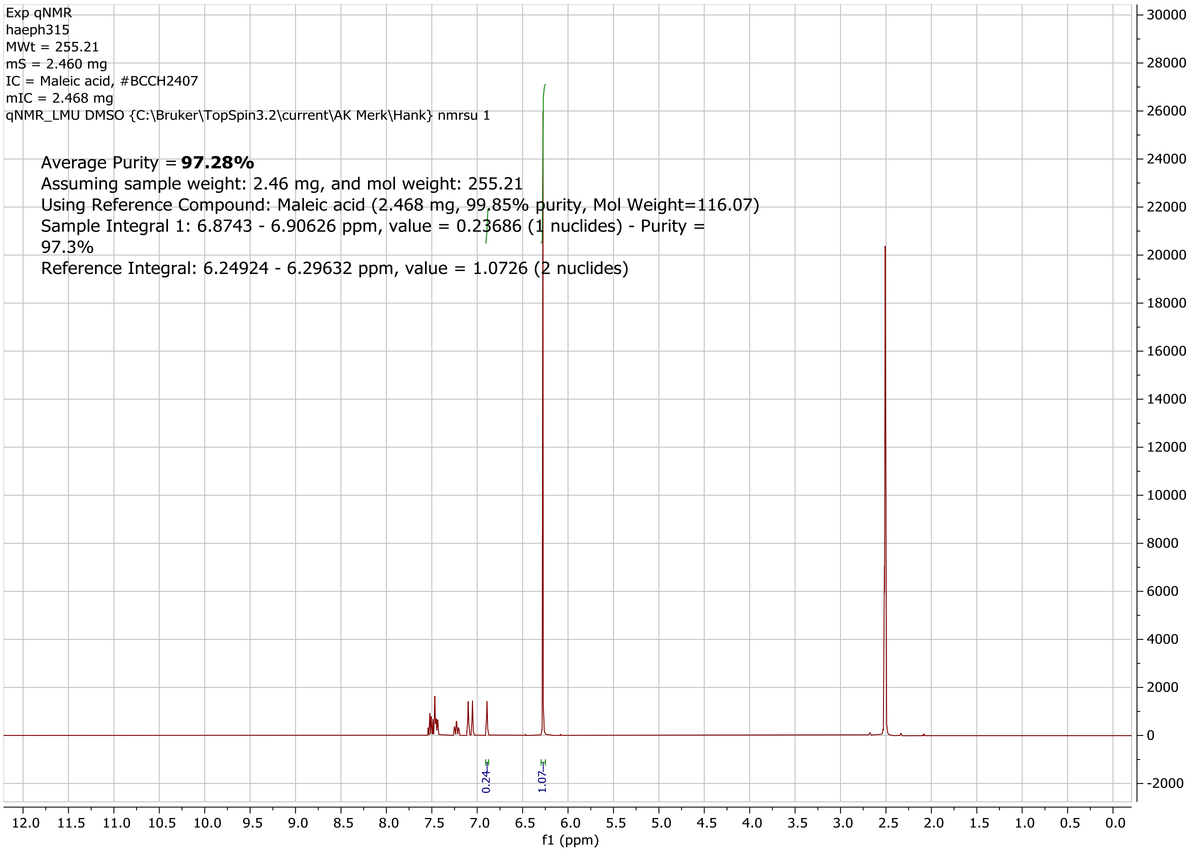

Compound **61**:

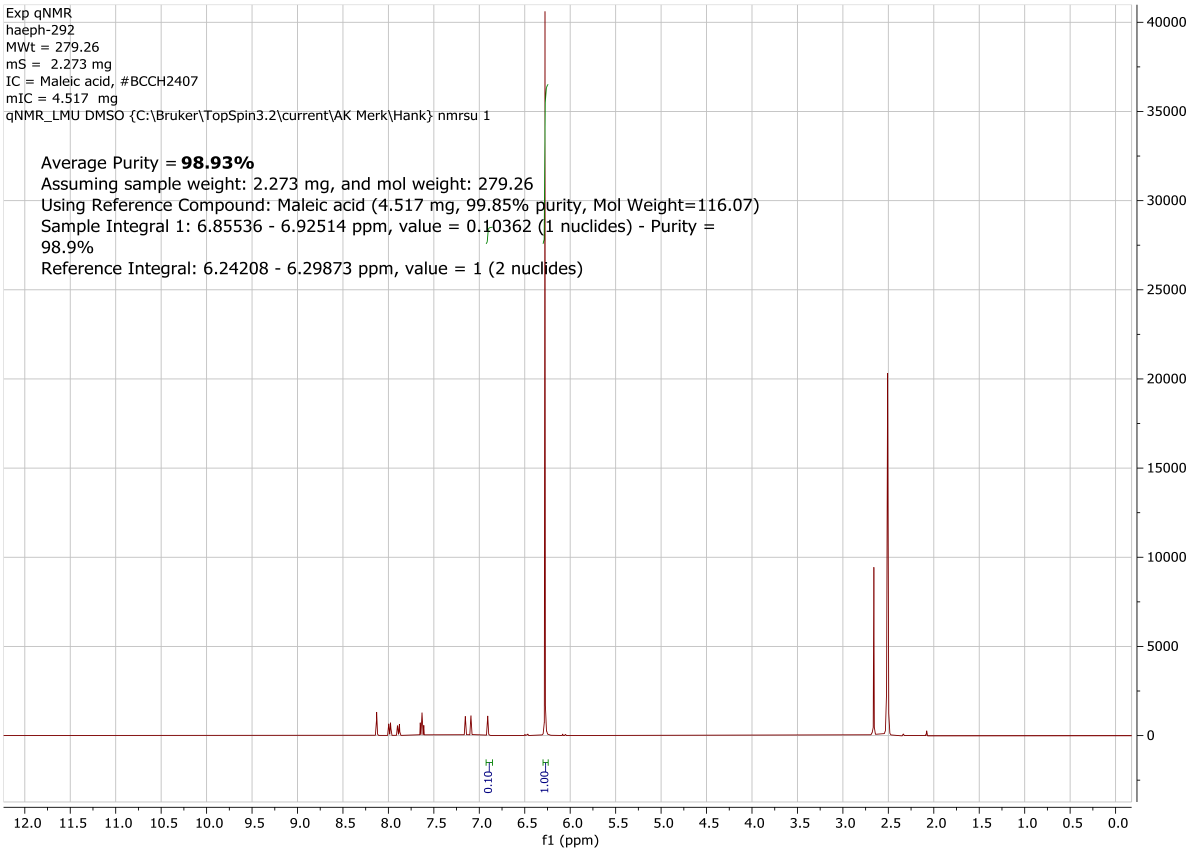

Compound **62**:

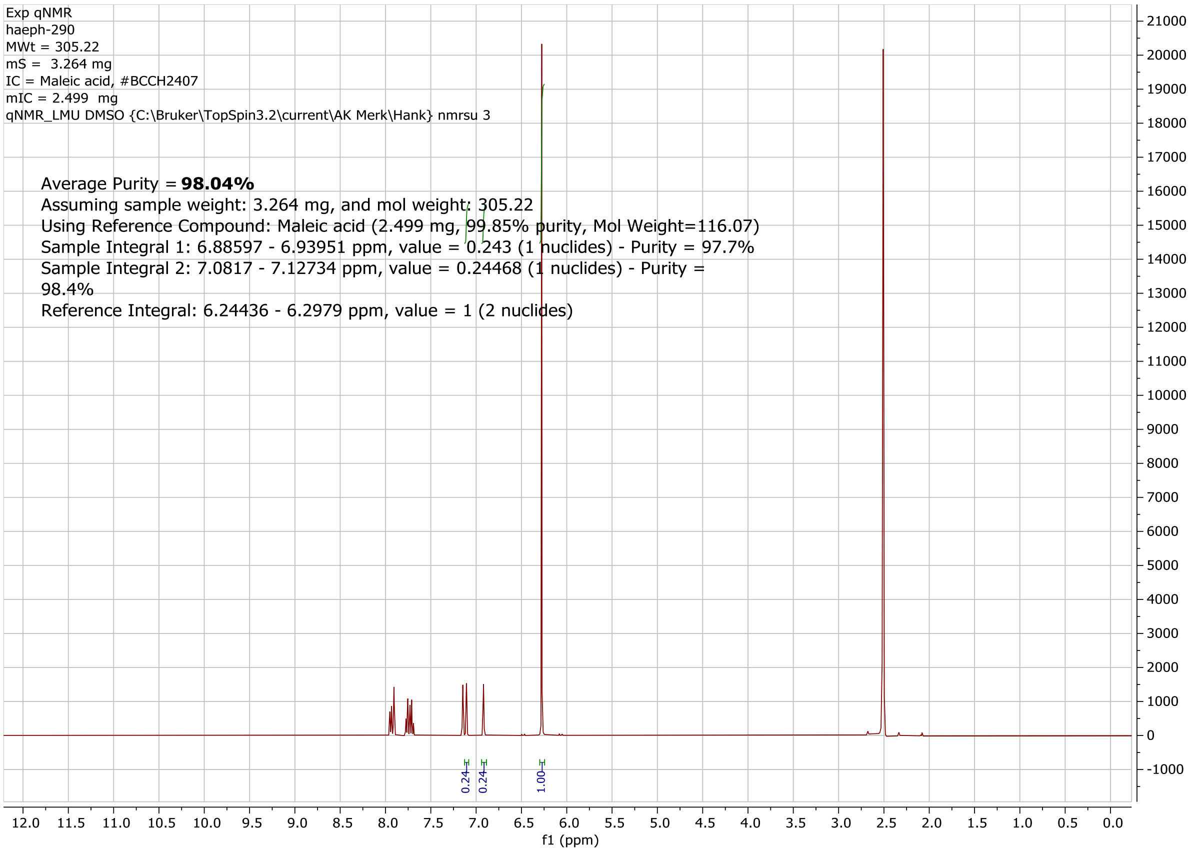

Compound **63**:

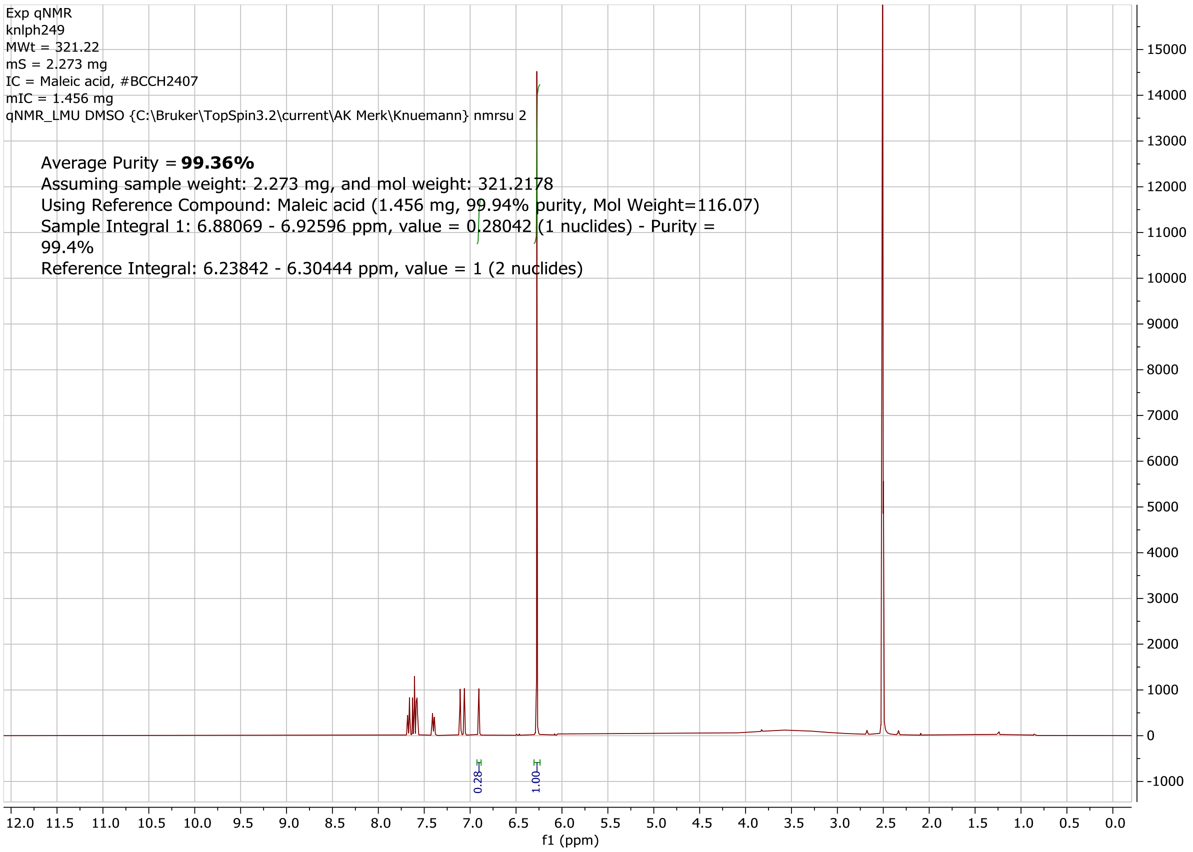

Compound **64**:

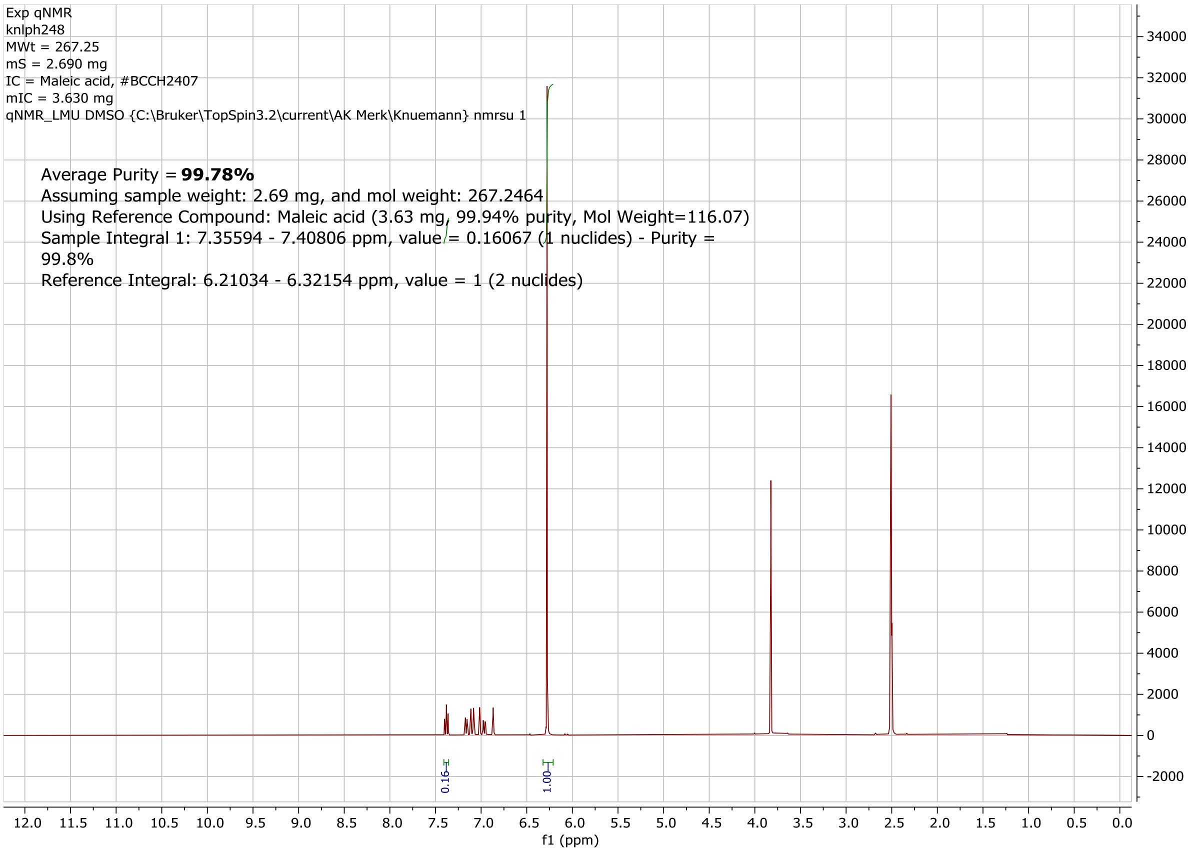

Compound **65**:

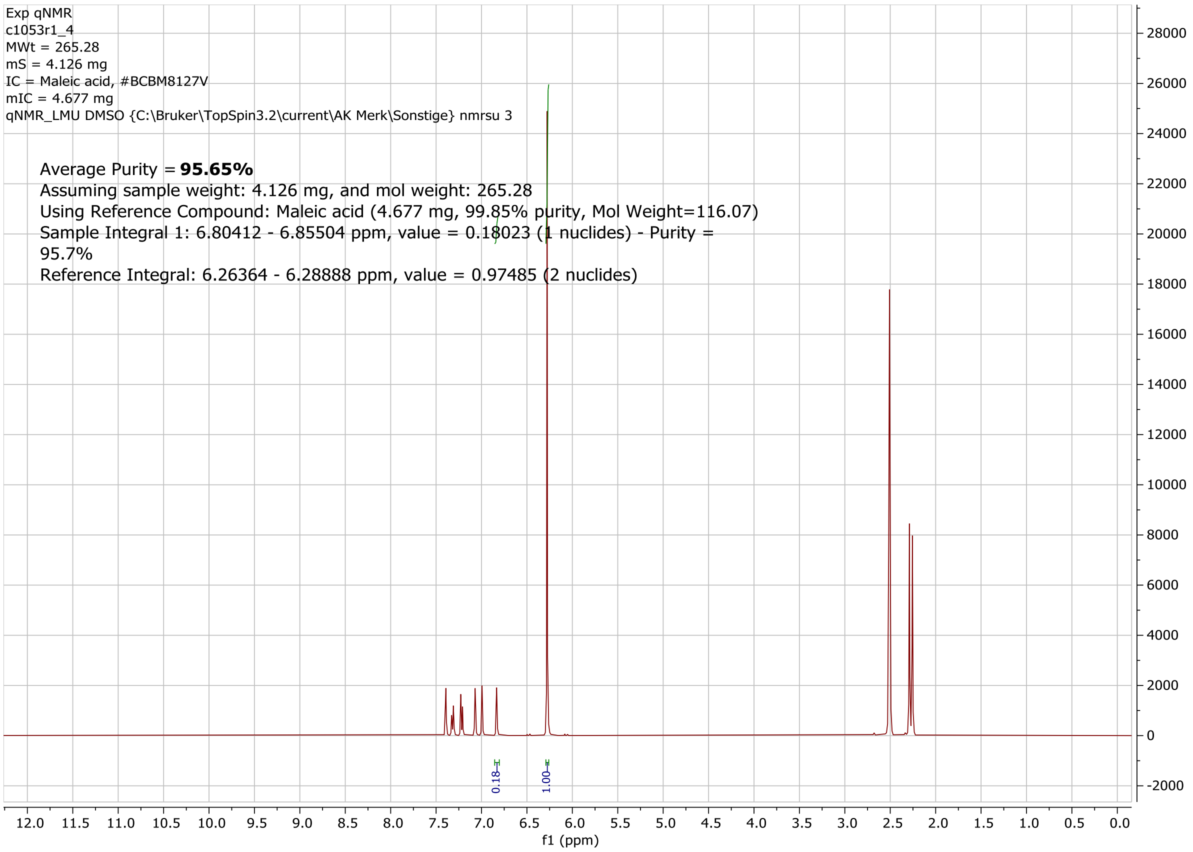

Compound **66**:

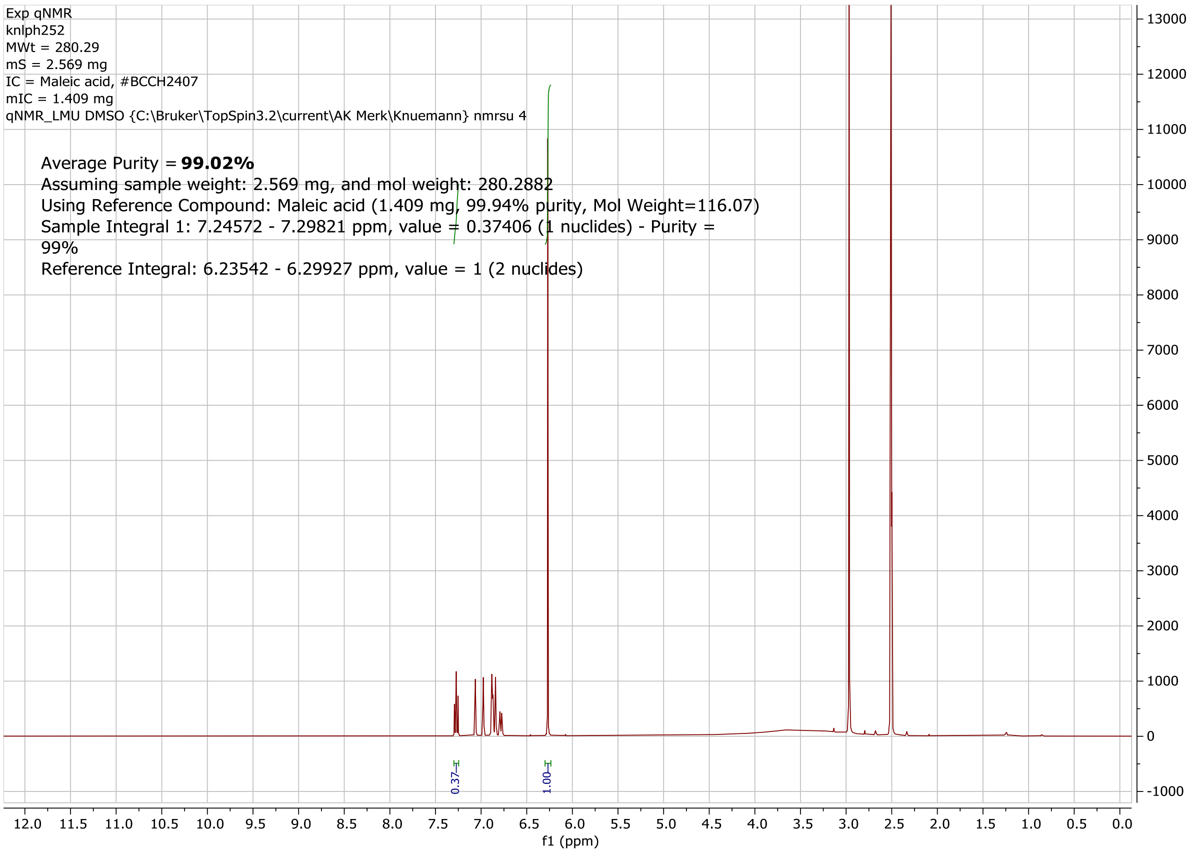

Compound **67**:

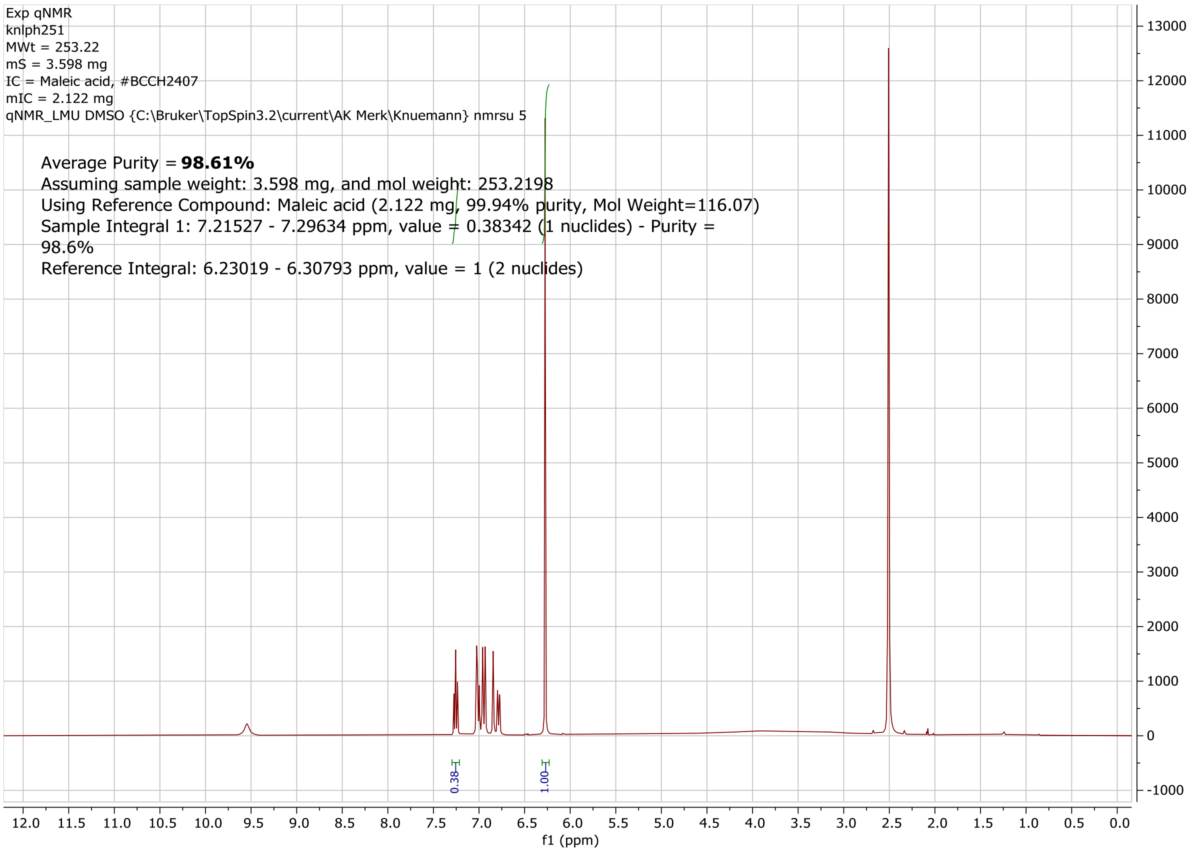

Compound **68**:

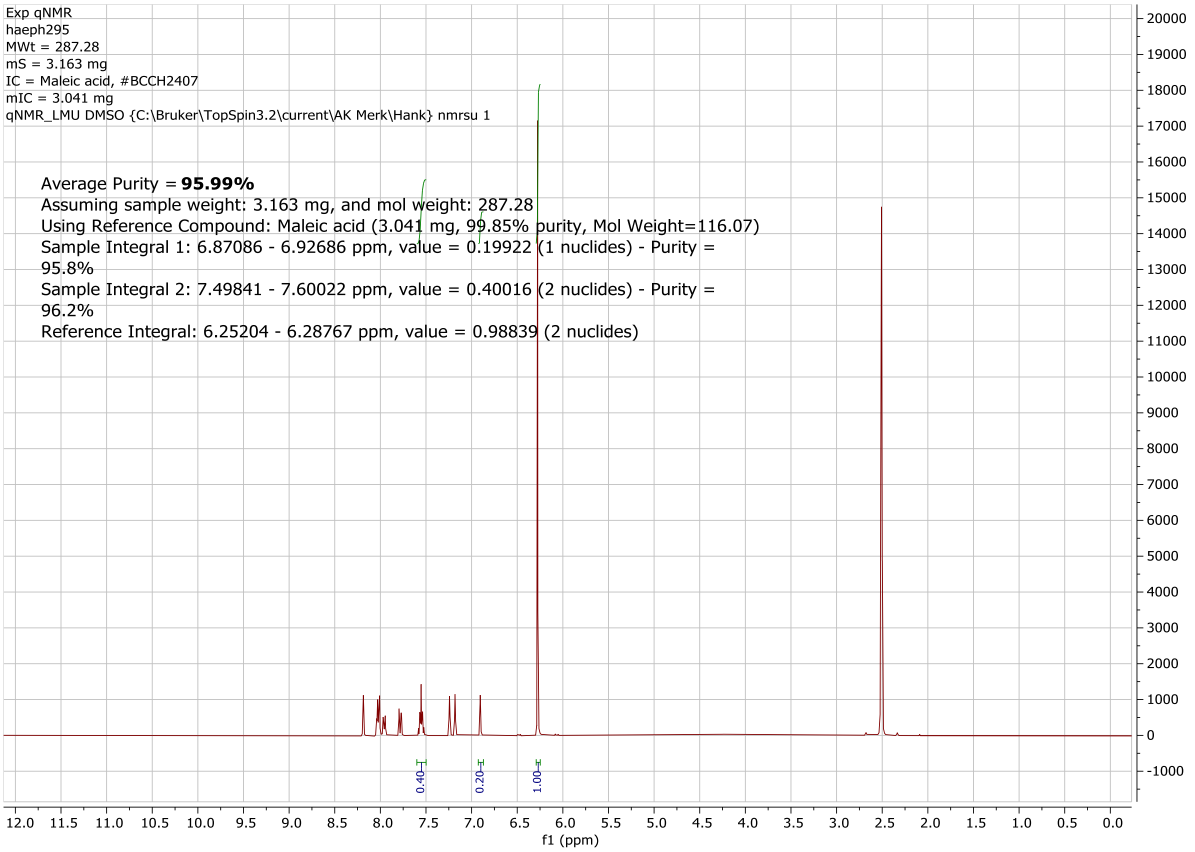

Compound **69**:

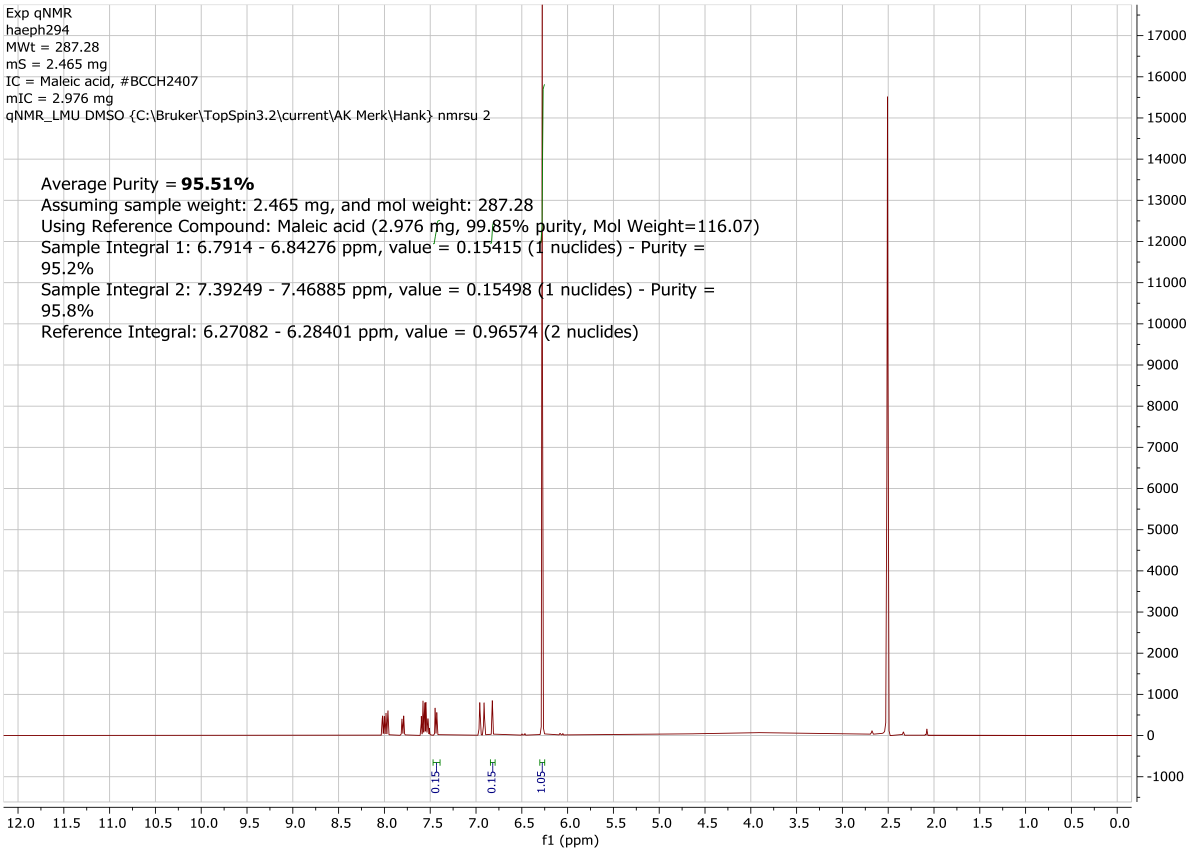

Compound **70**:

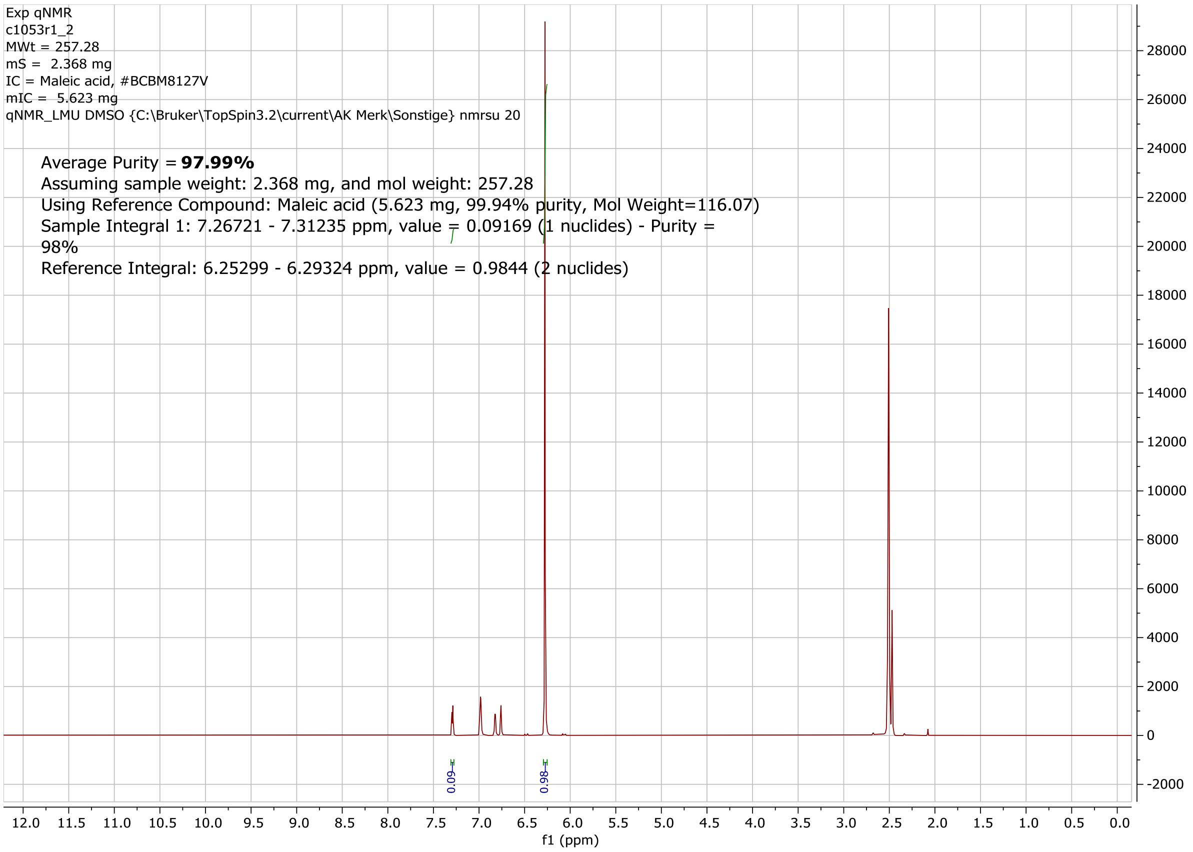

Compound **71**:

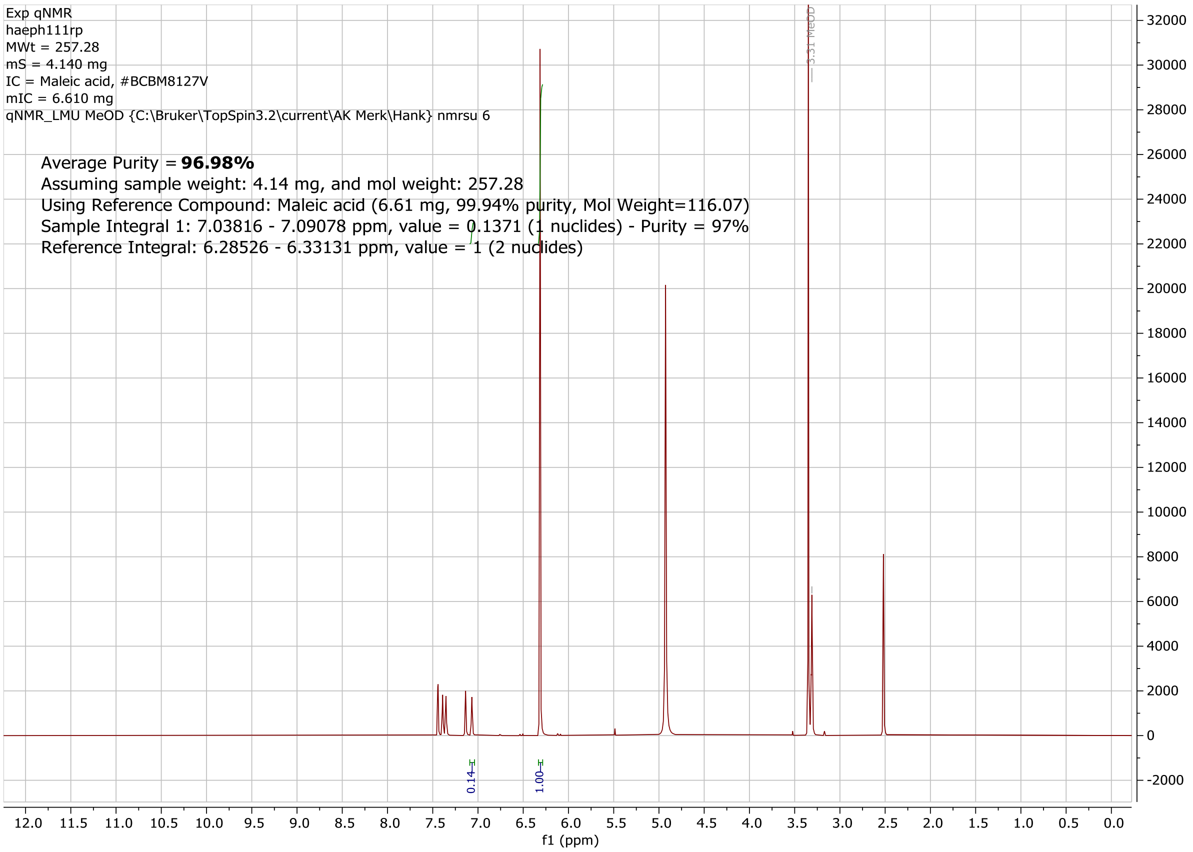

Compound **72**:

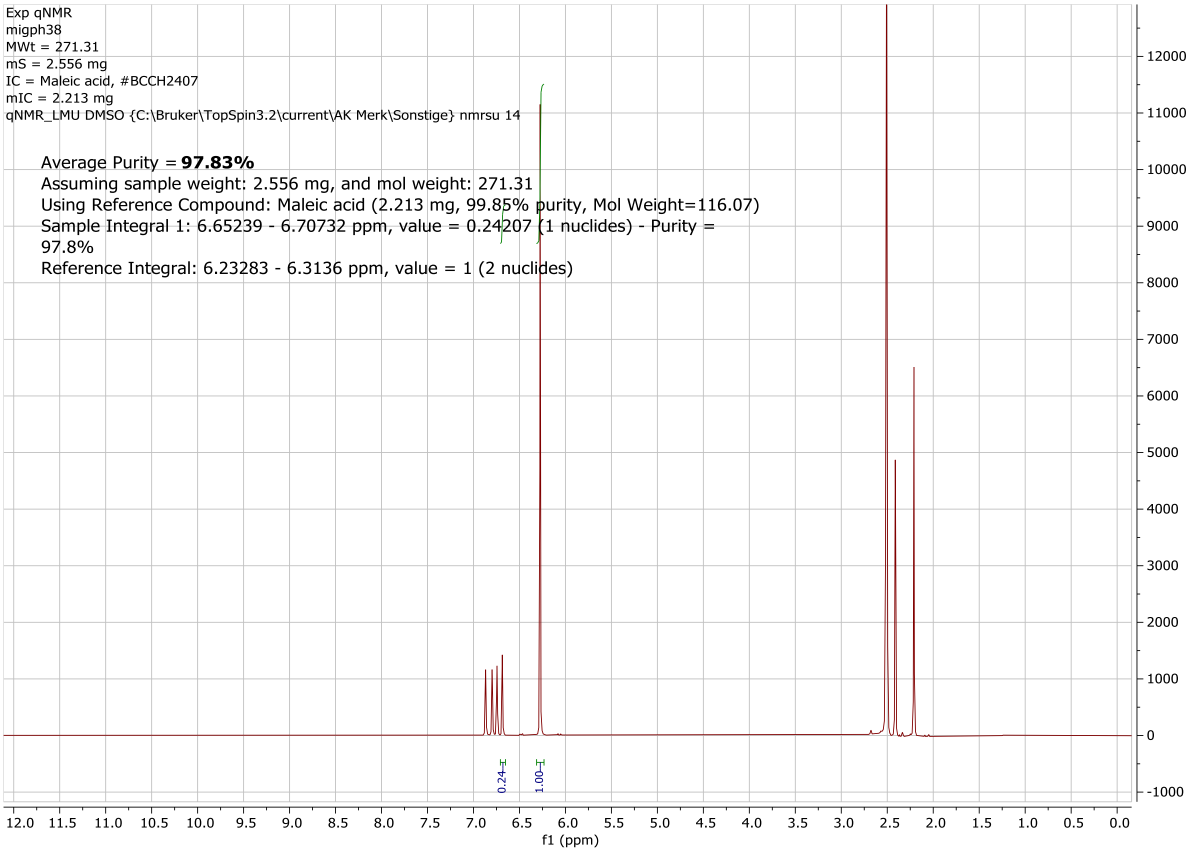

Compound **73**:

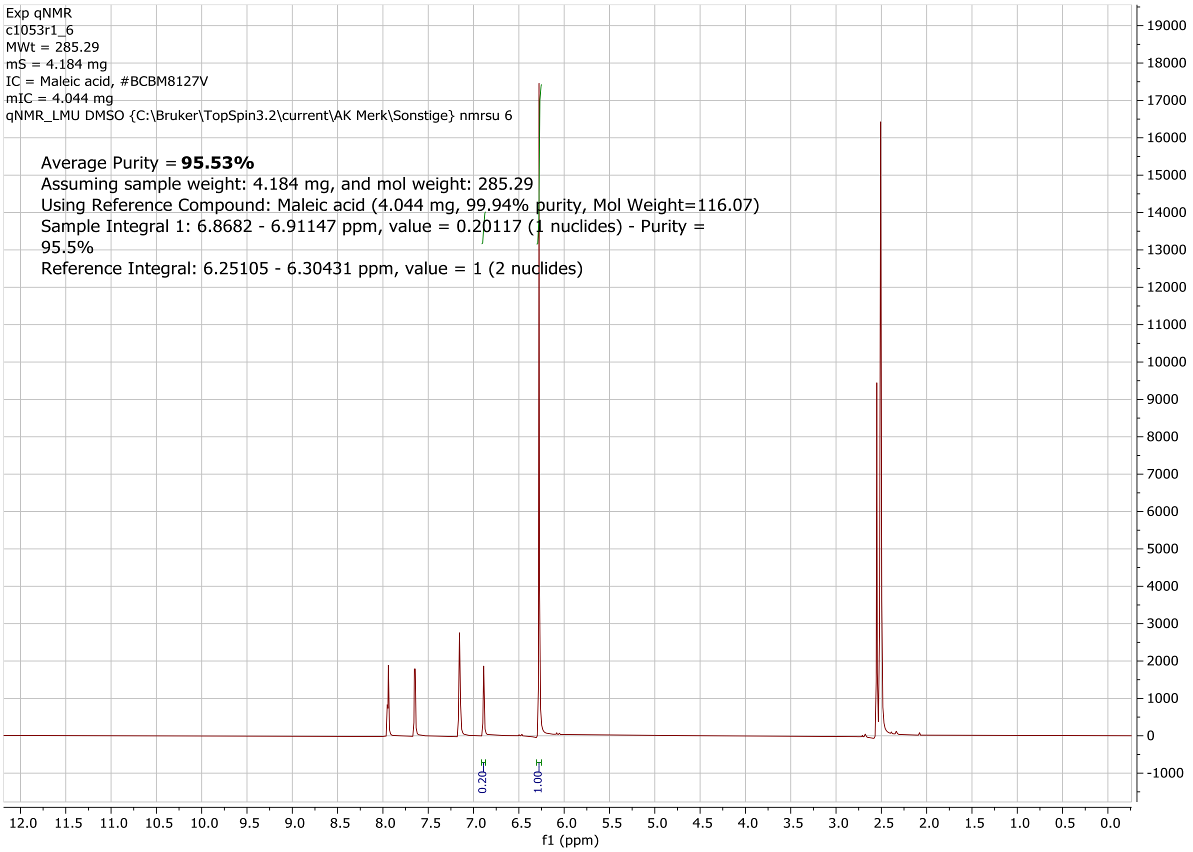

Compound **74**:

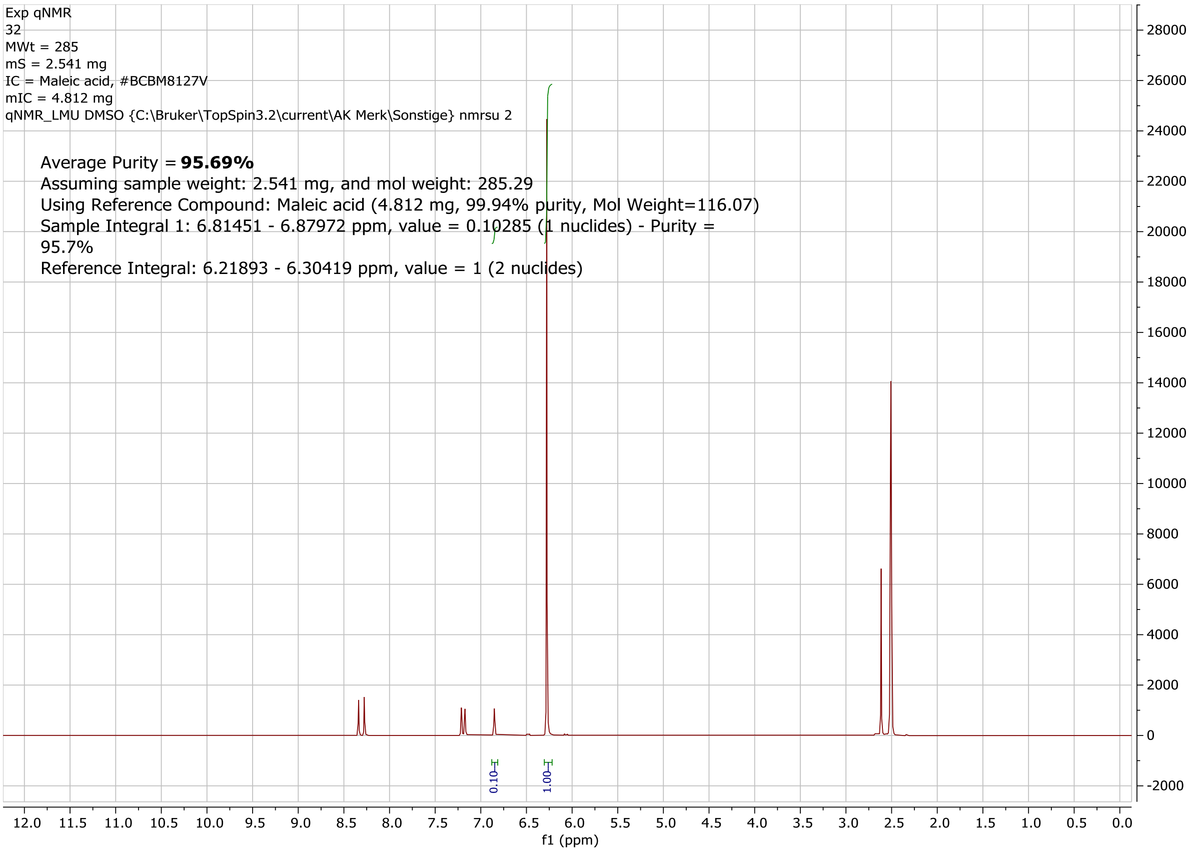

Compound **75**:

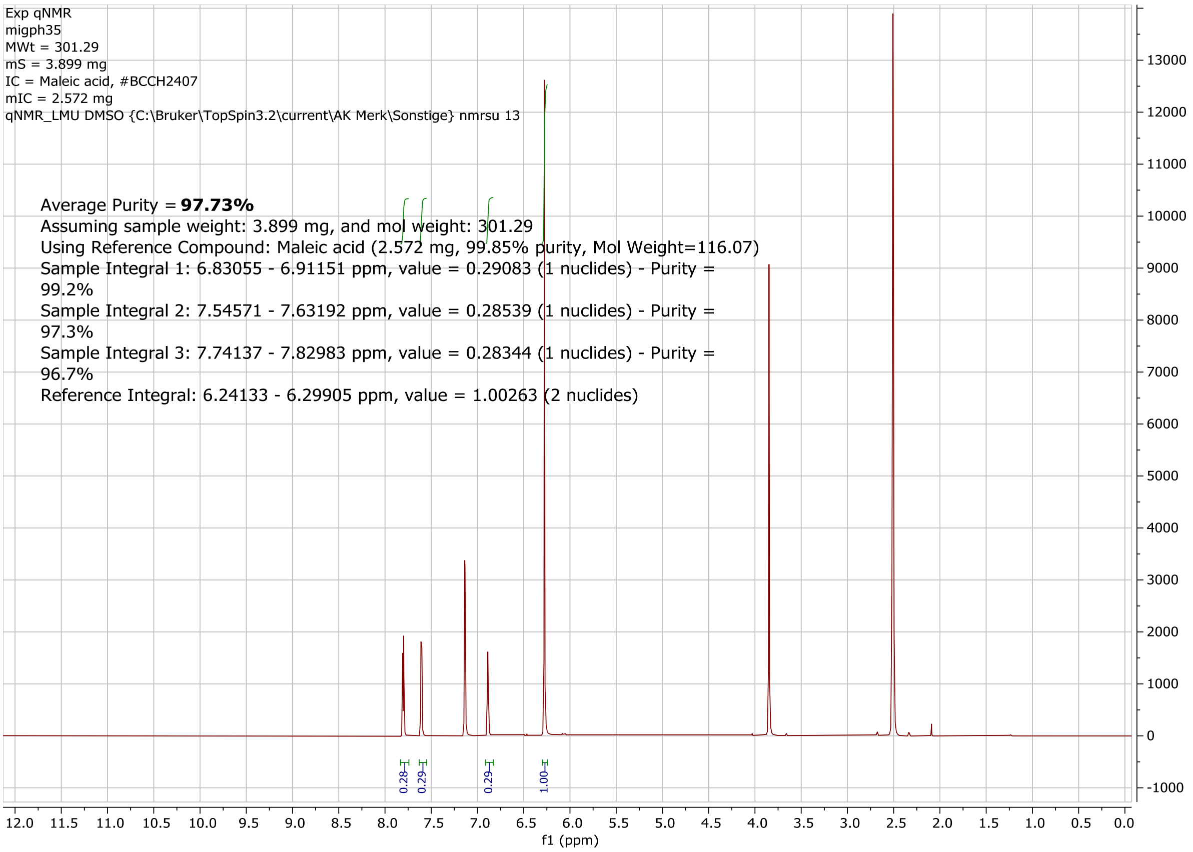

Compound **76**:

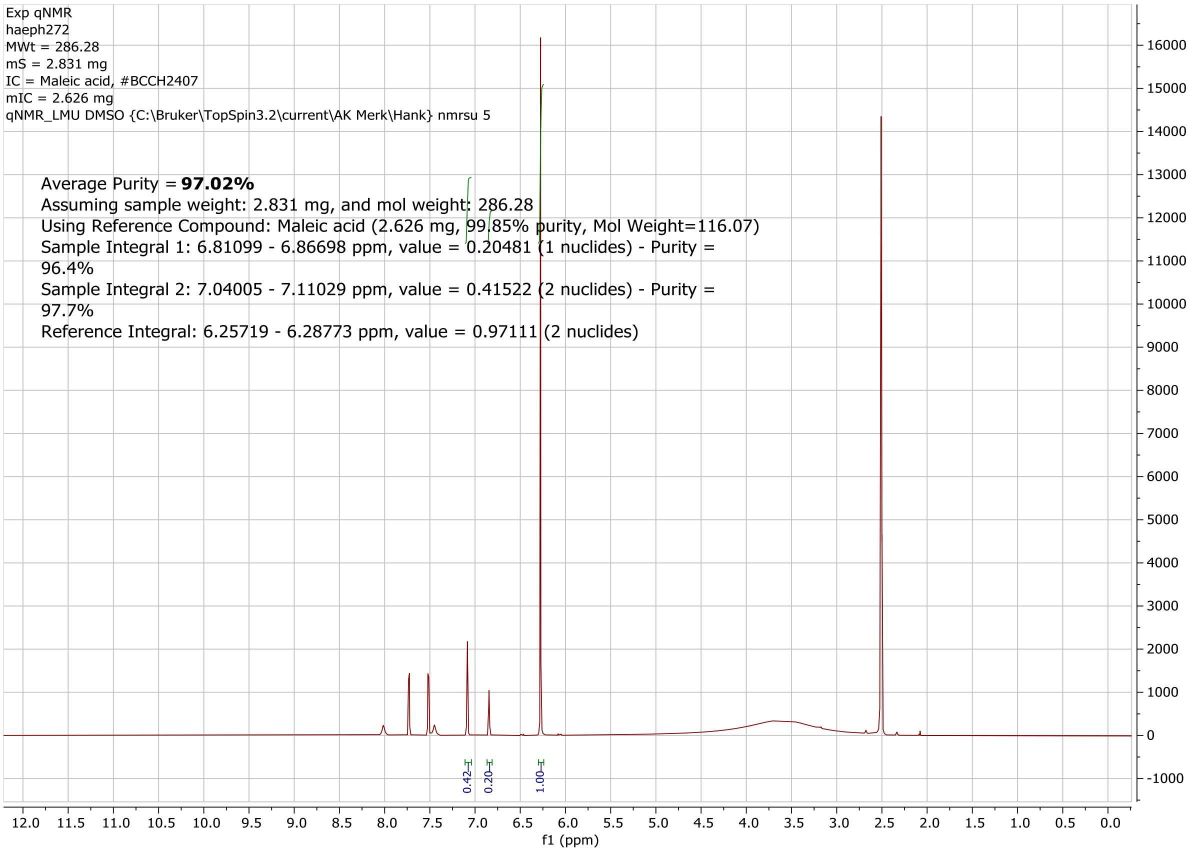

Compound **77**:

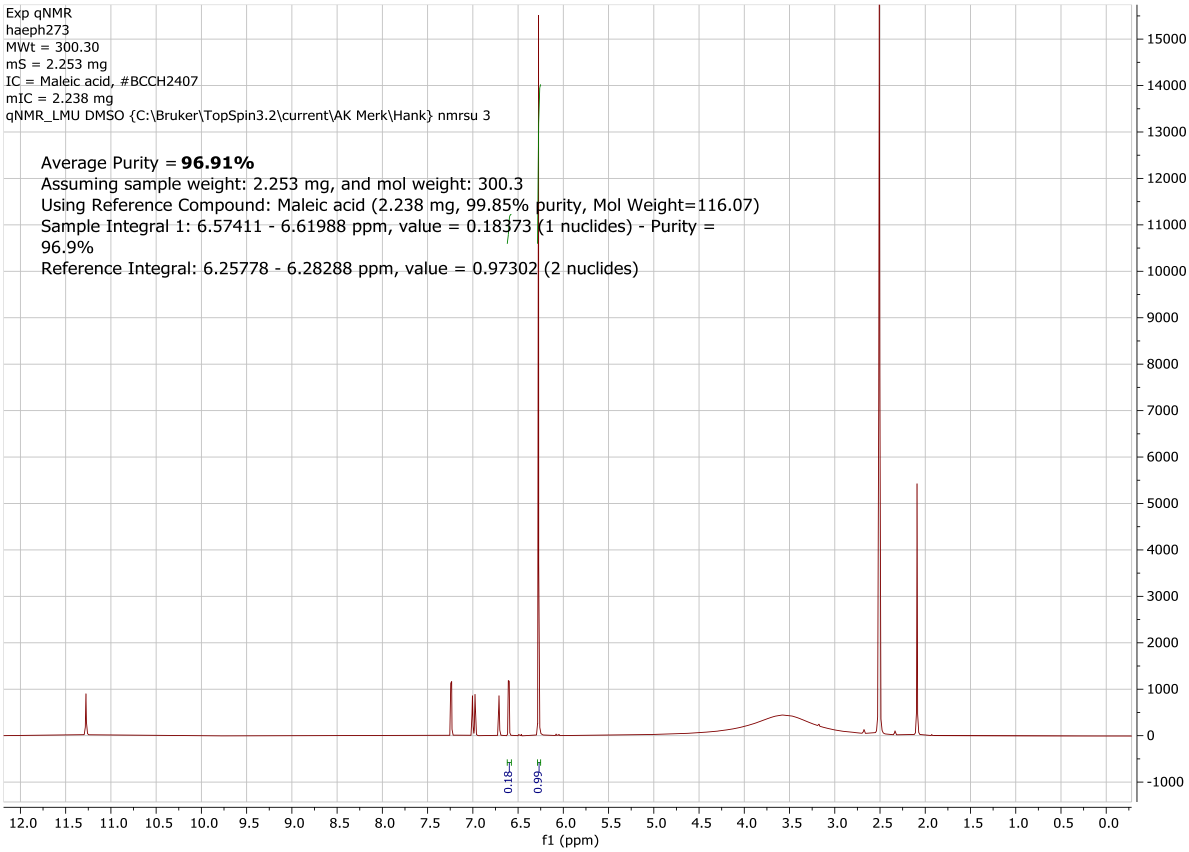

Compound **78**:

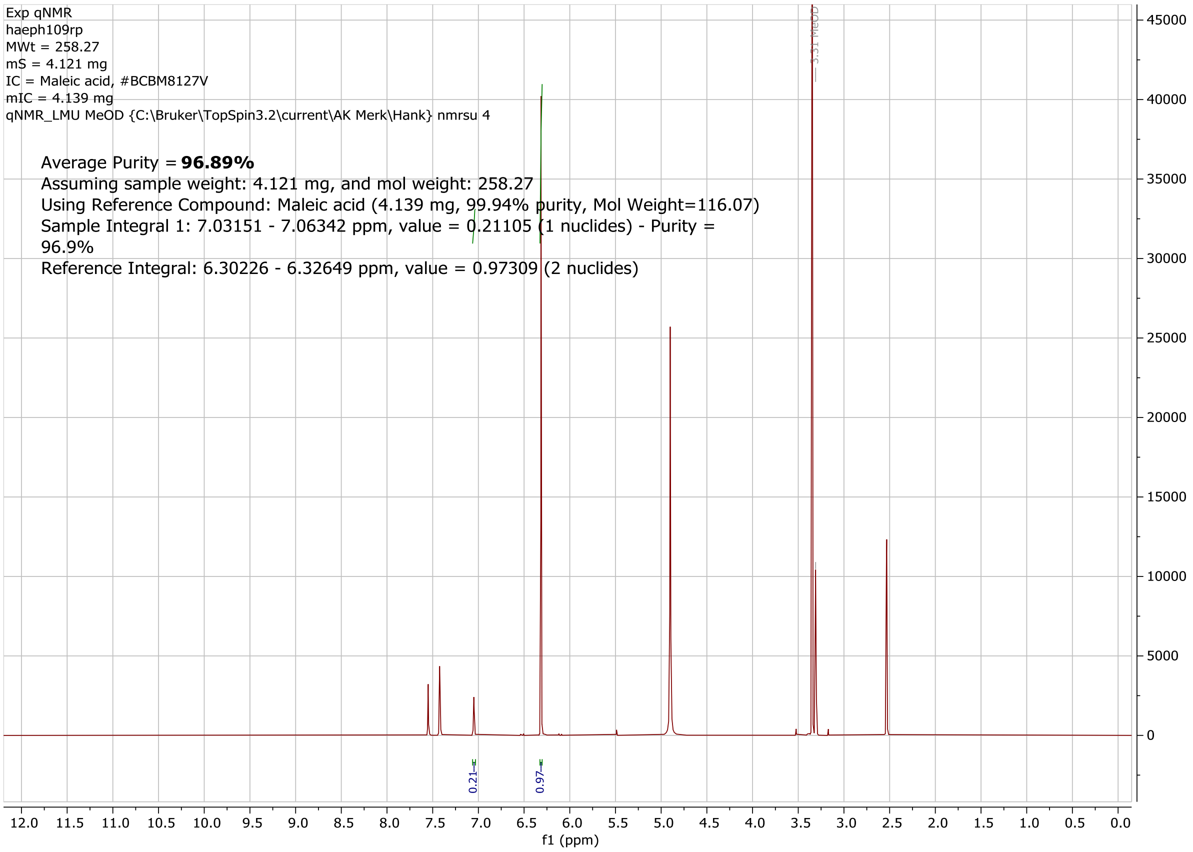

Compound **79**:

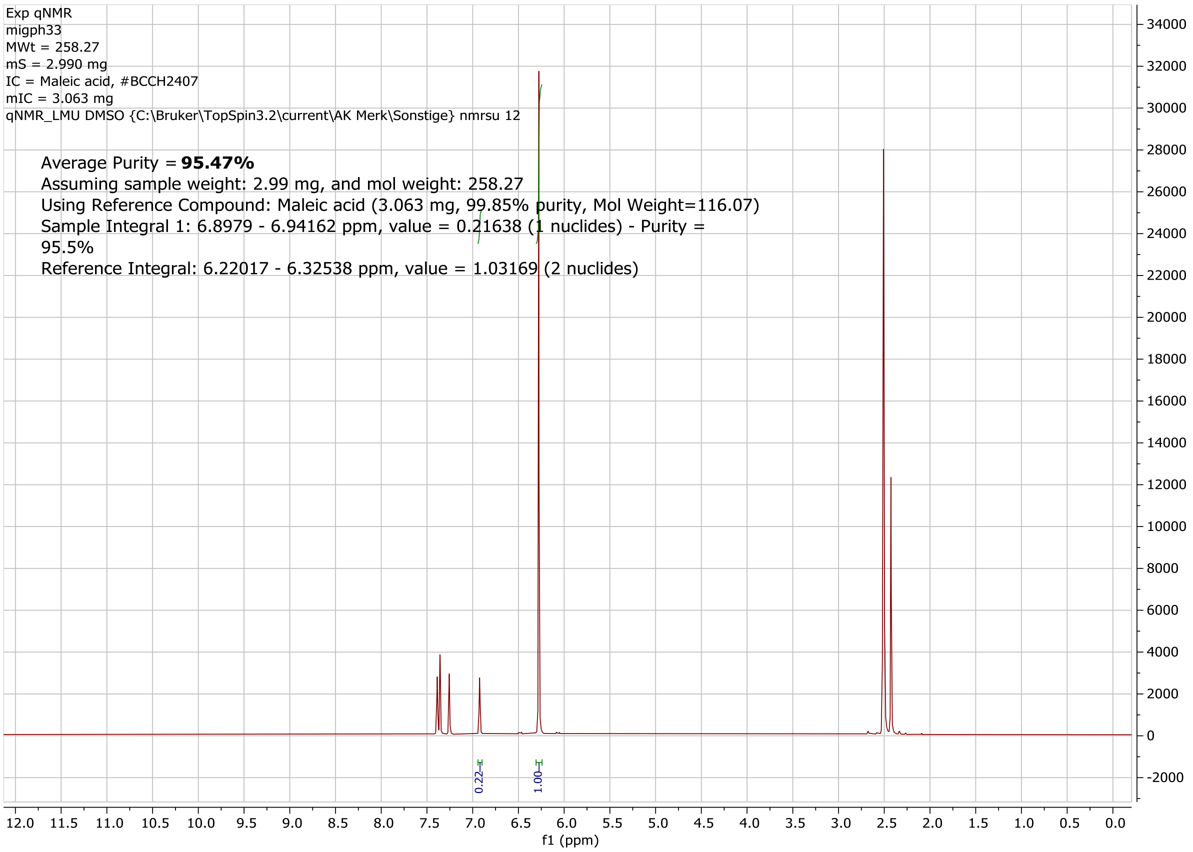

Compound **80**:

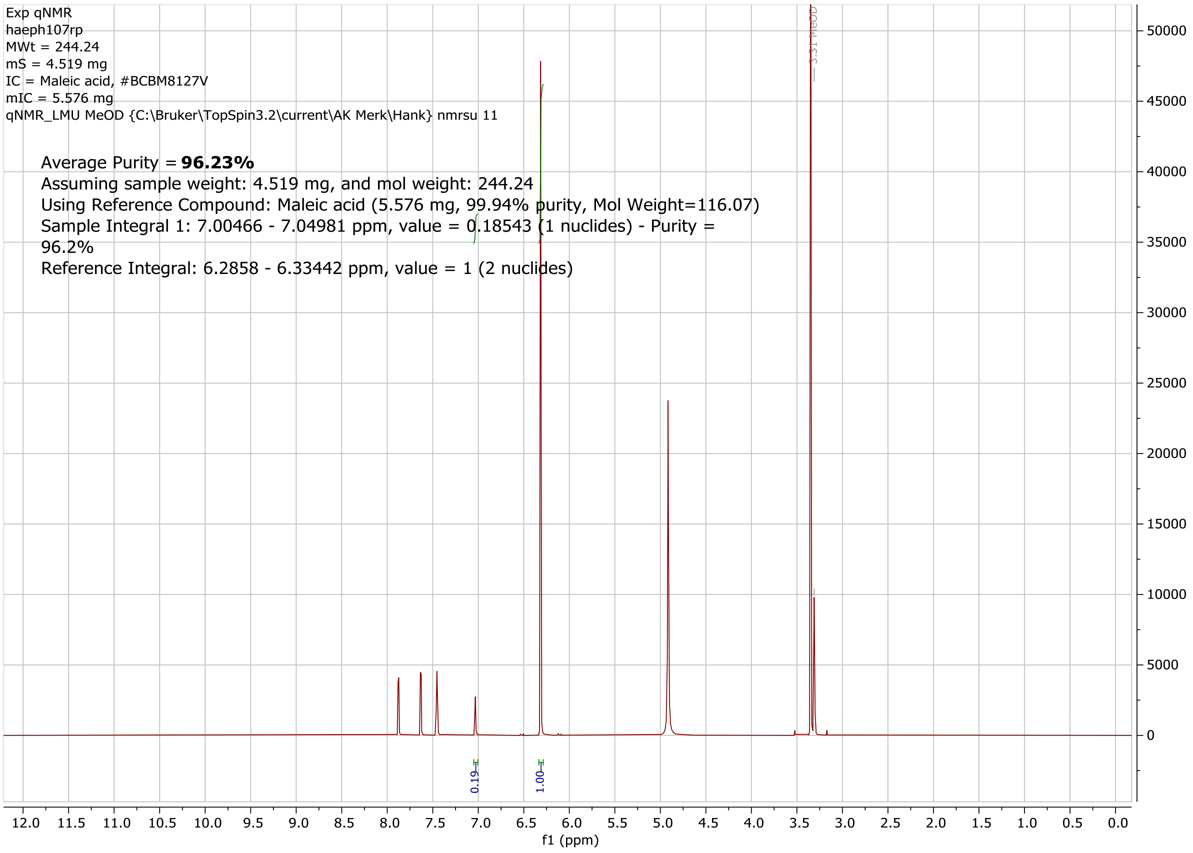

Exp qNMR
haeph107rp
MWt = 244.24
mS = 4.519 mg
IC = Maleic acid, #BCBM8127V
mIC = 5.576 mg
qNMR_LMU MeOD {C:\Bruker\TopSpin3.2\current\AK Merk\Hank} nmrsu 11
Average Purity = **96.23%**
Assuming sample weight: 4.519 mg, and mol weight: 244.24
Using Reference Compound: Maleic acid (5.576 mg, 99.94% purity, Mol Weight=116.07)
Sample Integral 1: 7.00466 - 7.04981 ppm, value = 0.18543 (1 nuclides) - Purity = 96.2%
Reference Integral: 6.2858 - 6.33442 ppm, value = 1 (2 nuclides)
3.31 MeOD
0.19
1.00
f1 (ppm)

Supplementary References

(1) Hank, E. C.; Sai, M.; Kasch, T.; Meijer, I.; Marschner, J. A.; Merk, D. Development of Tailless Homologue Receptor (TLX) Agonist Chemical Tools. *J. Med. Chem.* **2024**, *67* (18), 16598–16611.

(2) Faudone, G.; Bischoff-Kont, I.; Rachor, L.; Willems, S.; Zhubi, R.; Kaiser, A.; Chaikuad, A.; Knapp, S.; Fürst, R.; Heering, J.; Merk, D. Propranolol Activates the Orphan Nuclear Receptor TLX to Counteract Proliferation and Migration of Glioblastoma Cells. *J. Med. Chem.* **2021**, *64* (12), 8727–8738.

(3) Nawa, F.; Kardanov, A.; Kasch, T.; Lewandowski, M.; Wein, T.; Höfner, G.; Marschner, J. A.; Morozov, V.; Merk, D. Development of an RXR Agonist Scaffold with Pronounced Homodimer Preference. *J. Med. Chem.* **2025**, *68* (15), 16172–16187.

(4) Vietor, J.; Gege, C.; Stiller, T.; Busch, R.; Schallmayer, E.; Kohlhof, H.; Höfner, G.; Pabel, J.; Marschner, J. A.; Merk, D. Development of a Potent Nurr1 Agonist Tool for In Vivo Applications. *J. Med. Chem.* **2023**, *66* (9), 6391–6402.
